# Highly efficient Ir-catalyzed asymmetric hydrogenation of benzoxazinones and derivatives with a Brønsted acid cocatalyst†
†Electronic supplementary information (ESI) available: See DOI: 10.1039/c8sc05797d


**DOI:** 10.1039/c8sc05797d

**Published:** 2019-03-19

**Authors:** Zhengyu Han, Gang Liu, Rui Wang, Xiu-Qin Dong, Xumu Zhang

**Affiliations:** a Key Laboratory of Biomedical Polymers , Engineering Research Center of Organosilicon Compounds & Materials , Ministry of Education , College of Chemistry and Molecular Sciences , Wuhan University , Wuhan , Hubei 430072 , P. R. China . Email: xiuqindong@whu.edu.cn; b Department of Chemistry , Shenzhen Grubbs Institute , Southern University of Science and Technology , Shenzhen , Guangdong 518055 , P. R. China . Email: zhangxm@sustc.edu.cn

## Abstract

The Ir-catalyzed highly efficient asymmetric hydrogenation of benzoxazinones and derivatives was successfully developed with *N*-methylated ZhaoPhos **L5** as the ligand, which may display a new activation mode with a single anion-binding interaction among the substrate, cocatalyst Brønsted acid and ligand. This synthetic approach afforded a series of chiral dihydrobenzoxazinones and derivatives with excellent results (>99% conversion, 88–96% yields, 91–>99% ee, up to 40 500 TON). A key to success is the utilization of a strong Brønsted acid as the cocatalyst, such as hydrochloric acid, to form a possible single anion-binding interaction with the substrate and catalyst, which greatly contributed to the improvement of reactivity and enantioselectivity. Importantly, a creative and efficient synthetic route was developed to construct the important intermediate for the potential IgE/IgG receptor modulator through our catalytic methodology system.

## Abstract 2

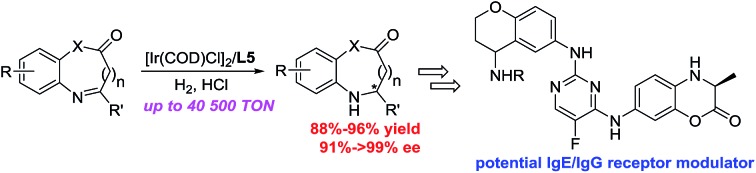
The Ir-catalyzed highly efficient asymmetric hydrogenation of benzoxazinones and derivatives was successfully developed with *N*-methylated ZhaoPhos **L5** as the ligand, affording various chiral dihydrobenzoxazinones and derivatives with excellent results.

## Introduction

**Fig. 1:**
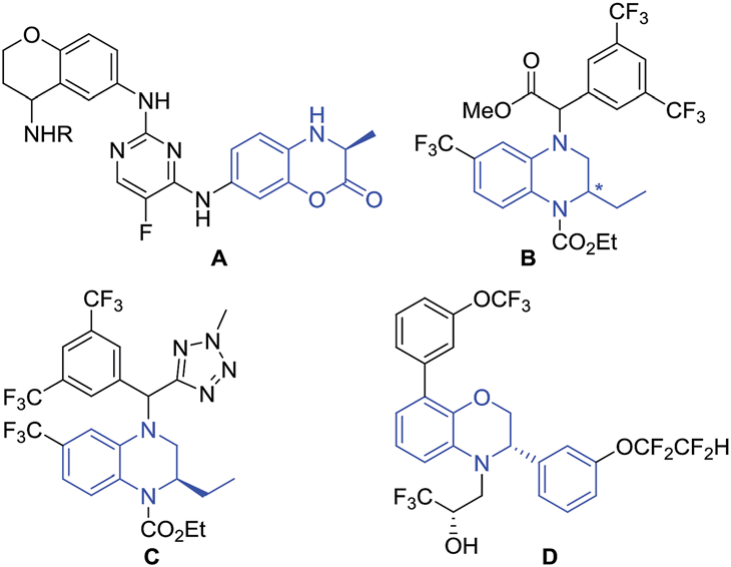
Selected examples of bioactive compounds containing the framework of chiral dihydrobenzoxazinones and derivatives.

Chiral dihydrobenzoxazinones and derivatives are important and unique building blocks in the biologically active molecule discovery process (Fig. 1).^1^ Chiral dihydrobenzoxazinone derivatives **A** are potential IgE/IgG receptor modulators for the treatment of autoimmune diseases.^2^ Chiral 1,2,3,4-tetrahydroquinoxaline compounds **B–C** and 2,3-dihydro-3,8-diphenylbenzo[1,4]oxazine **D** are disclosed as active and promising cholesteryl ester transfer protein inhibitors.^3^–^6^

**Scheme 1:**
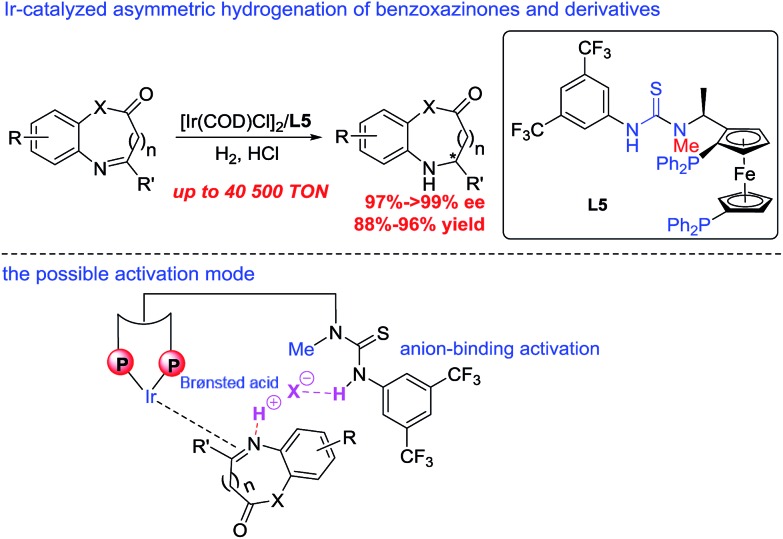
Ir-catalyzed asymmetric hydrogenation of benzoxazinones and derivatives, and the possible activation mode.

Taking into account the growing importance of these compounds, great attention had been paid to the development of efficient enantioselective synthetic methodologies. Among various synthetic approaches for the construction of these chiral dihydrobenzoxazinones and derivatives,^7^ the direct asymmetric hydrogenation of prochiral benzoxazinones and derivatives was paid great attention with the advantages of high atom economy, a relatively simple procedure and easy work-up. Zhou and co-workers realized Ru-catalyzed biomimetic asymmetric hydrogenation of benzoxazinones and derivatives in the presence of chiral phosphoric acid through the NAD(P)H mode.7*c* In 2015, Beller and co-workers described asymmetric hydrogenation of benzoxazinones *via* a relay iron/chiral Brønsted acid catalysis with good to excellent enantioselectivities.7*e* However, the reactivity of most catalytic systems is not very high with less than 2000 TON (turnover numbers). It is well known that the substrate activation strategy with noncovalent interactions has been widely used in the field of asymmetric organic catalysis, which played an important role in greatly improving the reactivity and stereoselectivity.^8^,^9^ The thiourea motif as the hydrogen bonding donor can recognize suitable guest molecules, which usually works on the direct activation of neutral substrates bearing hydrogen bonding acceptor groups.^10^ Compared with the hydrogen bonding interaction, the anion-binding ion-pairing strategy did not draw much attention until recent development in organocatalysis.9b–d,^11^ In 2013, our group successfully developed a series of bifunctional bisphosphine–thiourea ligands, which extended the powerful hydrogen-bonding and anion-binding activation strategy in organocatalysis to transition-metal-catalyzed asymmetric hydrogenation, and a variety of functionalized substrates had been hydrogenated well.^12^ Herein, we successfully realized Ir-catalyzed highly enantioselective hydrogenation of prochiral benzoxazinones and derivatives using *N*-methylated ZhaoPhos **L5** as the ligand, which may exhibit a single anion-binding activation mode among the substrate, cocatalyst Brønsted acid hydrochloric acid and ligand. A variety of hydrogenation products, chiral dihydrobenzoxazinones and derivatives, can be obtained with excellent results (>99% conversion, 88–96% yields, 91–>99% ee), and our catalytic system displayed extremely high activity with up to 40 500 TON (Scheme 1). In addition, a highly efficient synthetic route was successfully developed to prepare the important intermediate for the potential IgE/IgG receptor modulator with our asymmetric hydrogenation methodology as the key reaction step.

## Results and discussion

Inspired by the powerful performance of the ligand ZhaoPhos **L1** in Rh-catalyzed asymmetric hydrogenation of iminium salts, isoquinoline hydrochloride and N-unprotected indoles,12b,d,e,g we initially investigated the hydrogenation of model substrate 3-phenyl-2*H*-benzo[*b*][1,4]oxazin-2-one **1a** catalyzed by Rh(NBD)_2_BF_4_/ZhaoPhos **L1** in toluene with 1.0 equiv. HCl (4 M in dioxane) as the additive, affording the hydrogenation product **2a** with 63% conversion and 82% ee (Table 1, entry 1). Other metal precursors were then screened, and [Ir(COD)Cl]_2_ was proved to be the best with 90% conversion and 94% ee (Table 1, entry 3). Solvents played an important role in asymmetric reactions, and always affected the reactivity and enantioselectivity. This asymmetric hydrogenation was then conducted in different solvents. Moderate to high conversions and excellent enantioselectivities were observed in CH_2_Cl_2_, tetrahydrofuran (THF), 1,4-dioxane, CHCl_3_, ethyl acetate (EA) and CH_3_CN (55–>99% conversions, 93–98% ee, Table 1, entries 4–9). And the hydrogenation product **2a** can be obtained with the best result in THF (>99% conversion, 98% ee, Table 1, entry 5).

**Table 1:** Optimization of reaction conditions for asymmetric hydrogenation of 3-phenyl-2*H*-benzo[*b*][1,4]oxazin-2-one (**1a**)^*a*^

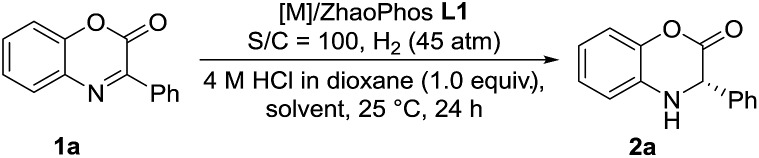				
Entry	Metal precursor	Solvent	Conv.^*b*^ (%)	ee^*c*^ (%)
1	Rh(NBD)_2_BF_4_	Toluene	63	82
2	[Rh(COD)Cl]_2_	Toluene	71	64
3	[Ir(COD)Cl]_2_	Toluene	90	94
4	[Ir(COD)Cl]_2_	CH_2_Cl_2_	97	93
5	[Ir(COD)Cl]_2_	THF	>99	98
6	[Ir(COD)Cl]_2_	1,4-Dioxane	60	95
7	[Ir(COD)Cl]_2_	CHCl_3_	95	95
8	[Ir(COD)Cl]_2_	Ethyl acetate	95	93
9	[Ir(COD)Cl]_2_	CH_3_CN	55	94

^*a*^Reaction conditions: 0.05 mmol **1a** in 1.0 mL solvent, S/C = 100, 45 atm H_2_, 1.0 equiv. HCl (4 M in dioxane), 25 °C, 24 h.

^*b*^Determined by ^1^H NMR analysis.

^*c*^Determined by HPLC analysis using a chiral stationary phase.

**Fig. 2:**
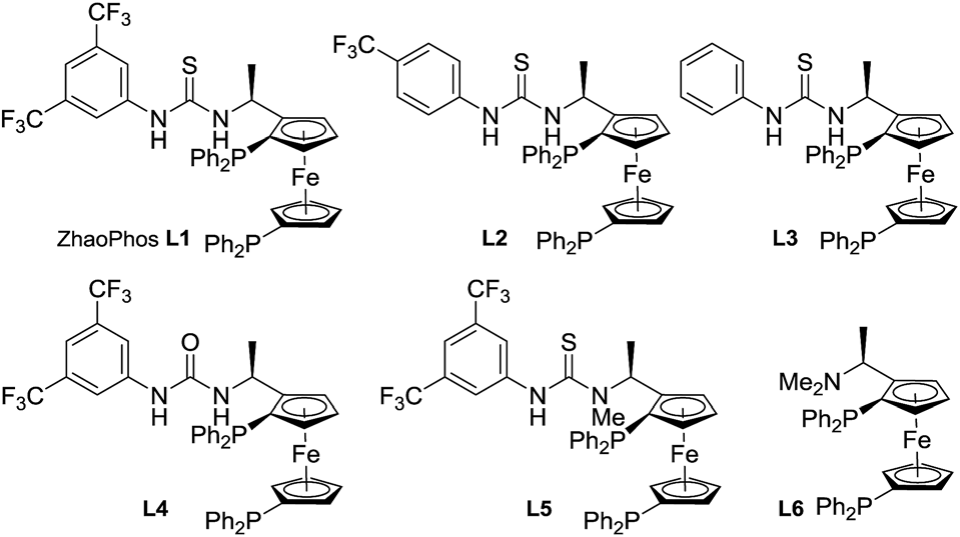
The structure of bisphosphine ligands.

A series of bisphosphine–(thio)urea ligands (Fig. 2) were then applied to this Ir-catalyzed asymmetric hydrogenation of 3-phenyl-2*H*-benzo[*b*][1,4]oxazin-2-one **1a** in THF. Full conversions and excellent enantioselectivities can be obtained in the presence of ZhaoPhos **L1** and *N*-methylated ZhaoPhos **L5**, and *N*-methylated ZhaoPhos **L5** provided higher enantioselectivity (>99% conversion, 98–99% ee, Table 2, entries 1 and 5). The ligand **L2** containing one trifluoromethyl group and ligand **L3** without any trifluoromethyl group on the phenyl ring provided poor conversions and excellent enantioselectivities (33–45% conversions, 97% ee, Table 2, entries 2 and 3). The ligand **L4** displayed very poor reactivity and enantioselectivity, which changed the thiourea motif to the urea motif (11% conversion, 13% ee, Table 2, entry 4). In addition, no reaction was observed in the presence of ligand **L6** without the thiourea motif (Table 2, entry 6). This indicated that the thiourea motif may make a great contribution to activate our substrate through anion-binding interactions. When the catalyst loading is decreased from 1.0 mol% to 0.2 mol%, full conversion and excellent enantioselectivity can be obtained with ligand **L5** (>99% conversion, 99% ee, Table 2, entry 8). Interestingly, it is better than ZhaoPhos **L1** (65% conversion, 98% ee, Table 2, entry 7 *vs.* entry 8). It is possible that a single anion-binding interaction in a precise position is sufficient in this asymmetric reaction, which is different from previous reports.^11^,12b,d,e,g

**Table 2:** Screening a series of bisphosphine–(thio)urea ligands for asymmetric hydrogenation of 3-phenyl-2*H*-benzo[*b*][1,4]oxazin-2-one (**1a**)^*a*^

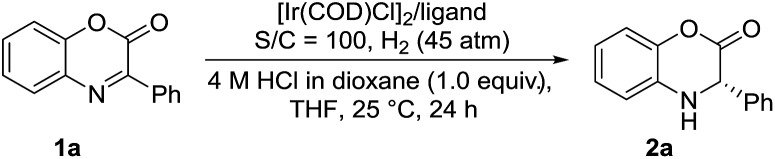			
Entry	Ligand	Conv.^*b*^ (%)	ee^*c*^ (%)
1	ZhaoPhos **L1**	>99	98
2	**L2**	45	97
3	**L3**	33	97
4	**L4**	11	13
5	**L5**	>99	99
6	**L6**	NR	NA
7^*d*^	ZhaoPhos **L1**	65	98
8^*d*^	**L5**	>99	99

^*a*^Reaction conditions: 0.05 mmol **1a** in 1.0 mL THF, S/C = 100, 45 atm H_2_, 1.0 equiv. HCl (4 M in dioxane), 25 °C, 24 h.

^*b*^Determined by ^1^H NMR analysis.

^*c*^Determined by HPLC with a chiral stationary phase.

^*d*^30 atm H_2_, S/C = 500, 16 h.

A series of representative Brønsted acids were then deeply inspected in this Ir/ligand **L5**-catalyzed asymmetric hydrogenation of 3-phenyl-2*H*-benzo[*b*][1,4]oxazin-2-one **1a**, and we found that there is a significant correlation between the reactivity, enantioselectivity and acid strength of the Brønsted acid. When hydrochloric acid, HCl (4 M in dioxane), was switched to a strong acid, CF_3_SO_3_H, this hydrogenation proceeded smoothly to provide the same result with full conversion and 99% ee (Table 3, entries 1 and 7). CF_3_COOH and H_3_PO_4_ gave high conversions and good enantioselectivities (95–>99% conversions, 81–84% ee, Table 3, entries 2 and 3). As expected, the weaker acids HCOOH and AcOH afforded poor reactivities and enantioselectivities (21–30% conversions, 57–58% ee, Table 3, entries 4–5). In addition, the amount of Brønsted acid HCl was further investigated in this asymmetric hydrogenation. We found that the amount of HCl had little effect on the reactivity and enantioselectivity, and the reaction results remained excellent, when the amount of HCl was gradually reduced from 2.0 equiv. to 0.01 equiv. (>99% conversion, 95–99% ee, Table 3, entries 6–10). However, no reaction was observed in the absence of the cocatalyst HCl (Table 3, entry 11), which showed that HCl was involved in this transformation with great importance. These results also displayed that the acid strength of the Brønsted acid cocatalyst is very important to achieve excellent results in this asymmetric hydrogenation, which may affect the formation of anion-binding activation among the substrate, Brønsted acid and ligand. To our delight, this asymmetric hydrogenation still proceeded smoothly with the same result even when catalyzed by only the 0.1 mol% [Ir(COD)Cl]_2_/ligand **L5** catalyst with 1.0 equiv. HCl (4 M in dioxane) (Table 3, entry 12).

**Table 3:** Investigation of the effect of the Brønsted acid cocatalyst^*a*^

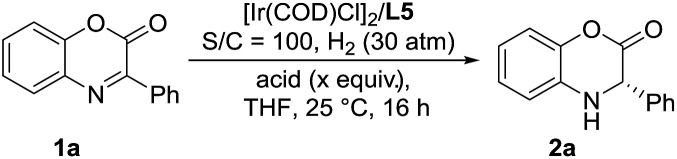					
Entry	Acid	p*K*_a_ (in H_2_O)^*b*^	*x* (equiv.)	Conv.^*c*^ (%)	ee^*d*^ (%)
1	CF_3_SO_3_H	–14	1.0	>99	99
2	CF_3_COOH	–0.25	1.0	>99	84
3	H_3_PO_4_	2.12	1.0	95	81
4	HCOOH	3.77	1.0	30	58
5	CH_3_COOH	4.76	1.0	21	57
6	HCl	–8	2.0	>99	99
7	HCl	–8	1.0	>99	99
8	HCl	–8	0.5	>99	99
9	HCl	–8	0.1	>99	99
10	HCl	–8	0.01	>99	95
11	HCl	–8	0	NR	NA
12^*e*^	HCl	–8	1.0	>99	99

^*a*^Reaction conditions: 0.05 mmol substrate **1a** in 1.0 mL THF, 30 atm H_2_, 25 °C; S/C = 100, 16 h.

^*b*^The p*K*_a_ data are available from Evans's p*K*_a_ table.

^*c*^Conversion was determined by ^1^H NMR analysis.

^*d*^ee was determined by HPLC with a chiral stationary phase.

^*e*^S/C = 1000. NR is no reaction, NA is not available.

We then continued to investigate the substrate generality of this Ir-catalyzed asymmetric hydrogenation of prochiral benzoxazinones using *N*-methylated ZhaoPhos **L5** as the ligand. These reaction results are summarized in Table 4. A series of prochiral benzoxazinones were hydrogenated smoothly in this catalytic system to prepare various chiral dihydrobenzoxazinones with excellent results (>99% conversion, 88–96% yields, 91–>99% ee). The benzoxazinone substrates with different substituents on the benzo ring (**1b–1e**) were hydrogenated well with >99% conversion, 93–95% yields and >99% ee. In addition, regardless of the position or electronic properties of the substituents on the phenyl ring of the benzoxazinone substrates (**1f–1j**), the corresponding hydrogenation products (**2f–2j**) were obtained with 88–95% yields and >99% ee. The naphthyl substituted substrates with bulky steric hindrance (**1k–1l**) were obtained smoothly with excellent results (91–95% yields, 97–>99% ee). It is worth noting that the heteroaromatic substrate 3-(thiophen-3-yl)-2*H*-benzo[*b*][1,4]oxazin-2-one (**1m**) was well tolerated to produce the desired product (**2m**) with 96% yield and >99% ee. Moreover, the alkyl substrates (**1n–1p**) were hydrogenated smoothly with excellent results (>99% conversion, 91–95% yields, and 91–98% ee).

**Table 4:** Substrate scope study of Ir-catalyzed asymmetric hydrogenation of benzoxazinones^*a*^

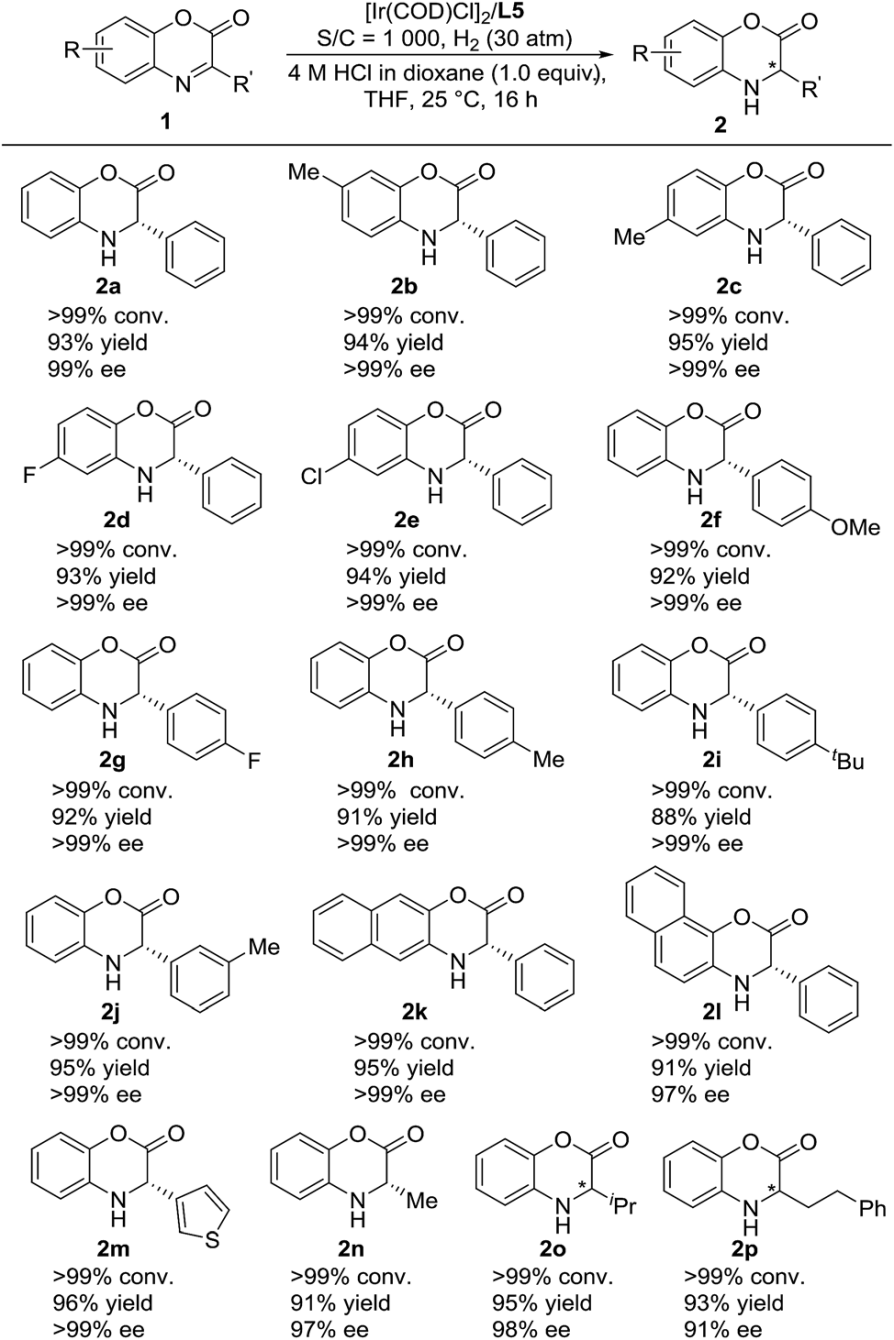

^*a*^Unless otherwise noted, all reactions were carried out with a [Ir(COD)Cl]_2_/ligand **L5**/substrate **1** (0.05 mmol) ratio of 0.5 : 1.1 : 1000 in 1.0 mL THF with 1.0 equiv. HCl (4 M in dioxane) at room temperature under 30 atm H_2_ for 16 h. Conversion was determined by ^1^H NMR analysis, ee was determined by HPLC with a chiral stationary phase, and the yield was isolated yield.

Encouraged by the success of the Ir/*N*-methylated ZhaoPhos **L5**-catalyzed asymmetric hydrogenation of prochiral benzoxazinones, the asymmetric hydrogenation of several quinoxalinones was subsequently explored. As shown in Table 5, the asymmetric hydrogenation of quinoxalinones (**3a–3c**) proceeded efficiently, affording the desired products (**4a–4c**) with >99% conversion, 90–91% yields and 98–>99% ee (Table 5, entries 1–3). To our delight, the hydrogenation of benzo-seven-membered cyclic imine 4-phenyl-1*H*-benzo[*b*][1,4]diazepin-2(3*H*)-one (**3d**) proceeded smoothly to obtain the hydrogenation product (**4d**) with excellent results (>99% conversion, 91% yield, >99% ee, Table 5, entry 4). In addition, our catalytic system displayed extremely high activity in this asymmetric hydrogenation, and when the catalyst loading was reduced from 0.1 mol% to 0.02 mol% (S/C = 5000), the asymmetric hydrogenation of benzo-seven-membered cyclic imine (**3d**) can be finished with >99% conversion, 93% yield and >99% ee (Table 5, entry 5).

**Table 5:** Substrate scope study for Ir-catalyzed asymmetric hydrogenation of quinoxalinones and 4-phenyl-1*H*-benzo[*b*][1,4]diazepin-2(3*H*)-one^*a*^

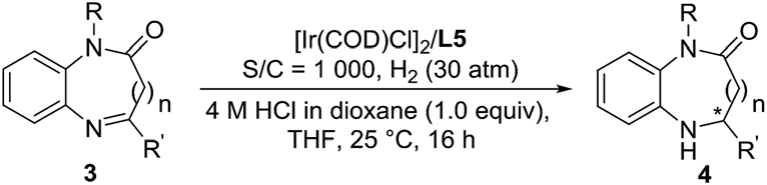								
Entry	R	R′	*n*	Sub.	Prod.	Conv.^*b*^ (%)	Yield^*c*^ (%)	ee^*d*^ (%)
1	H	Ph	0	**3a**	**4a**	>99	90	>99
2	H	Et	0	**3b**	**4b**	>99	91	99
3	Me	Ph	0	**3c**	**4c**	>99	91	98
4	H	Ph	1	**3d**	**4d**	>99	91	>99
5^*e*^	H	Ph	1	**3d**	**4d**	>99	93	>99

^*a*^Unless otherwise noted, all reactions were carried out with a [Ir(COD)Cl]_2_/ligand **L5**/substrate **3** (0.05 mmol) ratio of 0.5 : 1.1 : 1000 in 1.0 mL THF at room temperature under 30 atm H_2_ for 16 h.

^*b*^Determined by ^1^H NMR analysis.

^*c*^Isolated yield.

^*d*^Determined by HPLC analysis using a chiral stationary phase.

^*e*^S/C = 5000.

In order to further investigate the high activity of this Ir/*N*-methylated ZhaoPhos **L5** catalytic system, the model substrate 3-phenyl-2*H*-benzo[*b*][1,4]oxazin-2-one **1a** was applied in this asymmetric hydrogenation with very low catalyst loading under 40 atm H_2_. These results are summarized in Table 6. The hydrogenation product **2a** can be obtained with full conversion, 91% yield and 99% ee when the catalyst loading is decreased from 0.1 mol% (S/C = 1000) to 0.01 mol% (S/C = 10 000) (Table 6, entries 1 and 2). Our catalytic system still exhibited high activity and excellent enantioselectivity with further diminution of the catalyst loading to 0.0025 mol% (S/C = 40 000), affording the product **2a** with excellent results (>99% conversion, 89% yield and 99% ee, Table 6, entry 3). In addition, good conversion and excellent enantioselectivity can be obtained even in the presence of 0.002 mol% (S/C = 50 000) catalyst (81% conversion, TON = 40 500, 72% yield, 99% ee, Table 6, entry 4).

**Table 6:** High TON experiment^*a*^

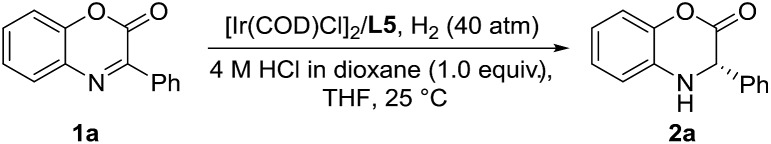					
Entry	S/C	Time (h)	Conv.^*b*^ (%)	Yield^*c*^ (%)	ee^*d*^ (%)
1	1000	16	>99	93	99
2	10 000	48	>99	91	99
3	40 000	72	>99	89	99
4	50 000	72	81	72	99

^*a*^Reaction conditions: substrate **1a** (5.0 mmol) in 20.0 mL THF, 40 atm H_2_, 1.0 equiv. HCl (4 M in dioxane), 25 °C.

^*b*^Conversion was determined by ^1^H NMR analysis.

^*c*^Isolated yield.

^*d*^ee was determined by HPLC with a chiral stationary phase.

**Scheme 2:**
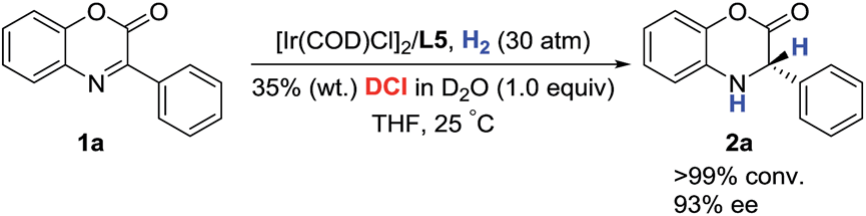
Deuterium-labeling experiment.

The deuterium-labeling experiment was conducted to verify the hydrogen atom source of the hydrogenation product dihydrobenzoxazinone. As shown in Scheme 2, the asymmetric hydrogenation of 3-phenyl-2*H*-benzo[*b*][1,4]oxazin-2-one **1a** proceeded in the presence of DCl in D_2_O, and the product **2a** without deuterium was obtained. This observation displayed that the hydrogen atom of the hydrogenation product was from H_2_ and not HCl to a great extent.

**Fig. 3:**
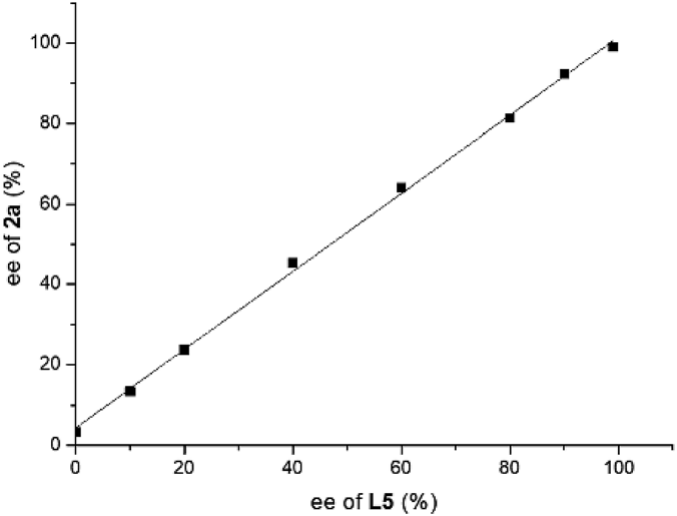
Investigation of the nonlinear effect of the hydrogenation of substrate **1a** using ligand **L5** with different ee values.

A series of asymmetric reductions of model substrate 3-phenyl-2*H*-benzo[*b*][1,4]oxazin-2-one **1a** were performed using ligand **L5** with varying ee values. As shown in Fig. 3, there is no nonlinear effect in this transformation, which indicated that there should be no catalyst self-aggregation or ligand–substrate agglomeration in this catalytic system.^13^

**Scheme 3:**
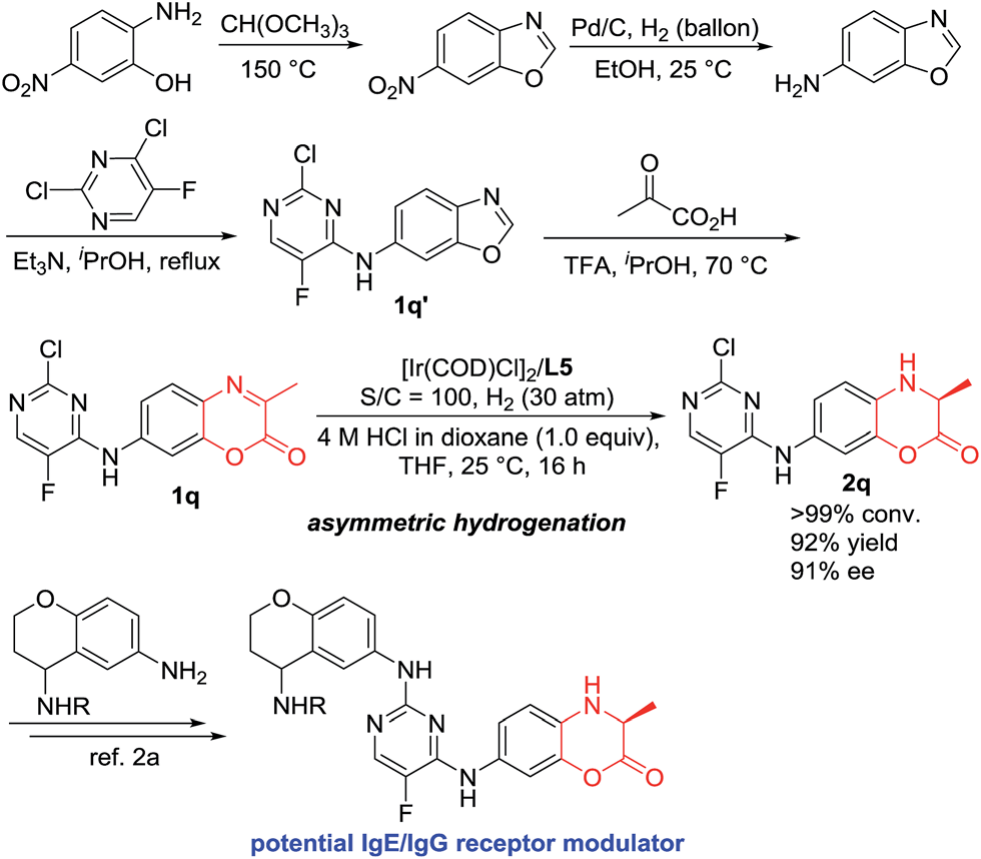
Synthetic application for the construction of the key intermediate of biologically active molecules.

IgE/IgG is one of the most important immunoglobulins, which are associated with the release of vasoactive amines stored in basophils and tissue mast cell granules to cause allergic effects. Our catalytic hydrogenation methodology showed great synthetic application. As shown in Scheme 3, 7-((2-chloro-5-fluoropyrimidin-4-yl)amino)-3-methyl-2*H*-benzo[*b*][1,4]oxazin-2-one **1q** was efficiently obtained within four steps using the easily commercially available 2-amino-5-nitrophenol as the starting material.^14^ The Ir-catalyzed asymmetric hydrogenation of compound **1q** was efficiently accomplished to obtain the chiral compound **2q** with 92% yield and 91% ee, which is the key intermediate to construct the potential IgE/IgG receptor modulator for the treatment of autoimmune diseases.^2^

## Conclusions

In summary, the Ir-catalyzed asymmetric hydrogenation of benzoxazinones and derivatives was successfully developed with *N*-methylated ZhaoPhos **L5** as the ligand through a new anion-binding activation strategy. A series of chiral dihydrobenzoxazinones and derivatives were obtained with excellent results (88–96% yields, 91–>99% ee, up to 40 500 TON). The utilization of a strong Brønsted acid, such as hydrochloric acid even at a small catalytic amount, as the cocatalyst is very important to form a single anion-binding interaction with the substrate and ligand **L5**, which strongly improved the reactivity. Moreover, a highly efficient synthetic route was developed to construct the key intermediate to synthesize a potential IgE/IgG receptor modulator through our catalytic methodology system.

## Conflicts of interest

There are no conflicts to declare.

## Supplementary Material

Supplementary information
