# Supplementary material for: Highly efficient Ir-catalyzed asymmetric hydrogenation of benzoxazinones and derivatives with a Brønsted acid cocatalyst
Source: Chem Sci. 2019 Mar 19;10(15):4328–33. doi: 10.1039/c8sc05797d (PMC6471538; doi:10.1039/c8sc05797d)
Supplement: Supplementary file 1 [file SC-010-C8SC05797D-s001.pdf]

Electronic Supplementary Material (ESI) for Chemical Science.
This journal is © The Royal Society of Chemistry 2019

Supporting information

Highly Efficient Ir-Catalyzed Asymmetric Hydrogenation of Benzoxazinones and Derivatives with a Brønsted Acid Cocatalyst

Zhengyu Han,† Gang Liu,† Rui Wang,† Xiu-Qin Dong,*† and Xumu Zhang*§,†

† Key Laboratory of Biomedical Polymers, Engineering Research Center of Organosilicon Compounds & Materials, Ministry of Education, College of Chemistry and Molecular Sciences, Wuhan University, Wuhan, Hubei, 430072, P. R. China.

§ Department of Chemistry and Shenzhen Grubbs Institute, Southern University of Science and Technology, Shenzhen, Guangdong, 518055, P. R. China.

E-mail: zhangxm@sustc.edu.cn, xiuqindong@whu.edu.cn.

Content

I. General Remarks……………………………………………...........………….……….….…2

II. General Procedure for the Synthesis of Substrates…………………………………….…….……2

III. General Procedure for Asymmetric Hydrogenation…………………………..……..…..….11

IV. Linear Effect of the Hydrogenation of Substrate **1a**………………………………...…...…..21

V. Reference………………………………………………………….……………….…..…..22

VI. NMR Spectra……………………………………………..………………….…..…..23

VII. HPLC Spectra…………………………………………………………………….…..65

I. General Remarks

All the reactions with air- or moisture-sensitive compounds were carried out in a dry reaction vessel under a positive pressure of nitrogen or in the argon-filled glovebox. Unless otherwise noted, all reagents and solvents were purchased from commercial suppliers without further purification. Anhydrous solvents were purchased from J&K Chemical Technology company and transferred by syringe. ^{1}H NMR and ^{13}C NMR spectra were recorded on a Bruker ADVANCE III (400 MHz) spectrometer with $CDCl_3$ as the solvent and tetramethylsilane (TMS) as the internal standard. Chemical shifts are reported in parts per million (ppm, δ scale) downfield from TMS at 0.00 ppm and referenced to the $CDCl_3$ at 7.26 ppm (for ^{1}H NMR) or 77.0 ppm (for ^{13}C NMR). Data are reported as: multiplicity (s = singlet, d = doublet, t = triplet, q = quartet, m = multiplet), coupling constant in hertz (Hz) and signal area integration in natural numbers. ^{13}C NMR and ^{31}P NMR analyses were run with decoupling. Enantiomeric excess values were determined by Daicel chiral column on an Agilent 1260 Series HPLC instrument. Optical rotations $[\alpha]_D$ were measured on a PERKIN ELMER polarimeter 343 instrument.

All the benzoxazinone or quinoxalinones were prepared according the literature.[1-7] The absolute configuration of products **2a**, **2c**, **2e-2h**, **2j**, **2m**, **4a** and **4c** were determined by comparison of analytical data with the literature (HPLC spectra, optical rotation).[3, 5, 8-9] The absolute configuration of others were assigned by analogy.

II. General Procedure for the Synthesis of Substrates

A) Preparation of substrates 1a-1p [1]

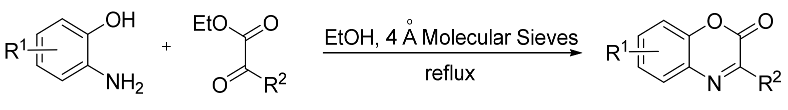

To the solution of appropriate 2-aminophenol derivative (10.0 mmol, 1.0 eq.) and the corresponding α-ketoester (11.0 mmol, 1.1 eq.) in dry ethanol (20 mL) 4Å molecular sieves (2.0 g) was added and the resulting mixture was refluxed for 12 h. Then the solution was cooled to room temperature and passed through a pad of celite, and concentrated in vacuo. The crude product was purified by column chromatography using petroleum ether/EtOAc (20:1) mixtures as the eluent. If

required, the isolated product after chromatography was further recrystallized from EtOH to obtain the corresponding benzoxazinones.

B) Preparation of substrates 3a-3d [1]

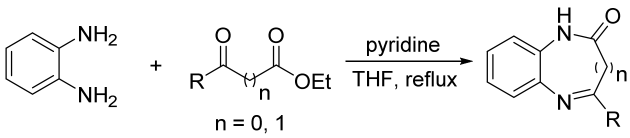

To the solution of 1,2-diaminobenzene (10.0 mmol, 1.0 eq.) in THF (20 mL) the appropriate α-ketoester or ethyl benzoylacetate (10 mmol, 1.0 eq.) was added followed with pyridine (2 mL), the resulting mixture was refluxed for 12 h. Then the solution was cooled to room temperature and concentrated in vacuo. The crude product was purified by column chromatography using petroleum ether/EtOAc (20:1) mixtures as the eluent. If required, the isolated product after chromatography was further recrystallized from EtOH to obtain the corresponding quinoxalinones or 4-phenyl-1H-benzo[*b*][1,4]diazepin-2(3H)-one.

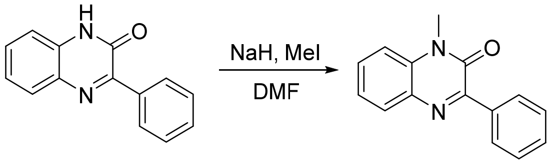

Dissolve the 3-phenylquinoxaline-2(1H)-one (2.0 mmol, 1.0 eq.) in DMF (15 mL) and NaH (6.0 mmol, 3.0 eq.) was added slowly at 0 °C, methyl iodide (6.0 mmol) was then added. The resulting mixture was stirred at 0 °C for another 2 h, and then quenched with water and extracted with EtOAc. The organic layer was washed with saturated $NaHCO_3$ and brine and then dried over Na_2SO_4. The solvent was removed in vacuo to give a solid that was purified by column chromatography using petroleum ether/EtOAc (20:1) mixtures as the eluent to give the corresponding N-methylated 3-substituted quinoxalin-2(1H)-one.

C) Preparation of substrate 1q [2]

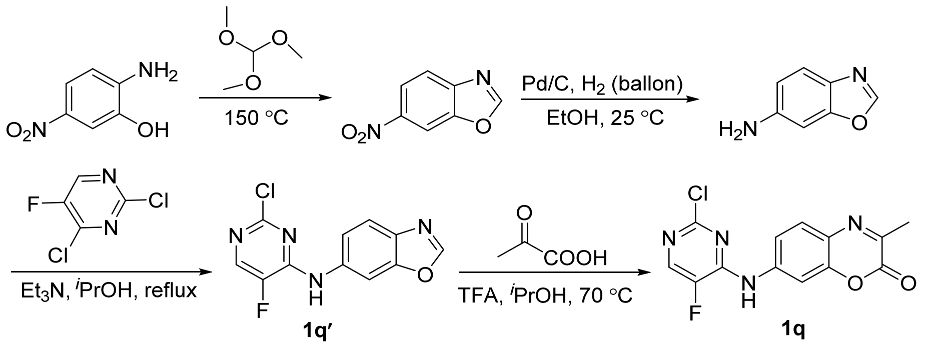

2-amino-5-nitro-phenol (50.0 mmol) was dissolved in trimethylorthoformate (25 mL) and stirred at 150 °C for 12 h. Then the solution was cooled to room temperature and concentrated in vacuo. The crude product was purified by column chromatography using petroleum ether/EtOAc (10:1) mixtures as the eluent to get the desired 6-nitrobenzo[*d*]oxazole.

A solution of 6-nitrobenzo[*d*]oxazole (50.0 mmol) in EtOH (25 mL) was added Pd/C (1.6 g) and stirred under H_2 atmosphere (ballon) at 25 °C for 12 h, then the reaction mixture passed through a column of silica gel to remove the Pd/C and the solution was concentrated under vacuo to get the benzo[*d*]oxazol-6-amine.

2,4-dichloro-5-fluoro-pyrimidine (55.0 mmol) and benzo[*d*]oxazol-6-amine (50.0 mmol) was dissolved in ^{i}PrOH (50 mL) and Et_3N (75 mmol), the resulting mixture was allowed heated to reflux for 12 h. Then the reaction mixture was concentrated under vacuo and purified by column chromatography to get the desired product **1q'**.

1q' (4 mmol) and pyruvic acid (6 mmol) was dissolved in ^{i}PrOH (5 mL) and TFA (0.8 mmol) was added slowly. The resulting mixture was heated to 70 °C and stirred for 18 h. Then the solution was cooled to room temperature and washed with $NaHCO_3$ (aqueous) then extracted with EA (2×20 mL). The organic layer was concentrated under vacuo and purified by column chromatography to get the desired product **1q**.

3-phenyl-2*H*-benzo[*b*][1,4]oxazin-2-one (1a)

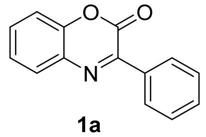

1a

Light yellow solid, 77% yield; ^{1}H NMR (400 MHz, $CDCl_3$): δ (ppm) 8.34-8.32 (m, 2H), 7.87-7.85 (m, 1H), 7.57-7.48 (m, 4H), 7.42-7.38 (m, 1H), 7.35-7.33 (m, 1H); ^{13}C NMR (100 MHz, $CDCl_3$): δ (ppm) 152.3, 150.9, 146.4, 134.1, 131.6, 131.4, 131.1, 129.44, 129.41, 128.4, 125.6, 116.2. The characterization data of compound **1a** is in accordance with the reported data in the literature.[2a]

7-methyl-3-phenyl-2*H*-benzo[*b*][1,4]oxazin-2-one (1b)

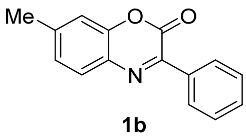

1b

Yellow solid, mp = 147-149 °C, 82% yield; ^{1}H NMR (400 MHz, $CDCl_3$): δ (ppm) 8.32-8.30 (m, 2H), 7.73 (d, *J* = 8.0 Hz, 1H), 7.53-7.47 (m, 3H), 7.22-7.20 (m, 1H) , 7.14 (s, 1H), 2.49 (s, 3H); ^{13}C NMR (100 MHz, $CDCl_3$): δ (ppm) 152.5, 149.6, 146.3, 142.5, 134.3, 131.1, 129.7, 129.3, 129.0, 128.3, 126.7, 116.2, 21.8. The characterization data of compound **1b** is in accordance with the reported data in the literature.[1, 2a]

6-methyl-3-phenyl-2*H*-benzo[*b*][1,4]oxazin-2-one (1c)

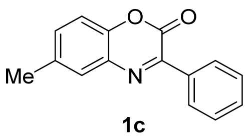

1c

Yellow solid; ^{1}H NMR (400 MHz, $CDCl_3$): δ (ppm) 8.32-8.30 (m, 2H), 7.62 (s, 1H), 7.54-7.46 (m, 3H), 7.31-7.28 (m, 1H), 7.20-7.18 (m, 1H), 2.43 (s, 3H); ^{13}C NMR (100 MHz, $CDCl_3$): δ (ppm) 152.4, 150.6, 144.3, 135.4, 134.2, 132.1, 131.28, 131.25, 129.3, 129.2, 128.3, 115.7, 20.8. The characterization data of compound **1c** is in accordance with the reported data in the literature.[2a]

6-fluoro-3-phenyl-2*H*-benzo[*b*][1,4]oxazin-2-one (1d)

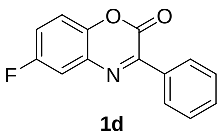

1d

Yellow solid, mp = 143-145 °C, 89% yield; ^{1}H NMR (400 MHz, $CDCl_3$): δ (ppm) 8.36-8.33 (m, 2H), 7.58-7.49 (m, 4H), 7.34-7.30 (m, 1H), 7.28-7.23 (m, 1H); ^{13}C NMR (100 MHz, $CDCl_3$): δ

(ppm) 159.4 (d, J = 244.0 Hz), 151.9 (d, J = 11.0 Hz), 142.8 (d, J = 3.0 Hz), 133.7, 132.0 (d, J = 12.0 Hz), 131.8, 129.6, 128.5, 118.5 (d, J = 25.0 Hz), 117.2 (d, J = 9.0 Hz), 115.0 (d, J = 24.0 Hz). HRMS (ESI): [M+H$^+$] Calc. 242.0617, found 242.0611.

6-chloro-3-phenyl-2*H*-benzo[*b*][1,4]oxazin-2-one (1e)

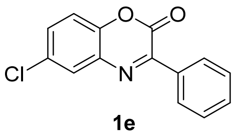

1e

Light yellow solid, mp = 149-151 °C, 86% yield; ^{1}H NMR (400 MHz, $CDCl_3$): δ (ppm) 8.34-8.32 (m, 2H), 7.85 (s, 1H), 7.58-7.46 (m, 4H), 7.29-7.27 (m, 1H); ^{13}C NMR (100 MHz, $CDCl_3$): δ (ppm) 151.74, 151.68, 145.0, 133.7, 132.1, 131.9, 131.0, 130.7, 129.6, 128.8, 128.5, 117.3. The characterization data of compound **1e** is in accordance with the reported data in the literature. [2a]

3-(4-methoxyphenyl)-2*H*-benzo[*b*][1,4]oxazin-2-one (1f)

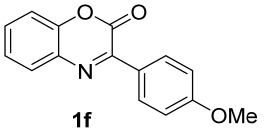

1f

Yellow solid, mp = 146-149 °C, 66% yield; ^{1}H NMR (400 MHz, $CDCl_3$): δ (ppm) 8.41-8.39 (m, 2H), 7.84-7.81 (m, 1H), 7.49-7.47 (m, 1H), 7.38-7.36 (m, 1H), 7.34-7.31 (m, 1H), 7.02-7.00 (m, 2H), 3.90 (s, 3H); ^{13}C NMR (100 MHz, $CDCl_3$): δ (ppm) 162.3, 152.5, 149.8, 146.2, 131.7, 131.4, 130.4, 129.1, 126.8, 125.5, 116.0, 113.8, 55.4. The characterization data of compound **1f** is in accordance with the reported data in the literature.[2a]

3-(4-fluorophenyl)-2*H*-benzo[*b*][1,4]oxazin-2-one (1g)

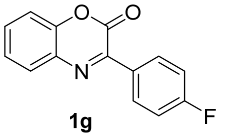

1g

Light brown solid, mp = 166-169 °C, 79% yield; ^{1}H NMR (400 MHz, $CDCl_3$): δ (ppm) 8.43-8.40 (m, 2H), 7.86-7.83 (m, 1H), 7.55-7.51 (m, 1H), 7.43-7.39 (m, 1H), 7.36-7.33 (m, 1H), 7.21-7.16 (m, 2H); ^{13}C NMR (100 MHz, $CDCl_3$): δ (ppm) 164.8 (d, J = 252.0 Hz), 152.3, 149.5, 146.4,

131.9, 131.8, 131.5, 131.2, 129.4, 125.7, 116.2, 115.5 (d, J = 22.0 Hz). The characterization data of compound **1g** is in accordance with the reported data in the literature.[2a]

3-(*p*-tolyl)-2*H*-benzo[*b*][1,4]oxazin-2-one (1h)

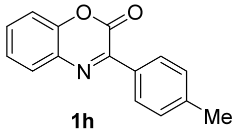

1h

Yellow solid, 70% yield; ^{1}H NMR (400 MHz, $CDCl_3$): δ (ppm) 8.26 (d, J = 8.0 Hz, 2H), 7.85-7.83 (m, 1H), 7.52-7.48 (m, 1H), 7.41-7.37 (m, 1H), 7.34-7.30 (m, 3H), 2.44 (s, 3H); ^{13}C NMR (100 MHz, $CDCl_3$): δ (ppm) 152.4, 150.7, 146.4, 142.0, 131.7, 131.4, 130.8, 129.4, 129.3, 129.1, 125.5, 116.1, 21.6. The characterization data of compound **1h** is in accordance with the reported data in the literature.[1, 2a]

3-(4-(tert-butyl)phenyl)-2*H*-benzo[*b*][1,4]oxazin-2-one (1i)

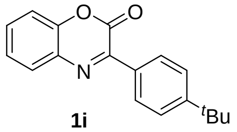

1i

Light yellow solid, mp = 141-144 °C, 73% yield; ^{1}H NMR (400 MHz, $CDCl_3$): δ (ppm) 8.28-8.26 (m, 2H), 7.86-7.83 (m, 1H), 7.54-7.48 (m, 3H), 7.41-7.32 (m, 2H), 1.37 (s, 9H); ^{13}C NMR (100 MHz, $CDCl_3$): δ (ppm) 155.0, 152.4, 150.8, 146.4, 131.7, 131.4, 130.8, 129.3, 129.2, 125.5, 125.4, 116.1, 35.0, 31.1.

3-(*m*-tolyl)-2*H*-benzo[*b*][1,4]oxazin-2-one (1j)

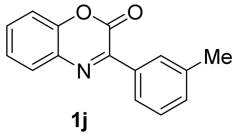

1j

Yellow solid, mp = 93-96 °C, 53% yield; ^{1}H NMR (400 MHz, $CDCl_3$): δ (ppm) 8.12 (d, J = 8.0 Hz, 2H), 7.87-7.85 (m, 1H), 7.54-7.49 (m, 1H), 7.41-7.32 (m, 4H), 2.46 (s, 3H); ^{13}C NMR (100 MHz, $CDCl_3$): δ (ppm) 152.3, 151.1, 146.4, 138.1, 134.0, 132.3, 131.6, 131.0, 129.8, 129.4, 128.3, 126.7, 125.5, 116.1, 21.5. HRMS (ESI): [M+H$^+$] Calc. 238.0868, found 238.0860.

3-phenyl-2*H*-naphtho[2,3-*b*][1,4]oxazin-2-one (1k)

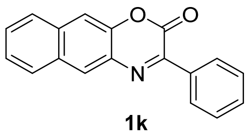

1k

Deep yellow solid, 69% yield; known compound;[4] ^{1}H NMR (400 MHz, $CDCl_3$): δ (ppm) 8.38-8.35 (m, 3H), 8.00 (d, *J* = 8.0 Hz, 1H), 7.90 (d, *J* = 8.0 Hz, 1H), 7.70 (s, 1H), 7.62-7.50 (m, 5H); ^{13}C NMR (100 MHz, $CDCl_3$): δ (ppm) 152.2, 151.1, 144.2, 134.2, 133.9, 131.5, 130.9, 130.8, 129.5, 129.2, 128.9, 128.5, 128.4, 127.4, 126.0, 112.3. The characterization data of compound **1k** is in accordance with the reported data in the literature.[4]

3-phenyl-2*H*-naphtho[1,2-b][1,4]oxazin-2-one (1l)

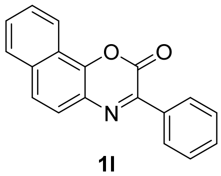

1l

Deep yellow solid, mp = 157-160 °C, 73% yield; ^{1}H NMR (400 MHz, $CDCl_3$): δ (ppm) 8.50-8.48 (m, 1H), 8.43-8.41 (m, 2H), 7.92-7.89 (m, 1H), 7.85-7.77 (m, 2H), 7.68-7.63 (m, 2H), 7.56-7.50 (m, 3H); ^{13}C NMR (100 MHz, $CDCl_3$): δ (ppm) 152.4, 150.0, 142.6, 134.3, 134.2, 131.3, 129.3, 128.7, 128.4, 128.0, 127.9, 127.3, 125.6, 125.5, 122.5, 122.1.

3-(thiophen-3-yl)-2*H*-benzo[*b*][1,4]oxazin-2-one (1m)

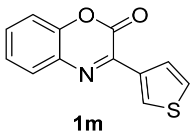

1m

Wheat solid, mp = 123-126 °C, 43% yield; ^{1}H NMR (400 MHz, $CDCl_3$): δ (ppm) 8.76 (brs, 1H), 8.00-7.98 (m, 1H), 7.79-7.77 (m, 1H), 7.49-7.45 (m, 1H), 7.38-7.34 (m, 2H), 7.31-7.26 (m, 1H); ^{13}C NMR (100 MHz, $CDCl_3$): δ (ppm) 152.1, 145.90, 145.87, 136.1, 132.2, 131.5, 130.6, 129.1, 127.8, 125.52, 125.48, 116.1. HRMS (ESI): [M+H^{+}] Calc. 230.0276, found 230.0269.

3-methyl-2*H*-benzo[*b*][1,4]oxazin-2-one (1n)

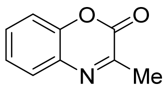

1n

Brown solid; ^{1}H NMR (400 MHz, $CDCl_3$): δ (ppm) 7.72-7.70 (m, 1H), 7.50-7.46 (m, 1H), 7.38-7.34 (m, 1H), 7.30-7.28 (m, 1H), 2.58 (s, 3H); ^{13}C NMR (100 MHz, $CDCl_3$): δ (ppm) 155.1, 153.2, 146.5, 131.0, 130.5, 128.6, 125.4, 116.4, 21.3. The characterization data of compound **1n** is in accordance with the reported data in the literature.[2a]

3-isopropyl-2*H*-benzo[*b*][1,4]oxazin-2-one (1o)

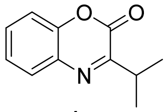

1o

White solid; mp = 37-39 °C, 82% yield; known compound;[3] ^{1}H NMR (400 MHz, $CDCl_3$) δ 7.75 (d, *J* = 7.9 Hz, 1H), 7.46 (t, *J* = 7.8 Hz, 1H), 7.34 (t, *J* = 7.6 Hz, 1H), 7.28- 7.26 (m, 1H), 3.46 (hept, *J* = 6.7 Hz, 1H), 1.33 (d, *J* = 6.8 Hz, 6H). ^{13}C NMR (100 MHz, $CDCl_3$) δ 161.9, 152.5, 146.2, 131.2, 130.3, 128.9, 125.2, 116.2, 31.9, 19.8.

3-phenethyl-2*H*-benzo[*b*][1,4]oxazin-2-one (1p)

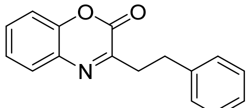

1p

Yellow solid; mp = 108-111 °C, 71% yield; known compound;[1] ^{1}H NMR (400 MHz, $CDCl_3$) δ 7.76 (dd, *J* = 7.9, 1.6 Hz, 1H), 7.50-7.46 (m, 1H), 7.39-7.35 (m, 1H), 7.31-7.28 (m, 5H), 7.23-7.20 (m, 1H), 3.25-3.20 (m, 2H), 3.16-3.11 (m, 2H). ^{13}C NMR (100 MHz, $CDCl_3$) δ 157.0, 152.9, 146.4, 140.7, 131.1, 130.6, 128.8, 128.51, 128.47, 126.2, 125.4, 116.4, 35.8, 32.1.

7-((2-chloro-5-fluoropyrimidin-4-yl)amino)-3-methyl-2*H*-benzo[*b*][1,4]oxazin-2-one (1q)

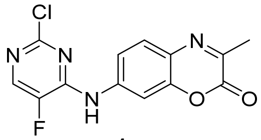

1q

Yellow solid, mp >300 °C, 35% total yield; ^{1}H NMR (400 MHz, C_2D_6SO): δ (ppm) 10.35 (s, 1H), 8.43 (d, *J* = 4.0 Hz, 1H), 7.88 (d, *J* = 4.0 Hz, 1H), 7.72-7.65 (m, 2H), 2.38 (s, 3H); ^{13}C NMR (100 MHz, C_2D_6SO): δ (ppm) 153.7 (d, *J* = 48.0 Hz), 153.1 (d, *J* = 4.0 Hz), 151.0 (d, *J* = 12.0 Hz), 147.1, 147.0, 144.5, 142.8 (d, *J* = 21.0 Hz), 140.1, 128.6, 127.6, 117.9, 107.7, 21.4. HRMS (ESI): [M+H$^+$] Calc. 307.0398, found 307.0391.

3-phenylquinoxalin-2(1*H*)-one (3a)

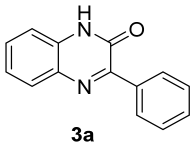

3a

Light yellow solid, 84% yield; known compound;[5] ^{1}H NMR (400 MHz, C_2D_6SO): δ (ppm) 12.60 (s, 1H), 8.32-8.29 (m, 2H), 7.84 (t, *J* = 4.0 Hz, 1H), 7.57-7.48 (m, 4H), 7.35-7.31 (m, 2H); ^{13}C NMR (100 MHz, C_2D_6SO): δ (ppm) 155.1, 154.6, 136.1, 132.52, 132.48, 130.8, 130.7, 129.7, 129.3, 128.3, 123.9, 115.6.

3-ethylquinoxalin-2(1*H*)-one (3b)

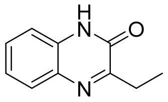

3b

Light yellow solid, 65% yield; ^{1}H NMR (400 MHz, $CDCl_3$): δ (ppm) 12.40 (s, 1H), 7.85-7.83 (m, 1H), 7.51-7.47 (m, 1H), 7.38-7.32 (m, 2H), 3.03 (q, *J* = 8.0 Hz, 2H), 1.39 (t, *J* = 8.0 Hz, 3H); ^{13}C NMR (100 MHz, $CDCl_3$): δ (ppm) 162.5, 156.5, 132.8, 130.9, 129.6, 128.7, 124.1, 115.6, 26.8, 10.9.

1-methyl-3-phenylquinoxalin-2(1*H*)-one (3c)

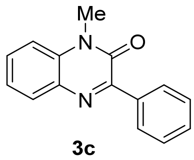

3c

Yellow white solid; 57% yield; known compound;[5] ^{1}H NMR (400 MHz, $CDCl_3$): δ (ppm) 8.31-8.28 (m, 2H), 7.96-7.94 (m, 1H), 7.60-7.54 (m, 1H), 7.50-7.47 (m, 3H), 7.38-7.33 (m, 2H), 3.78 (s, 3H); ^{13}C NMR (100 MHz, $CDCl_3$): δ (ppm) 154.7, 154.2, 136.1, 133.3, 133.1, 130.4, 130.3, 129.5, 128.1, 123.7, 113.6, 29.3.

4-phenyl-1,3-dihydro-2*H*-benzo[*b*][1,4]diazepin-2-one (3d)

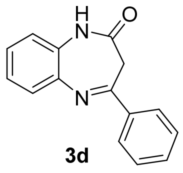

3d

White solid; 81% yield; known compound;[7] ^{1}H NMR (400 MHz, $CDCl_3$): δ (ppm) 8.88 (s, 1H), 8.12-8.10 (m, 2H), 7.52-7.47 (m, 4H), 7.28-7.24 (m, 2H), 7.12-7.10 (m, 1H), 3.58 (s, 2H);^{13}C NMR (100 MHz, $CDCl_3$): δ (ppm) 167.7, 158.8, 140.0, 137.6, 131.0, 129.0, 128.7, 128.3, 127.7, 126.5, 125.1, 121.8, 39.8.

III. General Procedure for Asymmetric Hydrogenation

In the argon-filled glovebox, a solution of **L5** (4.9 mg, 0.0055 mmol) and $[Ir(COD)Cl]_2$ (1.7 mg, 0.0025 mmol) in 1.0 mL anhydrous THF was stirred at room temperature for 45 min. 10 μL of the resulting solution transferred by syringe into a vial charged with **1** (0.05 mmol) in 1.0 mL anhydrous THF, then a solution of HCl (1.0 eq.) in dioxane (4 M) was added via syringe. The vials were transferred to an autoclave, which was then charged with 30 atm of H_2 and stirred at room temperature for 16 h. The hydrogen gas was released slowly and the solution was concentrated and passed through a short column of silica gel to remove the metal complex. The product was analyzed by NMR spectroscopy for conversion and chiral HPLC for ee values.

(*S*)-3-phenyl-3,4-dihydro-2*H*-benzo[*b*][1,4]oxazin-2-one (2a)

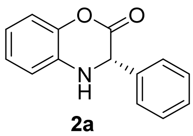

2a

White solid; >99% conversion; 93% yield, 10.5 mg; 99% ee; known compound;[3] $[\alpha]_D^{25}$ = +78.4 (c = 1.0, $CHCl_3$); The enantiomeric excess was determined by HPLC on Chiralcel OD-H column, hexane: isopropanol = 80:20; flow rate = 1.0 mL/min; UV detection at 220 nm; t_R = 16.6 min (major), 11.4 min (minor). ^1H NMR (400 MHz, $CDCl_3$): δ (ppm) 7.39-7.35 (m, 5H), 7.05-7.00 (m, 2H), 6.88-6.70 (m, 2H), 5.05 (d, *J* = 4.0 Hz, 1H), 4.27 (s, 1H); ^{13}C NMR (100 MHz, $CDCl_3$): δ (ppm) 165.2, 140.8, 136.3, 132.3, 128.97, 128.95, 127.5, 125.2, 120.4, 116.9, 114.8, 59.2.

(*S*)-7-methyl-3-phenyl-3,4-dihydro-2*H*-benzo[*b*][1,4]oxazin-2-one (2b)

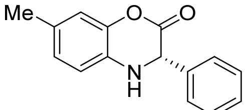

2b

Off-white solid, mp = 81-84 °C; >99% conversion; 94% yield, 11.2 mg; >99% ee; known compound;[8] $[\alpha]_D^{25}$ = +105.1 (c = 0.7, $CHCl_3$); The enantiomeric excess was determined by HPLC on Chiralcel OD-H column, hexane: isopropanol = 80:20; flow rate = 1.0 mL/min; UV detection at 210 nm; t_R = 21.0 min (major), 12.2 min (minor). ^1H NMR (400 MHz, $CDCl_3$): δ (ppm) 7.39-7.35 (m, 5H), 6.86-6.82 (m, 2H), 6.71 (d, *J* = 8.0 Hz, 1H), 5.02 (s, 1H), 4.15 (s, 1H), 2.29 (s, 3H); ^{13}C NMR (100 MHz, $CDCl_3$): δ (ppm) 165.5, 140.8, 136.4, 130.3, 129.8, 128.9, 127.5, 125.6, 117.3, 114.8, 59.4, 20.6.

(*S*)-6-methyl-3-phenyl-3,4-dihydro-2*H*-benzo[*b*][1,4]oxazin-2-one (2c)

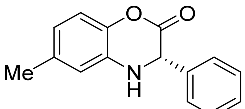

2c

Off-white solid; >99% conversion; 95% yield, 11.4 mg; >99% ee; known compound;[8] $[\alpha]_D^{25}$ = +115.3 (c = 0.7, $CHCl_3$); The enantiomeric excess was determined by HPLC on Chiralcel OD-H column, hexane: isopropanol = 80:20; flow rate = 1.0 mL/min; UV detection at 210 nm; t_R = 14.5 min (major), 10.2 min (minor). ^1H NMR (400 MHz, $CDCl_3$): δ (ppm) 7.41-7.36 (m, 5H), 6.93 (d, *J*

= 8.0 Hz, 1H), 6.66 (d, J = 8.0 Hz, 1H), 6.62 (s, 1H), 5.04 (s, 1H), 4.17 (s, 1H), 2.29 (s, 3H); ^{13}C NMR (100 MHz, $CDCl_3$): δ (ppm) 165.3, 138.8, 136.5, 135.0, 131.9, 128.96, 128.94, 127.4, 121.0, 116.6, 115.3, 59.3, 21.0.

(*S*)-6-fluoro-3-phenyl-3,4-dihydro-2*H*-benzo[*b*][1,4]oxazin-2-one (2d)

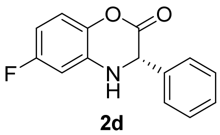

2d

Off-white solid, mp = 105-108 °C; >99% conversion; 93% yield, 11.3 mg; >99% ee; $[\alpha]_D^{25}$ = +109.2 (c = 0.7, $CHCl_3$); The enantiomeric excess was determined by HPLC on Chiralcel OD-H column, hexane: isopropanol = 80:20; flow rate = 1.0 mL/min; UV detection at 210 nm; t_R = 21.0 min (major), 12.7 min (minor). ^{1}H NMR (400 MHz, $CDCl_3$): δ (ppm) 7.40-7.38 (m, 5H), 7.00-6.96 (m, 1H), 6.58-6.52 (m, 2H), 5.07 (s, 1H), 4.38 (s, 1H); ^{13}C NMR (100 MHz, $CDCl_3$): δ (ppm) 164.6, 159.7 (d, J = 241.0 Hz), 136.84, 136.81, 136.0, 133.2 (d, J = 11.0 Hz), 129.1 (d, J = 7.0 Hz), 127.3, 117.8 (d, J = 10.0 Hz), 106.5 (d, J = 23.0 Hz), 101.8 (d, J = 27.0 Hz), 58.7. HRMS (ESI): $[M+H^+]$ Calc. 244.0774, found 244.0763.

(*S*)-6-chloro-3-phenyl-3,4-dihydro-2*H*-benzo[*b*][1,4]oxazin-2-one (2e)

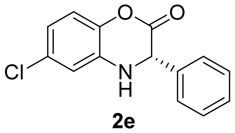

2e

Off-white solid, mp = 149-151 °C; >99% conversion; 94% yield, 12.2 mg; >99% ee; known compound;[5] $[\alpha]_D^{25}$ = +93.4 (c = 0.5, $CHCl_3$); The enantiomeric excess was determined by HPLC on Chiralpak AD-H column, hexane: isopropanol = 90:10; flow rate = 1.0 mL/min; UV detection at 220 nm; t_R = 20.4 min (major), 22.4 min (minor). ^{1}H NMR (400 MHz, $CDCl_3$): δ (ppm) 7.38-7.37 (m, 5H), 6.96 (d, J = 8.0 Hz, 1H), 6.84 (d, J = 4.0 Hz, 1H), 6.81 (s, 1H), 5.08 (s, 1H), 4.35 (s, 1H); ^{13}C NMR (100 MHz, $CDCl_3$): δ (ppm) 164.4, 139.3, 135.9, 133.1, 130.2, 129.2, 129.1, 127.3, 120.1, 118.0, 114.6, 58.7.

(*S*)-3-(4-methoxyphenyl)-3,4-dihydro-2*H*-benzo[*b*][1,4]oxazin-2-one (2f)

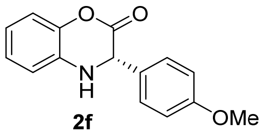

2f

Light yellow solid; >99% conversion; 92% yield, 11.7 mg; >99% ee; known compound;[3] $[\alpha]_D^{25}$ = +87.8 (c = 0.5, $CHCl_3$); The enantiomeric excess was determined by HPLC on Chiralcel OD-H column, hexane: isopropanol = 80:20; flow rate = 1.0 mL/min; UV detection at 220 nm; t_R = 36.6 min (major), 13.7 min (minor). ^{1}H NMR (400 MHz, $CDCl_3$): δ (ppm) 7.32 (d, *J* = 8.0 Hz, 2H), 7.05-7.02 (m, 2H), 6.90-6.86 (m, 3H), 6.81-6.79 (m, 1H), 4.99 (s, 1H), 4.22 (s, 1H), 3.79 (s, 3H); ^{13}C NMR (100 MHz, $CDCl_3$): δ (ppm) 165.6, 160.0, 140.9, 132.5, 128.8, 128.3, 125.1, 120.3, 116.9, 114.8, 114.3, 58.8, 55.3.

(*S*)-3-(4-fluorophenyl)-3,4-dihydro-2*H*-benzo[*b*][1,4]oxazin-2-one (2g)

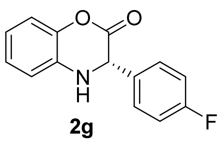

2g

Off-white solid; >99% conversion; 92% yield, 11.2 mg; >99% ee; known compound;[3] $[\alpha]_D^{25}$ = +93.1 (c = 1.0, $CHCl_3$); The enantiomeric excess was determined by HPLC on Chiralcel OD-H column, hexane: isopropanol = 80:20; flow rate = 1.0 mL/min; UV detection at 220 nm; t_R = 15.3 min (major), 8.6 min (minor). ^{1}H NMR (400 MHz, $CDCl_3$): δ (ppm) 7.42-7.38 (m, 2H), 7.09-7.04 (m, 4H), 6.89-6.82 (m, 2H), 5.04 (s, 1H), 4.23 (s, 1H); ^{13}C NMR (100 MHz, $CDCl_3$): δ (ppm) 165.1, 163.0 (d, *J* = 247.0 Hz), 140.9, 132.2, 132.0 (d, *J* = 3.0 Hz), 129.4 (d, *J* = 9.0 Hz), 125.2, 120.6, 117.0, 116.0 (d, *J* = 21.0 Hz), 114.9, 58.7.

(*S*)-3-(p-tolyl)-3,4-dihydro-2*H*-benzo[*b*][1,4]oxazin-2-one (2h)

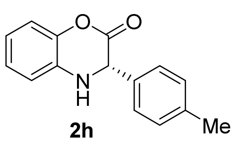

2h

Off-white solid; >99% conversion; 91% yield, 10.9 mg; >99% ee; known compound;[5] $[\alpha]_D^{25}$ = +99.8 (c = 0.5, $CHCl_3$); The enantiomeric excess was determined by HPLC on Chiralcel OD-H

column, hexane: isopropanol = 80:20; flow rate = 1.0 mL/min; UV detection at 220 nm; t_R = 33.2 min (major), 10.0 min (minor). ^{1}H NMR (400 MHz, $CDCl_3$): δ (ppm) 7.29 (d, *J* = 8.0 Hz, 2H), 7.18 (d, *J* = 8.0 Hz, 2H), 7.05-7.02 (m, 2H), 6.88-6.86 (m, 1H), 6.80 (d, *J* = 8.0 Hz, 1H), 5.01 (s, 1H), 4.23 (s, 1H), 2.34 (s, 3H); ^{13}C NMR (100 MHz, $CDCl_3$): δ (ppm) 165.4, 140.9, 138.9, 133.3, 132.5, 129.6, 127.3, 125.1, 120.3, 116.9, 114.8, 59.0, 21.2.

(*S*)-3-(4-(tert-butyl)phenyl)-3,4-dihydro-2*H*-benzo[*b*][1,4]oxazin-2-one (2i)

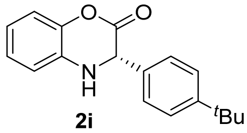

2i

Off-white solid; >99% conversion; 88% yield, 12.4 mg; >99% ee; known compound;[3] $[\alpha]_D^{25}$ = +98.8 (c = 0.5, $CHCl_3$); The enantiomeric excess was determined by HPLC on Chiralcel OD-H column, hexane: isopropanol = 80:20; flow rate = 1.0 mL/min; UV detection at 220 nm; t_R = 8.8 min (major), 7.3 min (minor). ^{1}H NMR (400 MHz, $CDCl_3$): δ (ppm) 7.39 (d, *J* = 8.0 Hz, 2H), 7.33 (d, *J* = 8.0 Hz, 2H), 7.05-7.02 (m, 2H), 6.86-6.84 (m, 1H), 6.80-6.78 (m, 1H), 5.03 (s, 1H), 4.22 (s, 1H), 1.30 (s, 9H); ^{13}C NMR (100 MHz, $CDCl_3$): δ (ppm) 165.4, 152.0, 140.9, 133.3, 132.5, 127.2, 125.9, 125.1, 120.3, 116.9, 114.8, 59.0, 34.6, 31.2.

(*S*)-3-(m-tolyl)-3,4-dihydro-2*H*-benzo[*b*][1,4]oxazin-2-one (2j)

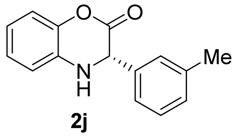

2j

Off-white solid, mp = 101-104 °C; >99% conversion; 95% yield, 11.4 mg; >99% ee; known compound;[8] $[\alpha]_D^{25}$ = +87.3 (c = 1.0, $CHCl_3$); The enantiomeric excess was determined by HPLC on Chiralcel OD-H column, hexane: isopropanol = 80:20; flow rate = 1.0 mL/min; UV detection at 210 nm; t_R = 15.4 min (major), 10.0 min (minor). ^{1}H NMR (400 MHz, $CDCl_3$): δ (ppm) 7.25-7.16 (m, 4H), 7.06-7.00 (m, 2H), 6.88-6.86 (m, 1H), 6.80 (d, *J* = 8.0 Hz, 1H), 5.01 (s, 1H), 4.24 (s, 1H), 2.34 (s, 3H); ^{13}C NMR (100 MHz, $CDCl_3$): δ (ppm) 165.4, 140.9, 138.8, 136.2, 132.4, 129.8, 128.8, 128.2, 125.1, 124.5, 120.3, 116.9, 114.8, 59.3, 21.4.

(*S*)-3-phenyl-3,4-dihydro-2*H*-naphtho[2,3-*b*][1,4]oxazin-2-one (2k)

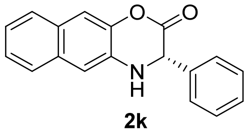

2k

Brown solid; >99% conversion; 95% yield, 13.1 mg; >99% ee; known compound of racemic product;[4] $[\alpha]_D^{25}$ = +165.4 (c = 0.5, $CHCl_3$); The enantiomeric excess was determined by HPLC on Chiralcel OD-H column, hexane: isopropanol = 80:20; flow rate = 1.0 mL/min; UV detection at 254 nm; t_R = 35.3 min (major), 25.8 min (minor). ^{1}H NMR (400 MHz, $CDCl_3$): δ (ppm) 7.73 (d, *J* = 8.0 Hz, 1H), 7.65 (d, *J* = 8.0 Hz, 1H), 7.48 (s, 1H), 7.43-7.32 (m, 7H), 7.15 (s, 1H), 5.19 (s, 1H), 4.55 (s, 1H); ^{13}C NMR (100 MHz, $CDCl_3$): δ (ppm) 165.2, 141.5, 136.4, 131.9, 131.6, 129.1, 128.5, 127.5, 127.3, 126.0, 125.8, 124.0, 113.7, 109.6, 59.2.

(*S*)-3-phenyl-3,4-dihydro-2*H*-naphtho[1,2-*b*][1,4]oxazin-2-one (2l)

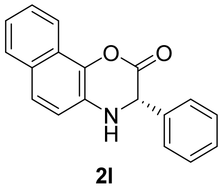

2l

Brown solid; >99% conversion; 91% yield, 12.5 mg; 97% ee; known compound of racemic product;[4] $[\alpha]_D^{25}$ = -22.4 (c = 0.5, $CHCl_3$); The enantiomeric excess was determined by HPLC on Chiralcel OD-H column, hexane: isopropanol = 80:20; flow rate = 1.0 mL/min; UV detection at 210 nm; t_R = 26.7 min (major), 22.4 min (minor). ^{1}H NMR (400 MHz, $CDCl_3$): δ (ppm) 8.09 (d, *J* = 8.0 Hz, 1H), 7.76 (d, *J* = 8.0 Hz, 1H), 7.56 (d, *J* = 8.0 Hz, 1H), 7.53-7.48 (m, 1H), 7.46-7.43 (m, 2H), 7.37-7.35 (m, 4H), 7.04 (d, *J* = 8.0 Hz, 1H), 5.17 (s, 1H), 4.37 (s, 1H); ^{13}C NMR (100 MHz, $CDCl_3$): δ (ppm) 164.9, 136.3, 133.3, 129.0, 128.8, 127.9, 127.7, 127.5, 127.0, 125.1, 124.1, 123.9, 119.3, 116.0, 59.3.

(*S*)-3-(thiophen-3-yl)-3,4-dihydro-2*H*-benzo[*b*][1,4]oxazin-2-one (2m)

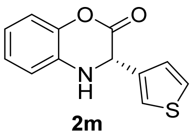

2m

Yellow solid; >99% conversion; 96% yield, 11.1 mg; >99% ee; known compound;[9] $[\alpha]_D^{25}$ = +53.4 (c = 0.5, $CHCl_3$); The enantiomeric excess was determined by HPLC on Chiralcel OD-H column, hexane: isopropanol = 80:20; flow rate = 1.0 mL/min; UV detection at 210 nm; t_R = 14.3 min (major), 10.6 min (minor). ^1H NMR (400 MHz, $CDCl_3$): δ (ppm) 7.33-7.31 (m, 1H), 7.27-7.26 (m, 1H), 7.09-7.07 (m, 1H), 7.05-7.01 (m, 2H), 6.87-6.81 (m, 2H), 5.20 (s, 1H), 4.31 (s, 1H); ^{13}C NMR (100 MHz, $CDCl_3$): δ (ppm) 164.6, 140.8, 137.0, 132.0, 127.0, 126.3, 125.2, 123.6, 120.5, 116.9, 115.0, 55.2.

(*S*)-3-methyl-3,4-dihydro-2*H*-benzo[*b*][1,4]oxazin-2-one (2n)

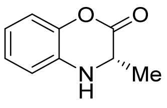

2n

Off-white solid; >99% conversion; 91% yield, 7.4 mg; 97% ee; known compound;[10] $[\alpha]_D^{25}$ = +17.8 (c = 1.0, $CHCl_3$); The enantiomeric excess was determined by HPLC on Chiralcel OD-H column, hexane: isopropanol = 90:10; flow rate = 1.0 mL/min; UV detection at 210 nm; t_R = 17.2 min (major), 13.6 min (minor). ^1H NMR (400 MHz, $CDCl_3$): δ (ppm) 7.04-6.98 (m, 2H), 6.88-6.84 (m, 1H), 6.79-6.76 (m, 1H), 3.98 (q, J = 4.0 Hz, 1H), 1.55 (d, J = 4.0 Hz, 3H); ^{13}C NMR (100 MHz, $CDCl_3$): δ (ppm) 167.3, 141.4, 132.9, 124.9, 120.4, 116.9, 115.0, 50.5, 17.2.

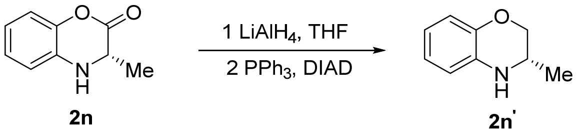

Experimental value for (*S*)-**2n'**: $[\alpha]_D^{20}$ = +15.4 (c = 0.4, $CHCl_3$); Literature data $[\alpha]_D^{20}$ = +19.8 (c = 1.0, $CHCl_3$).[11]

(+)-3-isopropyl-3,4-dihydro-2H-benzo[b][1,4]oxazin-2-one (2o)

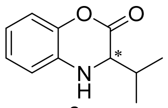

2o

White solid; mp = 61-63 °C; >99% conversion; 95% yield, 9.0 mg; 98% ee; known compound;[3] $[\alpha]_D^{25}$ = +24.0 (c = 0.5, $CHCl_3$); The enantiomeric excess was determined by HPLC on Chiralcel OD-H column, hexane: isopropanol = 90:10; flow rate = 1.0 mL/min; UV detection at 254 nm; t_R = 8.2 min (minor), 10.8 min (major). ^{1}H NMR (400 MHz, $CDCl_3$) δ 7.00-6.96 (m, 2H), 6.83-6.75 (m, 2H), 3.98 (s, 1H), 3.77-3.76 (m, 1H), 2.31-2.13 (m, 1H), 1.07 (d, J = 6.9 Hz, 3H), 1.01 (d, J = 6.7 Hz, 3H). ^{13}C NMR (100 MHz, $CDCl_3$) δ 165.7, 140.7, 132.1, 124.9, 119.8, 116.6, 114.7, 60.4, 29.8, 18.9, 17.6.

(+)-3-phenethyl-3,4-dihydro-2H-benzo[b][1,4]oxazin-2-one (2p)

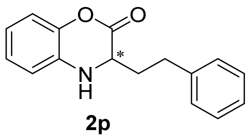

2p

Brown solid; >99% conversion; 91% yield, 11.5 mg; 91% ee; known compound;[1] $[\alpha]_D^{25}$ = +6.5 (c = 1.0, $CHCl_3$); The enantiomeric excess was determined by HPLC on Chiralcel OD-H column, hexane: isopropanol = 80:20; flow rate = 1.0 mL/min; UV detection at 210 nm; t_R = 13.7 min (minor), 22.4 min (major).^{1}H NMR (400 MHz, $CDCl_3$) δ 7.31 (t, J = 7.3 Hz, 2H), 7.23 (d, J = 8.0 Hz, 3H), 7.02-6.96 (m, 2H), 6.86-6.82 (m, 1H), 6.66-6.64 (m, 1H), 3.93 (ddd, J = 7.3, 5.2, 2.1 Hz, 1H), 3.74 (s, 1H), 2.86-2.80 (m, 2H), 2.32-2.27 (m, 1H), 2.11-2.05 (m, 1H). ^{13}C NMR (100 MHz, $CDCl_3$) δ 166.4, 141.1, 140.3, 132.1, 128.7, 128.4, 126.5, 124.9, 120.4, 116.8, 115.2, 54.4, 32.6, 31.7.

(*S*)-7-((2-chloro-5-fluoropyrimidin-4-yl)amino)-3-methyl-3,4-dihydro-2*H*-benzo[*b*][1,4]oxazin-2-one (2q)

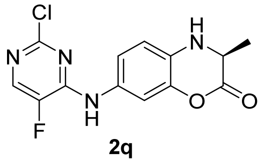

2q

Off-white solid; >99% conversion; 92% yield, 14.1 mg; 91% ee; $[\alpha]_D^{25}$ = +17.8 (c = 1.0, $CHCl_3$); The enantiomeric excess was determined by HPLC on Chiralcel OD-H column, hexane: isopropanol = 80:20; flow rate = 1.0 mL/min; UV detection at 210 nm; t_R = 18.1 min (major), 26.2 min (minor). ^{1}H NMR (400 MHz, C_2D_6SO): δ (ppm) 9.90 (s, 1H), 8.27 (d, J = 4.0 Hz, 1H), 7.41 (d, J = 4.0 Hz, 1H), 7.26-7.24 (m, 1H), 6.86-6.84 (m, 1H), 6.37 (s, 1H), 4.03-3.98 (m, 1H), 1.38 (d, J = 8.0 Hz, 3H); ^{13}C NMR (100 MHz, C_2D_6SO): δ (ppm) 167.9, 153.5, 151.5 (d, J = 11.0 Hz), 145.6 (d, J = 257.0 Hz), 143.2, 141.5 (d, J = 20.0 Hz), 140.8, 131.9, 129.7, 119.2, 115.0, 110.8, 50.0, 17.0.

(*S*)-3-phenyl-3,4-dihydroquinoxalin-2(1*H*)-one (4a)

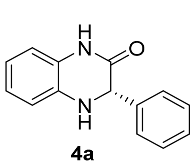

4a

Off-white solid; >99% conversion; 90% yield, 10.1 mg; >99% ee; known compound;[5] $[\alpha]_D^{25}$ = +78.6 (c = 0.5, $CHCl_3$); The enantiomeric excess was determined by HPLC on Chiralcel OD-H column, hexane: isopropanol = 80:20; flow rate = 1.0 mL/min; UV detection at 210 nm; t_R = 16.0 min (major), 26.1 min (minor). ^{1}H NMR (400 MHz, $CDCl_3$): δ (ppm) 9.09 (s, 1H), 7.41-7.40 (m, 2H), 7.32-7.28 (m, 3H), 6.92-6.88 (m, 1H), 6.76-6.68 (m, 3H), 5.06 (s, 1H), 4.30 (s, 1H); ^{13}C NMR (100 MHz, $CDCl_3$): δ (ppm) 167.2, 139.0, 132.8, 128.8, 128.4, 127.1, 124.7, 124.0, 119.3, 115.7, 113.6, 60.6.

(+)-3-ethyl-3,4-dihydroquinoxalin-2(1*H*)-one (4b)

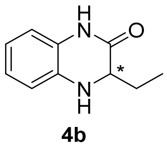

4b

Viscous liquid; >99% conversion; 91% yield, 8.0 mg; 99% ee; $[\alpha]_D^{25}$ = +27.0 (c = 0.5, $CHCl_3$), The enantiomeric excess was determined by HPLC on Chiralcel OD-H column, hexane: isopropanol = 80:20; flow rate = 1.0 mL/min; UV detection at 220 nm; t_R = 7.9 min (major), 9.5 min (minor). ^{1}H NMR (400 MHz, $CDCl_3$): δ (ppm) 9.10 (s, 1H), 6.90-6.86 (m, 1H), 6.77-6.72 (m, 2H), 6.68 (d,

J = 8.0 Hz, 1H), 3.99 (s, 1H), 3.89-3.86 (m, 1H), 1.90-1.75 (m, 2H), 1.04 (t, J = 8.0 Hz, 3H); ^{13}C NMR (100 MHz, $CDCl_3$): δ (ppm) 169.2, 133.0, 125.2, 123.8, 119.2, 115.4, 113.9, 57.5, 25.0, 9.6.

(*S*)-1-methyl-3-phenyl-3,4-dihydroquinoxalin-2(1*H*)-one (4c)

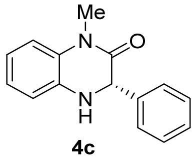

4c

Off-white solid; >99% conversion; 91% yield, 10.8 mg; 98% ee; known compound;[5] $[\alpha]_D^{25}$ = +115.9 (c = 1.0, $CHCl_3$); The enantiomeric excess was determined by HPLC on Chiralpak AD-H column, hexane: isopropanol = 80:20; flow rate = 1.0 mL/min; UV detection at 210 nm; t_R = 11.5 min (major), 14.9 min (minor). ^{1}H NMR (400 MHz, $CDCl_3$): δ (ppm) 7.38-7.36 (m, 2H), 7.31-7.29 (m, 3H), 6.98-6.86 (m, 3H), 6.75-6.73 (m, 1H), 5.05 (s, 1H), 4.36 (s, 1H), 3.38 (s, 3H); ^{13}C NMR (100 MHz, $CDCl_3$): δ (ppm) 166.0, 139.0, 134.4, 128.7, 128.3, 127.1, 123.7, 119.5, 114.8, 113.9, 60.8, 29.2.

(*R*)-1,3,4,5-Tetrahydro-4-phenyl-2*H*-1,5-benzodiazepin-2-one (4d)

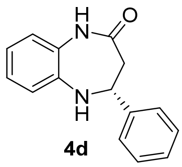

4d

Solid; >99% conversion; 93% yield, 11.1 mg; >99% ee; known compound;[12] $[\alpha]_D^{25}$ = -115.9 (c = 1.0, CH_2Cl_2); The enantiomeric excess was determined by HPLC on Chiralcel OJ-H column, hexane: isopropanol = 80:20; flow rate = 1.0 mL/min; UV detection at 210 nm; t_R = 32.6 min (major), 18.9 min (minor). ^{1}H NMR (400 MHz, $CDCl_3$): δ (ppm) 8.18 (s, 1H), 7.38-7.33 (m, 5H), 7.09-7.05 (m, 1H), 6.96-6.94 (m, 2H), 6.85-6.83 (m, 1H), 5.04-5.01 (m, 1H), 3.84 (s, 1H), 2.92-2.86 (m, 1H), 2.76-2.72 (m, 1H); ^{13}C NMR (100 MHz, $CDCl_3$): δ (ppm) 172.0, 144.3, 138.3, 129.0, 128.1, 127.8, 126.1, 125.9, 122.4, 121.5, 121.1, 63.3, 41.8.

IV. Linear Effect of the Hydrogenation of Substrate 1a

Table S1. Linear effect for Ir/ligand **L5**-catalyzed asymmetric hydrogenation of **1a**.

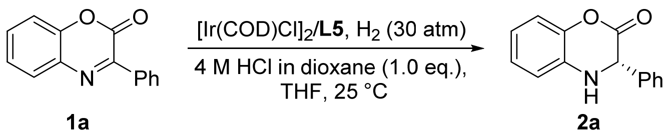

entry	ee of **L5** (%)	conv. (%)	ee of **2a**(%)
1	0	>99	3
2	10	>99	13
3	20	>99	24
4	40	>99	45
5	60	>99	64
6	80	>99	81
7	90	>99	92
8	>99	>99	99

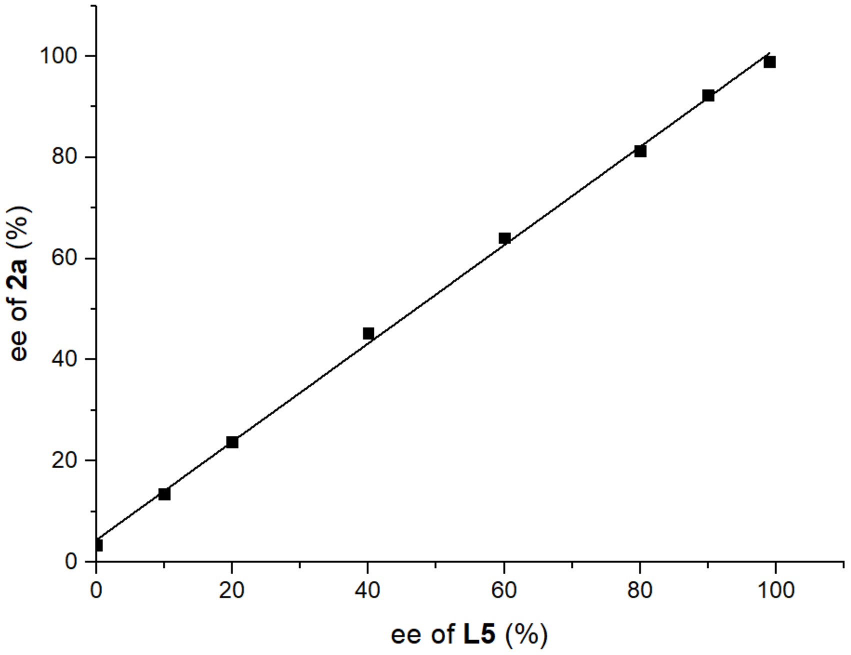

Figure S1. Linear effect of the hydrogenation of substrate **1a** using ligand **L5** with different ee values

V. Reference

1. Z.-Y. Xue, Y. Jiang, X.-Z. Peng, W.-C. Yuan, X.-M. Zhang, *Adv. Syn. Catal.* **2010**, *352*, 2132-2136.
2. a) S. Yan, L. Ye, M. Liu, J. Chen, J. Ding, W. Gao, X. Huang, H. Wu, *RSC Adv.* **2014**, *4*, 16705-16709. b) D.-J. Zhang, W.-F. Sun, Z.-J. Zhong, R.-M. Gao, H. Yi, Y.-H. Li, Z.-G. Peng, Z.-R. Li, *Molecules* **2014**, *19*, 925-939. c) W.-G. Su, H. Jia, W. Zhang, Y. Cui, X. Yan, Y. Ren, J. Duan, Y. Sai, U.S. Pat. Appl. Publ., 20080255172, 16 Oct 2008. d) Y. C. Teo, S. N. Riduan, Y. Zhang, *Green Chem.* **2013**, *15*, 2365-2368. e) Y. Kamada, N. Sakai, S. Sogabe, K. Ida, H. Oki, K. Sakamoto, W. Lane, G. Snell, M. Iida, Y. Imaeda, J. Sakamoto, J. Matsui, *J. Med. Chem.* **2017**, *60*, 4358-4368.
3. L.-Q. Lu, Y. Li, K. Junge, M. Beller, *J. Am. Chem. Soc.* **2015**, *137*, 2763-2768.
4. S. Nonell, L. R. Ferreras, A. Cañete, E. Lemp, G. Günther, N. Pizarro, A. L. Zanocco, *J. Org. Chem.* **2008**, *73*, 5371-5378.
5. J. L. Núñez-Rico, A. Vidal-Ferran, *Org. Lett.* **2013**, *15*, 2066-2069.
6. a) K. R. Rao, A. Raghunadh, R. Mekala, S. B. Meruva, K. R. Ganesh, T. Krishna, D. Kalita, E. Laxminarayana, M. Palc, *J. Heterocyclic. Chem.* **2016**, *53*, 901-908. b) L. Shi, J. Zhou, J. Wu, J. Cao, Y. Shen, H. Zhou, X. Li, *Bioorg. Med. Chem.* **2016**, *24*, 1840-1852.
7. H. Mtiraoui, R. Gharbi, M. Msaddek, Y. Bretonniere, C. Andraud, C. Sabot, P.-Y. Renard, *J. Org. Chem.* **2016**, 81, 4720-4727.
8. Q.-A. Chen, K. Gao, Y. Duan, Z.-S. Ye, L. Shi, Y. Yang, Y.-G. Zhou, *J. Am. Chem. Soc.* **2012**, *134*, 2442-2448.
9. X. Zhang, B. Xu, M.-H. Xu, *Org. Chem. Front.* **2016**, *3*, 944-948.
10. R. I. Storer, D. E. Carrera, Y. Ni, D. W. C. MacMillan, *J. Am. Chem. Soc.* **2006**, *128*, 84-86.
11. V. N. Charushin, V. P. Krasnov, G. L. Levit, M. A. Korolyova, M. I. Kodess, O. N. Chupakhin, M. H. Kim, H. S. Lee, Y. J. Park, K.-C. Kim, *Tetrahedron: Asymmetry* **1999**, *10*, 2691-2702.
12. C. Fang, J. Cao, K. Sun, J. Zhu, T. Lu, D. Du, *Chem. Eur. J.* **2018**, *24*, 2103-2108.

VI. NMR Spectra

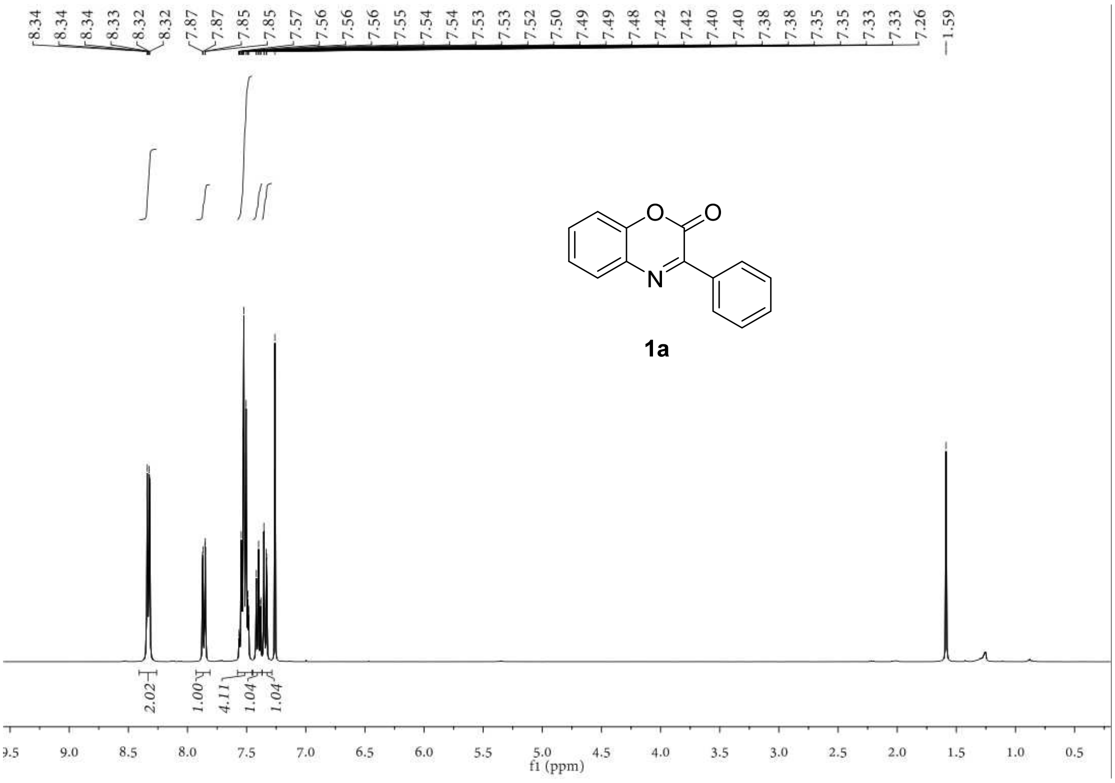

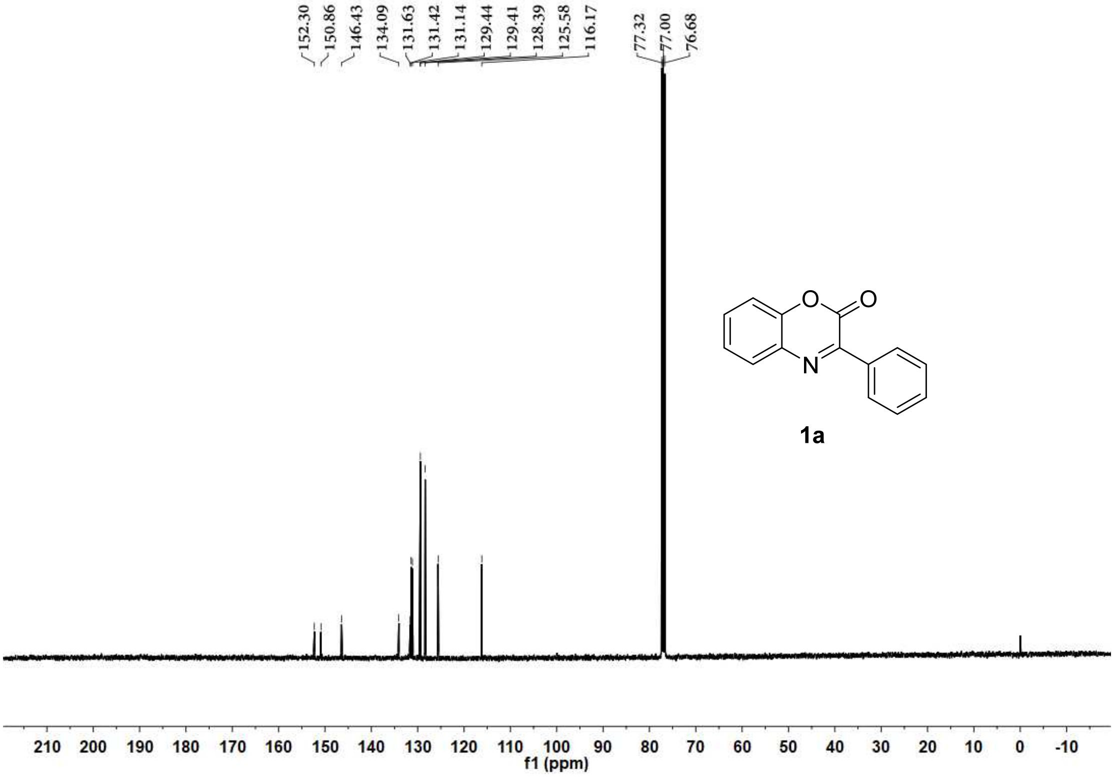

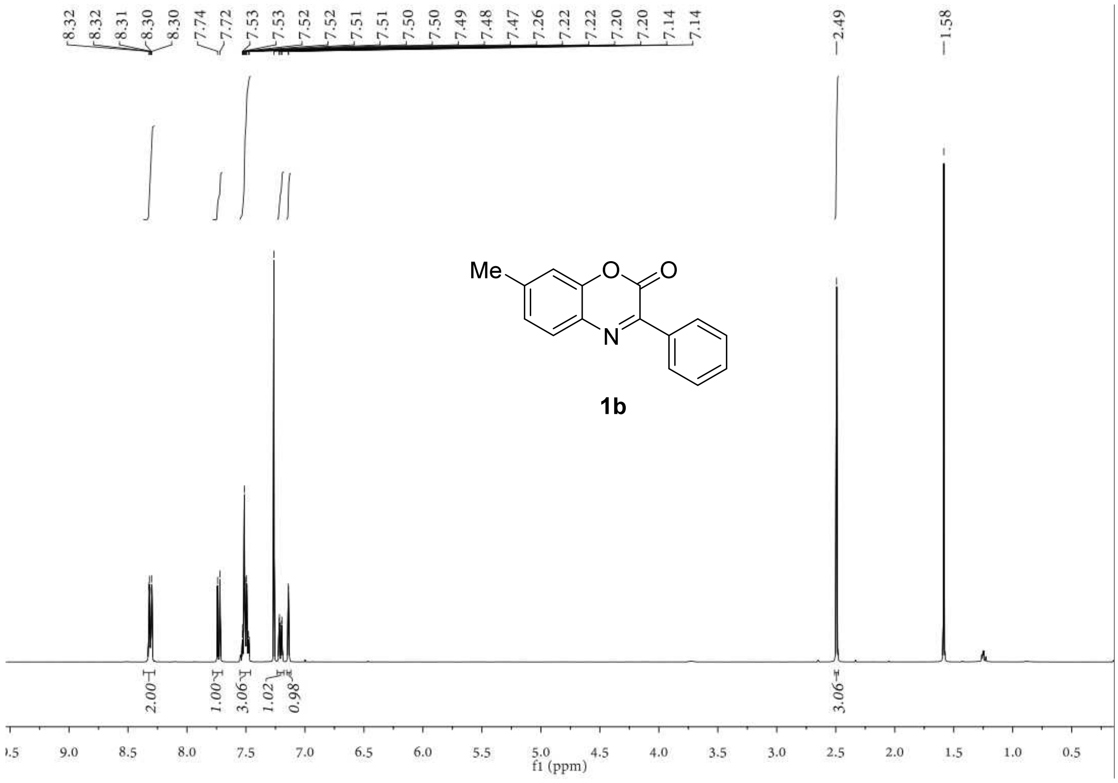
8.32
8.32
8.31
8.30
8.30
7.74
7.72
7.53
7.53
7.52
7.52
7.51
7.51
7.50
7.50
7.49
7.48
7.47
7.26
7.22
7.22
7.20
7.20
7.14
7.14
2.49
1.58
Me
O
O
N
1b
2.00
1.00
3.06
1.02
0.98
3.06
f1 (ppm)

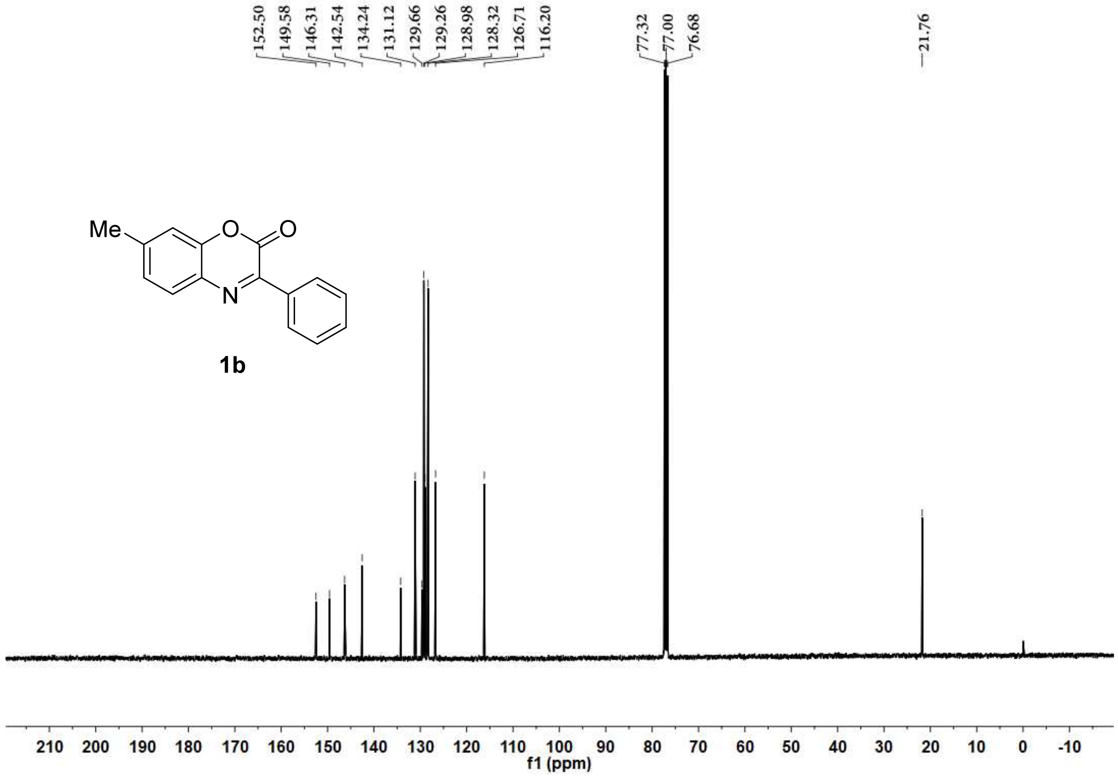
152.50
149.58
146.31
142.54
134.24
131.12
129.66
129.26
128.98
128.32
126.71
116.20
77.32
77.00
76.68
21.76
Me
O
O
N
1b
f1 (ppm)

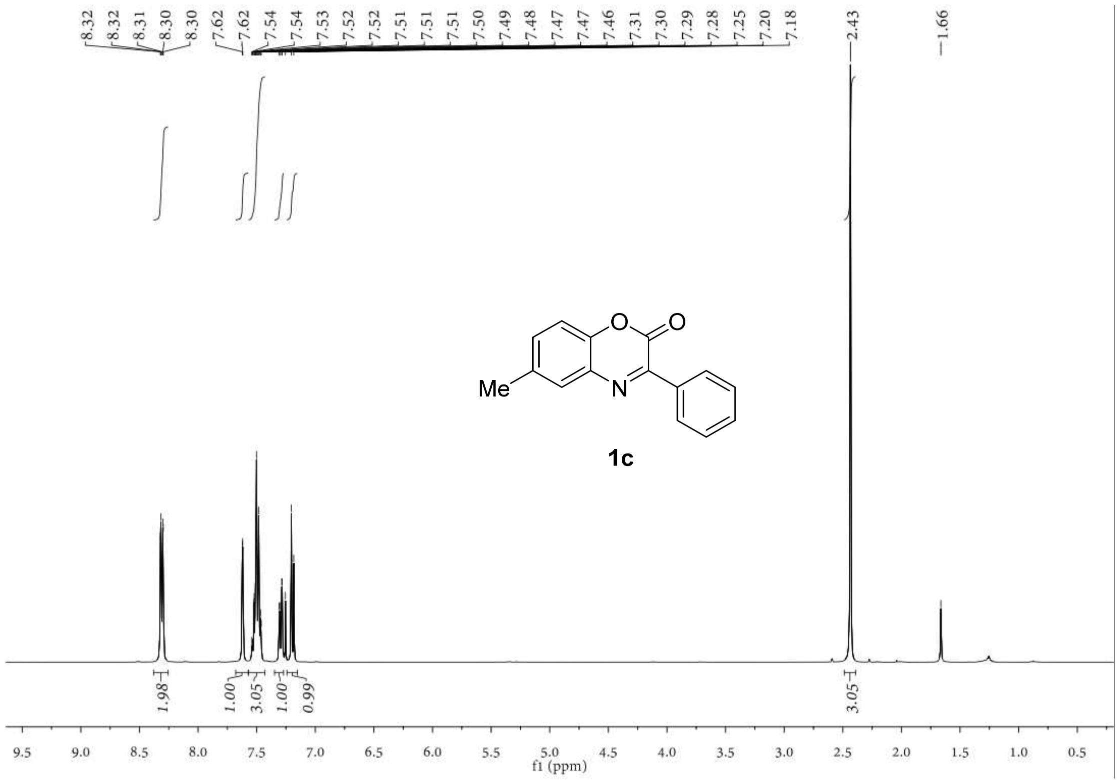

8.32
8.32
8.31
8.30
8.30
7.62
7.62
7.54
7.54
7.53
7.52
7.52
7.51
7.51
7.51
7.50
7.49
7.48
7.47
7.47
7.46
7.31
7.30
7.29
7.28
7.25
7.20
7.18
2.43
1.66
Me
O
O
N
1c
1.98
1.00
3.05
1.00
0.99
3.05
f1 (ppm)

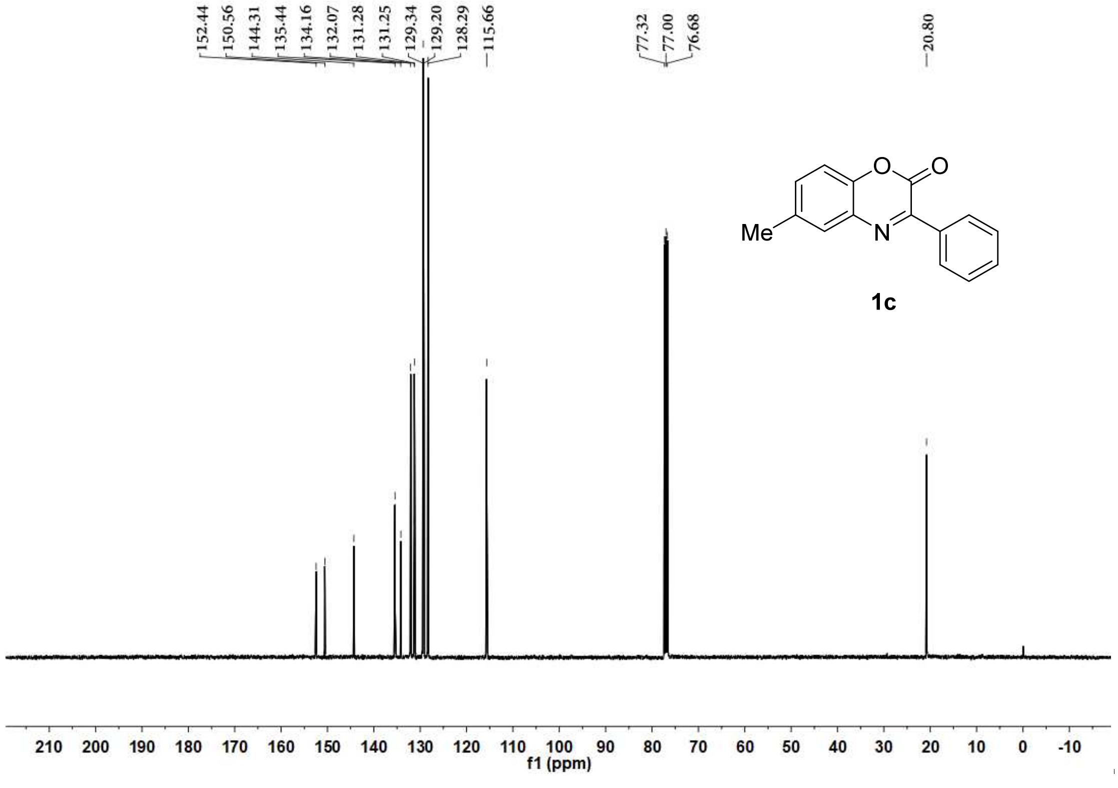

152.44
150.56
144.31
135.44
134.16
132.07
131.28
131.25
129.34
129.20
128.29
115.66
77.32
77.00
76.68
20.80
Me
O
O
N
1c
f1 (ppm)

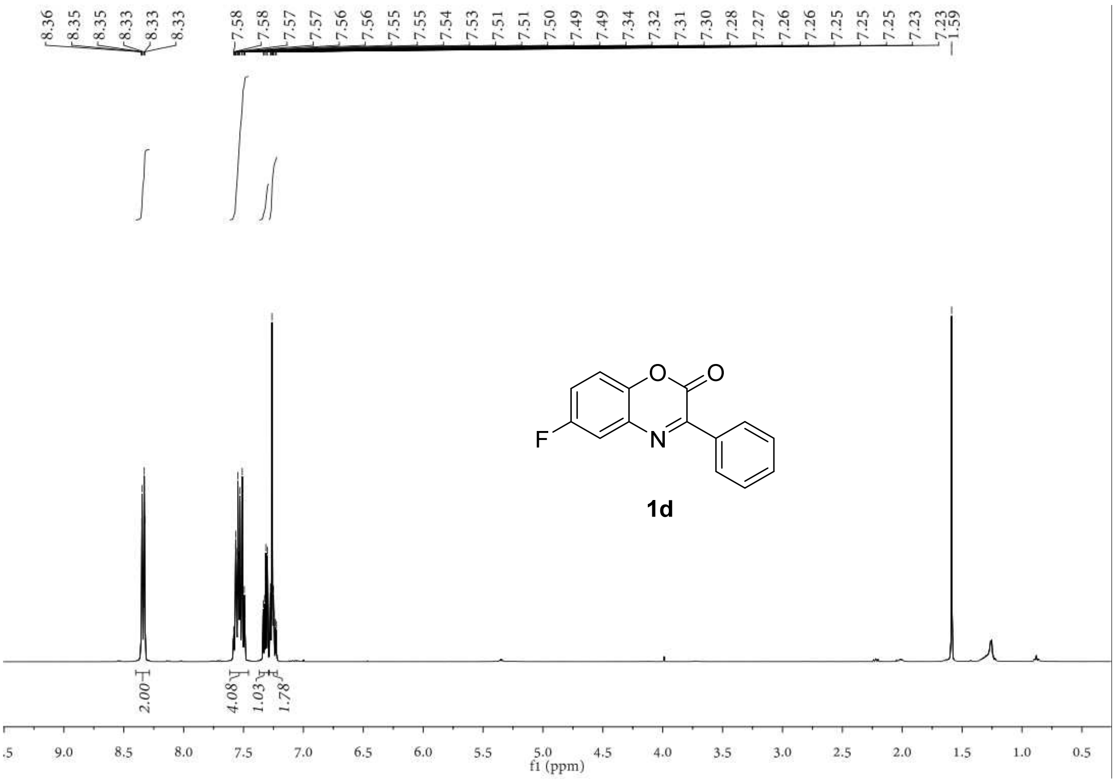

8.36
8.35
8.35
8.33
8.33
8.33
7.58
7.58
7.57
7.57
7.56
7.56
7.55
7.55
7.54
7.53
7.51
7.51
7.50
7.49
7.49
7.34
7.32
7.31
7.30
7.28
7.27
7.26
7.26
7.25
7.25
7.25
7.23
7.23
1.59
O
O
F
N
1d
2.00
4.08
1.03
1.78
f1 (ppm)

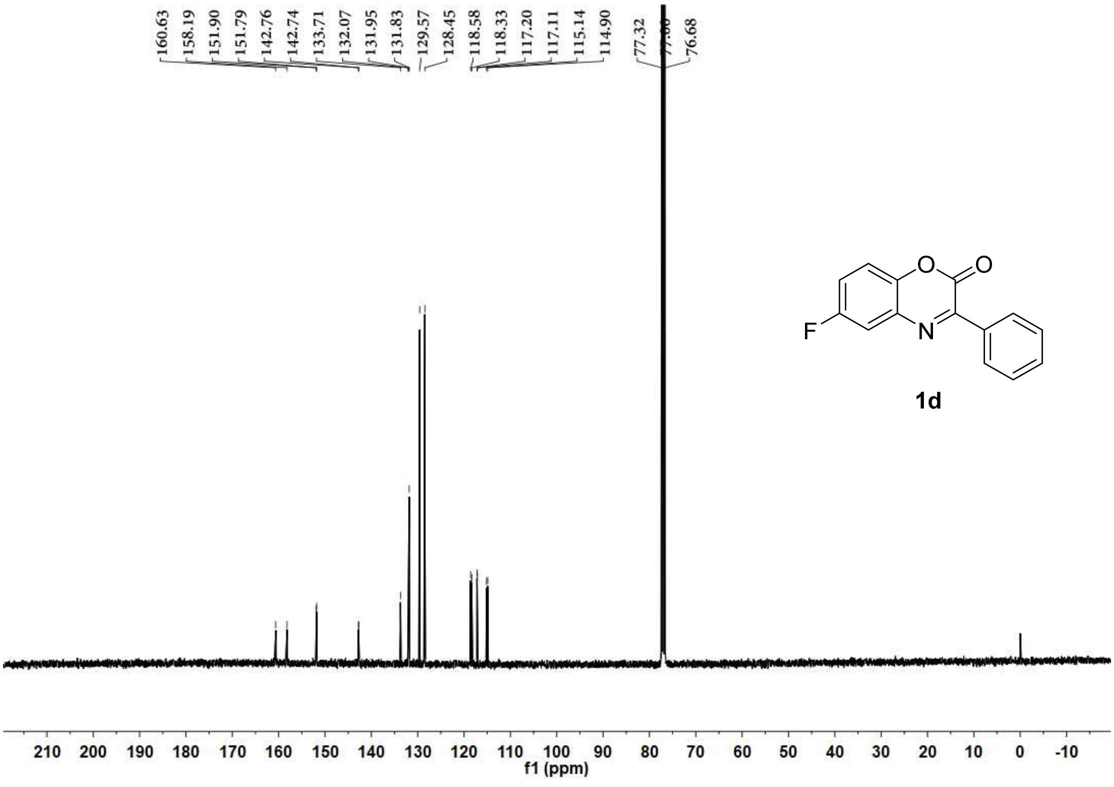

160.63
158.19
151.90
151.79
142.76
142.74
133.71
132.07
131.95
131.83
129.57
128.45
118.58
118.33
117.20
117.11
115.14
114.90
77.32
76.68
O
O
F
N
1d
f1 (ppm)

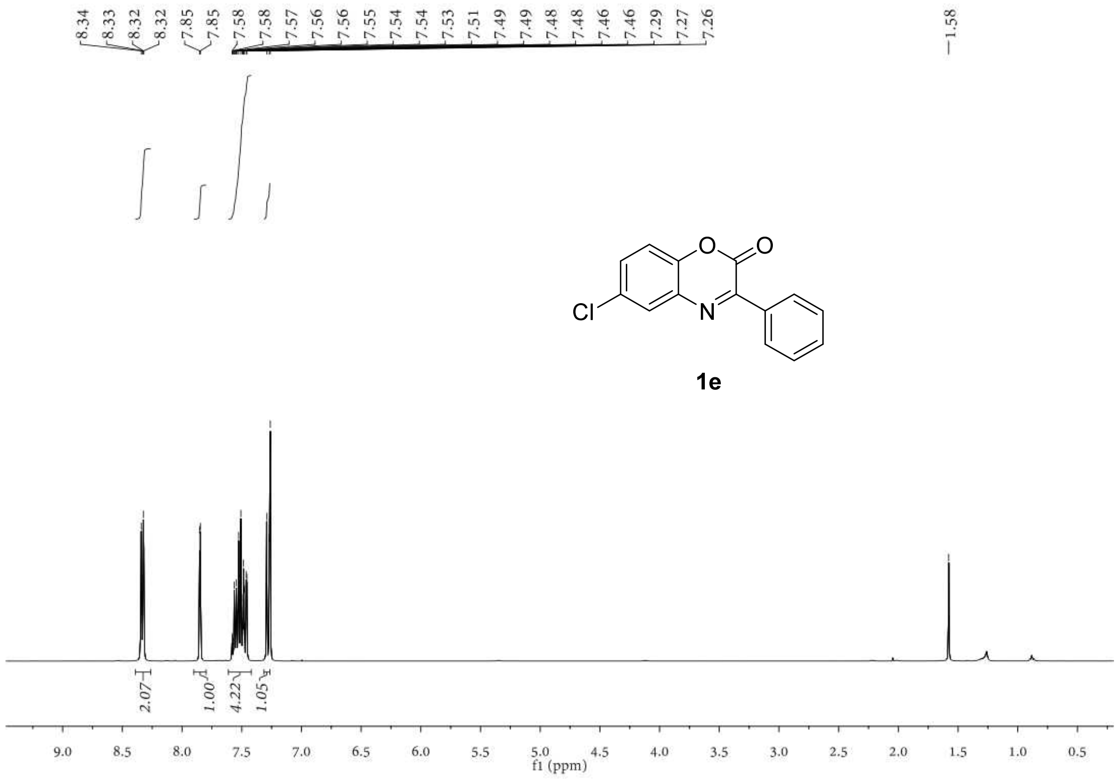
8.34
8.33
8.32
8.32
7.85
7.85
7.58
7.58
7.57
7.56
7.56
7.55
7.54
7.54
7.53
7.51
7.49
7.49
7.48
7.48
7.46
7.46
7.29
7.27
7.26
1.58
Cl
N
O
O
1e
2.07
1.00
4.22
1.05
9.0 8.5 8.0 7.5 7.0 6.5 6.0 5.5 5.0 4.5 4.0 3.5 3.0 2.5 2.0 1.5 1.0 0.5
f1 (ppm)

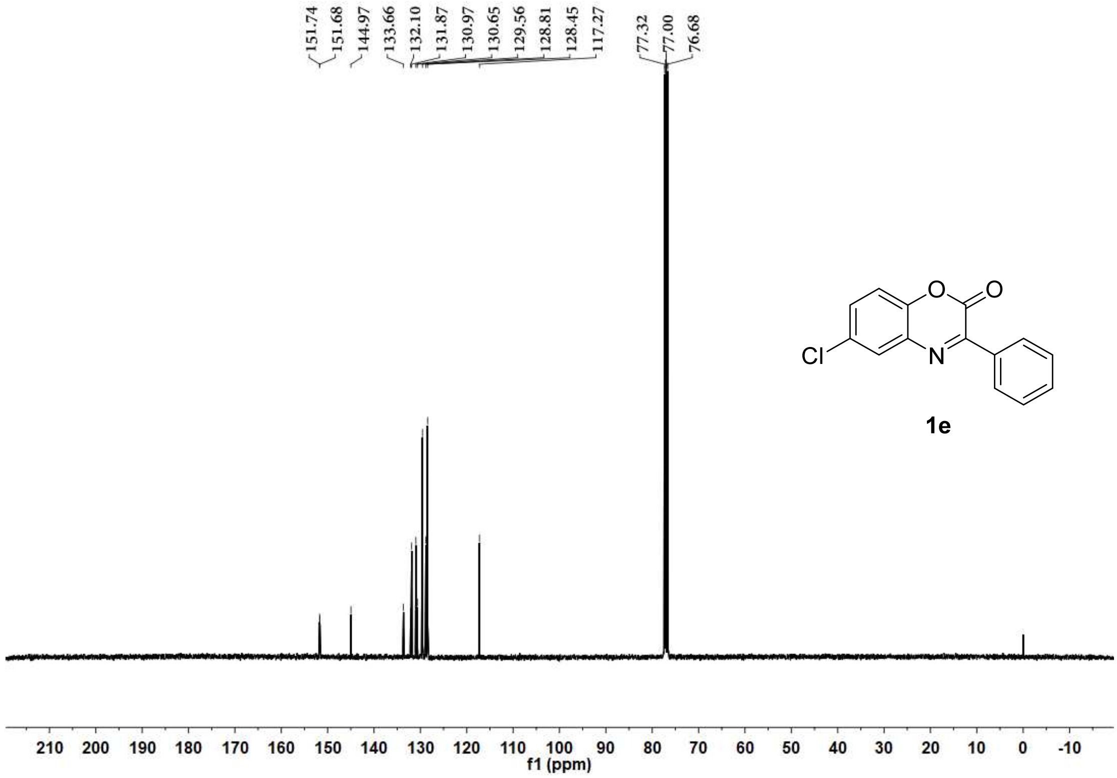
151.74
151.68
144.97
133.66
132.10
131.87
130.97
130.65
129.56
128.81
128.45
117.27
77.32
77.00
76.68
Cl
N
O
O
1e
210 200 190 180 170 160 150 140 130 120 110 100 90 80 70 60 50 40 30 20 10 0 -10
f1 (ppm)

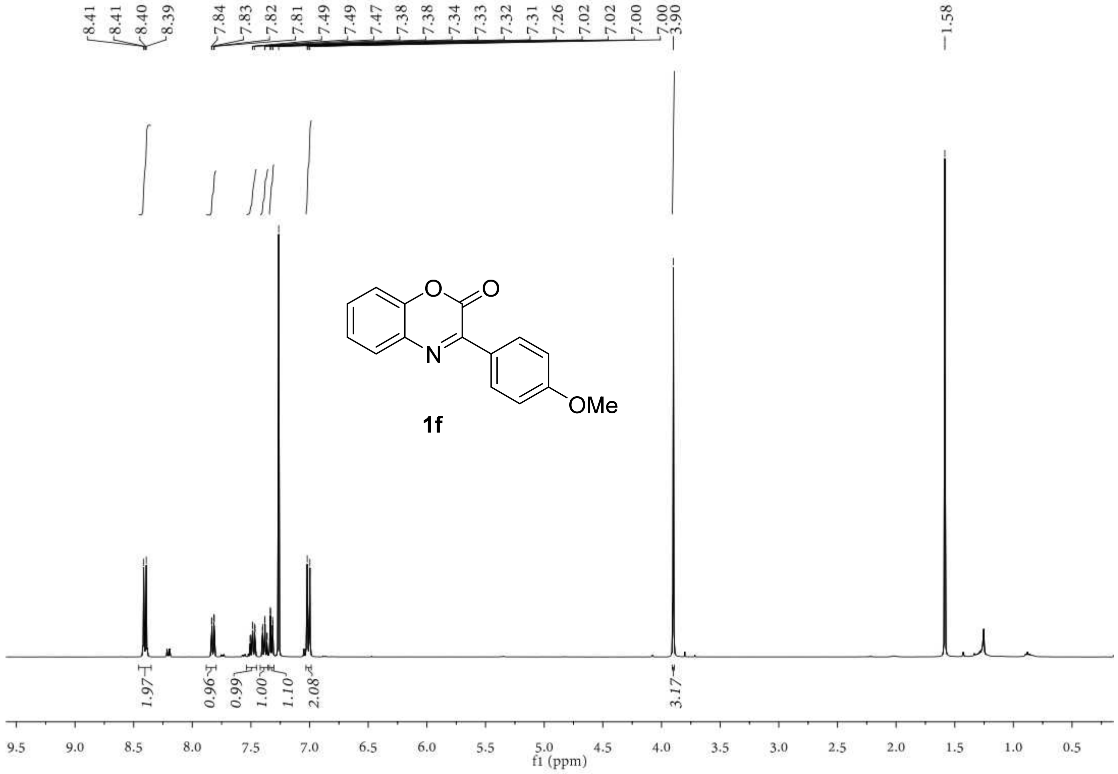
8.41
8.41
8.40
8.39
7.84
7.83
7.82
7.81
7.49
7.49
7.47
7.38
7.38
7.34
7.33
7.32
7.31
7.26
7.02
7.02
7.00
7.00
3.90
1.58
N
O
O
OMe
1f
1.97
0.96
0.99
1.00
1.10
2.08
3.17
9.5
9.0
8.5
8.0
7.5
7.0
6.5
6.0
5.5
5.0
4.5
4.0
3.5
3.0
2.5
2.0
1.5
1.0
0.5
f1 (ppm)

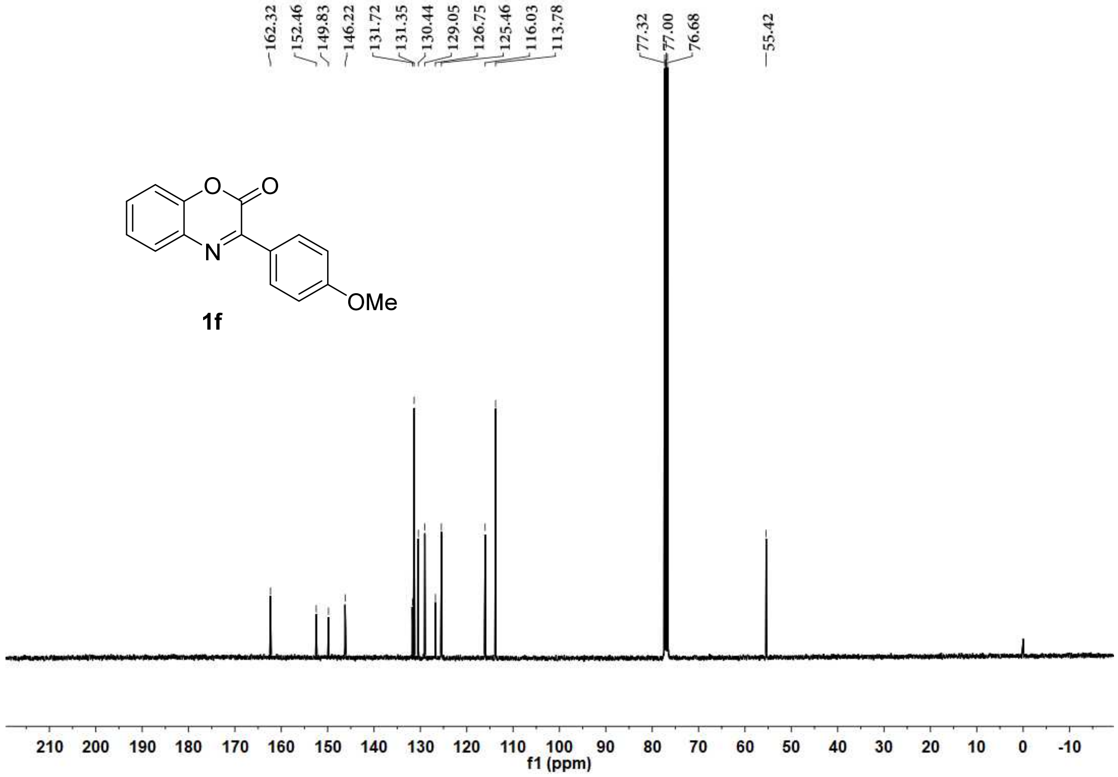
162.32
152.46
149.83
146.22
131.72
131.35
130.44
129.05
126.75
125.46
116.03
113.78
77.32
77.00
76.68
55.42
N
O
O
OMe
1f
210
200
190
180
170
160
150
140
130
120
110
100
90
80
70
60
50
40
30
20
10
0
-10
f1 (ppm)

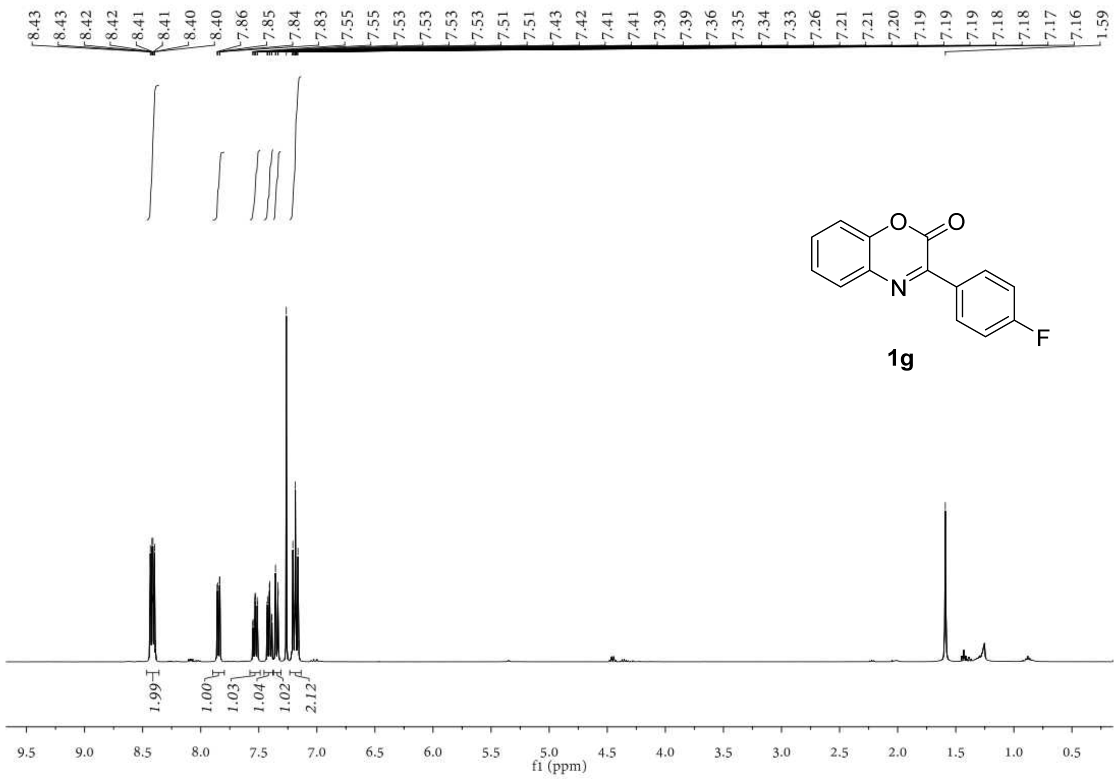
8.43
8.43
8.42
8.42
8.41
8.41
8.40
8.40
7.86
7.85
7.84
7.83
7.55
7.55
7.53
7.53
7.53
7.53
7.51
7.51
7.43
7.42
7.41
7.41
7.39
7.39
7.36
7.35
7.34
7.33
7.26
7.21
7.21
7.20
7.19
7.19
7.19
7.18
7.18
7.17
7.16
1.59
O
O
N
F
1g
1.99
1.00
1.03
1.04
1.02
2.12
9.5 9.0 8.5 8.0 7.5 7.0 6.5 6.0 5.5 5.0 4.5 4.0 3.5 3.0 2.5 2.0 1.5 1.0 0.5
f1 (ppm)

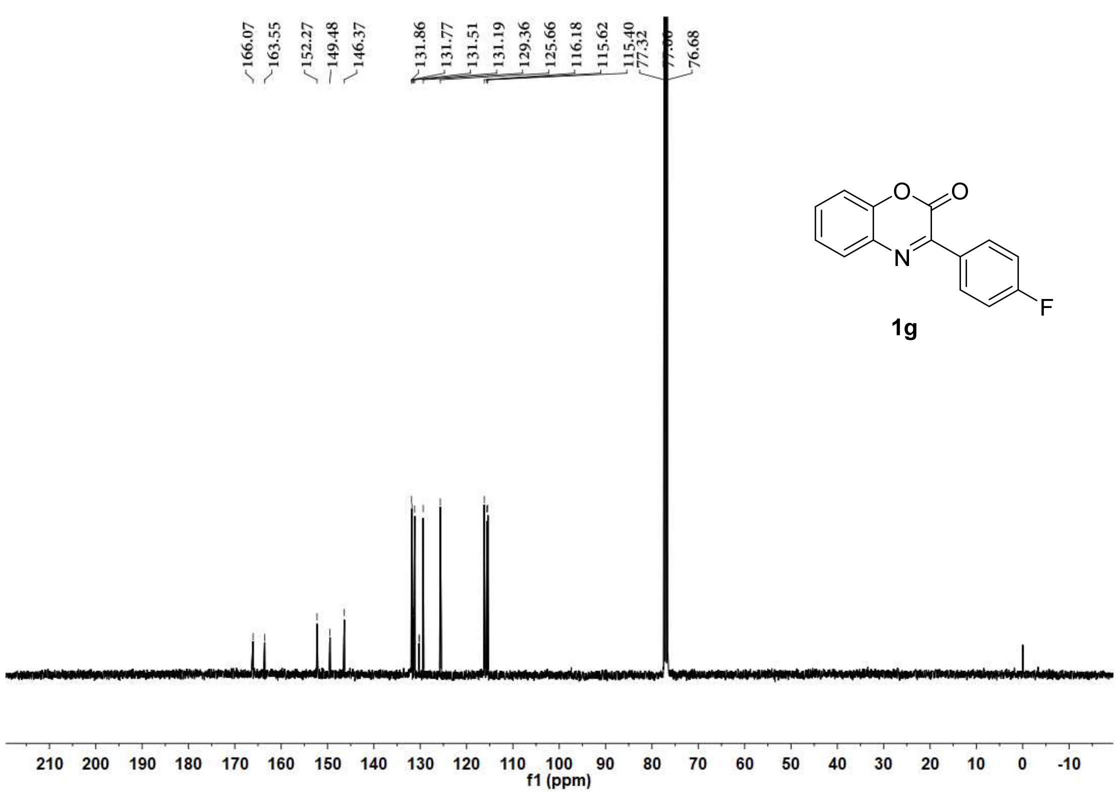
166.07
163.55
152.27
149.48
146.37
131.86
131.77
131.51
131.19
129.36
125.66
116.18
115.62
115.40
77.32
76.68
O
O
N
F
1g
210 200 190 180 170 160 150 140 130 120 110 100 90 80 70 60 50 40 30 20 10 0 -10
f1 (ppm)

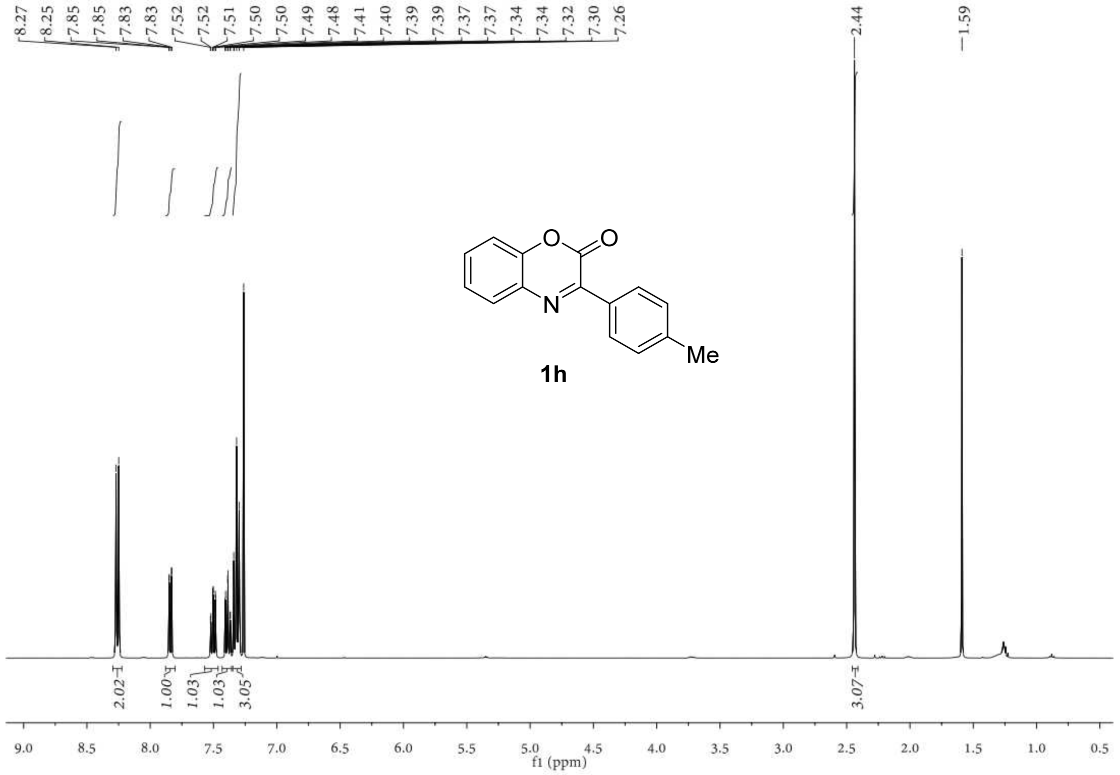

8.27
8.25
7.85
7.85
7.83
7.83
7.52
7.52
7.51
7.50
7.50
7.49
7.48
7.41
7.40
7.39
7.39
7.37
7.37
7.34
7.34
7.32
7.30
7.26
2.44
1.59
O
O
N
Me
1h
2.02
1.00
1.03
1.03
3.05
3.07
9.0 8.5 8.0 7.5 7.0 6.5 6.0 5.5 5.0 4.5 4.0 3.5 3.0 2.5 2.0 1.5 1.0 0.5
f1 (ppm)

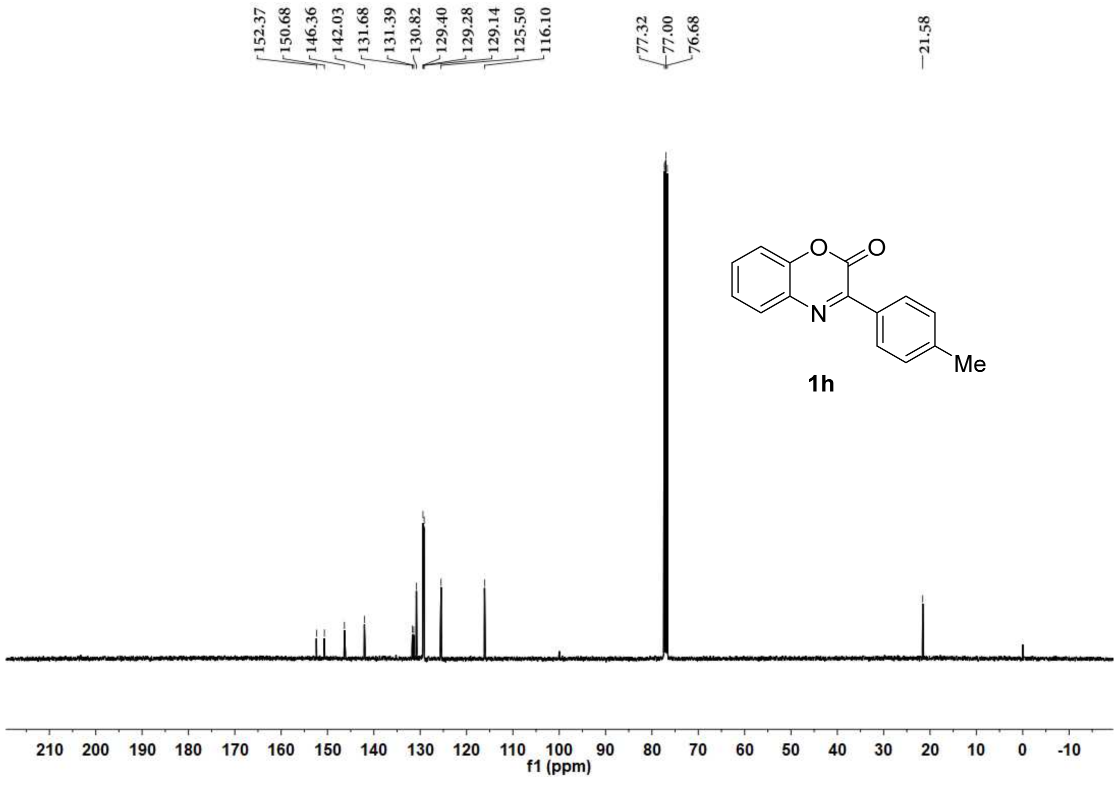

152.37
150.68
146.36
142.03
131.68
131.39
130.82
129.40
129.28
129.14
125.50
116.10
77.32
77.00
76.68
21.58
O
O
N
Me
1h
210 200 190 180 170 160 150 140 130 120 110 100 90 80 70 60 50 40 30 20 10 0 -10
f1 (ppm)

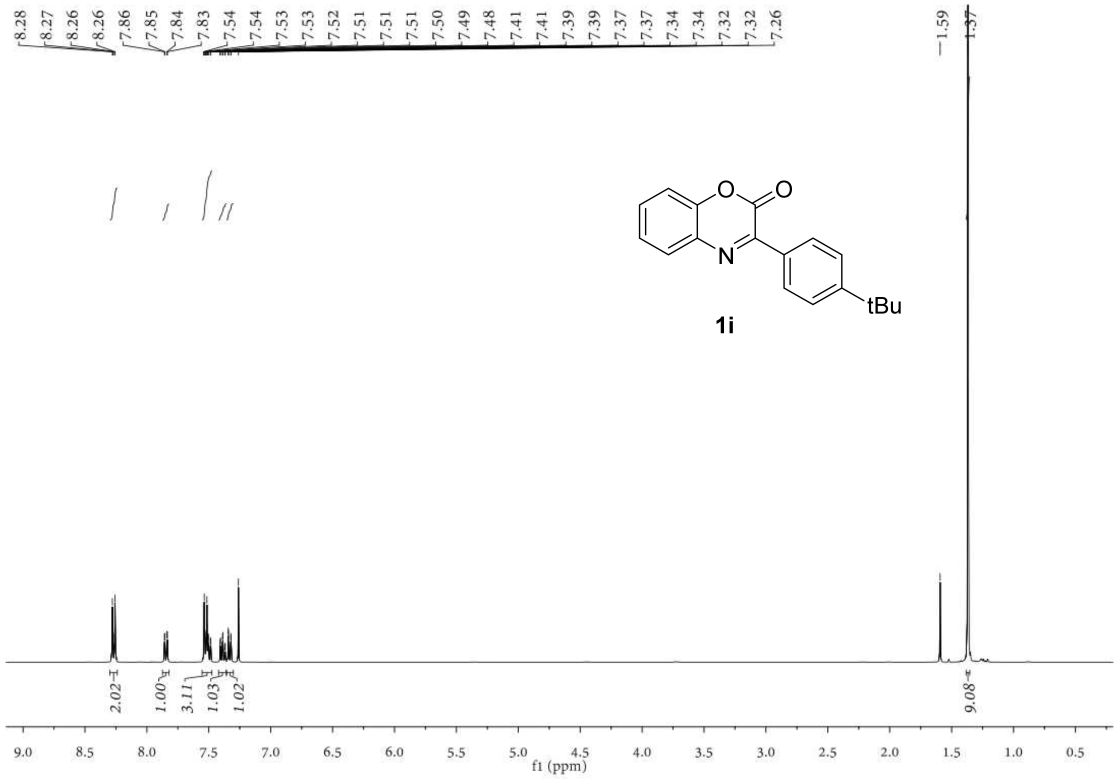
8.28 8.27 8.26 8.26 7.86 7.85 7.84 7.83 7.54 7.54 7.53 7.53 7.52 7.51 7.51 7.51 7.50 7.49 7.48 7.41 7.41 7.39 7.39 7.37 7.37 7.34 7.34 7.32 7.32 7.26
1.59 1.37
O
O
N
tBu
1i
2.02 1.00 3.11 1.03 1.02 9.08
9.0 8.5 8.0 7.5 7.0 6.5 6.0 5.5 5.0 4.5 4.0 3.5 3.0 2.5 2.0 1.5 1.0 0.5
f1 (ppm)

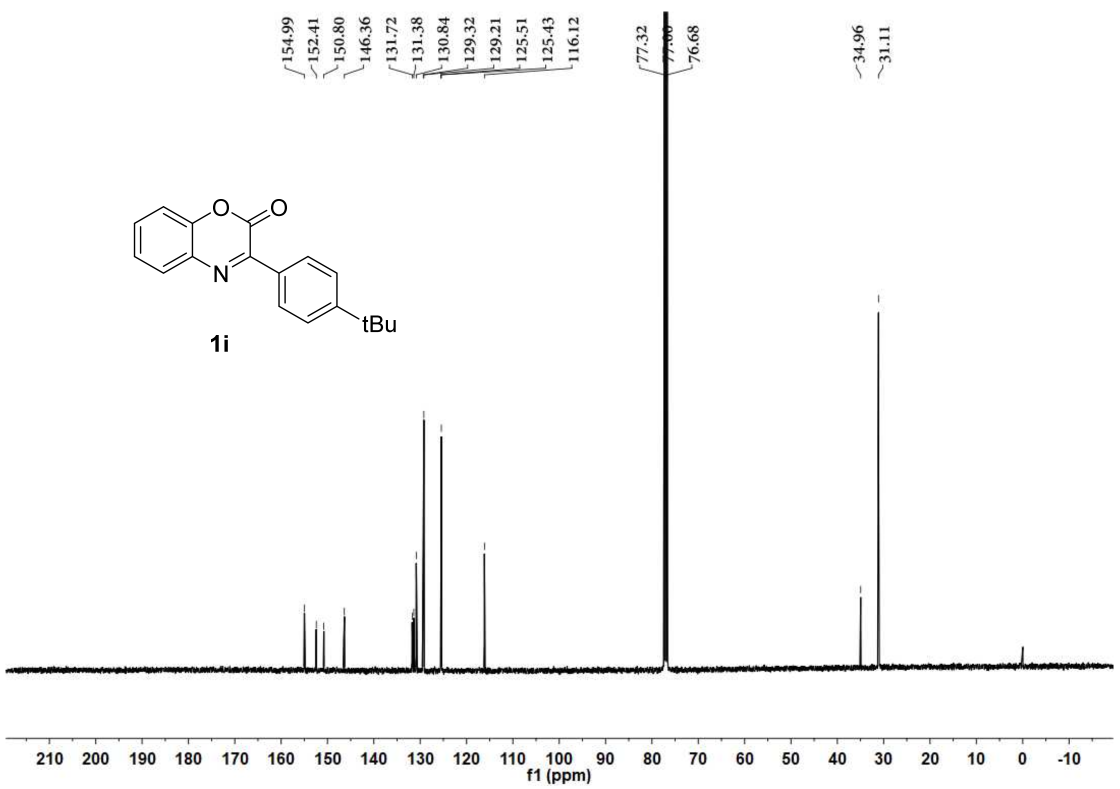
154.99 152.41 150.80 146.36 131.72 131.38 130.84 129.32 129.21 125.51 125.43 116.12
77.32 76.68
34.96 31.11
O
O
N
tBu
1i
210 200 190 180 170 160 150 140 130 120 110 100 90 80 70 60 50 40 30 20 10 0 -10
f1 (ppm)

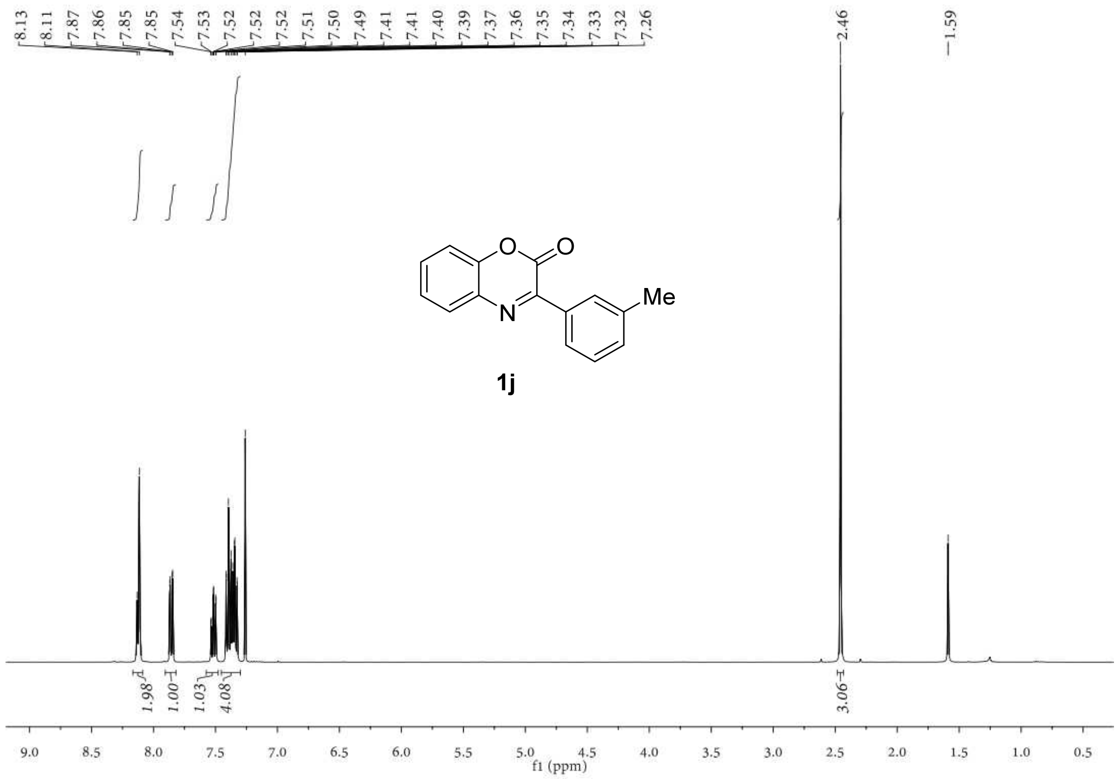
8.13
8.11
7.87
7.86
7.85
7.85
7.54
7.53
7.52
7.52
7.52
7.51
7.50
7.49
7.41
7.41
7.40
7.39
7.37
7.36
7.35
7.34
7.33
7.32
7.26
2.46
1.59
Me
N
O
O
1j
1.98
1.00
1.03
4.08
3.06
9.0 8.5 8.0 7.5 7.0 6.5 6.0 5.5 5.0 4.5 4.0 3.5 3.0 2.5 2.0 1.5 1.0 0.5
f1 (ppm)

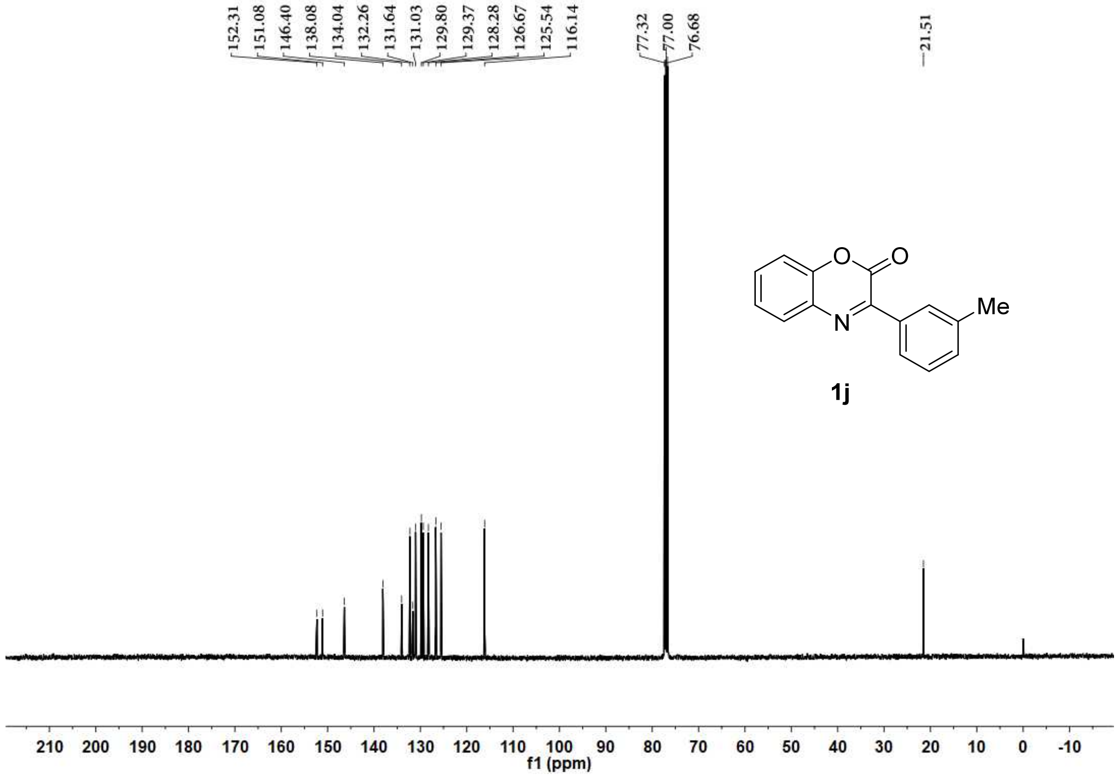
152.31
151.08
146.40
138.08
134.04
132.26
131.64
131.03
129.80
129.37
128.28
126.67
125.54
116.14
77.32
77.00
76.68
21.51
Me
N
O
O
1j
210 200 190 180 170 160 150 140 130 120 110 100 90 80 70 60 50 40 30 20 10 0 -10
f1 (ppm)

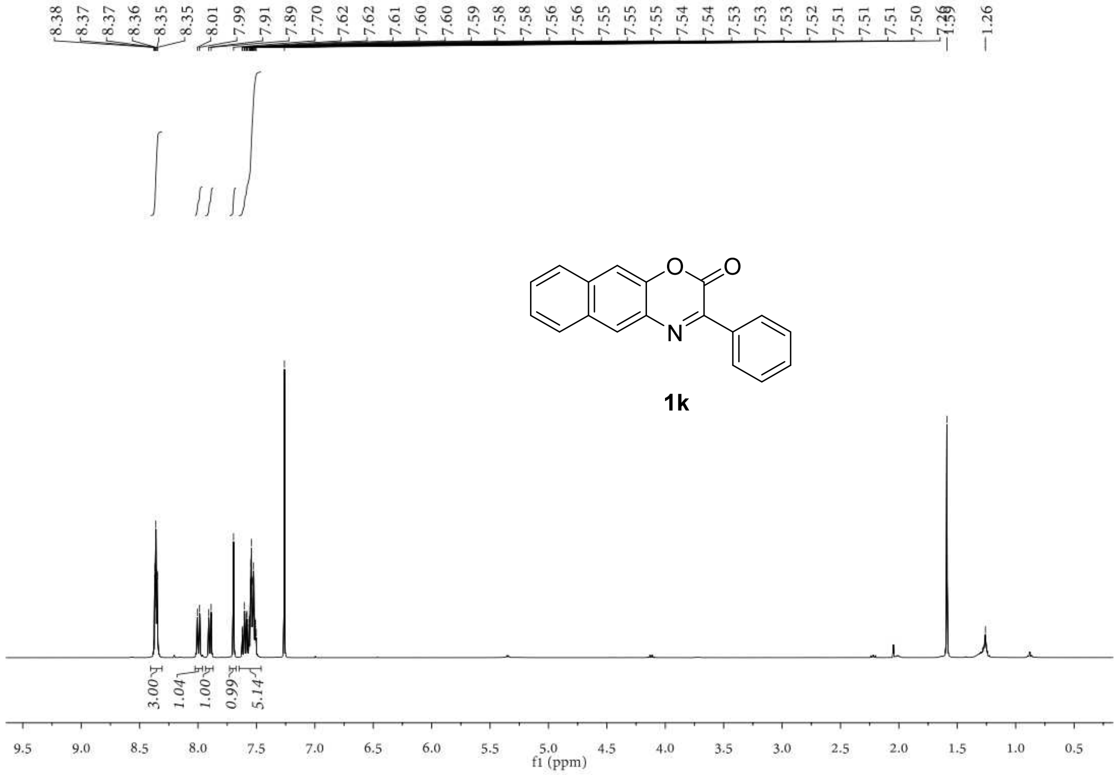
8.38 8.37 8.37 8.36 8.35 8.35 8.01 7.99 7.91 7.89 7.70 7.62 7.62 7.61 7.60 7.60 7.59 7.58 7.58 7.56 7.56 7.55 7.55 7.55 7.54 7.54 7.53 7.53 7.53 7.52 7.51 7.51 7.51 7.50 7.26 1.59 1.26
3.00 1.04 1.00 0.99 5.14
O
N
O
1k
9.5 9.0 8.5 8.0 7.5 7.0 6.5 6.0 5.5 5.0 4.5 4.0 3.5 3.0 2.5 2.0 1.5 1.0 0.5
f1 (ppm)

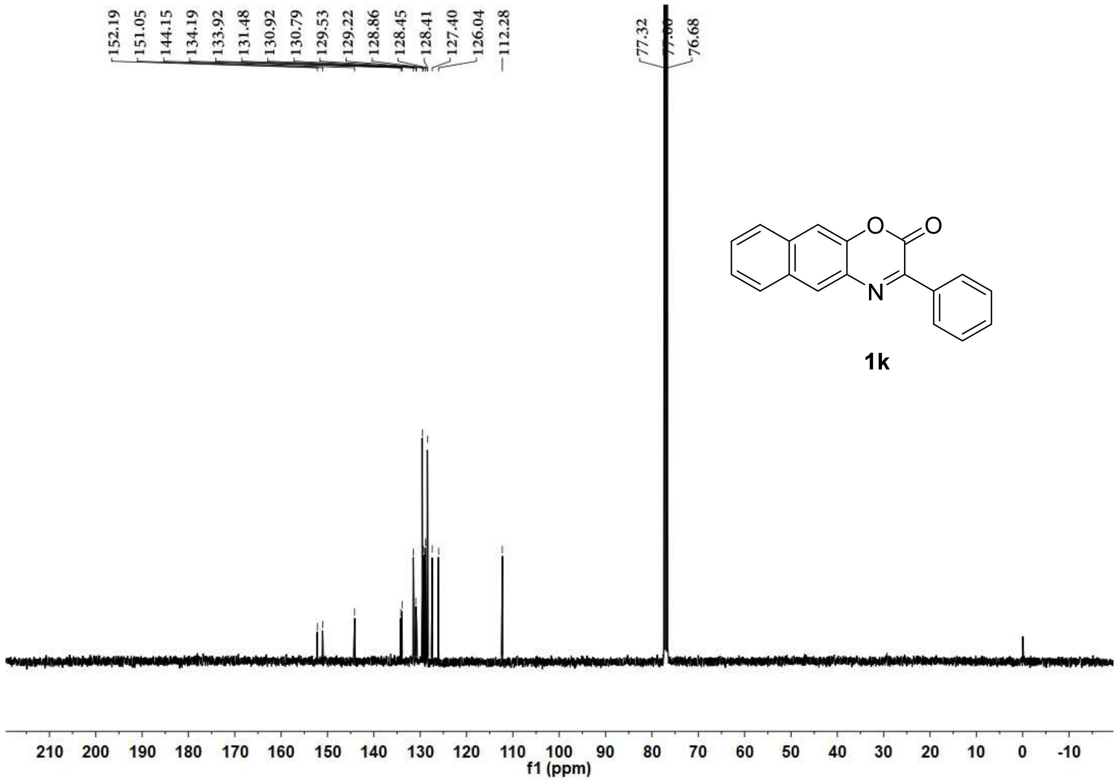
152.19 151.05 144.15 134.19 133.92 131.48 130.92 130.79 129.53 129.22 128.86 128.45 128.41 127.40 126.04 112.28
77.32 77.00 76.68
O
N
O
1k
210 200 190 180 170 160 150 140 130 120 110 100 90 80 70 60 50 40 30 20 10 0 -10
f1 (ppm)

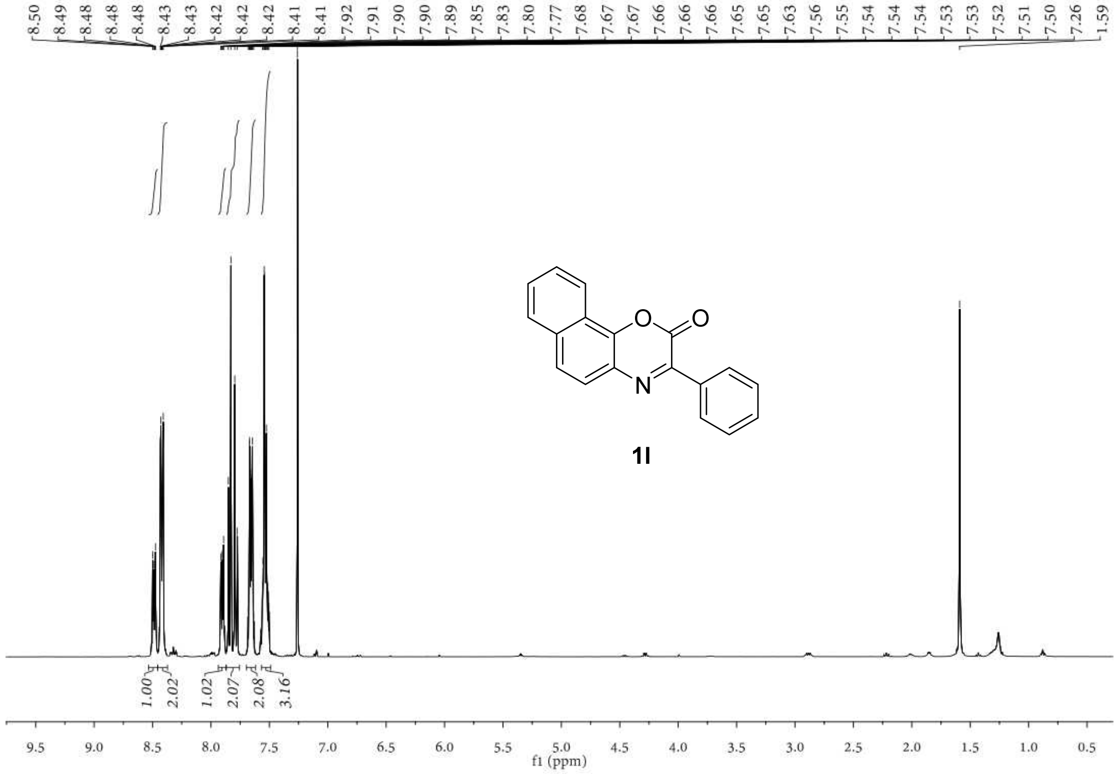
8.50 8.49 8.48 8.48 8.48 8.43 8.43 8.42 8.42 8.42 8.41 8.41 7.92 7.91 7.90 7.90 7.89 7.85 7.83 7.80 7.77 7.68 7.67 7.67 7.66 7.66 7.66 7.65 7.65 7.63 7.56 7.55 7.54 7.54 7.54 7.53 7.53 7.52 7.51 7.50 7.26 1.59
1l
1.00 2.02 1.02 2.07 2.08 3.16
9.5 9.0 8.5 8.0 7.5 7.0 6.5 6.0 5.5 5.0 4.5 4.0 3.5 3.0 2.5 2.0 1.5 1.0 0.5
f1 (ppm)

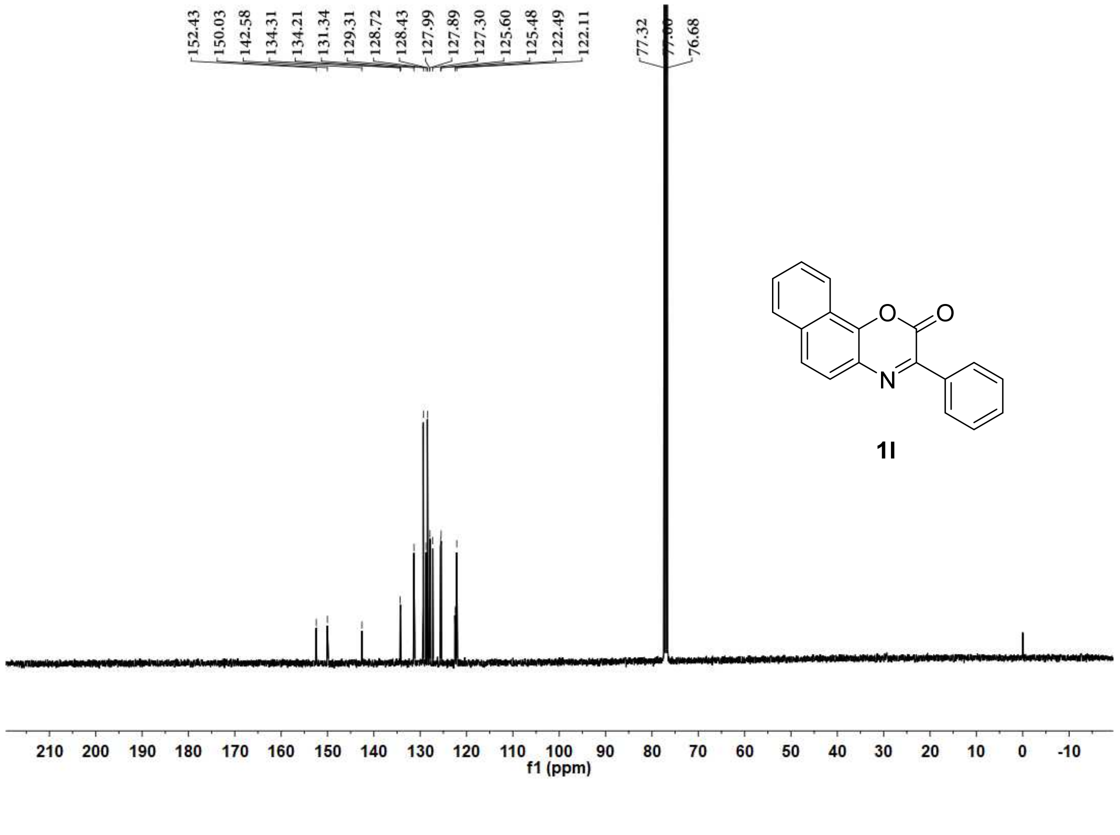
152.43 150.03 142.58 134.31 134.21 131.34 129.31 128.72 128.43 127.99 127.89 127.30 125.60 125.48 122.49 122.11
77.32 76.68
1l
210 200 190 180 170 160 150 140 130 120 110 100 90 80 70 60 50 40 30 20 10 0 -10
f1 (ppm)

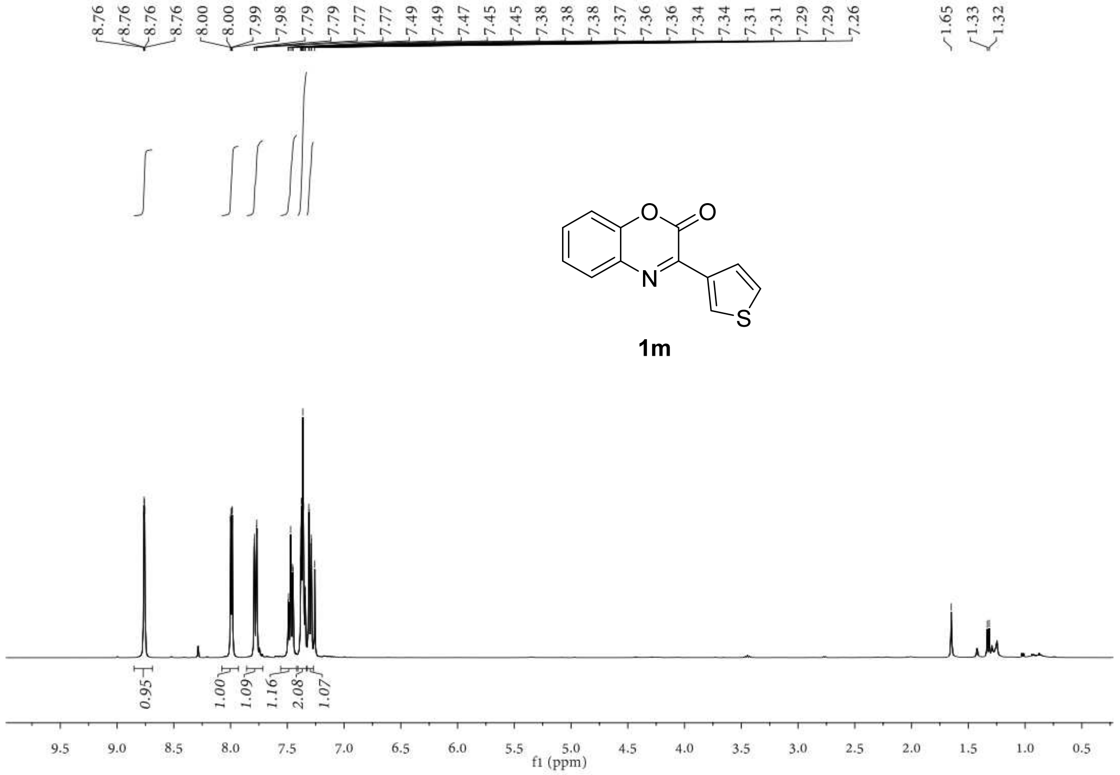

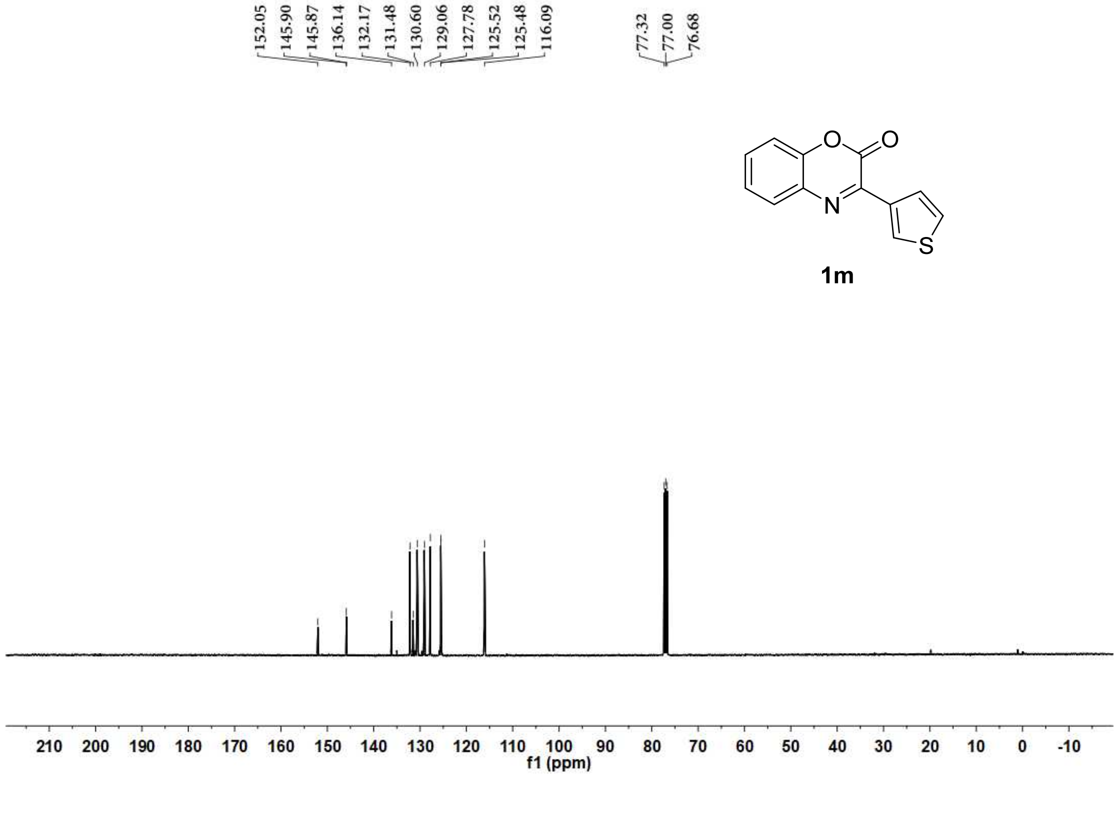

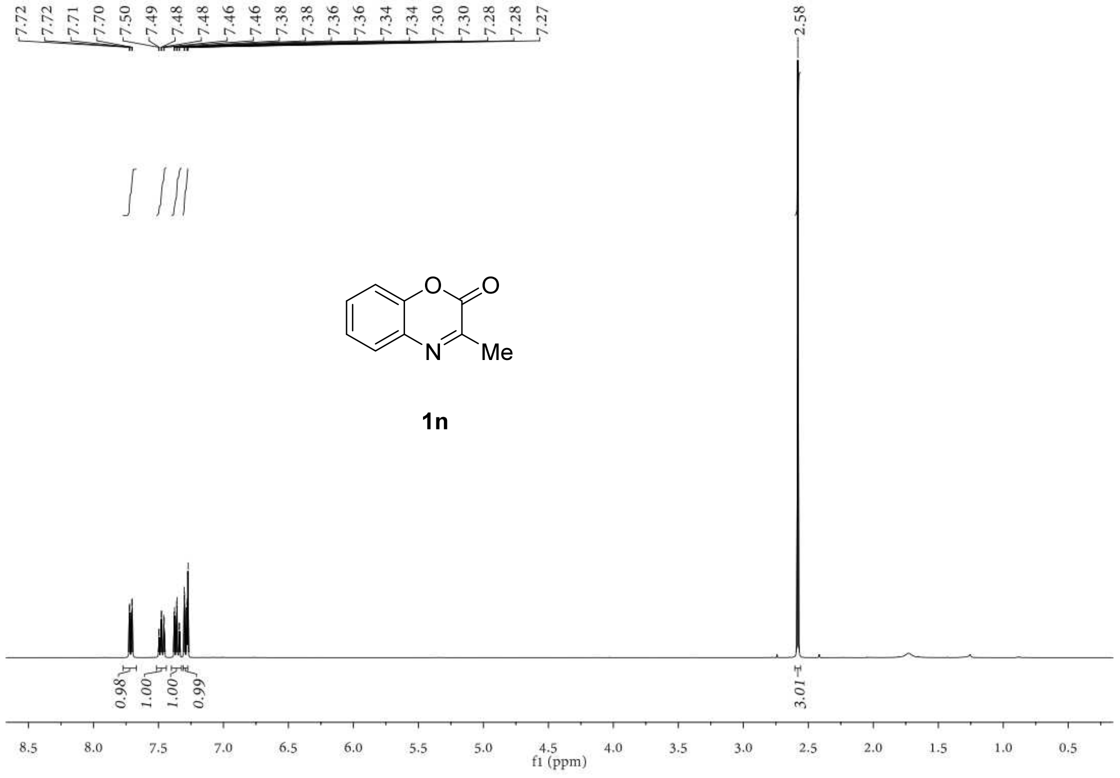
7.72 7.72 7.71 7.70 7.50 7.49 7.48 7.48 7.46 7.46 7.38 7.38 7.36 7.36 7.34 7.34 7.30 7.30 7.28 7.28 7.27
2.58
O
O
N
Me
1n
0.98
1.00
1.00
0.99
3.01
8.5 8.0 7.5 7.0 6.5 6.0 5.5 5.0 4.5 4.0 3.5 3.0 2.5 2.0 1.5 1.0 0.5
f1 (ppm)

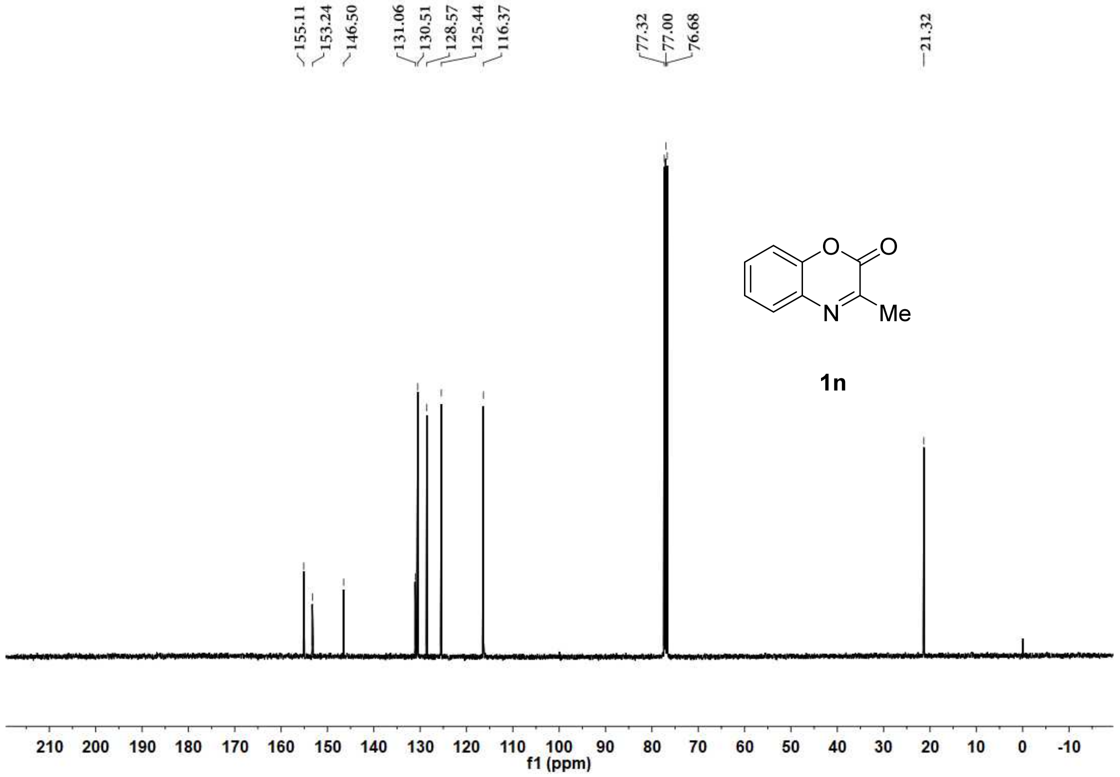
155.11 153.24 146.50
131.06 130.51 128.57 125.44 116.37
77.32 77.00 76.68
21.32
O
O
N
Me
1n
210 200 190 180 170 160 150 140 130 120 110 100 90 80 70 60 50 40 30 20 10 0 -10
f1 (ppm)

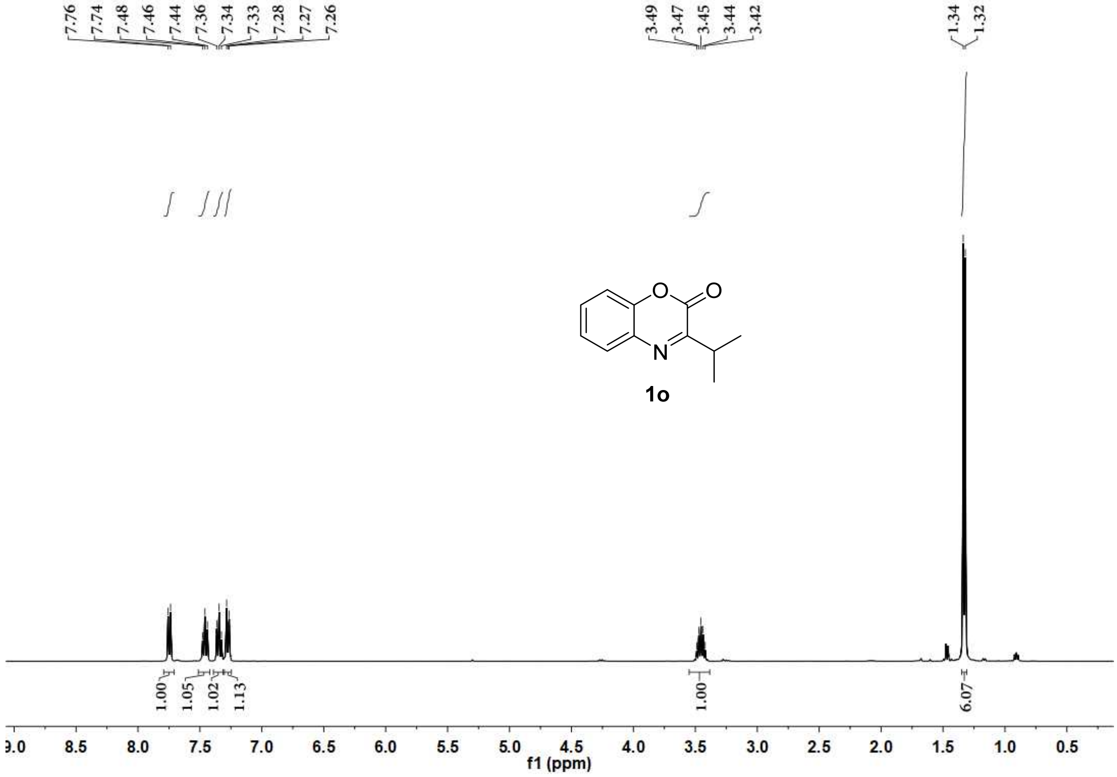
7.76
7.74
7.48
7.46
7.44
7.36
7.34
7.33
7.28
7.27
7.26
3.49
3.47
3.45
3.44
3.42
1.34
1.32
1o
1.00
1.05
1.02
1.13
1.00
6.07
f1 (ppm)

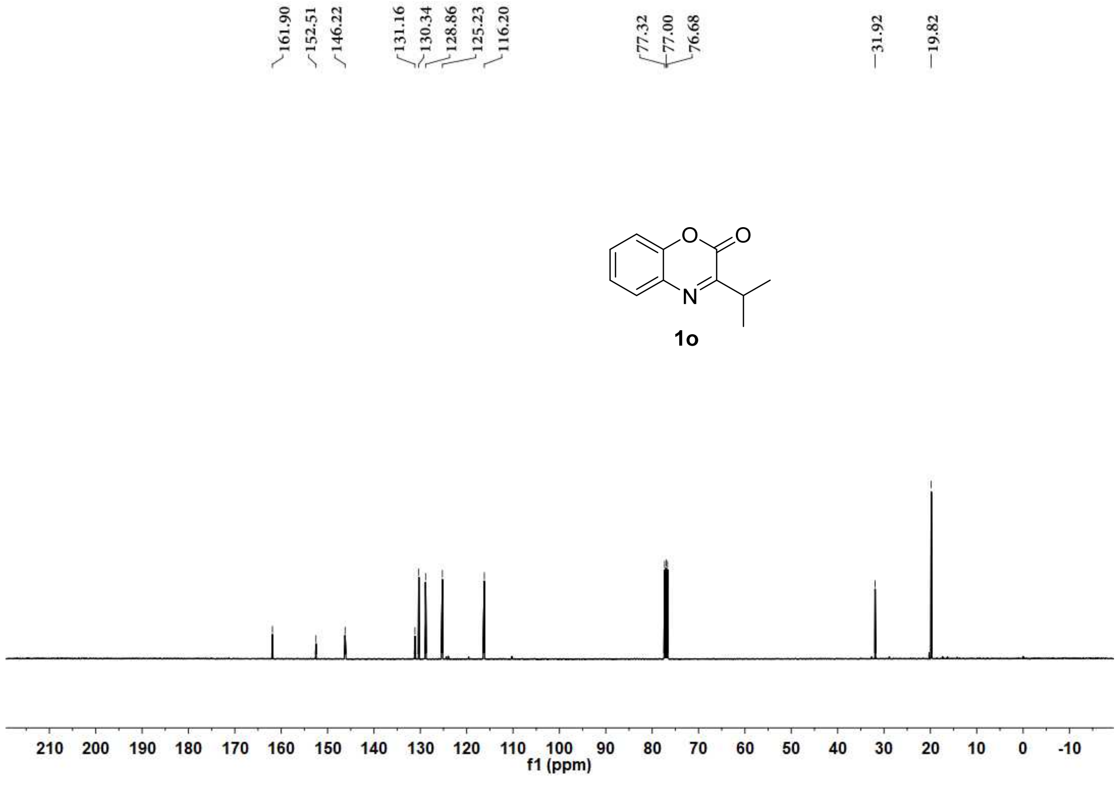
161.90
152.51
146.22
131.16
130.34
128.86
125.23
116.20
77.32
77.00
76.68
31.92
19.82
1o
f1 (ppm)

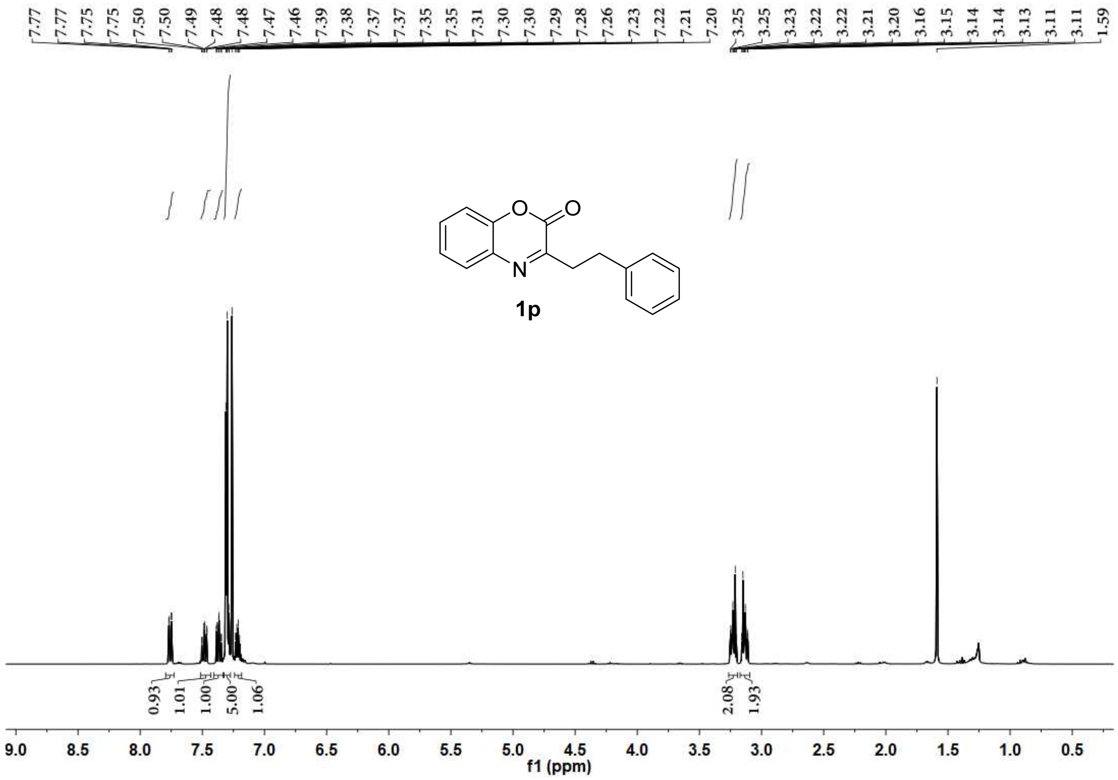
7.77 7.77 7.75 7.75 7.50 7.50 7.49 7.48 7.48 7.47 7.46 7.39 7.38 7.37 7.37 7.35 7.35 7.31 7.30 7.30 7.29 7.28 7.26 7.23 7.22 7.21 7.20 3.25 3.25 3.23 3.22 3.22 3.21 3.20 3.16 3.15 3.14 3.14 3.13 3.11 3.11 1.59
O
O
N
1p
0.93
1.01
1.00
5.00
1.06
2.08
1.93
9.0 8.5 8.0 7.5 7.0 6.5 6.0 5.5 5.0 4.5 4.0 3.5 3.0 2.5 2.0 1.5 1.0 0.5
f1 (ppm)

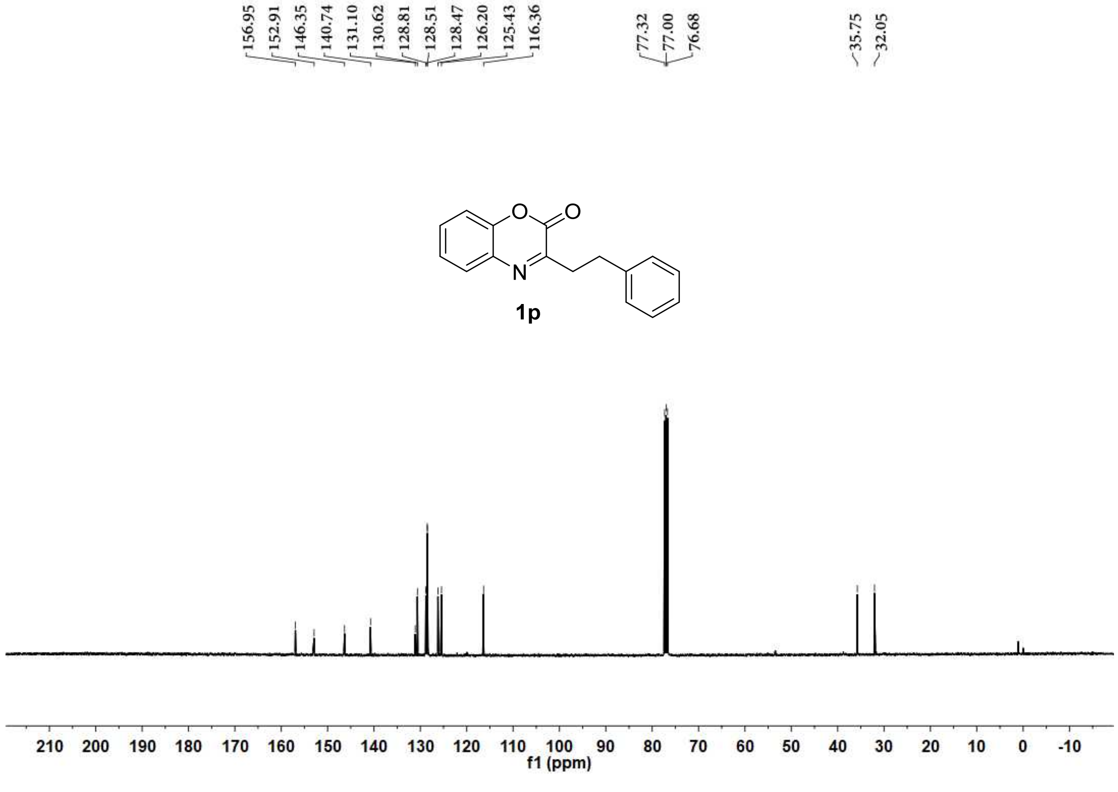
156.95 152.91 146.35 140.74 131.10 130.62 128.81 128.51 128.47 126.20 125.43 116.36
77.32 77.00 76.68
35.75 32.05
O
O
N
1p
210 200 190 180 170 160 150 140 130 120 110 100 90 80 70 60 50 40 30 20 10 0 -10
f1 (ppm)

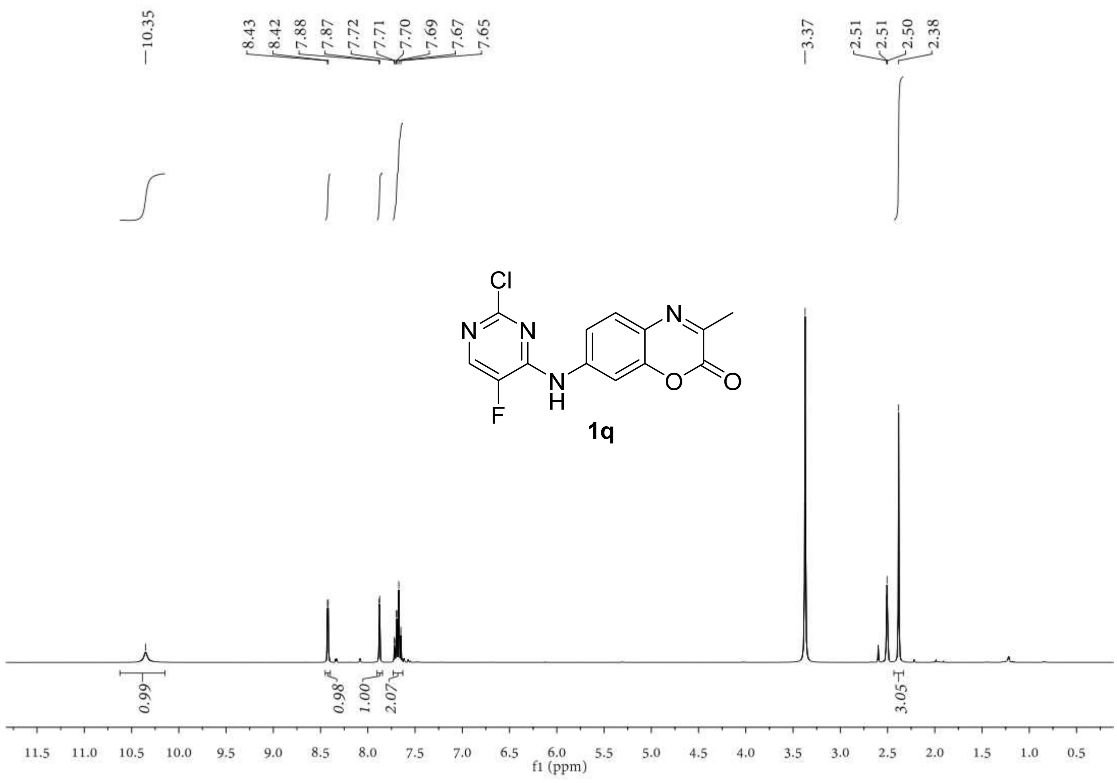

10.35
8.43
8.42
7.88
7.87
7.72
7.71
7.70
7.69
7.67
7.65
3.37
2.51
2.51
2.50
2.38
Cl
N
N
N
N
H
O
O
F
1q
0.99
0.98
1.00
2.07
3.05
11.5 11.0 10.5 10.0 9.5 9.0 8.5 8.0 7.5 7.0 6.5 6.0 5.5 5.0 4.5 4.0 3.5 3.0 2.5 2.0 1.5 1.0 0.5
f1 (ppm)

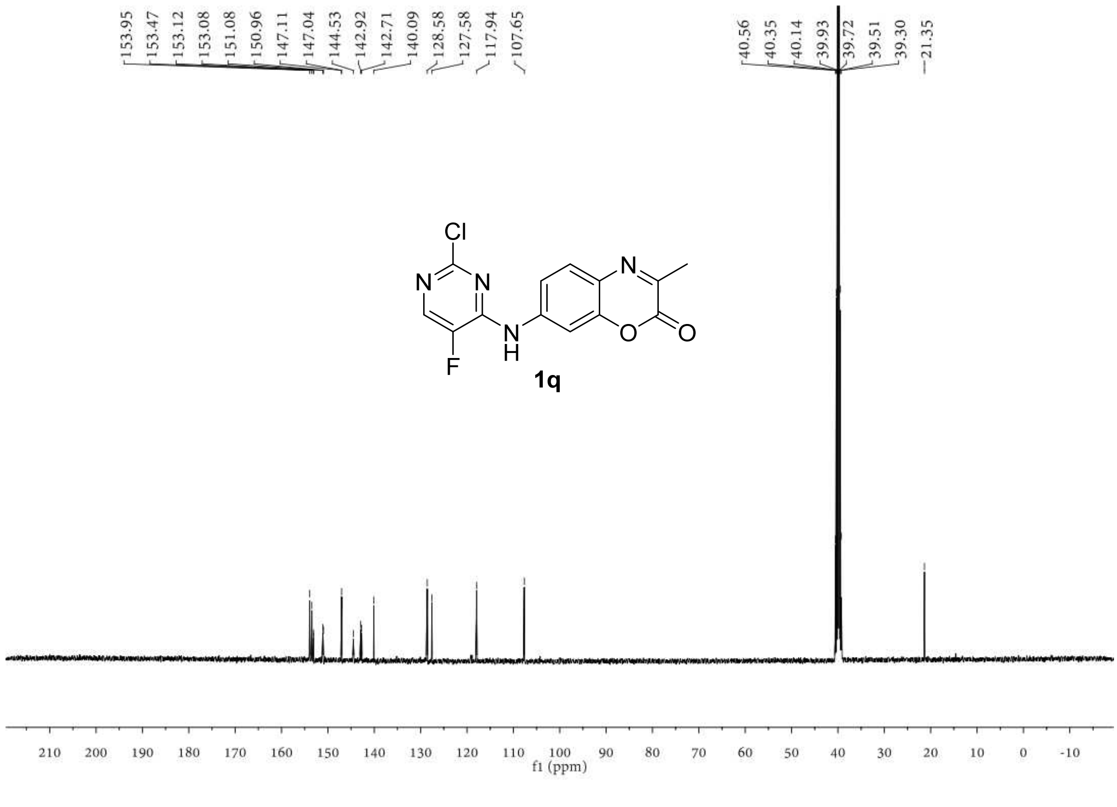

153.95
153.47
153.12
153.08
151.08
150.96
147.11
147.04
144.53
142.92
142.71
140.09
128.58
127.58
117.94
107.65
40.56
40.35
40.14
39.93
39.72
39.51
39.30
21.35
Cl
N
N
N
N
H
O
O
F
1q
210 200 190 180 170 160 150 140 130 120 110 100 90 80 70 60 50 40 30 20 10 0 -10
f1 (ppm)

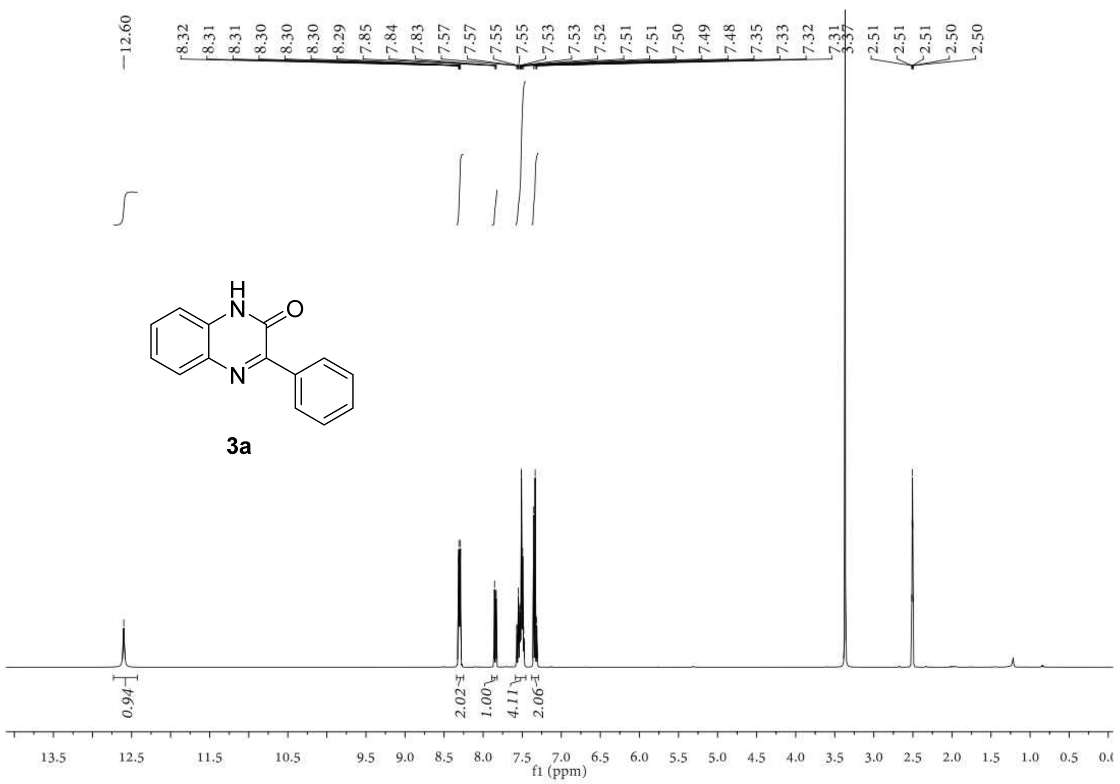
12.60
8.32
8.31
8.31
8.30
8.30
8.30
8.29
7.85
7.84
7.83
7.57
7.57
7.55
7.55
7.53
7.53
7.52
7.51
7.51
7.50
7.49
7.48
7.35
7.33
7.32
7.31
3.37
2.51
2.51
2.51
2.50
2.50
3a
0.94
2.02
1.00
4.11
2.06
f1 (ppm)

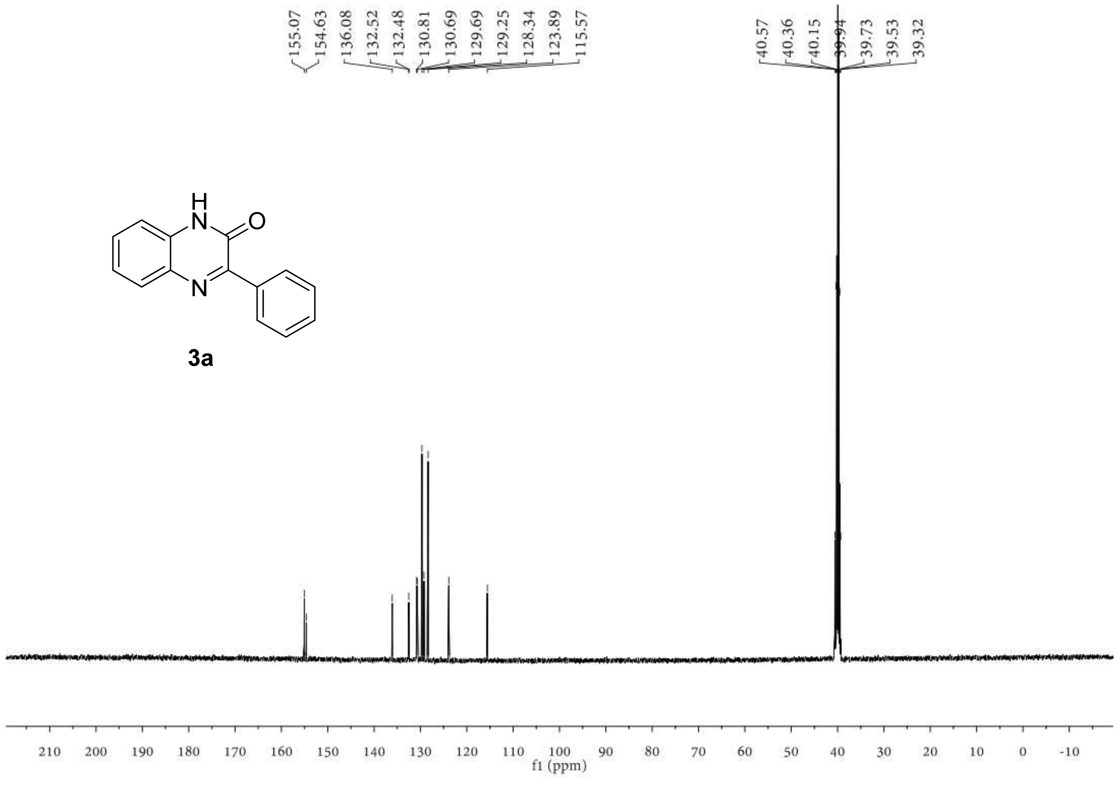
155.07
154.63
136.08
132.52
132.48
130.81
130.69
129.69
129.25
128.34
123.89
115.57
40.57
40.36
40.15
39.94
39.73
39.53
39.32
3a
f1 (ppm)

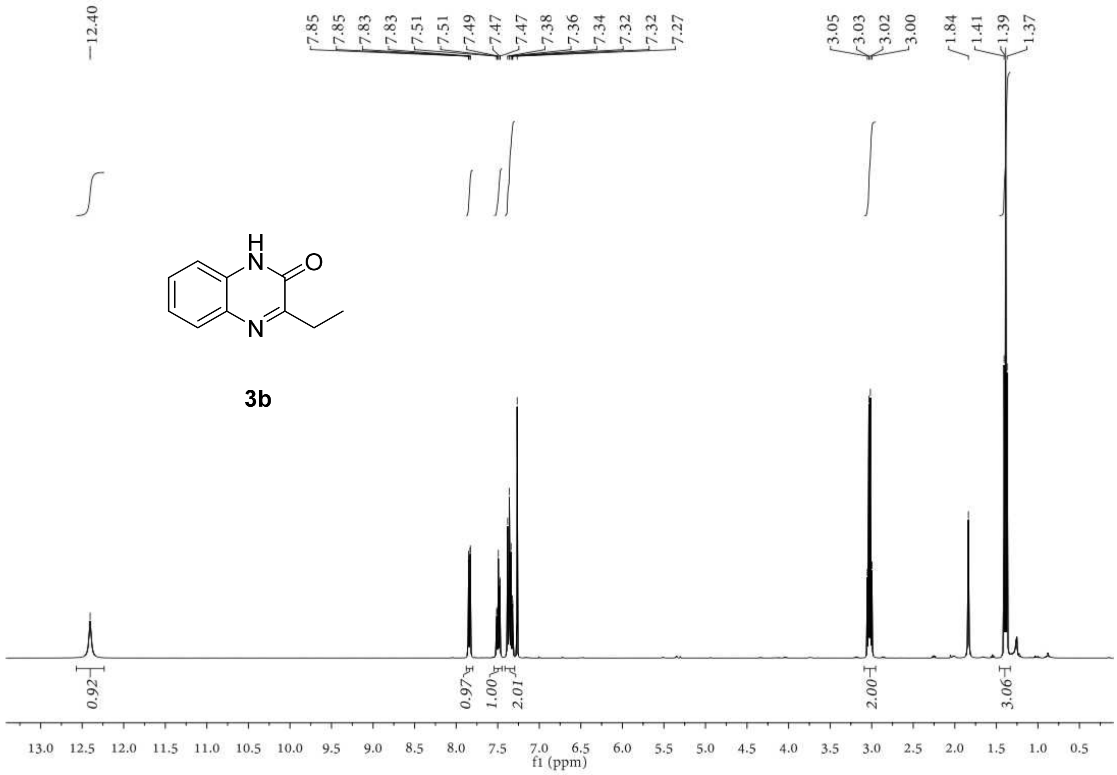
12.40
7.85 7.85 7.83 7.83 7.51 7.51 7.49 7.47 7.47 7.38 7.36 7.34 7.32 7.32 7.27
3.05 3.03 3.02 3.00 1.84 1.41 1.39 1.37
H
N
O
N
3b
0.92
0.97
1.00
2.01
2.00
3.06
13.0 12.5 12.0 11.5 11.0 10.5 10.0 9.5 9.0 8.5 8.0 7.5 7.0 6.5 6.0 5.5 5.0 4.5 4.0 3.5 3.0 2.5 2.0 1.5 1.0 0.5
f1 (ppm)

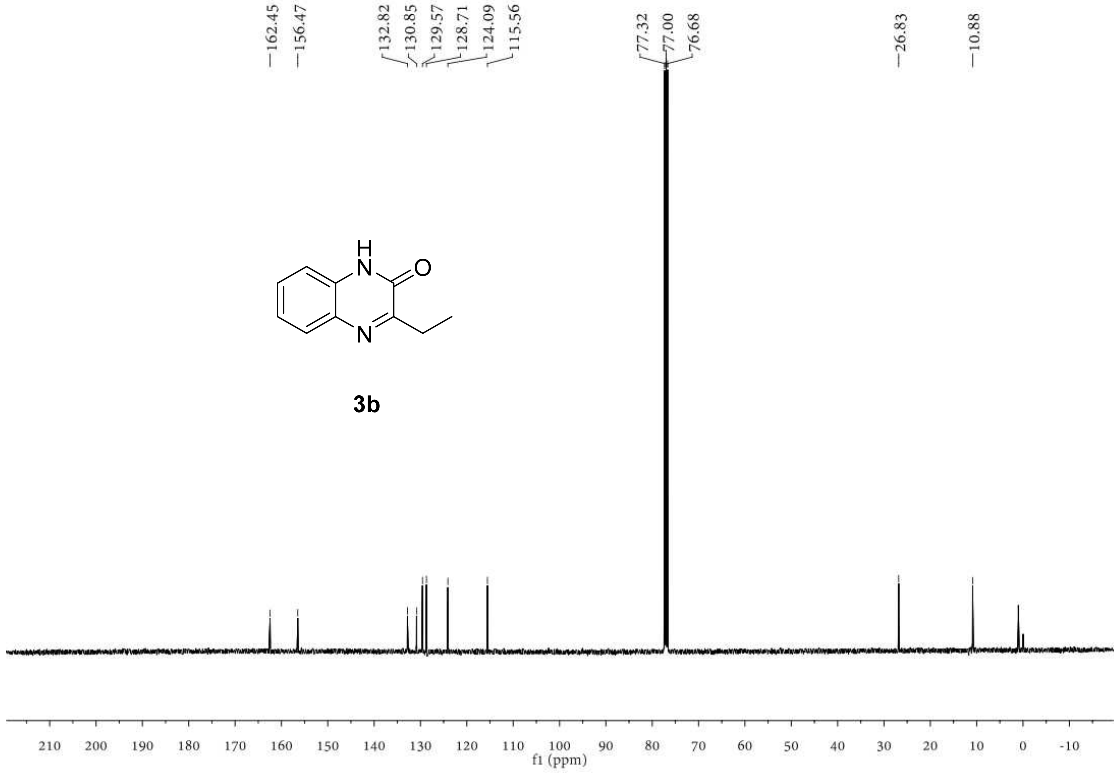
162.45
156.47
132.82 130.85 129.57 128.71 124.09 115.56
77.32 77.00 76.68
26.83
10.88
H
N
O
N
3b
210 200 190 180 170 160 150 140 130 120 110 100 90 80 70 60 50 40 30 20 10 0 -10
f1 (ppm)

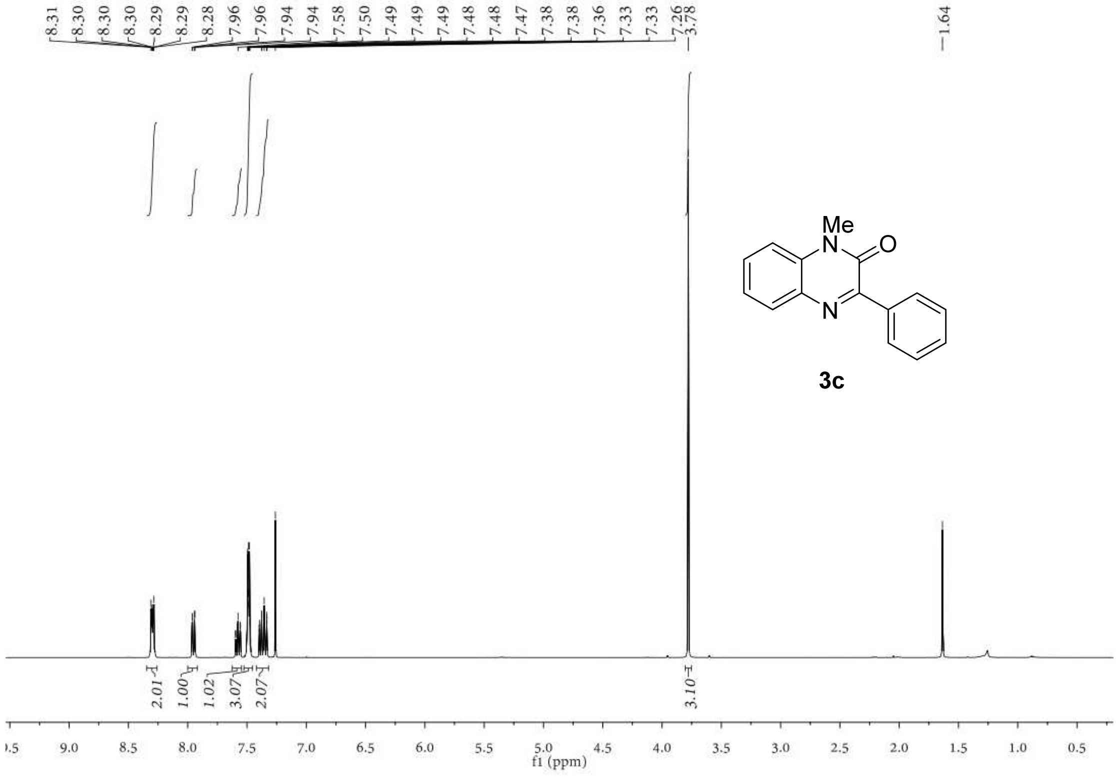
8.31
8.30
8.30
8.30
8.29
8.29
8.28
7.96
7.96
7.94
7.94
7.58
7.50
7.49
7.49
7.49
7.48
7.48
7.47
7.38
7.38
7.36
7.33
7.33
7.26
3.78
1.64
Me
N
O
N
3c
2.01
1.00
1.02
3.07
2.07
3.10
f1 (ppm)

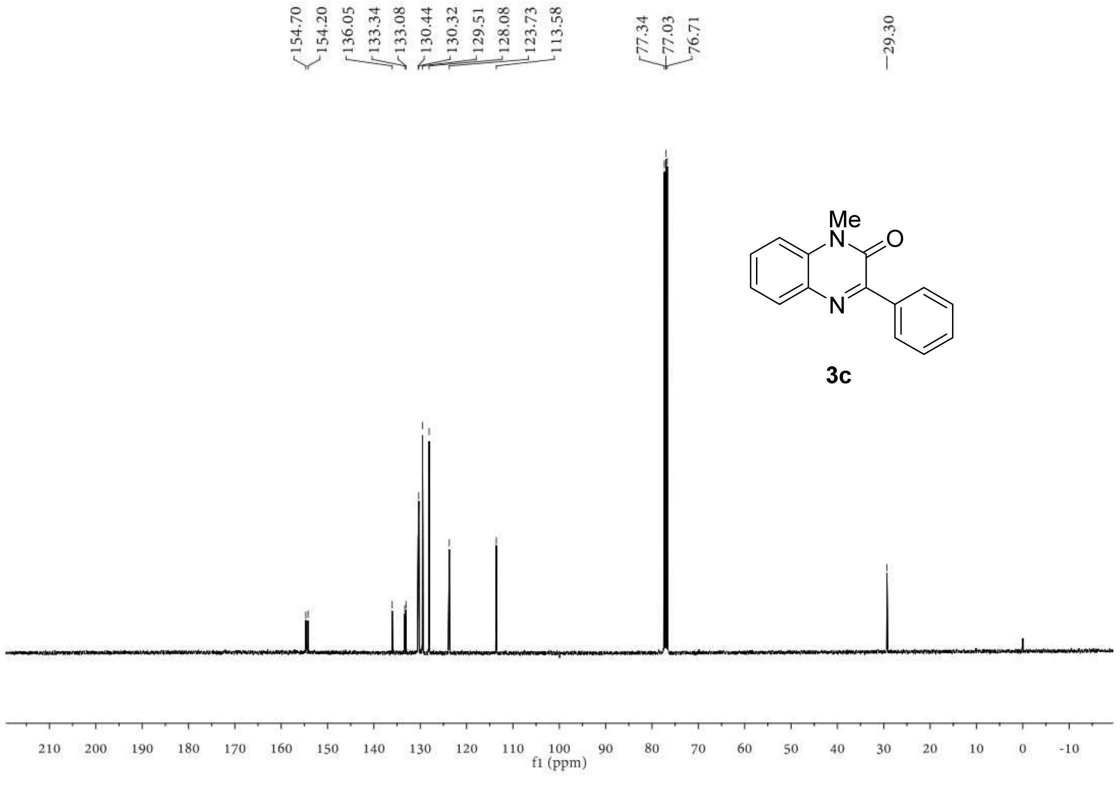
154.70
154.20
136.05
133.34
133.08
130.44
130.32
129.51
128.08
123.73
113.58
77.34
77.03
76.71
29.30
Me
N
O
N
3c
f1 (ppm)

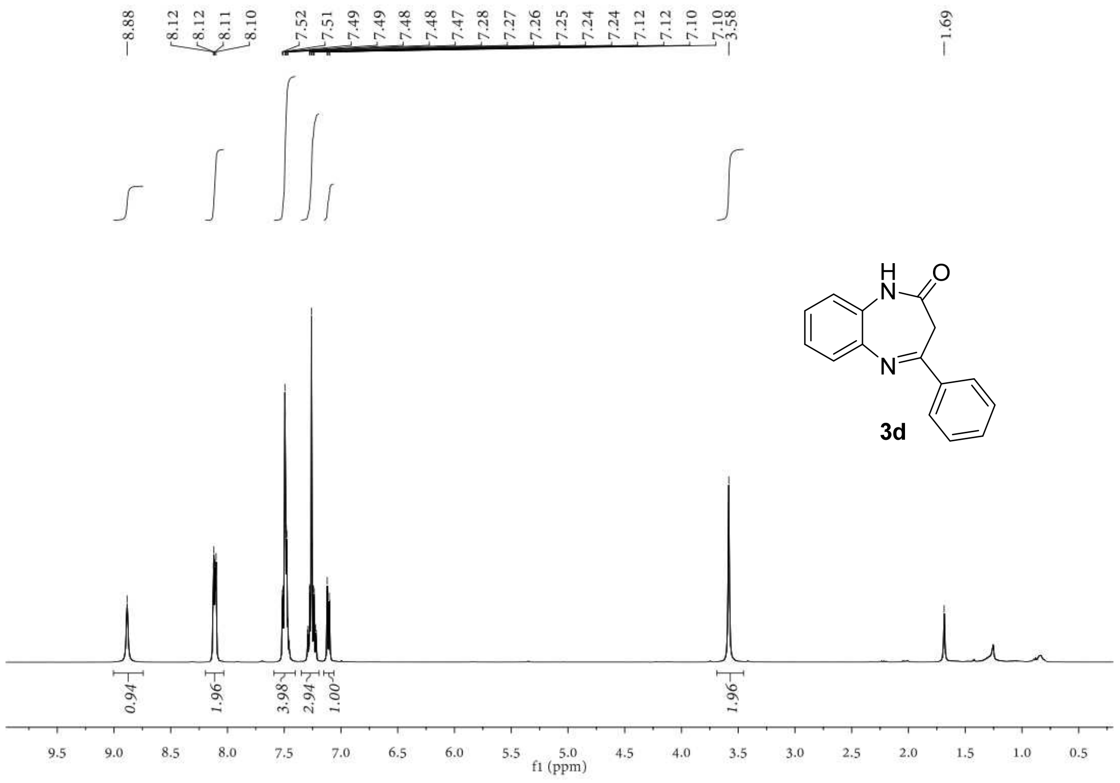

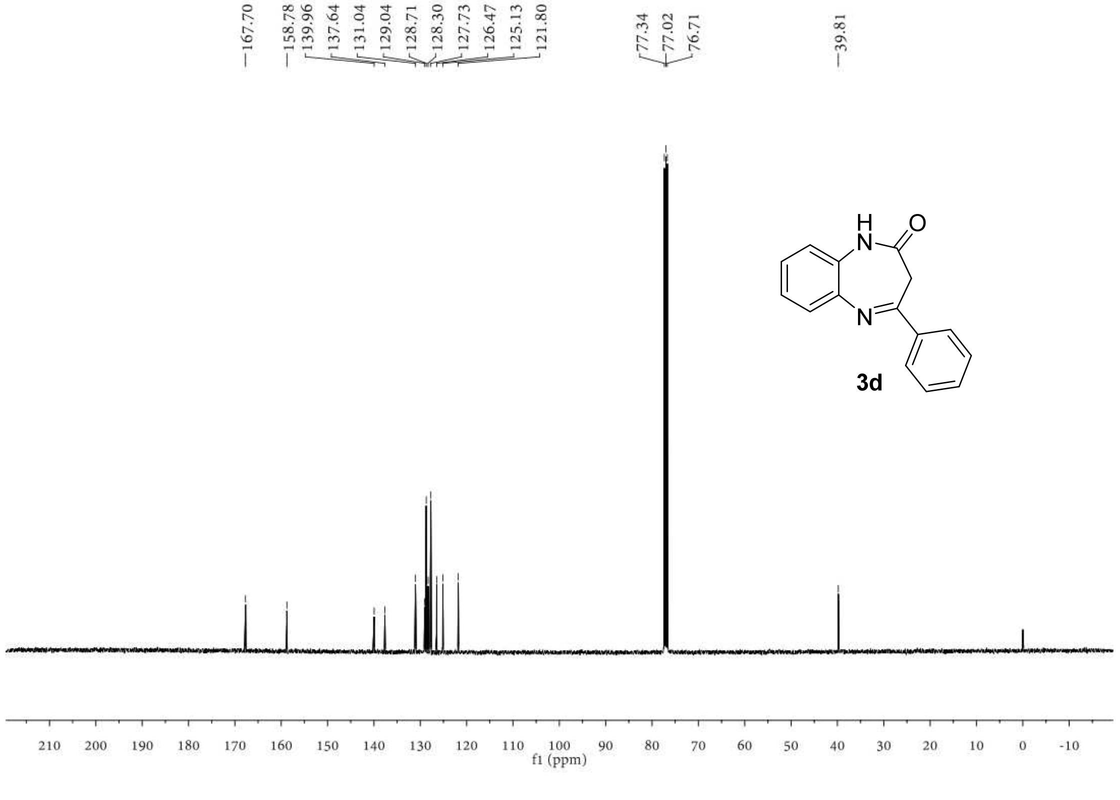

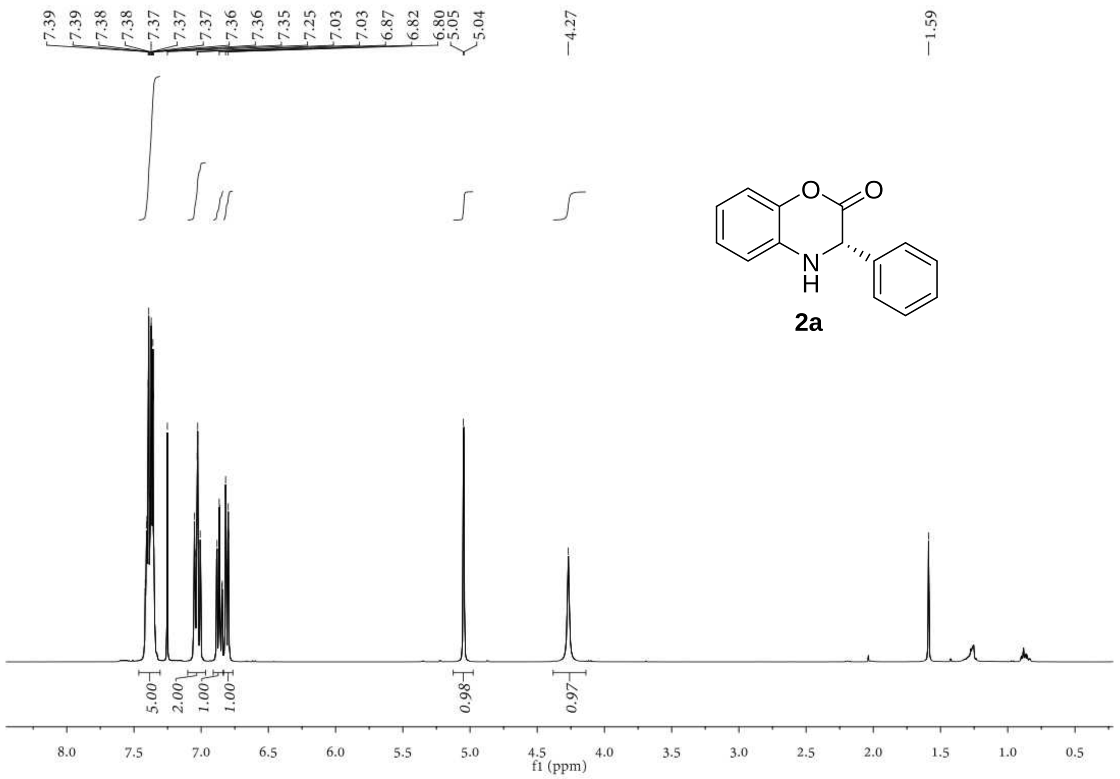
7.39
7.39
7.38
7.38
7.37
7.37
7.37
7.36
7.36
7.35
7.25
7.03
7.03
6.87
6.82
6.80
5.05
5.04
4.27
1.59
2a
5.00
2.00
1.00
1.00
0.98
0.97
f1 (ppm)

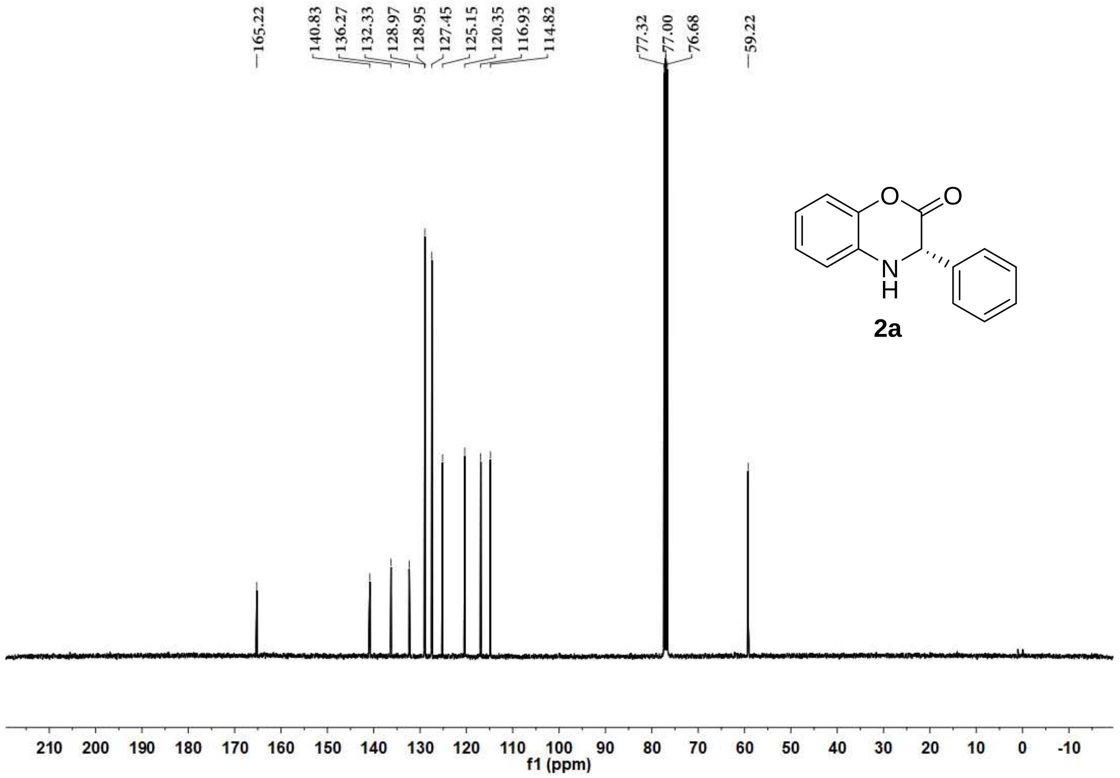
165.22
140.83
136.27
132.33
128.97
128.95
127.45
125.15
120.35
116.93
114.82
77.32
77.00
76.68
59.22
2a
f1 (ppm)

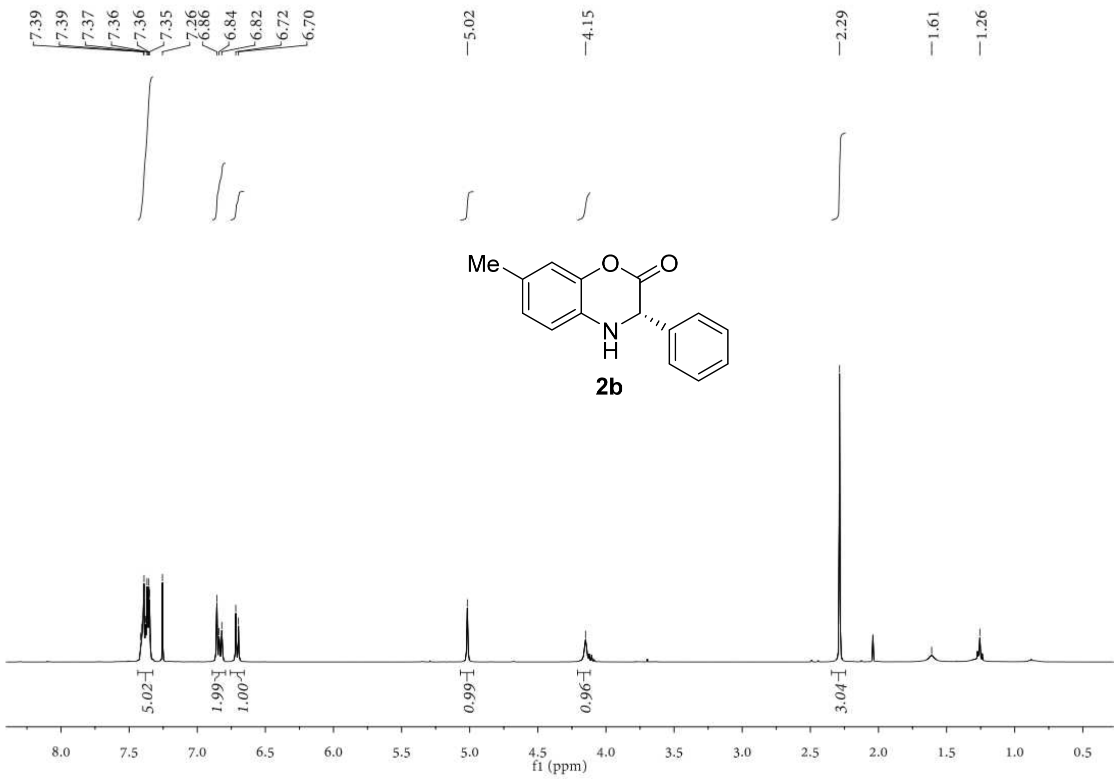
7.39
7.39
7.37
7.36
7.36
7.35
7.26
6.86
6.84
6.82
6.72
6.70
5.02
4.15
2.29
1.61
1.26
Me
O
O
N
H
2b
5.02
1.99
1.00
0.99
0.96
3.04
8.0 7.5 7.0 6.5 6.0 5.5 5.0 4.5 4.0 3.5 3.0 2.5 2.0 1.5 1.0 0.5
f1 (ppm)

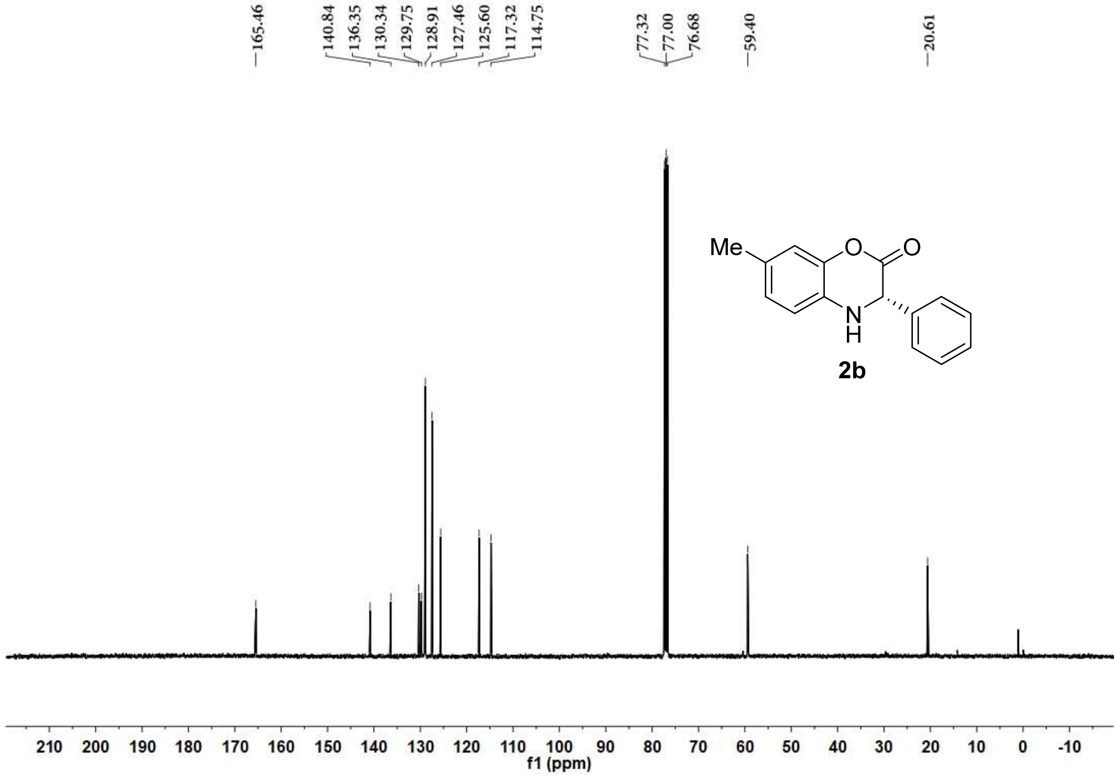
165.46
140.84
136.35
130.34
129.75
128.91
127.46
125.60
117.32
114.75
77.32
77.00
76.68
59.40
20.61
Me
O
O
N
H
2b
210 200 190 180 170 160 150 140 130 120 110 100 90 80 70 60 50 40 30 20 10 0 -10
f1 (ppm)

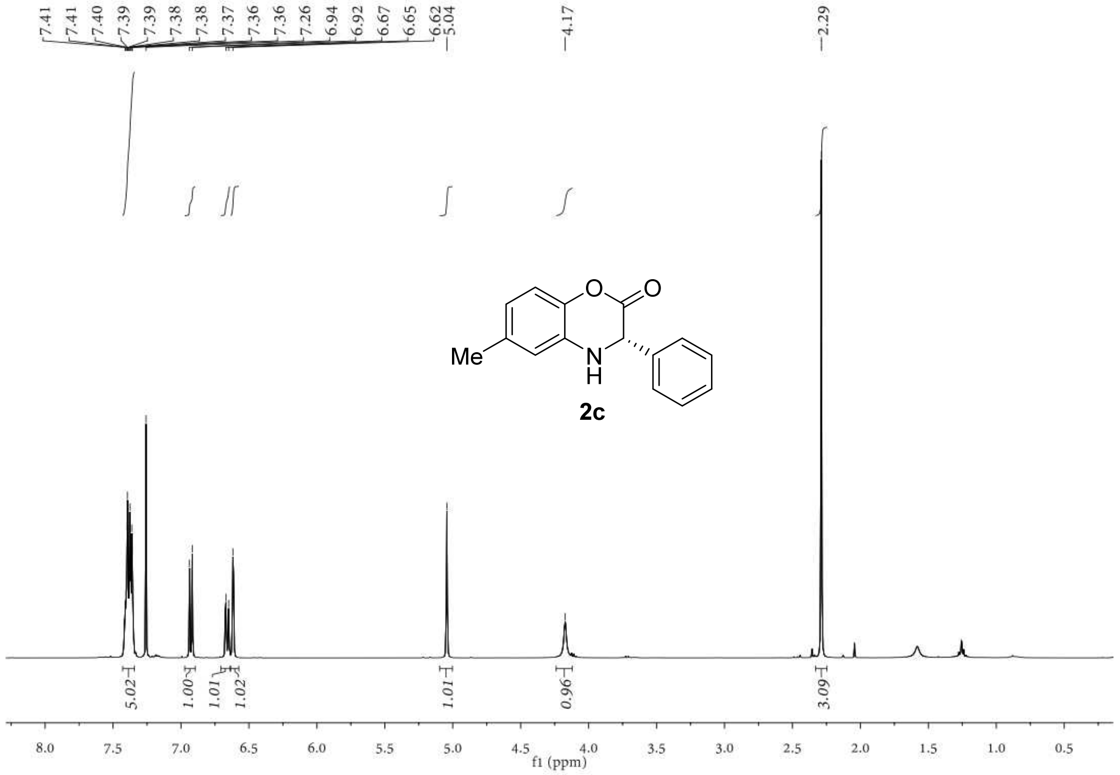
7.41
7.41
7.40
7.39
7.39
7.38
7.38
7.37
7.36
7.36
7.26
6.94
6.92
6.67
6.65
6.62
5.04
4.17
2.29
Me
O
O
N
H
2c
5.02
1.00
1.01
1.02
1.01
0.96
3.09
8.0 7.5 7.0 6.5 6.0 5.5 5.0 4.5 4.0 3.5 3.0 2.5 2.0 1.5 1.0 0.5
f1 (ppm)

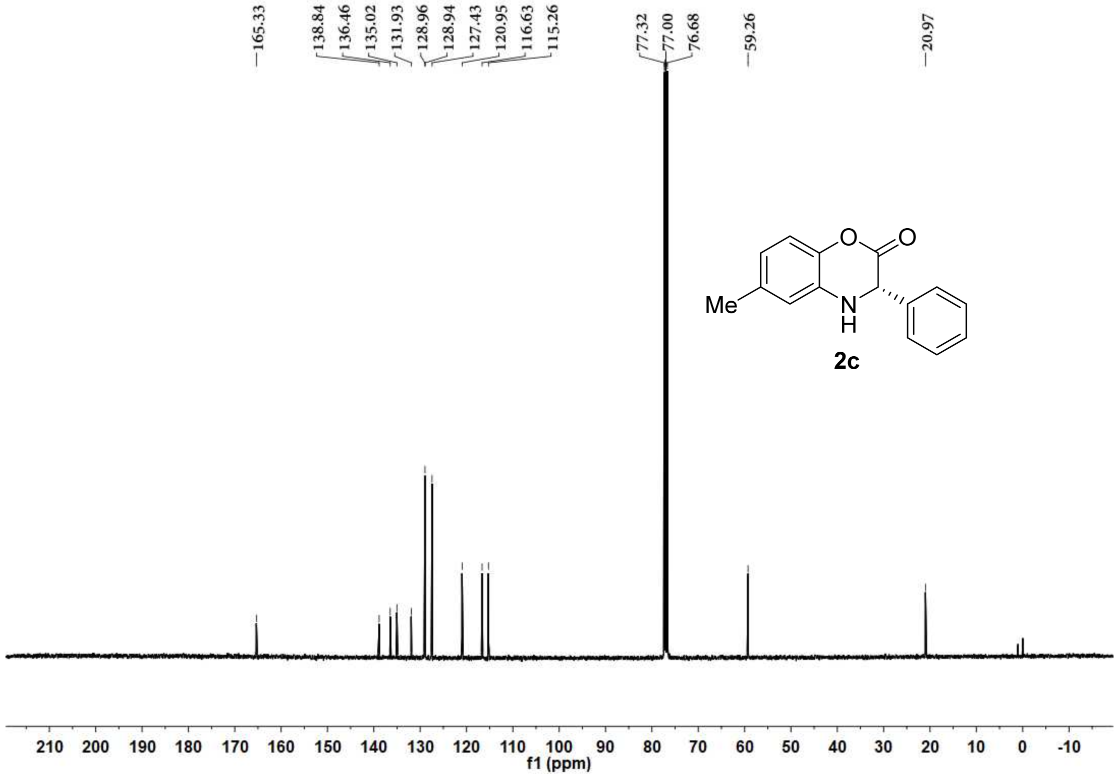
165.33
138.84
136.46
135.02
131.93
128.96
128.94
127.43
120.95
116.63
115.26
77.32
77.00
76.68
59.26
20.97
Me
O
O
N
H
2c
210 200 190 180 170 160 150 140 130 120 110 100 90 80 70 60 50 40 30 20 10 0 -10
f1 (ppm)

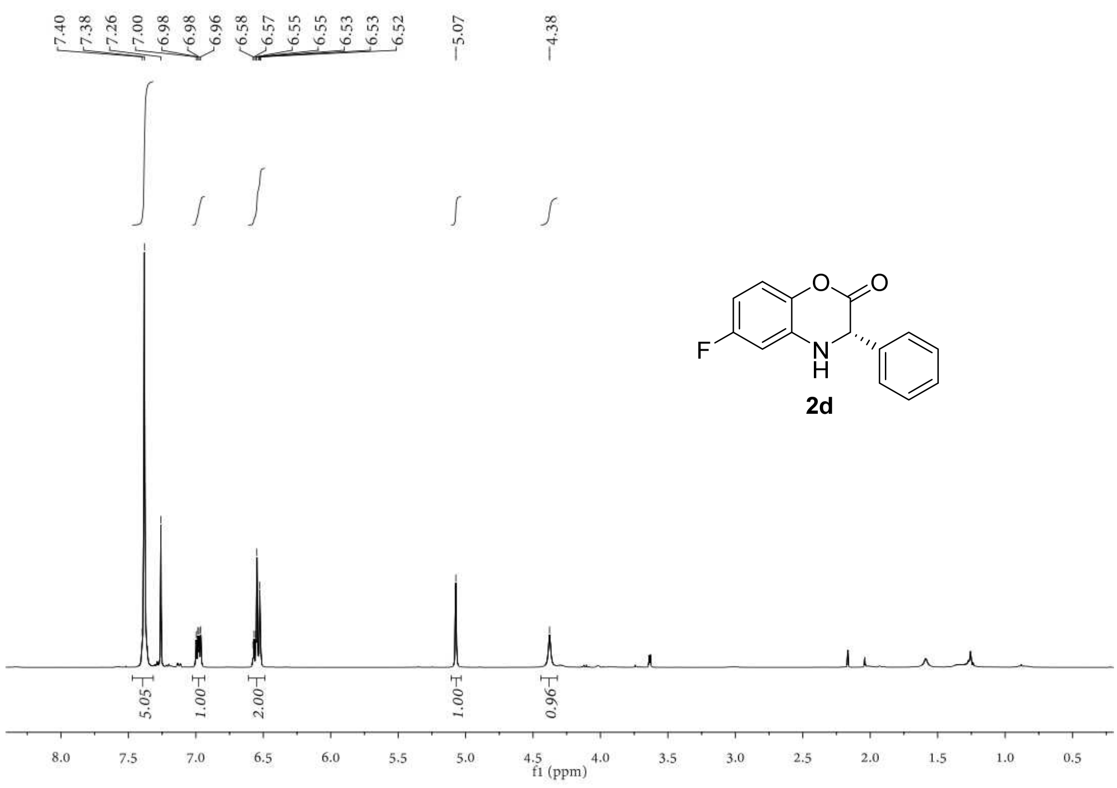
7.40
7.38
7.26
7.00
6.98
6.98
6.96
6.58
6.57
6.55
6.55
6.53
6.53
6.52
5.07
4.38
5.05
1.00
2.00
1.00
0.96
8.0 7.5 7.0 6.5 6.0 5.5 5.0 4.5 4.0 3.5 3.0 2.5 2.0 1.5 1.0 0.5
f1 (ppm)
F
O
O
N
H
2d

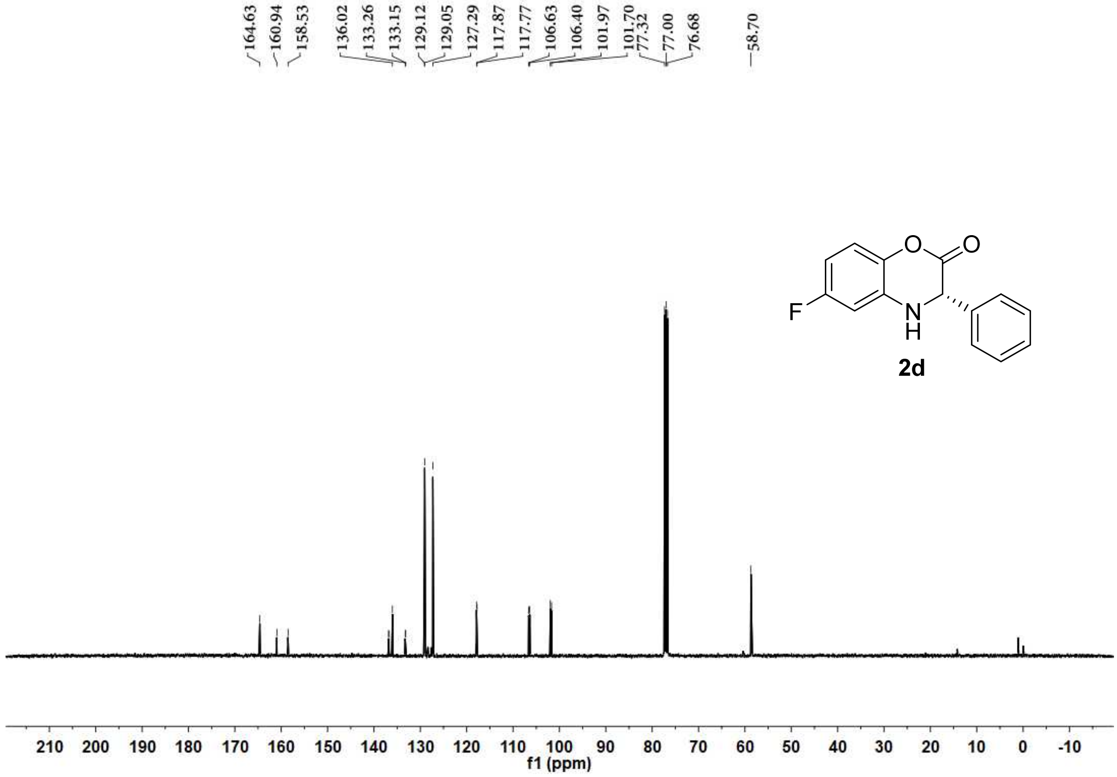
164.63
160.94
158.53
136.02
133.26
133.15
129.12
129.05
127.29
117.87
117.77
106.63
106.40
101.97
101.70
77.32
77.00
76.68
58.70
210 200 190 180 170 160 150 140 130 120 110 100 90 80 70 60 50 40 30 20 10 0 -10
f1 (ppm)
F
O
O
N
H
2d

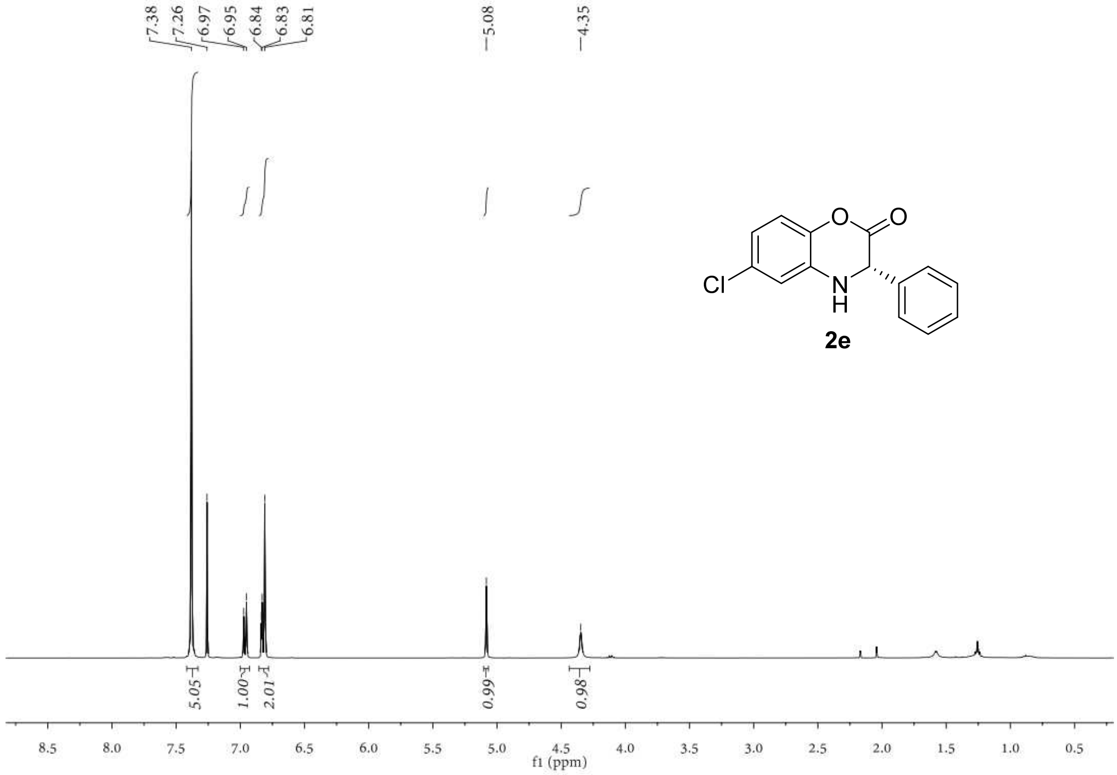
7.38
7.26
6.97
6.95
6.84
6.83
6.81
5.08
4.35
Cl
O
O
N
H
2e
5.05
1.00
2.01
0.99
0.98
8.5 8.0 7.5 7.0 6.5 6.0 5.5 5.0 4.5 4.0 3.5 3.0 2.5 2.0 1.5 1.0 0.5
f1 (ppm)

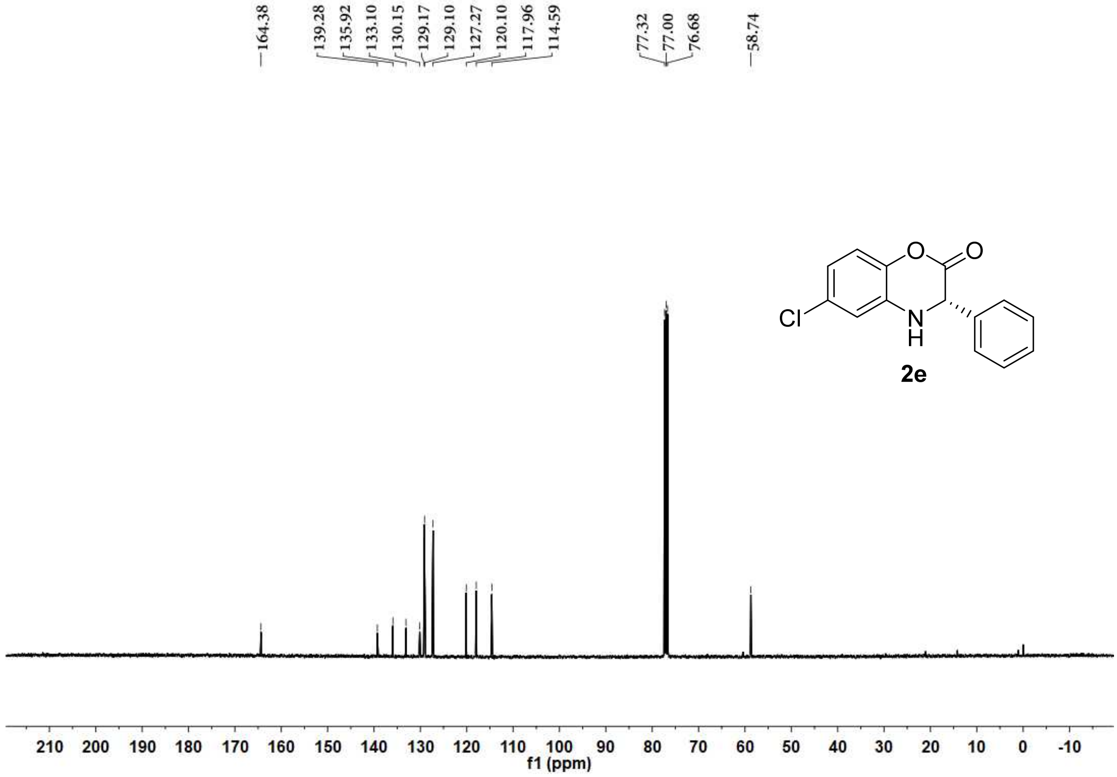
164.38
139.28
135.92
133.10
130.15
129.17
129.10
127.27
120.10
117.96
114.59
77.32
77.00
76.68
58.74
Cl
O
O
N
H
2e
210 200 190 180 170 160 150 140 130 120 110 100 90 80 70 60 50 40 30 20 10 0 -10
f1 (ppm)

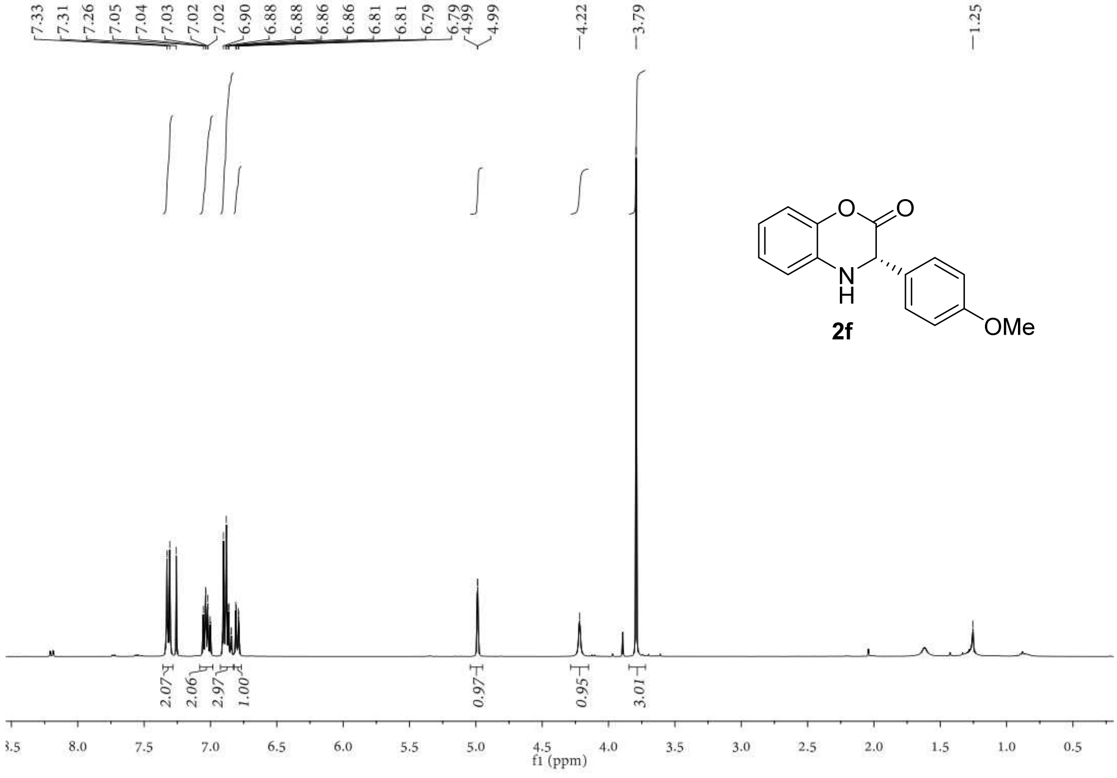
7.33
7.31
7.26
7.05
7.04
7.03
7.02
7.02
6.90
6.88
6.88
6.86
6.86
6.81
6.81
6.79
6.79
4.99
4.99
4.22
3.79
1.25
2.07
2.06
2.97
1.00
0.97
0.95
3.01
f1 (ppm)
2f
OMe

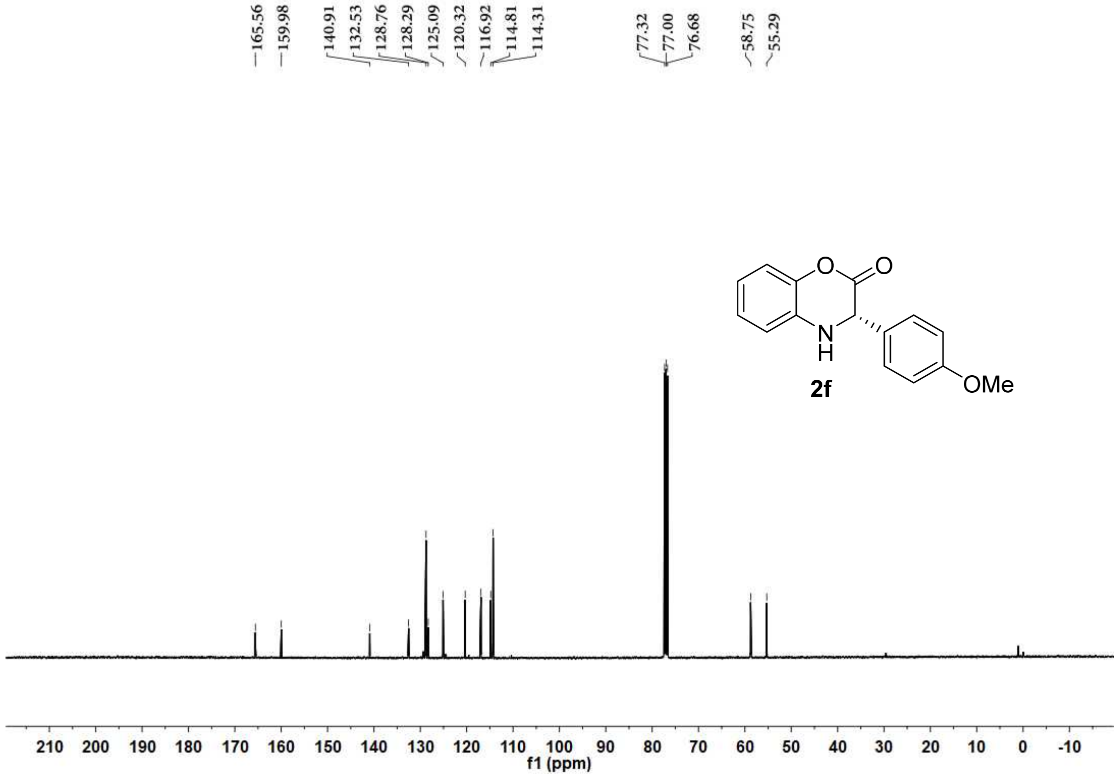
165.56
159.98
140.91
132.53
128.76
128.29
125.09
120.32
116.92
114.81
114.31
77.32
77.00
76.68
58.75
55.29
f1 (ppm)
2f
OMe

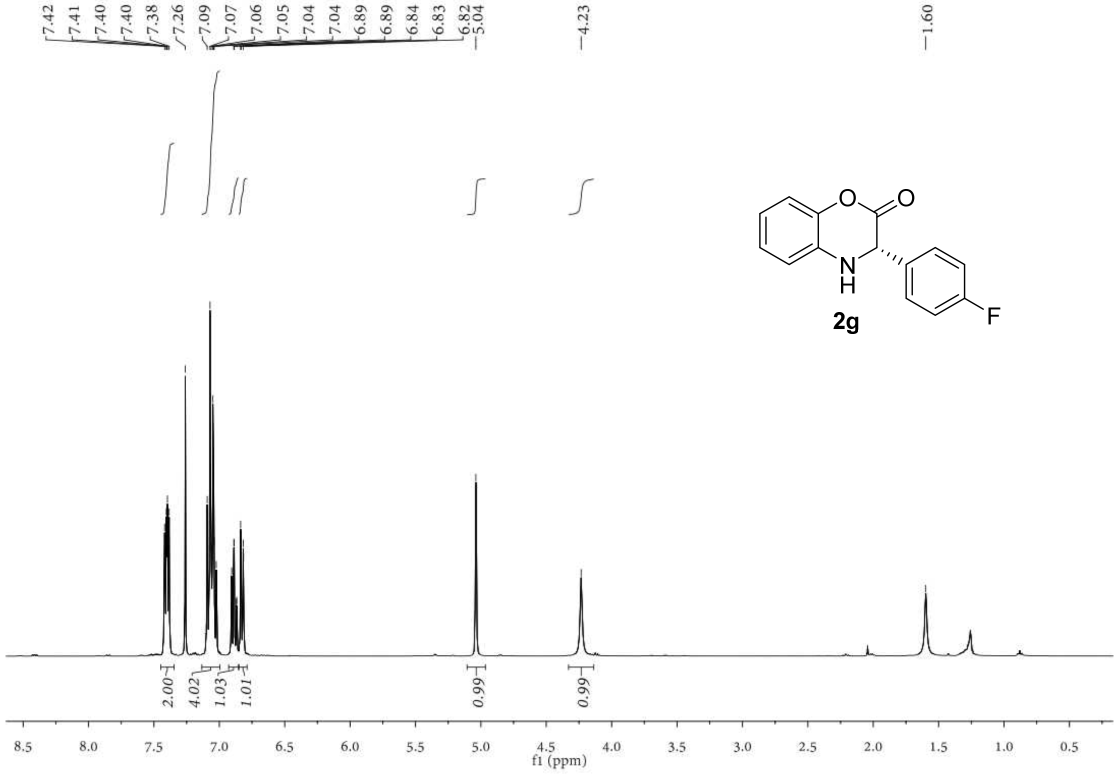

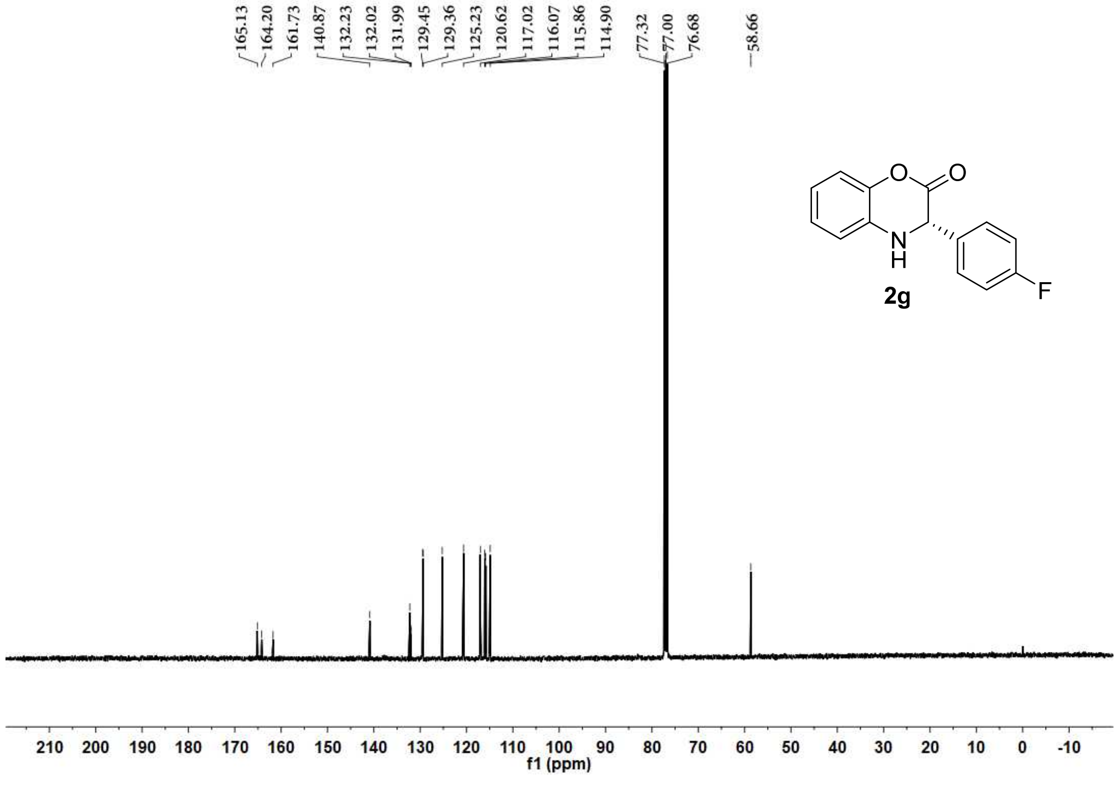

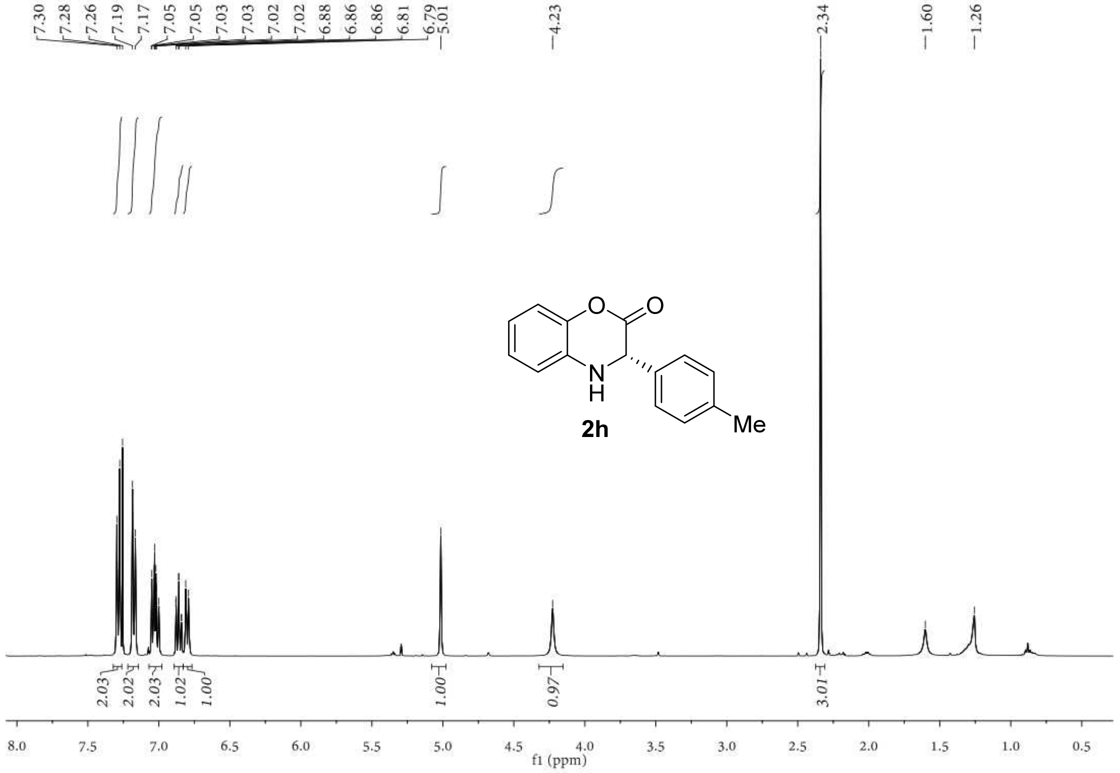
7.30
7.28
7.26
7.19
7.17
7.05
7.05
7.03
7.03
7.02
7.02
6.88
6.86
6.86
6.81
6.79
5.01
4.23
2.34
1.60
1.26
2h
Me
2.03
2.02
2.03
1.02
1.00
1.00
0.97
3.01
8.0 7.5 7.0 6.5 6.0 5.5 5.0 4.5 4.0 3.5 3.0 2.5 2.0 1.5 1.0 0.5
f1 (ppm)

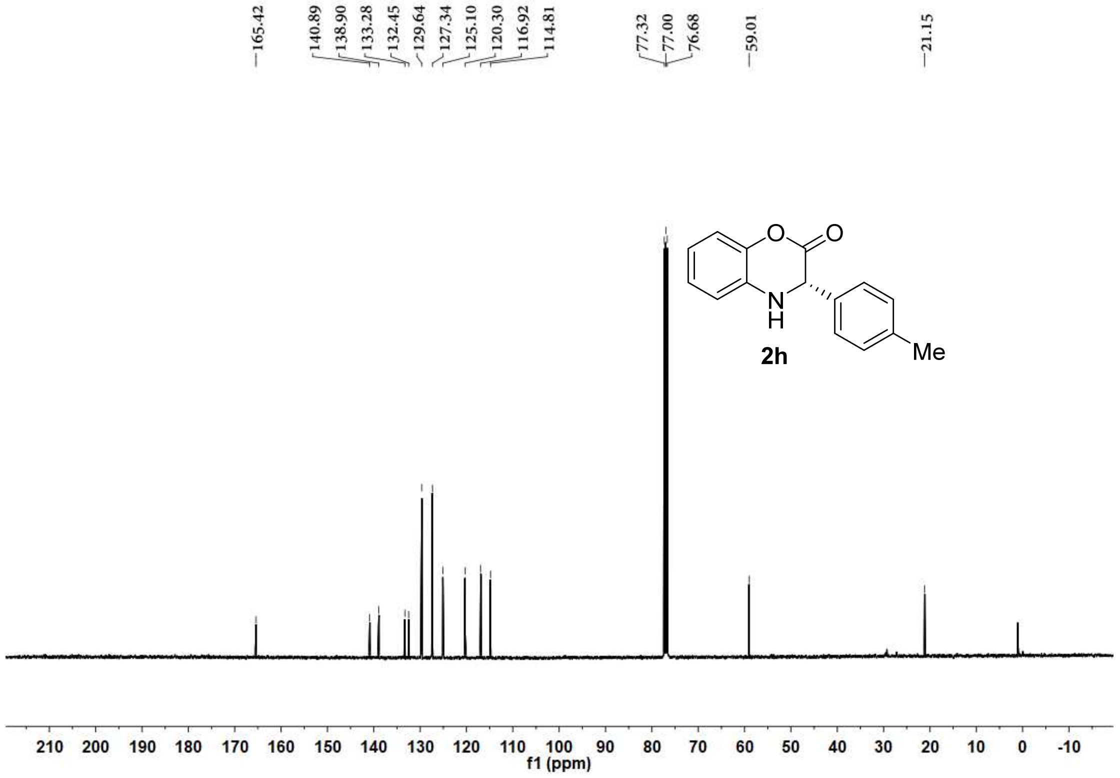
165.42
140.89
138.90
133.28
132.45
129.64
127.34
125.10
120.30
116.92
114.81
77.32
77.00
76.68
59.01
21.15
2h
Me
210 200 190 180 170 160 150 140 130 120 110 100 90 80 70 60 50 40 30 20 10 0 -10
f1 (ppm)

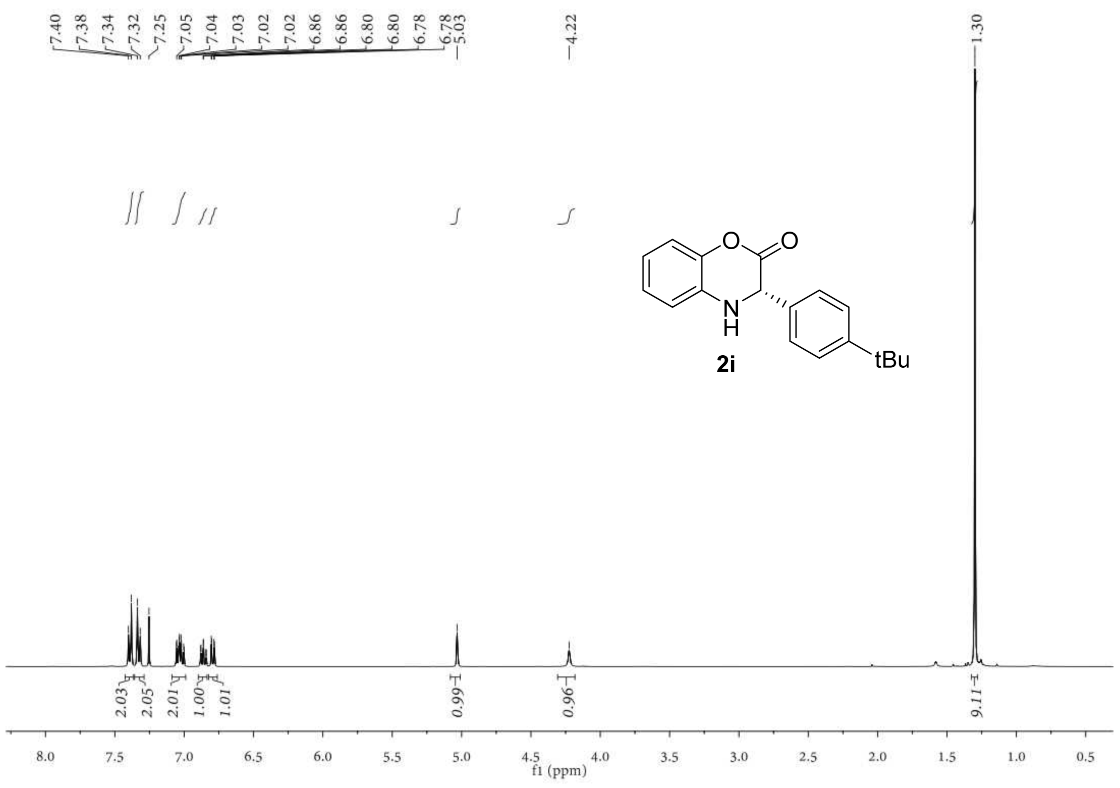
7.40
7.38
7.34
7.32
7.25
7.05
7.04
7.03
7.02
7.02
6.86
6.86
6.80
6.80
6.78
6.78
5.03
4.22
1.30
2i
tBu
2.03
2.05
2.01
1.00
1.01
0.99
0.96
9.11
f1 (ppm)

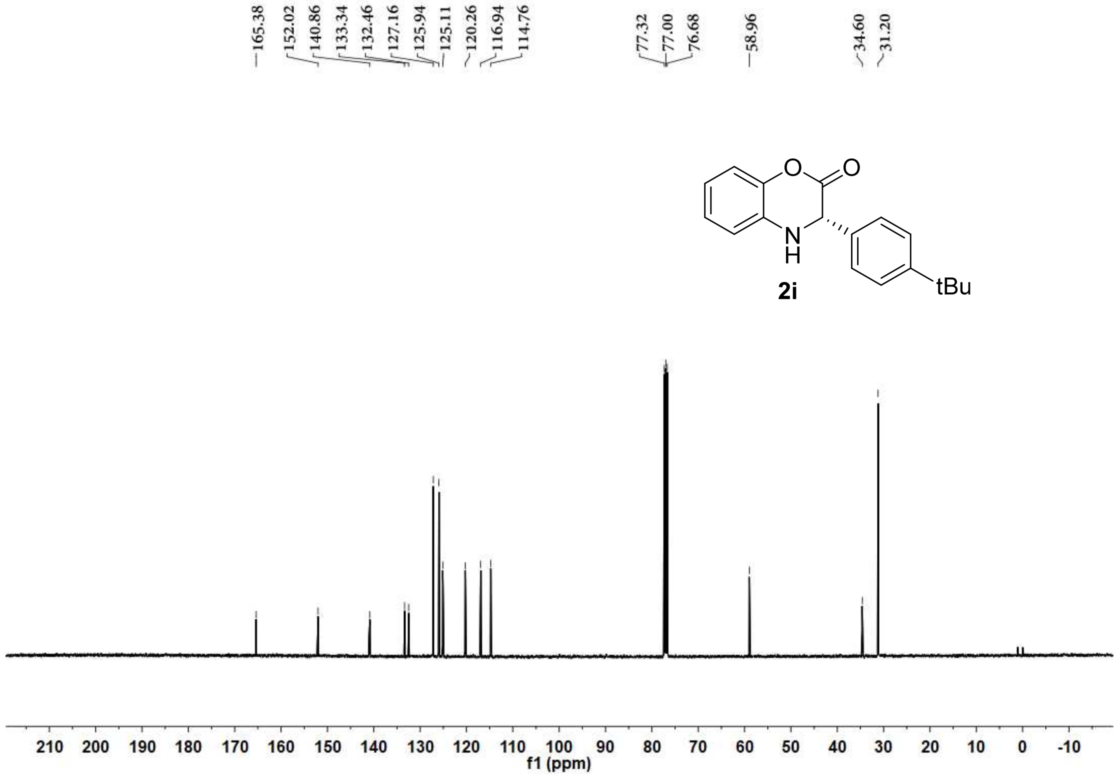
165.38
152.02
140.86
133.34
132.46
127.16
125.94
125.11
120.26
116.94
114.76
77.32
77.00
76.68
58.96
34.60
31.20
2i
tBu
f1 (ppm)

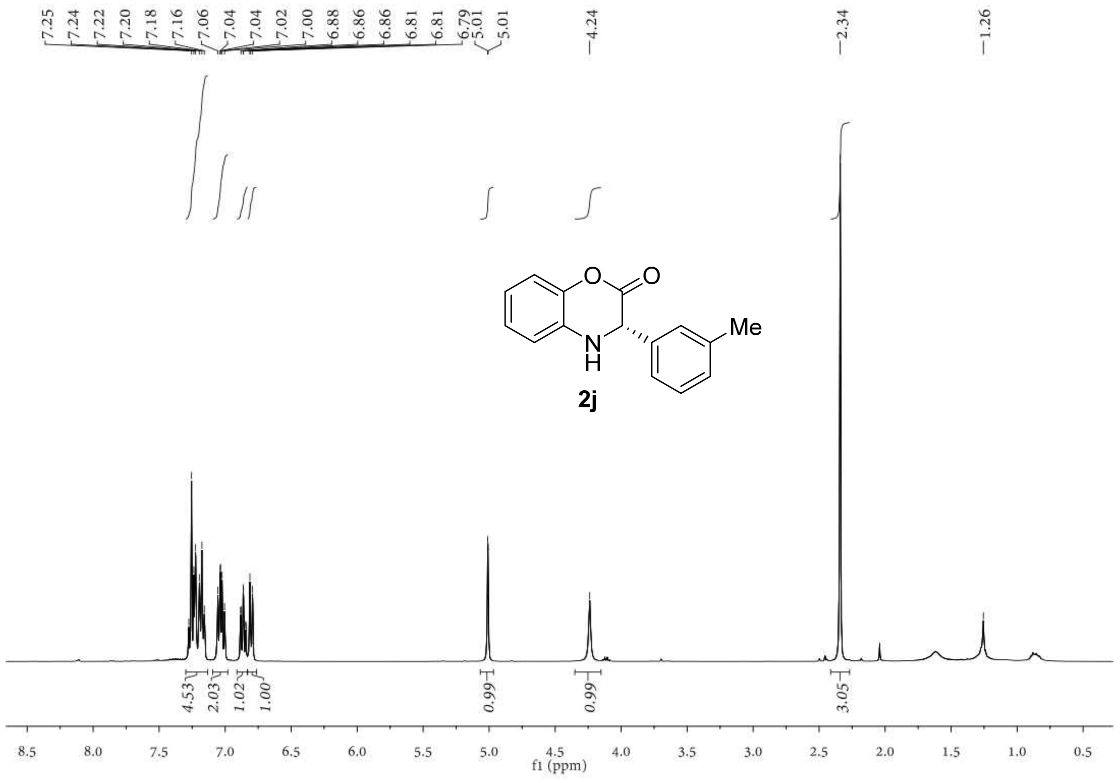
7.25 7.24 7.22 7.20 7.18 7.16 7.06 7.04 7.04 7.02 7.00 6.88 6.86 6.86 6.81 6.81 6.79 5.01 5.01
4.24
2.34
1.26
Me
N
H
O
O
2j
4.53 2.03 1.02 1.00
0.99
0.99
3.05
8.5 8.0 7.5 7.0 6.5 6.0 5.5 5.0 4.5 4.0 3.5 3.0 2.5 2.0 1.5 1.0 0.5
f1 (ppm)

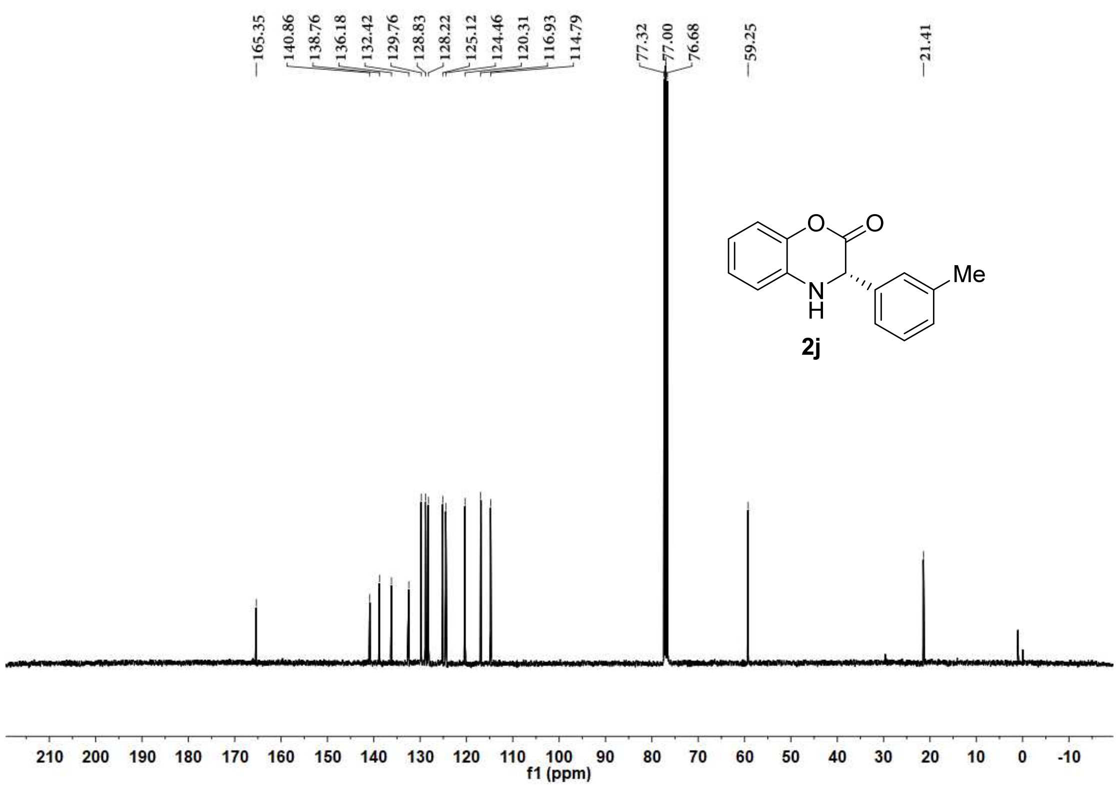
165.35
140.86 138.76 136.18 132.42 129.76 128.83 128.22 125.12 124.46 120.31 116.93 114.79
77.32 77.00 76.68
59.25
21.41
Me
N
H
O
O
2j
210 200 190 180 170 160 150 140 130 120 110 100 90 80 70 60 50 40 30 20 10 0 -10
f1 (ppm)

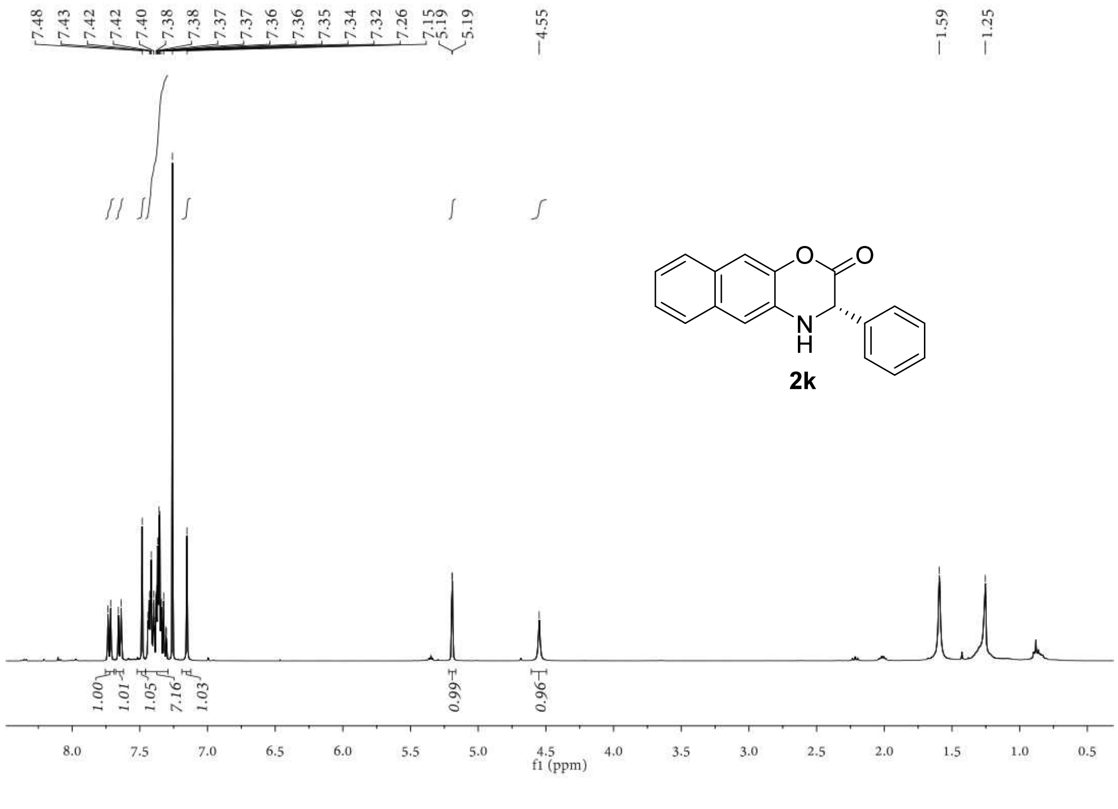
7.48 7.43 7.42 7.42 7.40 7.38 7.38 7.37 7.37 7.36 7.36 7.35 7.34 7.32 7.26 7.15 5.19 5.19
4.55
1.59
1.25
1.00 1.01 1.05 7.16 1.03
0.99
0.96
8.0 7.5 7.0 6.5 6.0 5.5 5.0 4.5 4.0 3.5 3.0 2.5 2.0 1.5 1.0 0.5
f1 (ppm)
O
O
N
H
2k

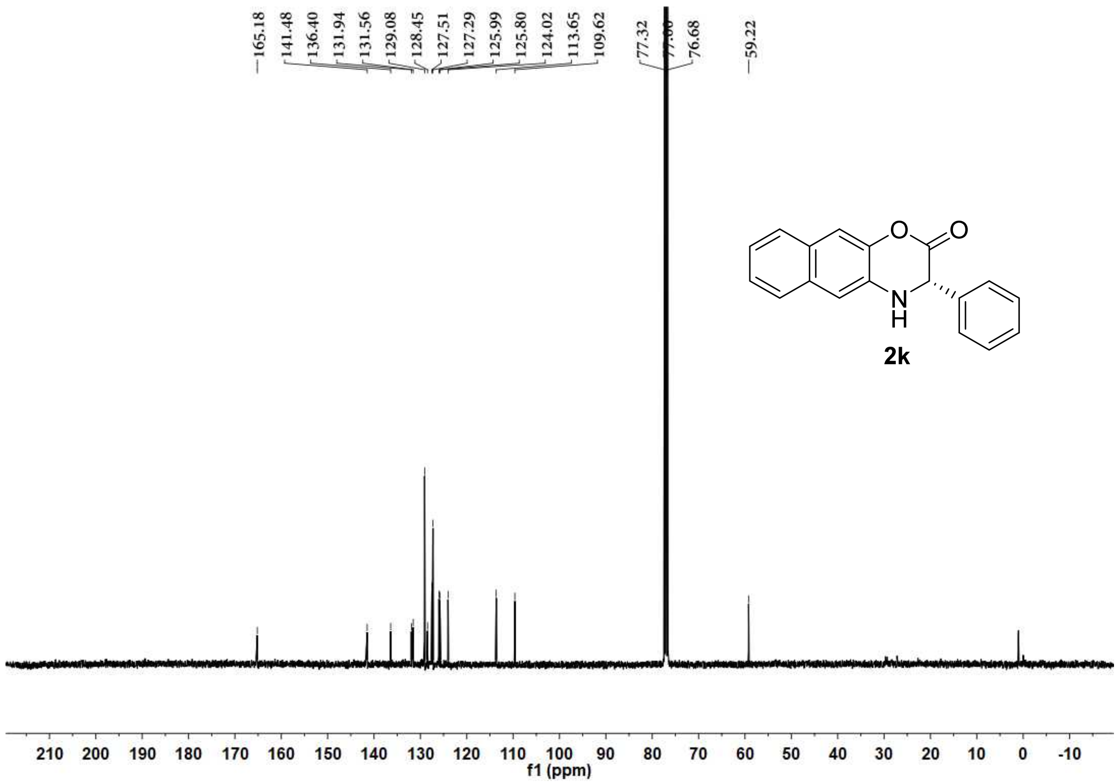
165.18
141.48 136.40 131.94 131.56 129.08 128.45 127.51 127.29 125.99 125.80 124.02 113.65 109.62
77.32 76.68
59.22
210 200 190 180 170 160 150 140 130 120 110 100 90 80 70 60 50 40 30 20 10 0 -10
f1 (ppm)
O
O
N
H
2k

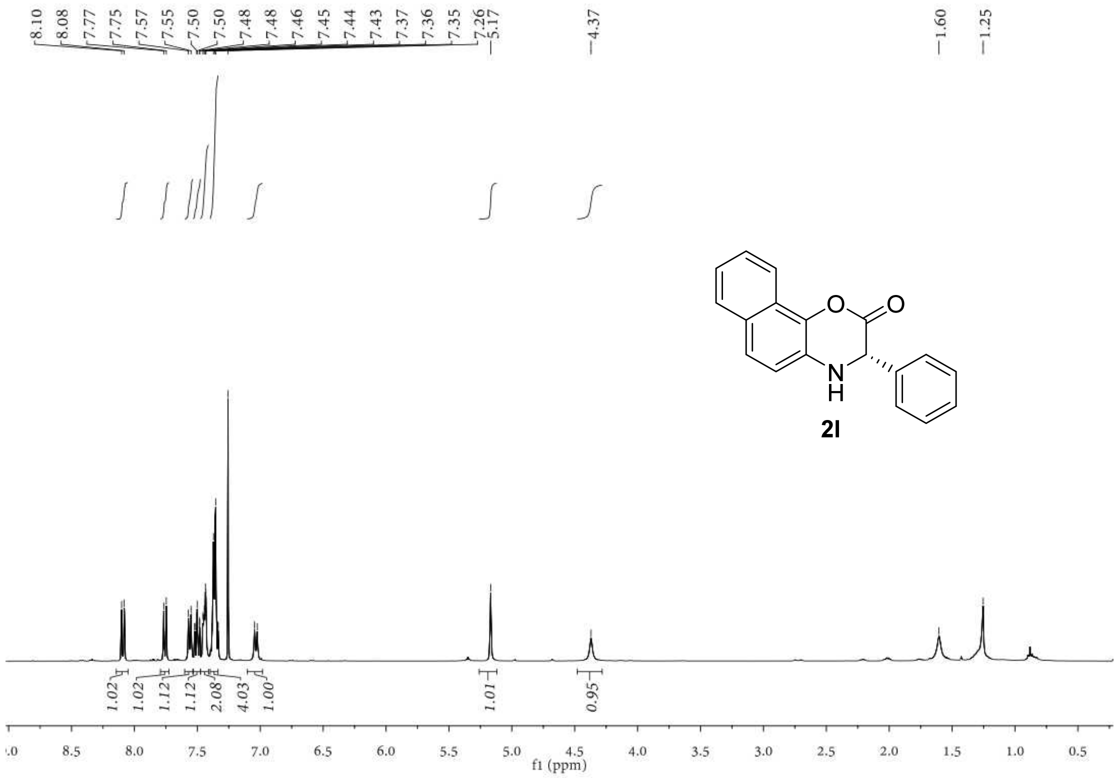
8.10 8.08 7.77 7.75 7.57 7.55 7.50 7.50 7.48 7.48 7.46 7.45 7.44 7.43 7.37 7.36 7.35 7.26 5.17
4.37
1.60
1.25
O
O
N
H
2l
1.02 1.02 1.12 1.12 2.08 4.03 1.00
1.01
0.95
f1 (ppm)

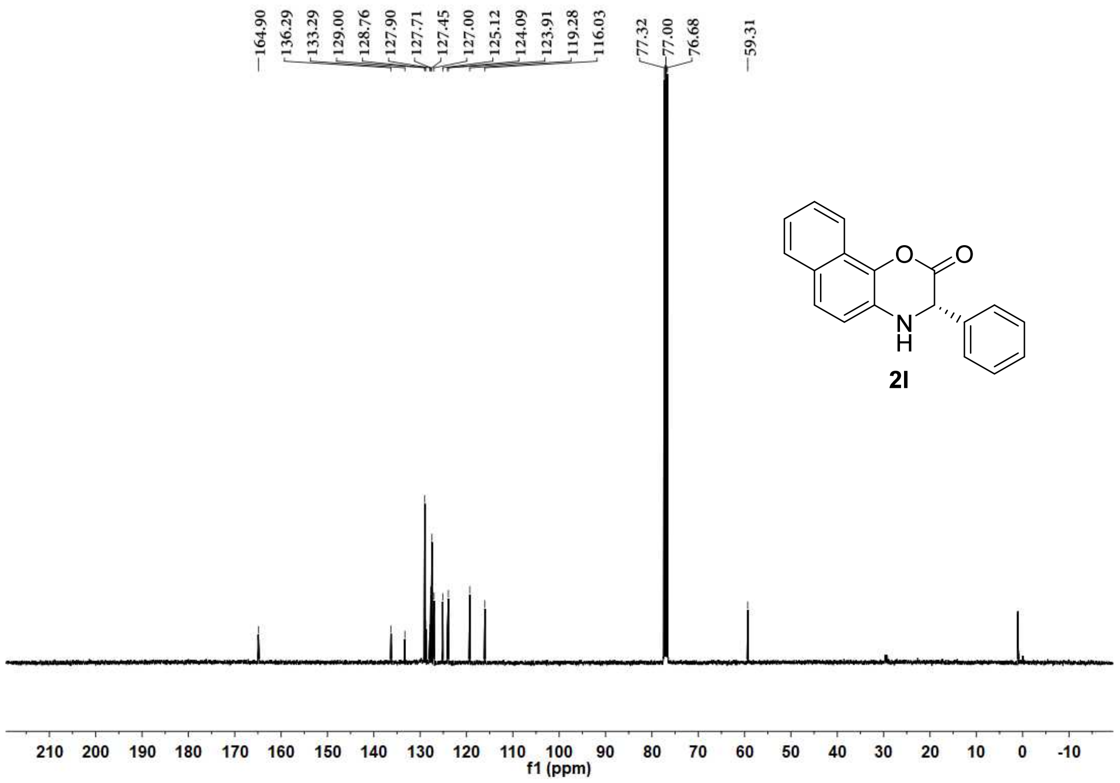
164.90
136.29 133.29 129.00 128.76 127.90 127.71 127.45 127.00 125.12 124.09 123.91 119.28 116.03
77.32 77.00 76.68
59.31
O
O
N
H
2l
f1 (ppm)

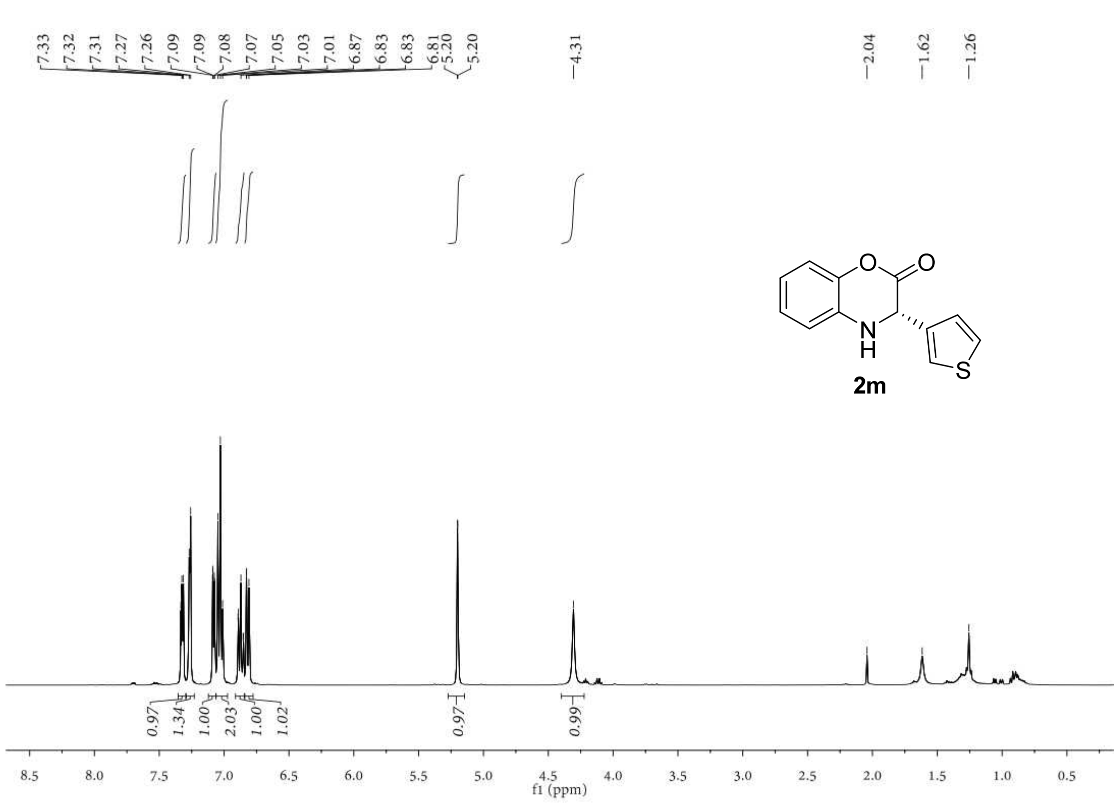
7.33 7.32 7.31 7.27 7.26 7.09 7.09 7.08 7.07 7.05 7.03 7.01 6.87 6.83 6.83 6.81 5.20 5.20
4.31
2.04
1.62
1.26
2m
0.97 1.34 1.00 2.03 1.00 1.02
0.97
0.99
8.5 8.0 7.5 7.0 6.5 6.0 5.5 5.0 4.5 4.0 3.5 3.0 2.5 2.0 1.5 1.0 0.5
f1 (ppm)

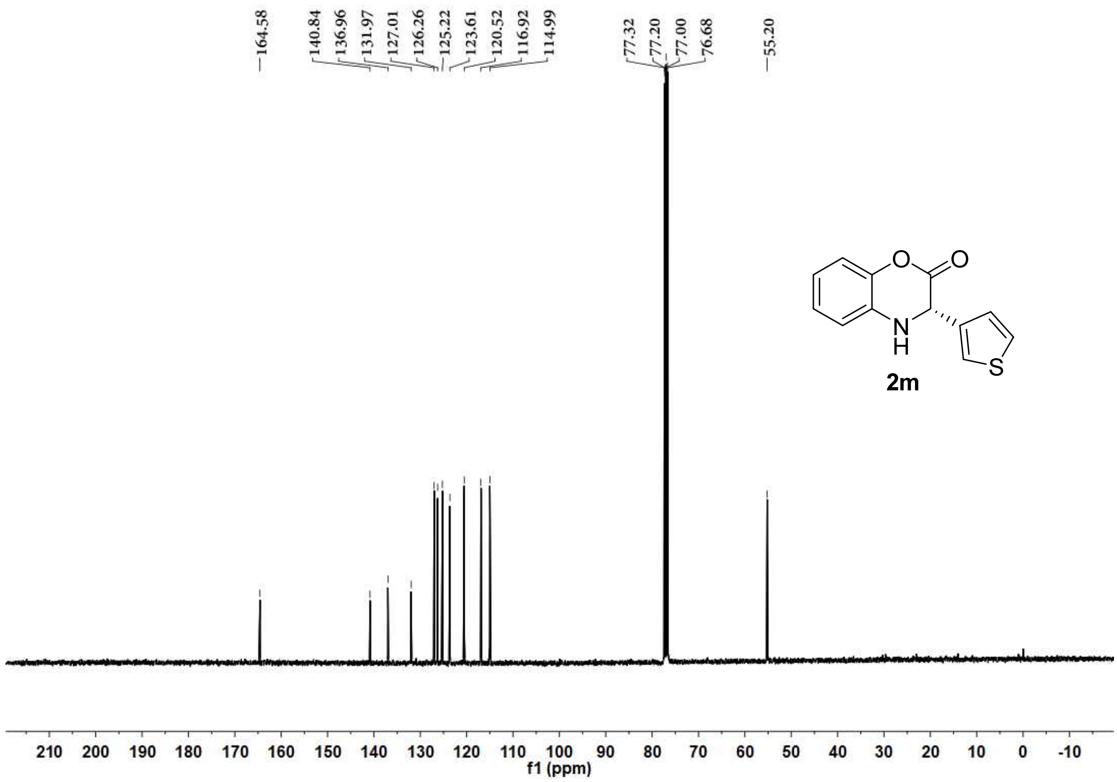
164.58
140.84 136.96 131.97 127.01 126.26 125.22 123.61 120.52 116.92 114.99
77.32 77.20 77.00 76.68
55.20
2m
210 200 190 180 170 160 150 140 130 120 110 100 90 80 70 60 50 40 30 20 10 0 -10
f1 (ppm)

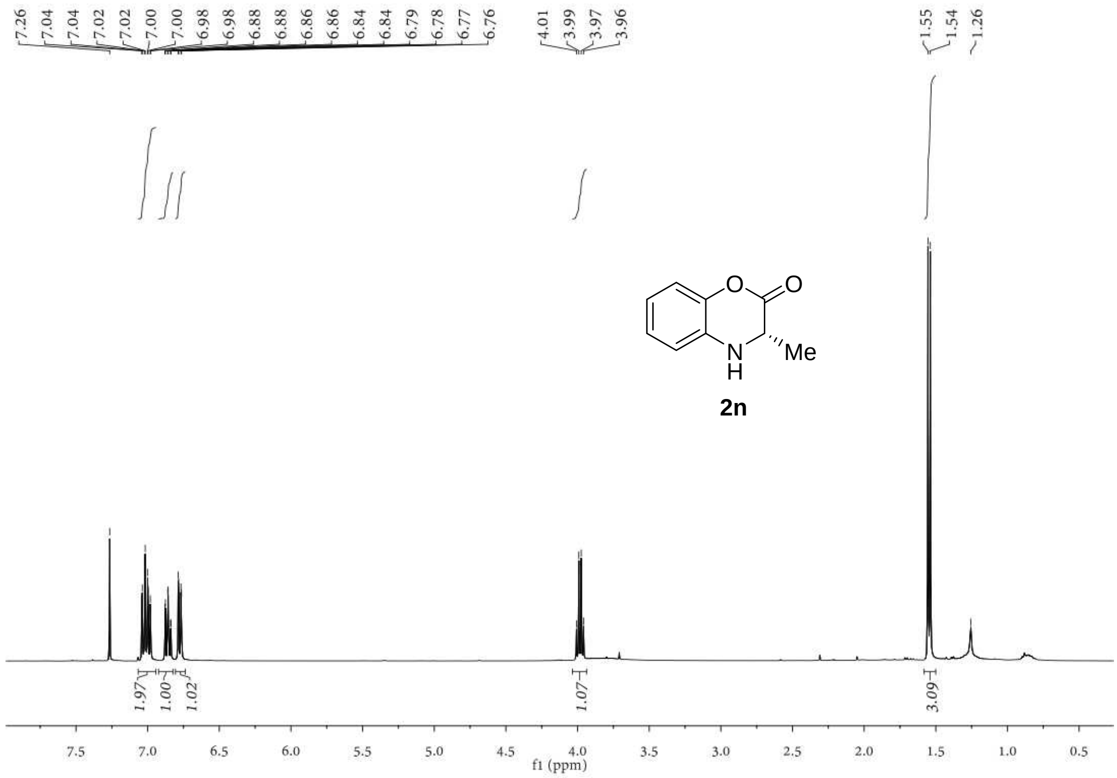
7.26
7.04
7.04
7.02
7.02
7.00
7.00
6.98
6.98
6.88
6.88
6.86
6.86
6.84
6.84
6.79
6.78
6.77
6.76
4.01
3.99
3.97
3.96
1.55
1.54
1.26
1.97
1.00
1.02
1.07
3.09
f1 (ppm)

2n

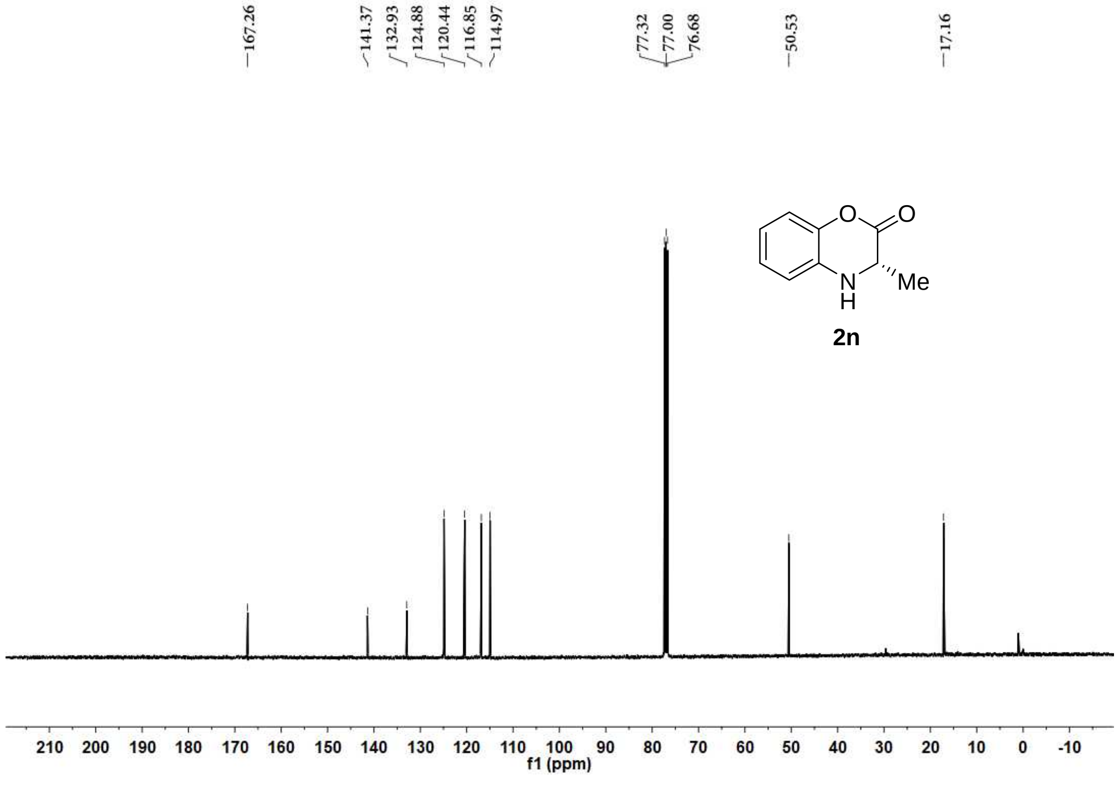
167.26
141.37
132.93
124.88
120.44
116.85
114.97
77.32
77.00
76.68
50.53
17.16
f1 (ppm)

2n

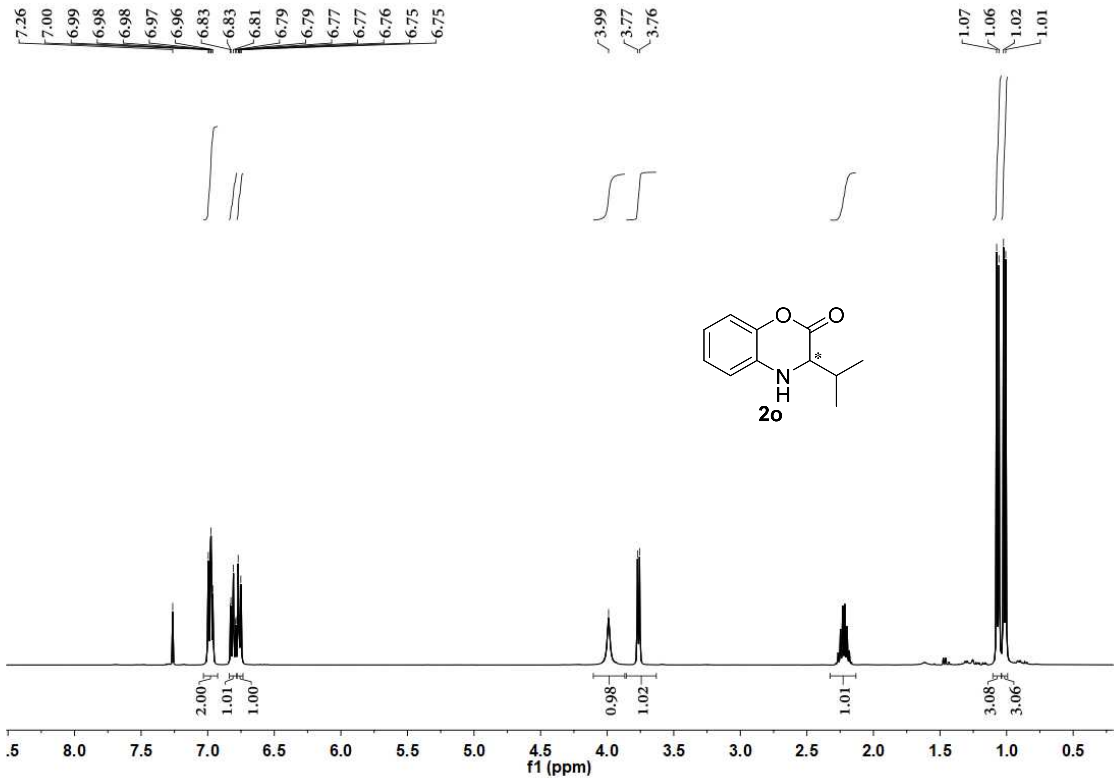

7.26
7.00
6.99
6.98
6.98
6.97
6.96
6.83
6.83
6.81
6.79
6.79
6.77
6.77
6.76
6.75
6.75
3.99
3.77
3.76
1.07
1.06
1.02
1.01
2o
2.00
1.01
1.00
0.98
1.02
1.01
3.08
3.06
f1 (ppm)

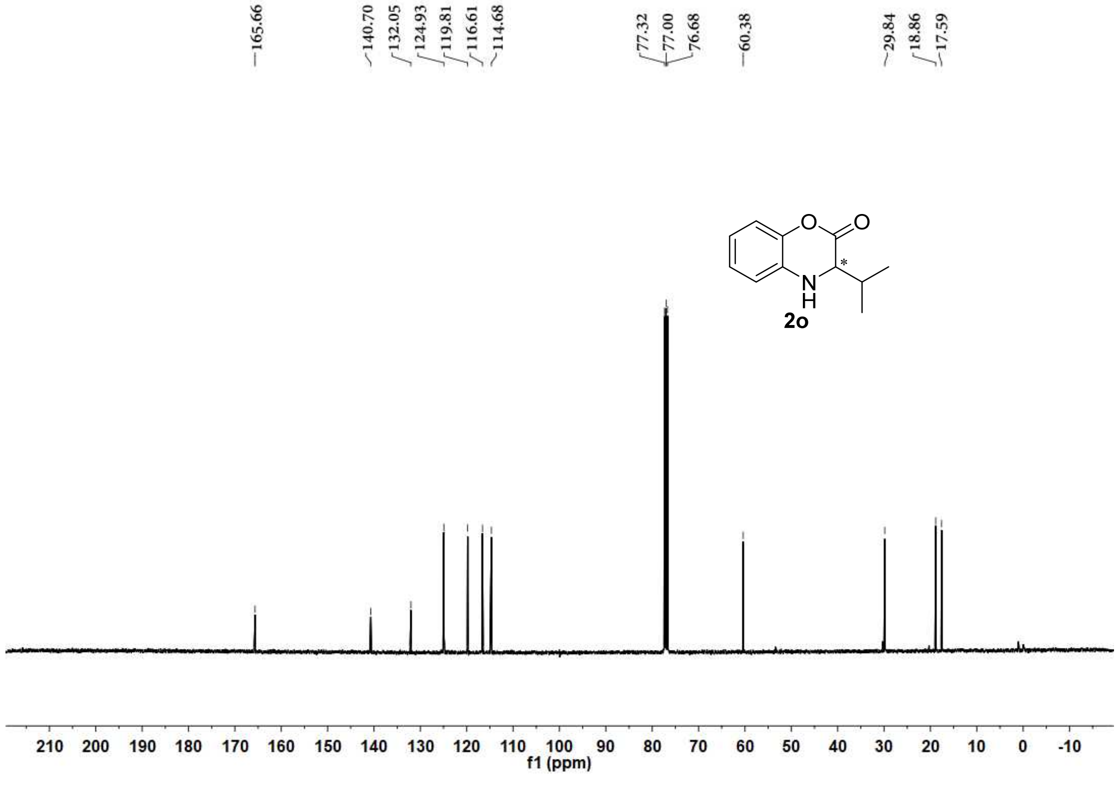

165.66
140.70
132.05
124.93
119.81
116.61
114.68
77.32
77.00
76.68
60.38
29.84
18.86
17.59
2o
f1 (ppm)

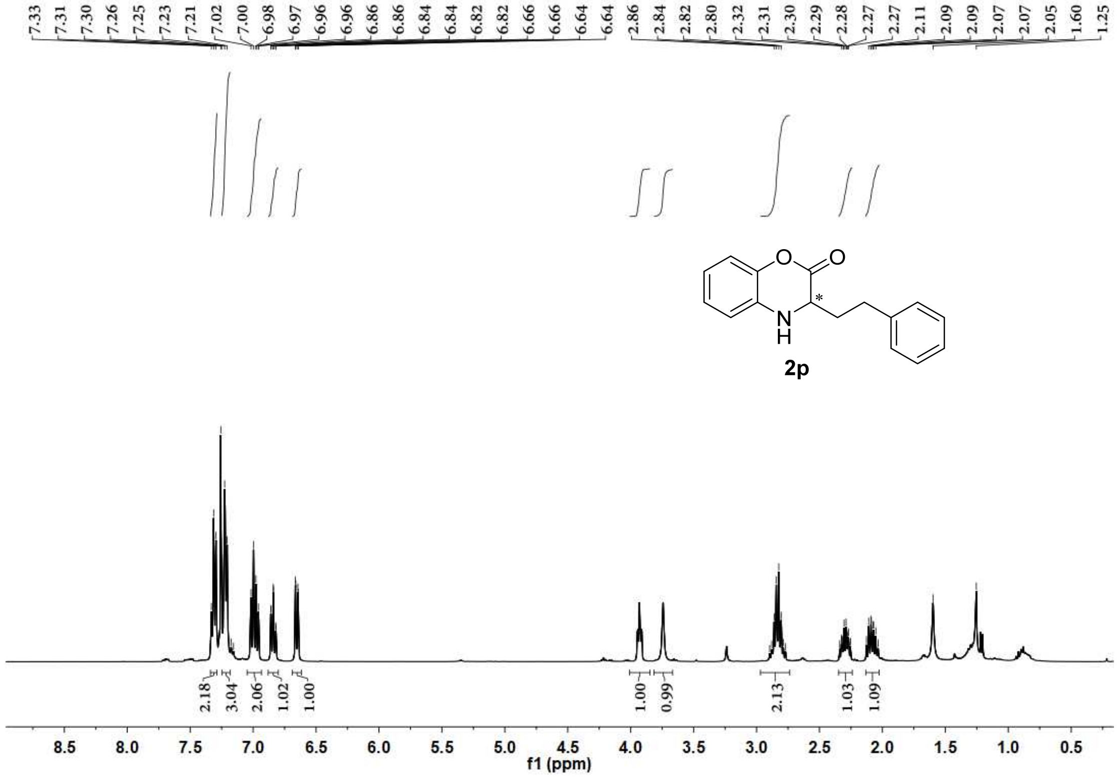
7.33 7.31 7.30 7.26 7.25 7.23 7.21 7.02 7.00 6.98 6.97 6.96 6.96 6.86 6.86 6.84 6.84 6.82 6.82 6.66 6.66 6.64 6.64 2.86 2.84 2.82 2.80 2.32 2.31 2.30 2.29 2.28 2.27 2.27 2.11 2.09 2.09 2.07 2.07 2.05 1.60 1.25
O
O
N
H
*
2p
2.18 3.04 2.06 1.02 1.00 1.00 0.99 2.13 1.03 1.09
8.5 8.0 7.5 7.0 6.5 6.0 5.5 5.0 4.5 4.0 3.5 3.0 2.5 2.0 1.5 1.0 0.5
f1 (ppm)

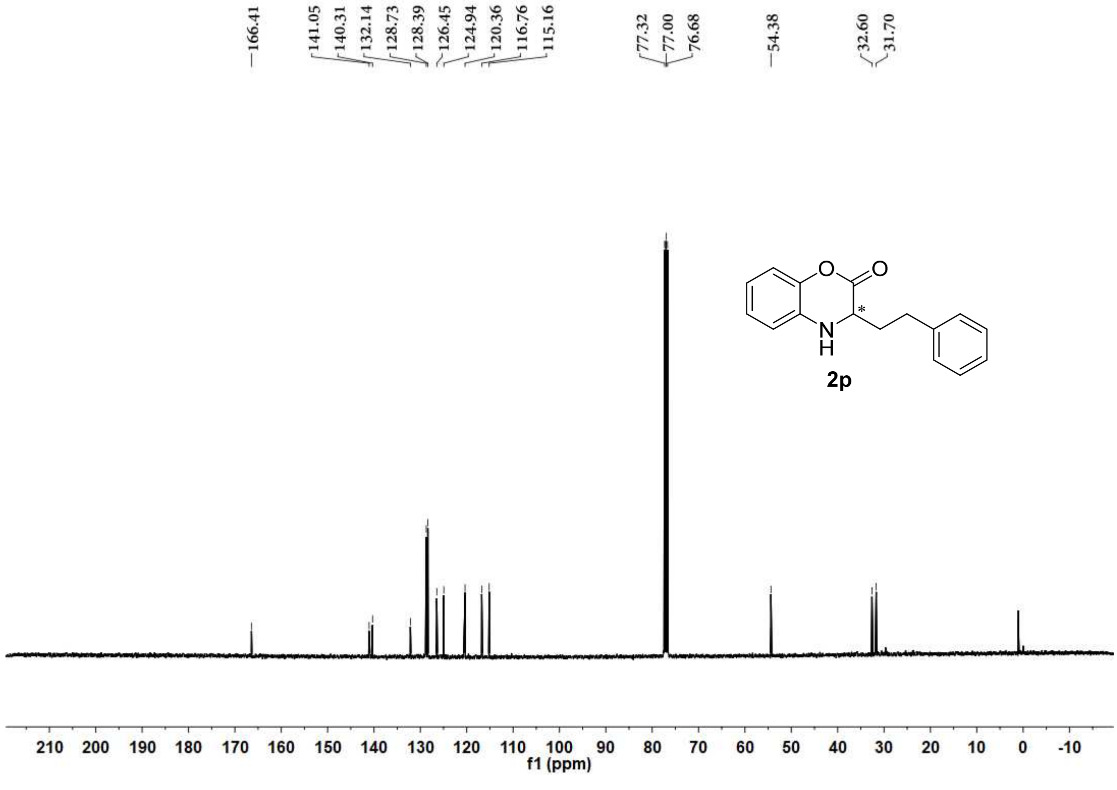
166.41 141.05 140.31 132.14 128.73 128.39 126.45 124.94 120.36 116.76 115.16 77.32 77.00 76.68 54.38 32.60 31.70
O
O
N
H
*
2p
210 200 190 180 170 160 150 140 130 120 110 100 90 80 70 60 50 40 30 20 10 0 -10
f1 (ppm)

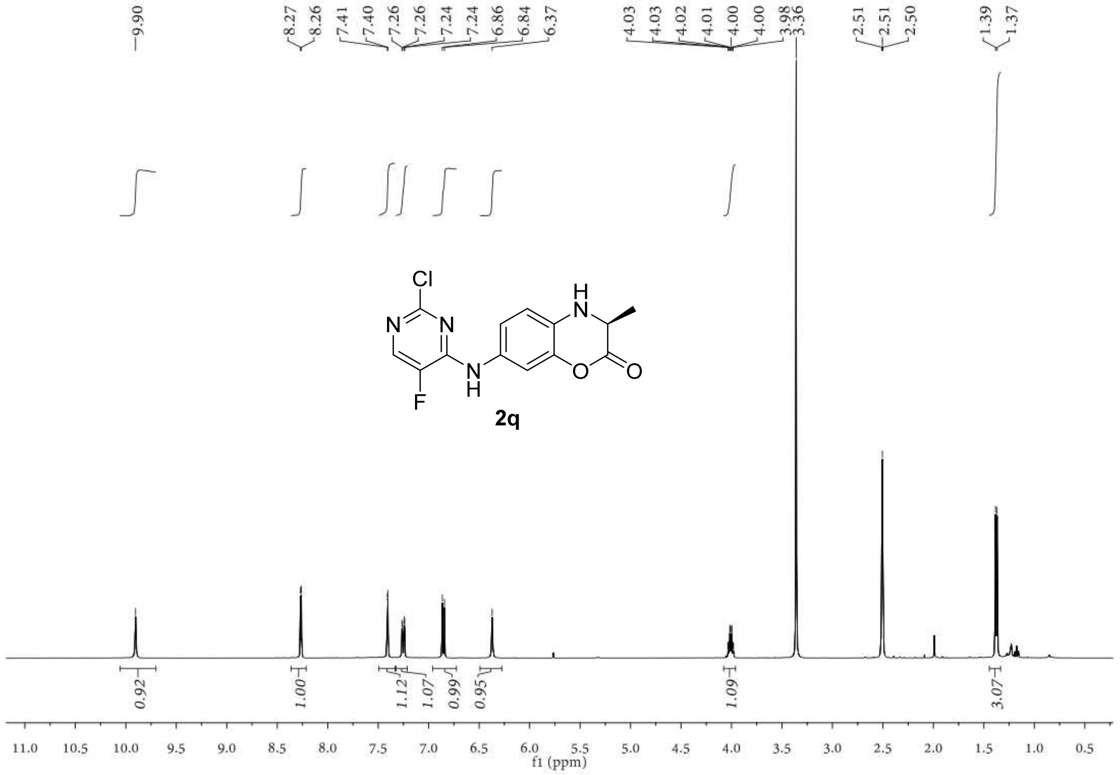
9.90
8.27 8.26 7.41 7.40 7.26 7.26 7.24 7.24 6.86 6.84 6.37
4.03 4.03 4.02 4.01 4.00 4.00 3.98 3.36
2.51 2.51 2.50
1.39 1.37
Cl
N
N
H
N
H
O
O
F
2q
0.92 1.00 1.12 1.07 0.99 0.95 1.09 3.07
11.0 10.5 10.0 9.5 9.0 8.5 8.0 7.5 7.0 6.5 6.0 5.5 5.0 4.5 4.0 3.5 3.0 2.5 2.0 1.5 1.0 0.5
f1 (ppm)

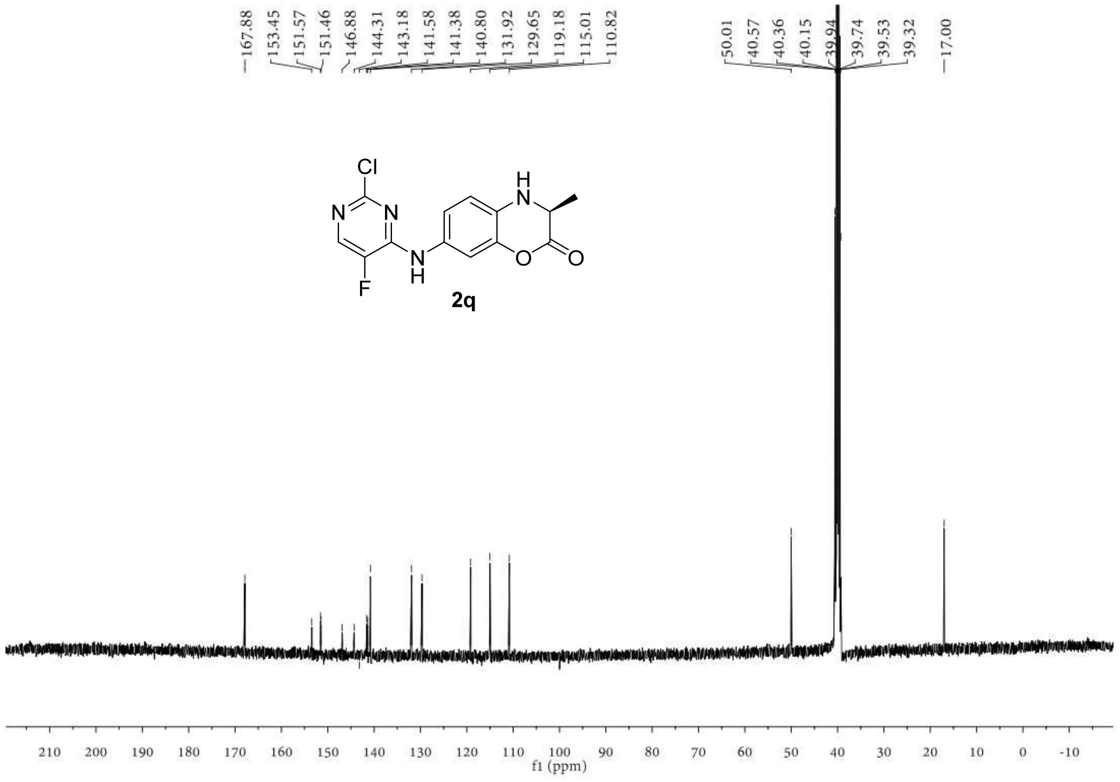
167.88 153.45 151.57 151.46 146.88 144.31 143.18 141.58 141.38 140.80 131.92 129.65 119.18 115.01 110.82
50.01 40.57 40.36 40.15 39.94 39.74 39.53 39.32 17.00
Cl
N
N
H
N
H
O
O
F
2q
210 200 190 180 170 160 150 140 130 120 110 100 90 80 70 60 50 40 30 20 10 0 -10
f1 (ppm)

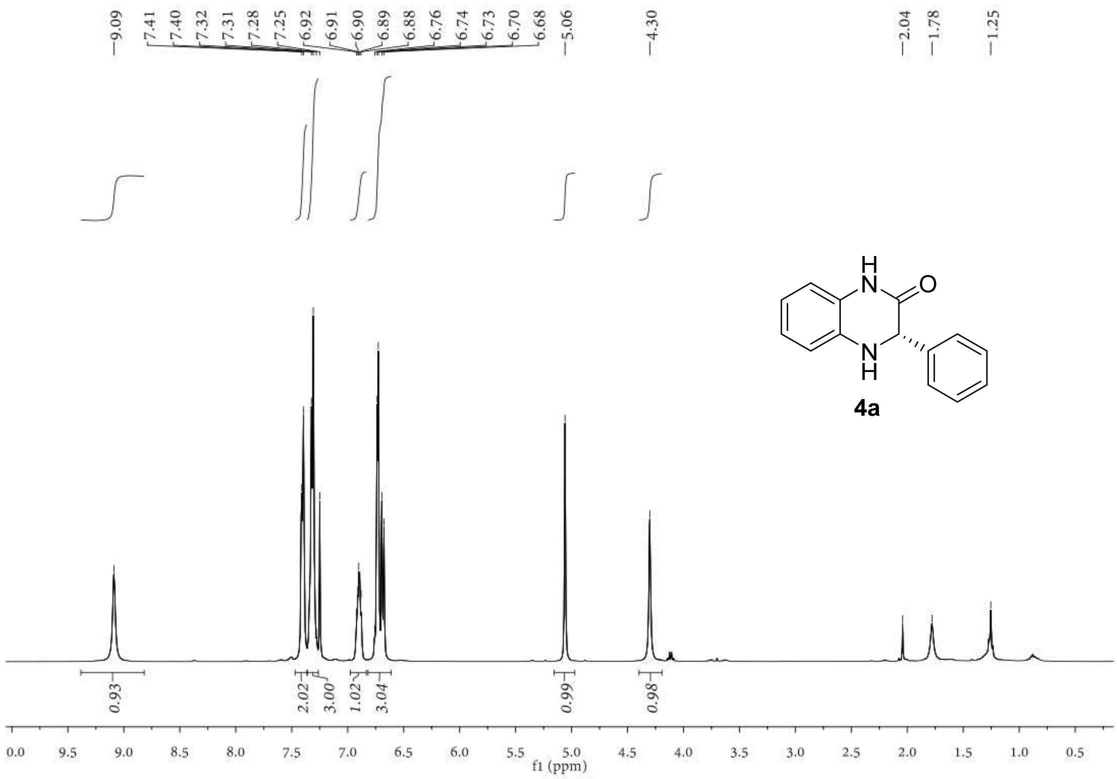
9.09
7.41
7.40
7.32
7.31
7.28
7.25
6.92
6.91
6.90
6.89
6.88
6.76
6.74
6.73
6.70
6.68
5.06
4.30
2.04
1.78
1.25
0.93
2.02
3.00
1.02
3.04
0.99
0.98
4a
f1 (ppm)

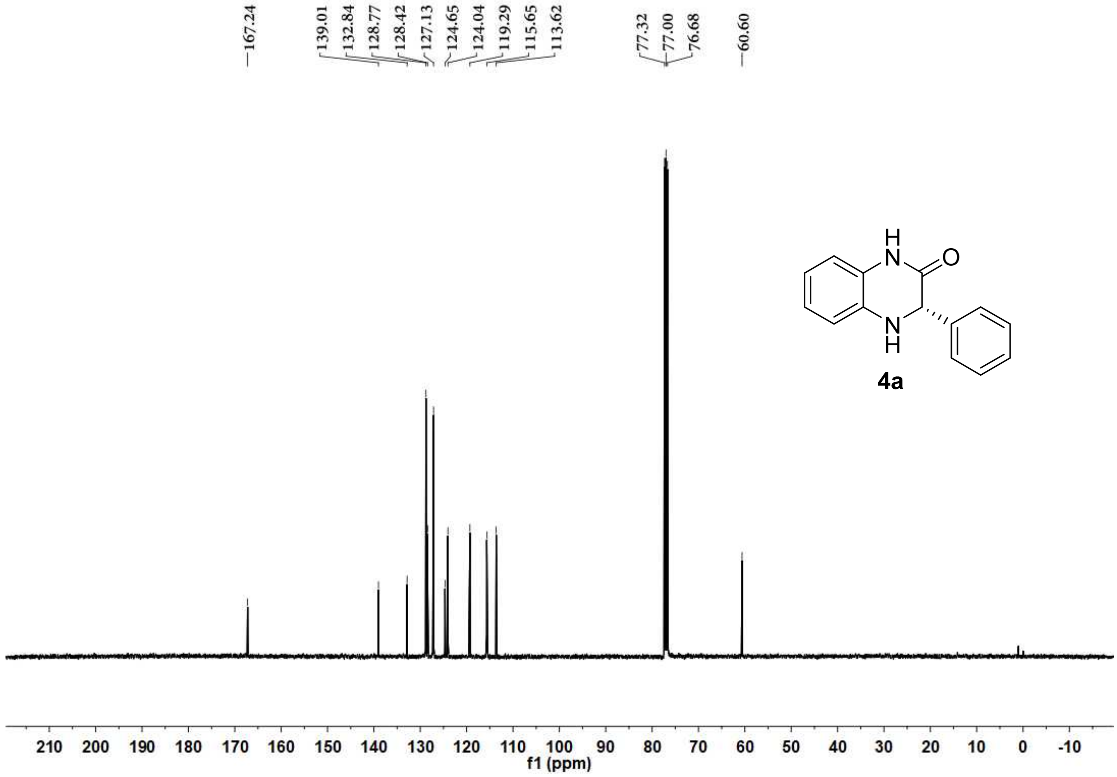
167.24
139.01
132.84
128.77
128.42
127.13
124.65
124.04
119.29
115.65
113.62
77.32
77.00
76.68
60.60
4a
f1 (ppm)

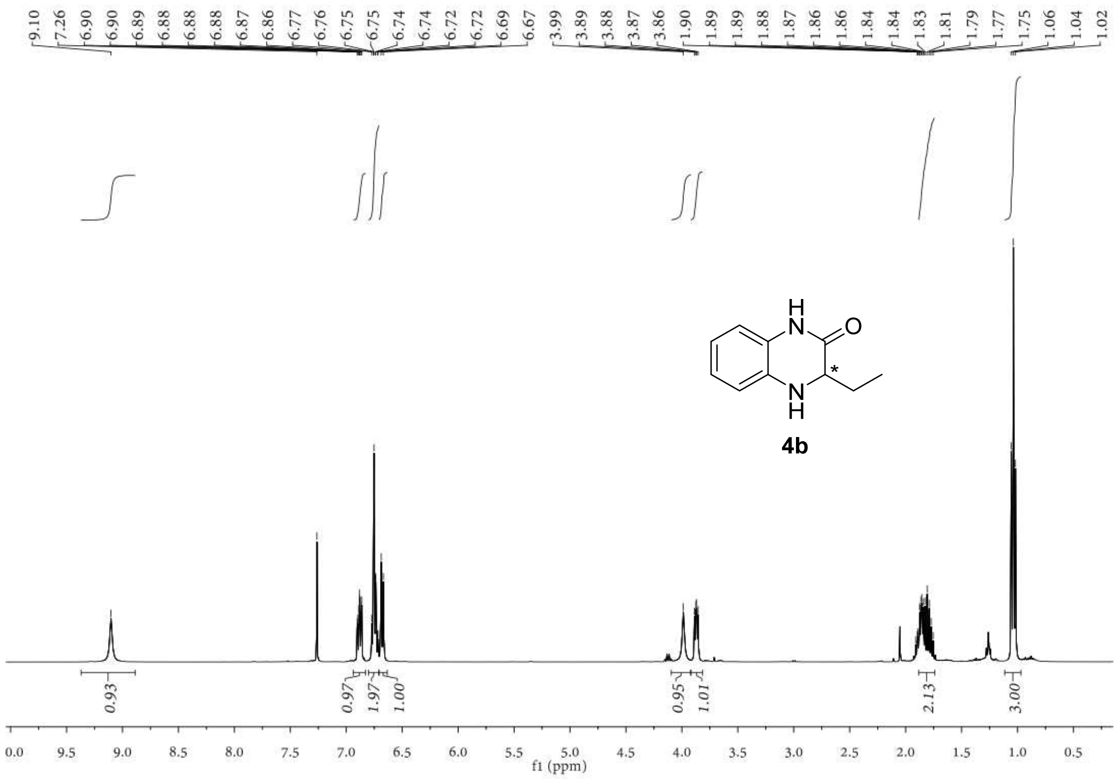
9.10
7.26
6.90
6.90
6.89
6.88
6.88
6.88
6.87
6.86
6.77
6.76
6.75
6.75
6.74
6.74
6.72
6.72
6.69
6.67
3.99
3.89
3.88
3.87
3.86
1.90
1.89
1.89
1.88
1.87
1.86
1.86
1.84
1.84
1.83
1.81
1.79
1.77
1.75
1.06
1.04
1.02
4b
0.93
0.97
1.97
1.00
0.95
1.01
2.13
3.00
f1 (ppm)

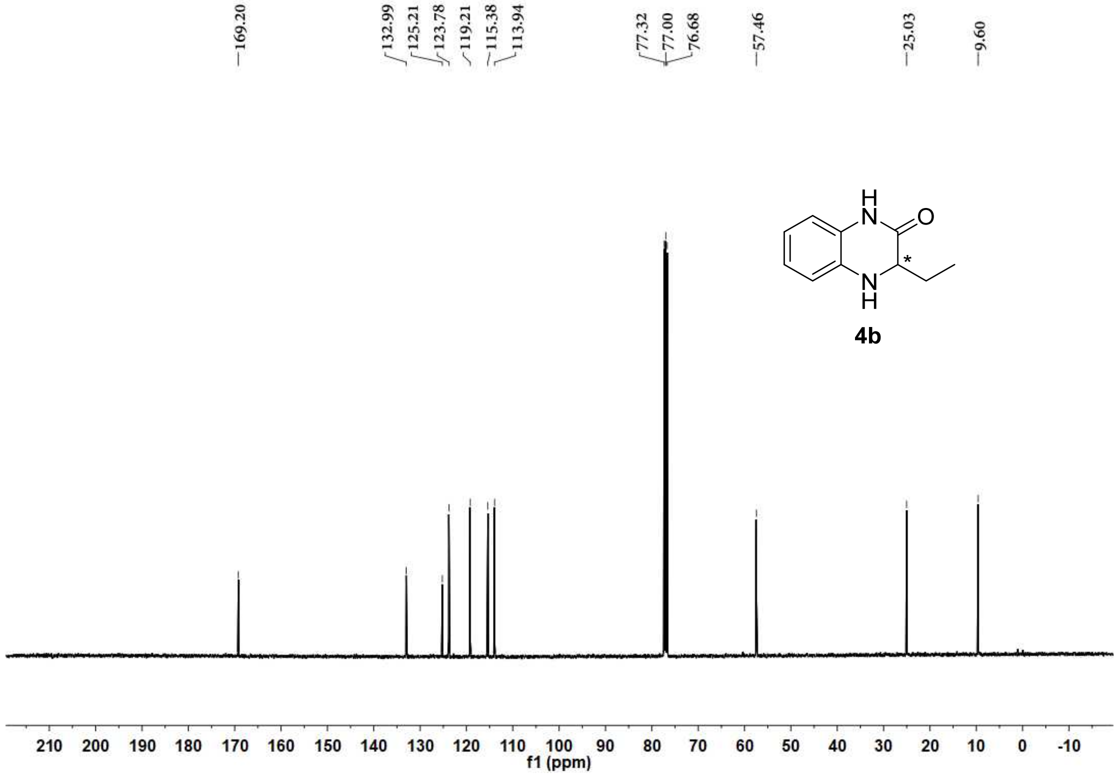
169.20
132.99
125.21
123.78
119.21
115.38
113.94
77.32
77.00
76.68
57.46
25.03
9.60
4b
f1 (ppm)

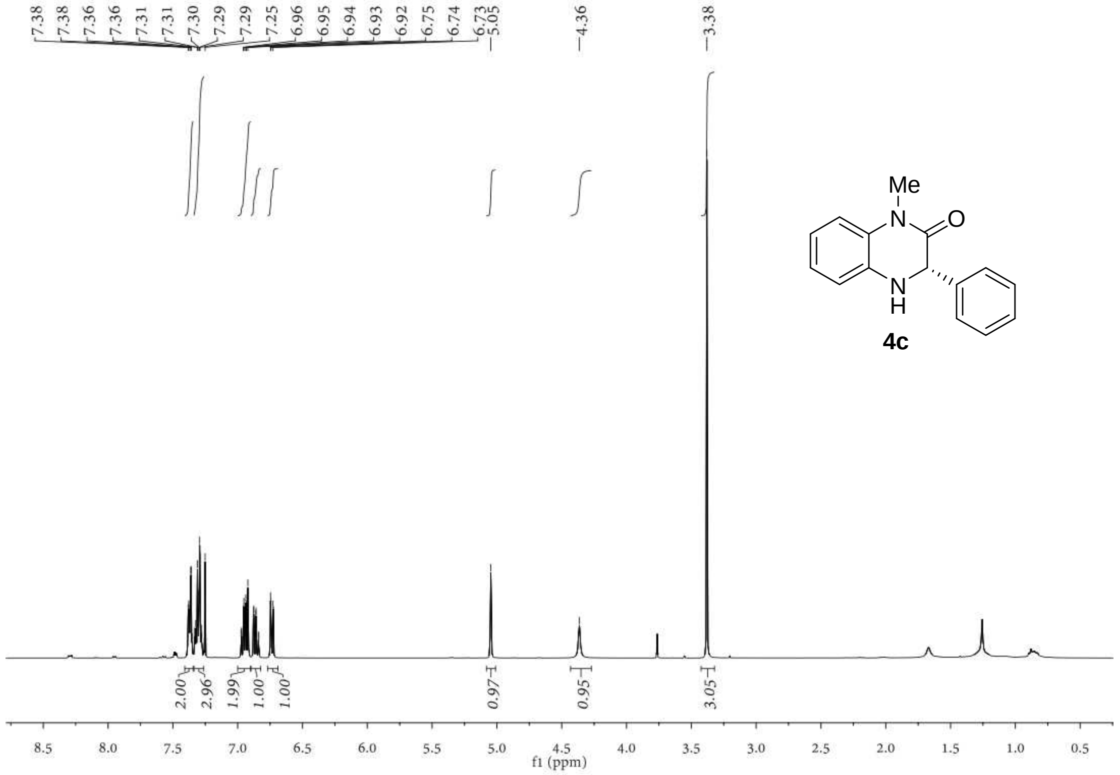
7.38
7.38
7.36
7.36
7.31
7.31
7.30
7.29
7.29
7.25
6.96
6.95
6.94
6.93
6.92
6.75
6.74
6.73
5.05
4.36
3.38
Me
N
O
N
H
4c
2.00
2.96
1.99
1.00
1.00
0.97
0.95
3.05
8.5 8.0 7.5 7.0 6.5 6.0 5.5 5.0 4.5 4.0 3.5 3.0 2.5 2.0 1.5 1.0 0.5
f1 (ppm)

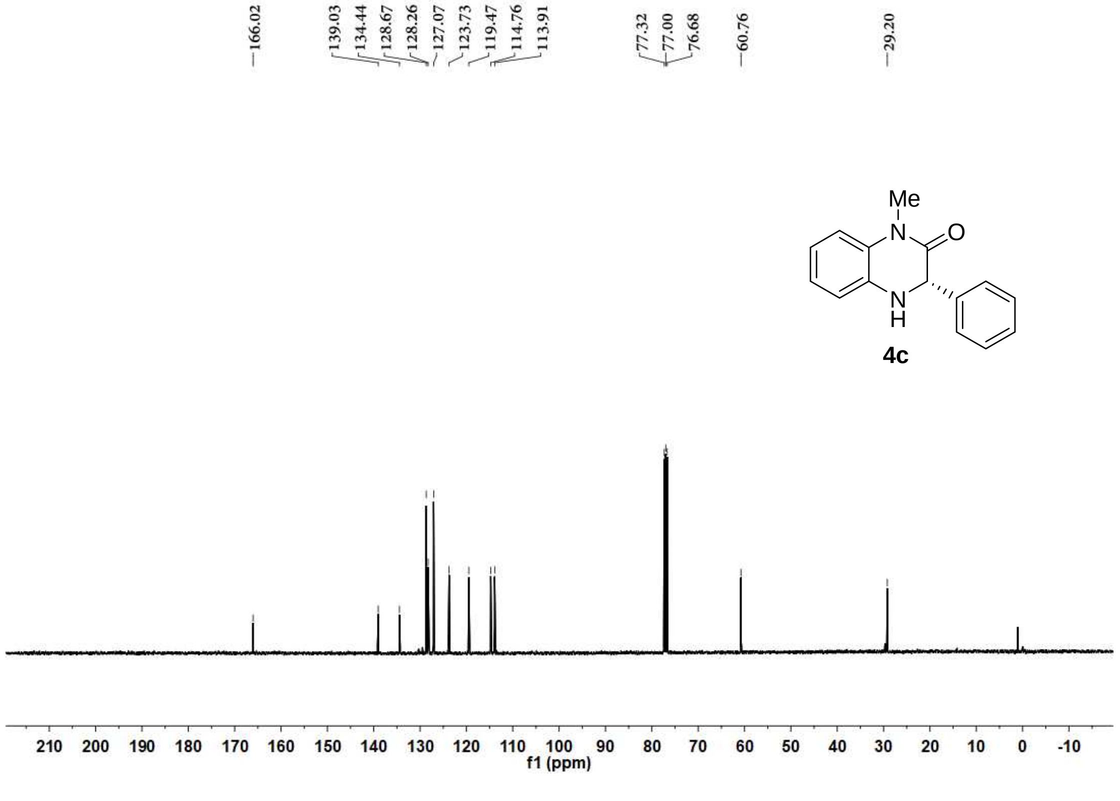
166.02
139.03
134.44
128.67
128.26
127.07
123.73
119.47
114.76
113.91
77.32
77.00
76.68
60.76
29.20
Me
N
O
N
H
4c
210 200 190 180 170 160 150 140 130 120 110 100 90 80 70 60 50 40 30 20 10 0 -10
f1 (ppm)

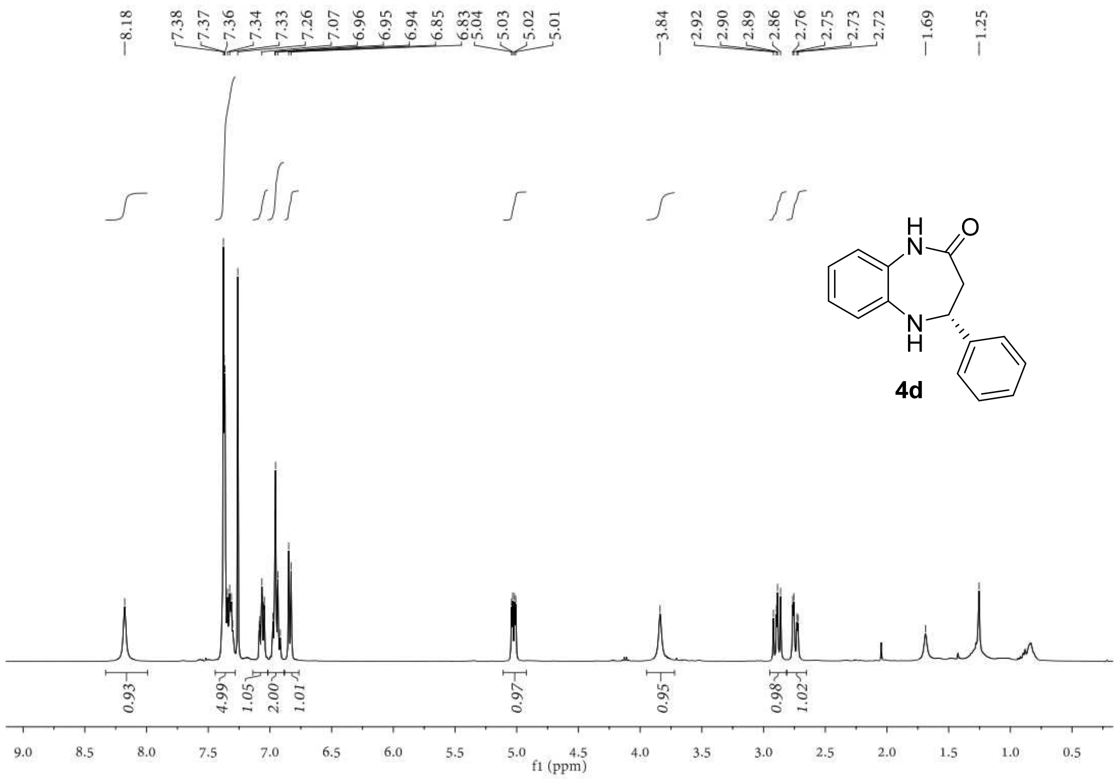
8.18
7.38
7.37
7.36
7.34
7.33
7.26
7.07
6.96
6.95
6.94
6.85
6.83
5.04
5.03
5.02
5.01
3.84
2.92
2.90
2.89
2.86
2.76
2.75
2.73
2.72
1.69
1.25
0.93
4.99
1.05
2.00
1.01
0.97
0.95
0.98
1.02
f1 (ppm)
4d

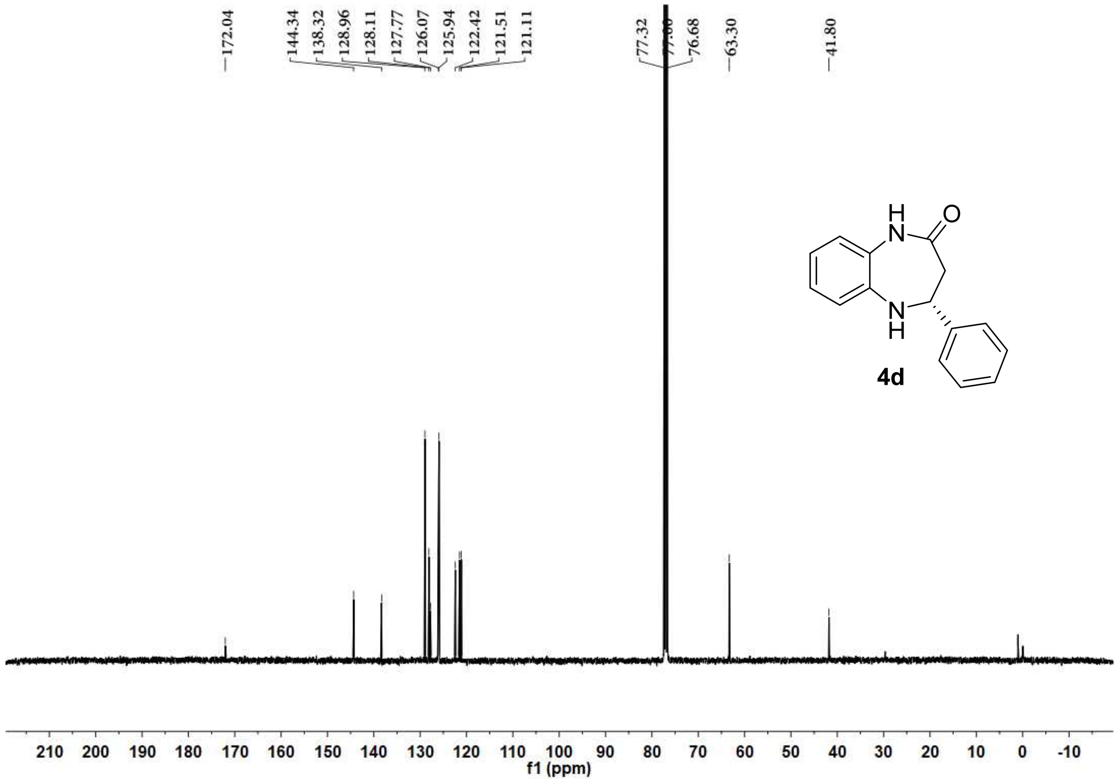
172.04
144.34
138.32
128.96
128.11
127.77
126.07
125.94
122.42
121.51
121.11
77.32
76.68
63.30
41.80
f1 (ppm)
4d

VII. HPLC Spectra

```
Data File E:\DATA\WSW\CX-2017-10-17\CX 2017-10-17 21-39-48\001-0501.D
Sample Name: HZY-STAND-RAC-PDC

    =====================================================================
    Acq. Operator   : SYSTEM                         Seq. Line :   5
    Acq. Instrument : 1260HPLC-DAD                    Location : Vial 1
    Injection Date  : 10/18/2017 12:04:44 AM               Inj :   1
                                                    Inj Volume : 1.000 µl
    Acq. Method     : E:\DATA\WSW\CX-2017-10-17\CX 2017-10-17 21-39-48\DAD-OD(1-2)-80-20-1ML-1UL-
                      25MIN.M
    Last changed    : 10/17/2017 9:41:26 PM by SYSTEM
    Analysis Method : E:\DATA\WSW\CX-2017-10-17\CX 2017-10-17 21-39-48\DAD-OD(1-2)-80-20-1ML-1UL-
                      25MIN.M (Sequence Method)
    Last changed    : 10/18/2017 2:29:25 PM by SYSTEM
                      (modified after loading)
    Additional Info : Peak(s) manually integrated
```

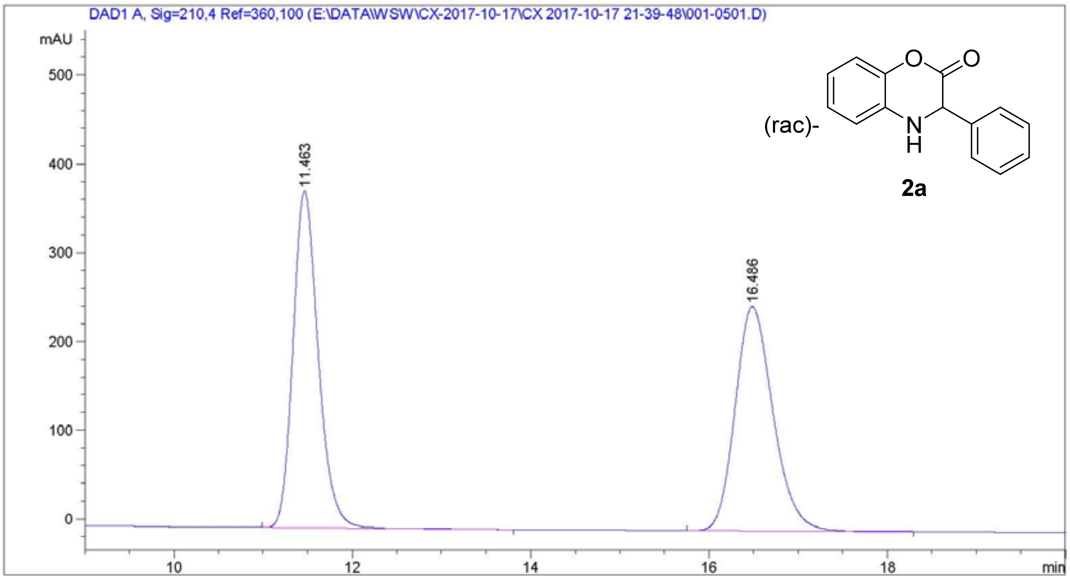


```
    =====================================================================
                             Area Percent Report
    =====================================================================

    Sorted By             :      Signal
    Multiplier            :      1.0000
    Dilution              :      1.0000
    Do not use Multiplier & Dilution Factor with ISTDs


    Signal 1: DAD1 A, Sig=210,4 Ref=360,100

    Peak RetTime Type  Width     Area      Height     Area
      #   [min]        [min]   [mAU*s]     [mAU]        %
    ----|-------|----|-------|----------|----------|--------|
       1  11.463 BB    0.3012 7456.20361  379.57709  49.9735
       2  16.486 BB    0.4543 7464.11328  252.91653  50.0265

    Totals :                  1.49203e4  632.49362
```

Data File E:\DATA\LXX\Y-COOET\LXX-4-168 2017-08-25 14-55-13\021-0401.D
Sample Name: HZY-N-ME-L

===

Acq. Operator : SYSTEM Seq. Line : 4
Acq. Instrument : 1260HPLC-DAD Location : Vial 21
Injection Date : 8/25/2017 4:19:18 PM Inj : 1
Inj Volume : 1.000 µl
Acq. Method : E:\DATA\LXX\Y-COOET\LXX-4-168 2017-08-25 14-55-13\DAD-OD(1-2)-80-20-1ML-1UL-25MIN.M
Last changed : 8/25/2017 3:16:42 PM by SYSTEM
Analysis Method : E:\DATA\LXX\Y-COOET\LXX-4-168 2017-08-25 14-55-13\DAD-OD(1-2)-80-20-1ML-1UL-25MIN.M (Sequence Method)
Last changed : 8/25/2017 6:31:11 PM by SYSTEM
(modified after loading)
Additional Info : Peak(s) manually integrated

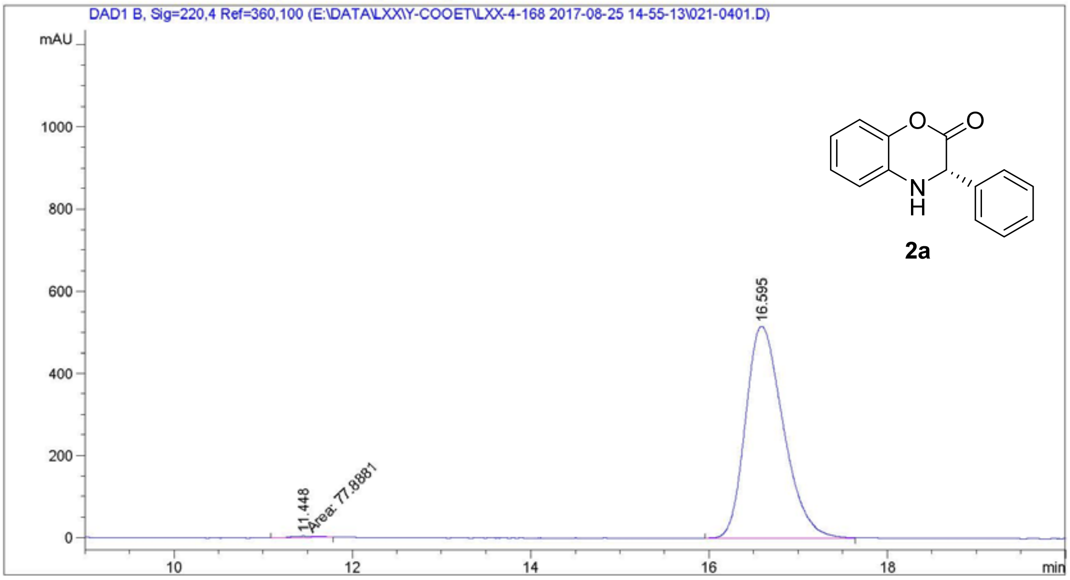

===

Area Percent Report

===

Sorted By : Signal
Multiplier : 1.0000
Dilution : 1.0000
Do not use Multiplier & Dilution Factor with ISTDs

Signal 1: DAD1 B, Sig=220,4 Ref=360,100

Peak #	RetTime [min]	Type	Width [min]	Area [mAU*s]	Height [mAU]	Area %
1	11.448	MM	0.3217	77.88808	4.03542	0.5097
2	16.595	BV	0.4370	1.52034e4	517.14484	99.4903

Totals : 1.52813e4 521.18026

Data File D:\DATA\HZY\AMINE\20190220 2019-02-20 12-06-00\013-0501.D
Sample Name: Me-PdC

===
Acq. Operator : Seq. Line : 5
Acq. Instrument : Instrument 2 Location : Vial 13
Injection Date : 2/20/2019 1:31:27 PM Inj : 1
Inj Volume : 1.000 µl
Acq. Method : D:\DATA\HZY\AMINE\20190220 2019-02-20 12-06-00\DAD-OD(1-2)-80-20-1ML-1UL-ALL-30MIN.M
Last changed : 12/15/2018 5:07:00 PM
Analysis Method : D:\METHOD\HZY\DAD-OJ(1-6)-80-20-1ML-1UL-ALL-50MIN.M
Last changed : 2/20/2019 3:31:40 PM
(modified after loading)
Additional Info : Peak(s) manually integrated

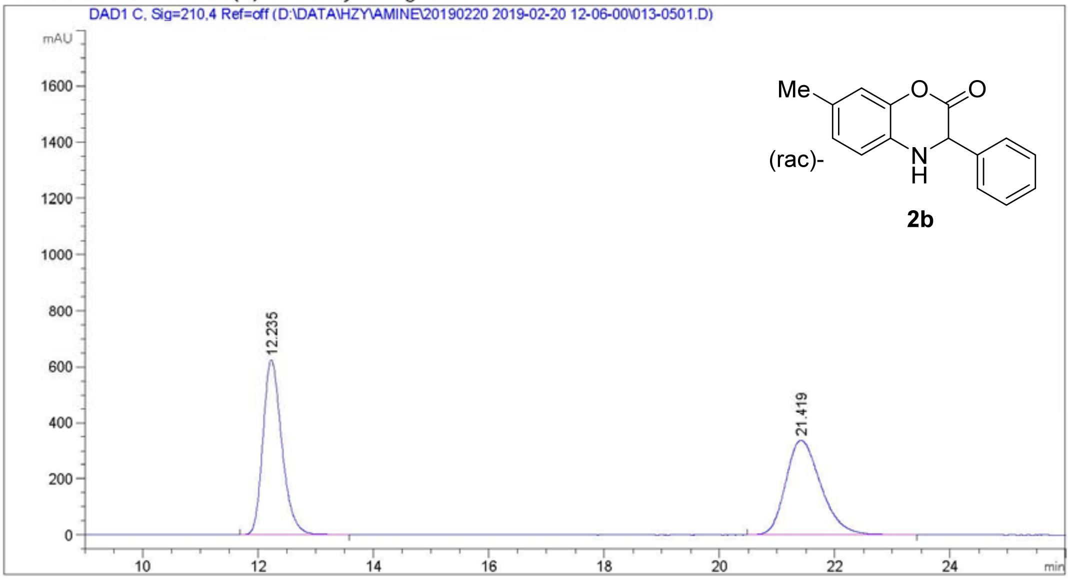

2b

===
Area Percent Report
===

Sorted By : Signal
Multiplier : 1.0000
Dilution : 1.0000
Use Multiplier & Dilution Factor with ISTDs

Signal 1: DAD1 C, Sig=210,4 Ref=off

Peak #	RetTime [min]	Type	Width [min]	Area [mAU*s]	Height [mAU]	Area %
1	12.235	BB	0.3519	1.42514e4	625.18842	49.8896
2	21.419	BB	0.6407	1.43144e4	337.58340	50.1104

Totals : 2.85658e4 962.77182

Data File E:\DATA\HZY\AMINE\ZHAOPHOS-171102 2017-11-02 10-46-55\042-0301.D
Sample Name: H-2

===

Acq. Operator : SYSTEM Seq. Line : 3
Acq. Instrument : 1260HPLC-DAD Location : Vial 42
Injection Date : 11/2/2017 11:24:42 AM Inj : 1
Inj Volume : 1.000 µl
Acq. Method : E:\DATA\HZY\AMINE\ZHAOPHOS-171102 2017-11-02 10-46-55\DAD-OD(1-2)-80-20-1ML-1UL-25MIN.M
Last changed : 11/2/2017 10:46:57 AM by SYSTEM
Analysis Method : E:\DATA\HZY\AMINE\ZHAOPHOS-171102 2017-11-02 10-46-55\DAD-OD(1-2)-80-20-1ML-1UL-25MIN.M (Sequence Method)
Last changed : 11/2/2017 2:58:04 PM by SYSTEM
(modified after loading)
Additional Info : Peak(s) manually integrated

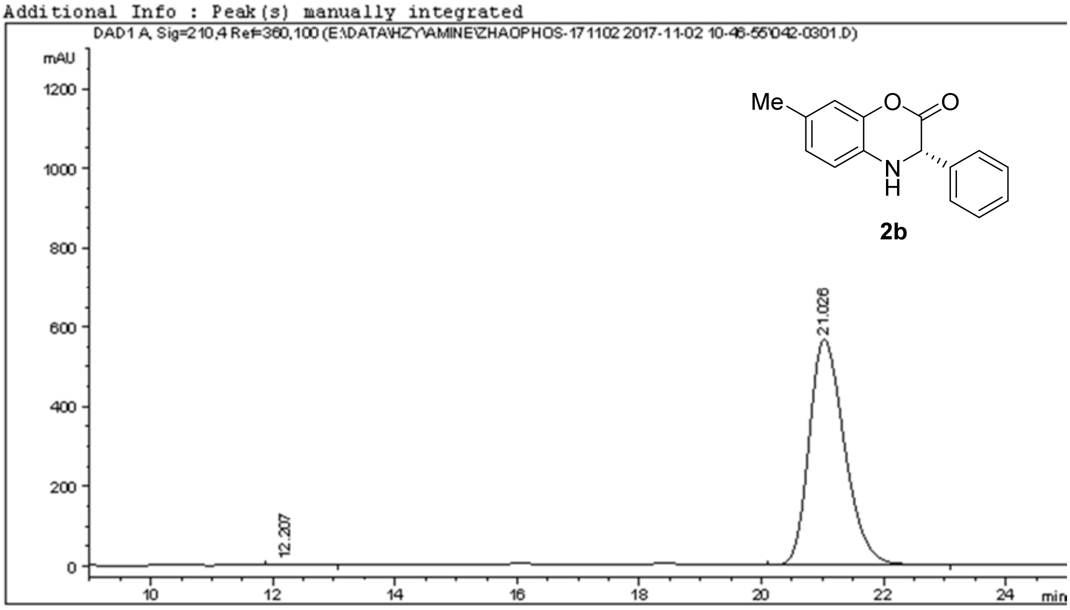

===

Area Percent Report

===

Sorted By : Signal
Multiplier : 1.0000
Dilution : 1.0000
Do not use Multiplier & Dilution Factor with ISTDs

Signal 1: DAD1 A, Sig=210,4 Ref=360,100

Peak #	RetTime [min]	Type	Width [min]	Area [mAU*s]	Height [mAU]	Area %
1	12.207	BB	0.2839	63.06870	2.72591	0.2773
2	21.026	BB	0.6202	2.26773e4	565.12823	99.7227

Totals : 2.27403e4 567.85415

===

*** End of Report ***

1260HPLC-DAD 11/2/2017 2:58:11 PM SYSTEM Page 1 of 1

Data File E:\DATA\HZY\AMINE\20170817-PDC 2017-08-17 11-28-10\032-0501.D
Sample Name: pdc-2

===

Acq. Operator : SYSTEM Seq. Line : 5
Acq. Instrument : 1260HPLC-DAD Location : Vial 32
Injection Date : 8/17/2017 12:58:00 PM Inj : 1
Inj Volume : 1.000 µl
Acq. Method : E:\DATA\HZY\AMINE\20170817-PDC 2017-08-17 11-28-10\DAD-OD(1-2)-80-20-1ML-1UL-25MIN.M
Last changed : 8/17/2017 11:28:10 AM by SYSTEM
Analysis Method : E:\DATA\HZY\AMINE\20170817-PDC 2017-08-17 11-28-10\DAD-OD(1-2)-80-20-1ML-1UL-25MIN.M (Sequence Method)
Last changed : 8/17/2017 4:57:01 PM by SYSTEM
(modified after loading)
Additional Info : Peak(s) manually integrated

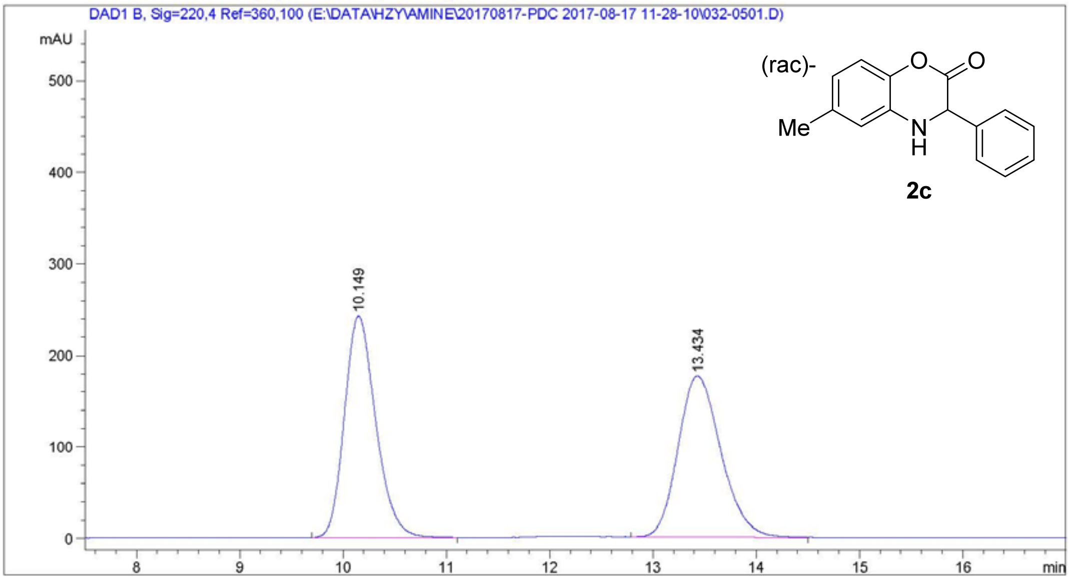

2c

===

Area Percent Report

===

Sorted By : Signal
Multiplier : 1.0000
Dilution : 1.0000
Do not use Multiplier & Dilution Factor with ISTDs

Signal 1: DAD1 B, Sig=220,4 Ref=360,100

Peak #	RetTime [min]	Type	Width [min]	Area [mAU*s]	Height [mAU]	Area %
1	10.149	VB	0.3154	5104.18604	242.61790	50.0894
2	13.434	BB	0.4222	5085.97412	176.48285	49.9106

Totals : 1.01902e4 419.10075

Data File E:\DATA\HZY\AMINE\ZHAOPHOS-171102 2017-11-02 10-46-55\041-0201.D
Sample Name: H-1

===

Acq. Operator : SYSTEM Seq. Line : 2
Acq. Instrument : 1260HPLC-DAD Location : Vial 41
Injection Date : 11/2/2017 10:58:48 AM Inj : 1
Inj Volume : 1.000 µl
Acq. Method : E:\DATA\HZY\AMINE\ZHAOPHOS-171102 2017-11-02 10-46-55\DAD-OD(1-2)-80-20-1ML-1UL-25MIN.M
Last changed : 11/2/2017 10:46:57 AM by SYSTEM
Analysis Method : E:\DATA\HZY\AMINE\ZHAOPHOS-171102 2017-11-02 10-46-55\DAD-OD(1-2)-80-20-1ML-1UL-25MIN.M (Sequence Method)
Last changed : 11/2/2017 2:54:49 PM by SYSTEM
(modified after loading)
Additional Info : Peak(s) manually integrated

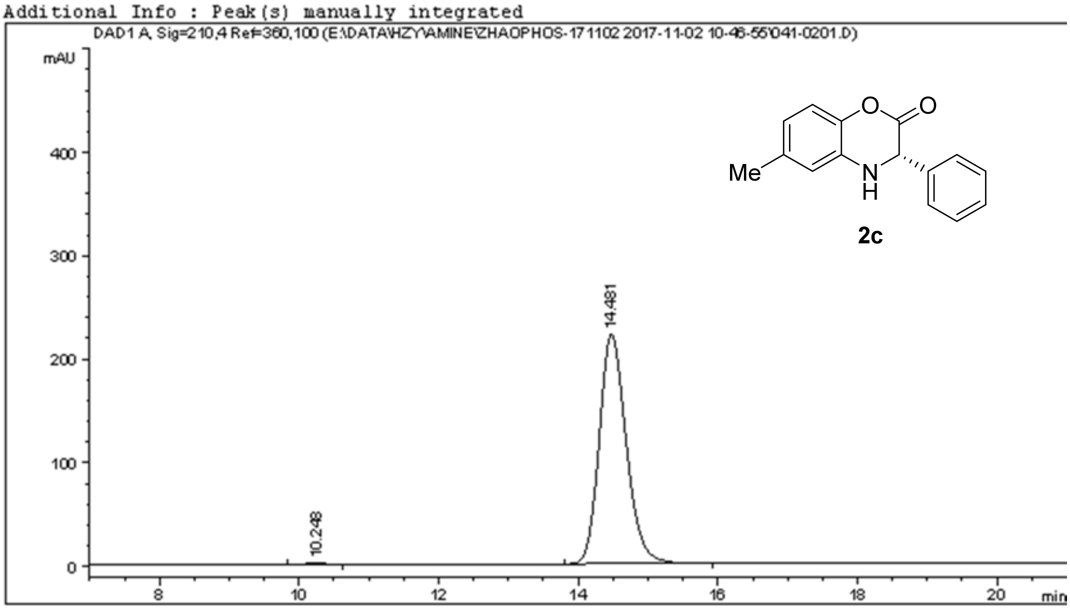

===

Area Percent Report

===

Sorted By : Signal
Multiplier : 1.0000
Dilution : 1.0000
Do not use Multiplier & Dilution Factor with ISTDs

Signal 1: DAD1 A, Sig=210,4 Ref=360,100

Peak #	RetTime [min]	Type	Width [min]	Area [mAU*s]	Height [mAU]	Area %
1	10.248	BB	0.2408	24.53086	1.25108	0.4111
2	14.481	BB	0.4152	5942.91553	221.38681	99.5889

Totals : 5967.44639 222.63789

===

*** End of Report ***

1260HPLC-DAD 11/2/2017 2:55:11 PM SYSTEM Page 1 of 1

Data File D:\DATA\HZY\AMINE\20190220 2019-02-20 12-06-00\015-0701.D
Sample Name: F-PdC

===

Acq. Operator   :   Seq. Line :   7
Acq. Instrument : Instrument 2   Location : Vial 15
Injection Date : 2/20/2019 2:33:18 PM   Inj :   1
                                                 Inj Volume : 1.000 µl
Acq. Method   : D:\DATA\HZY\AMINE\20190220 2019-02-20 12-06-00\DAD-OD(1-2)-80-20-1ML-1UL-ALL-30MIN.M
Last changed   : 12/15/2018 5:07:00 PM
Analysis Method : D:\METHOD\HZY\DAD-OJ(1-6)-80-20-1ML-1UL-ALL-50MIN.M
Last changed   : 2/20/2019 3:33:28 PM
                  (modified after loading)
Additional Info : Peak(s) manually integrated

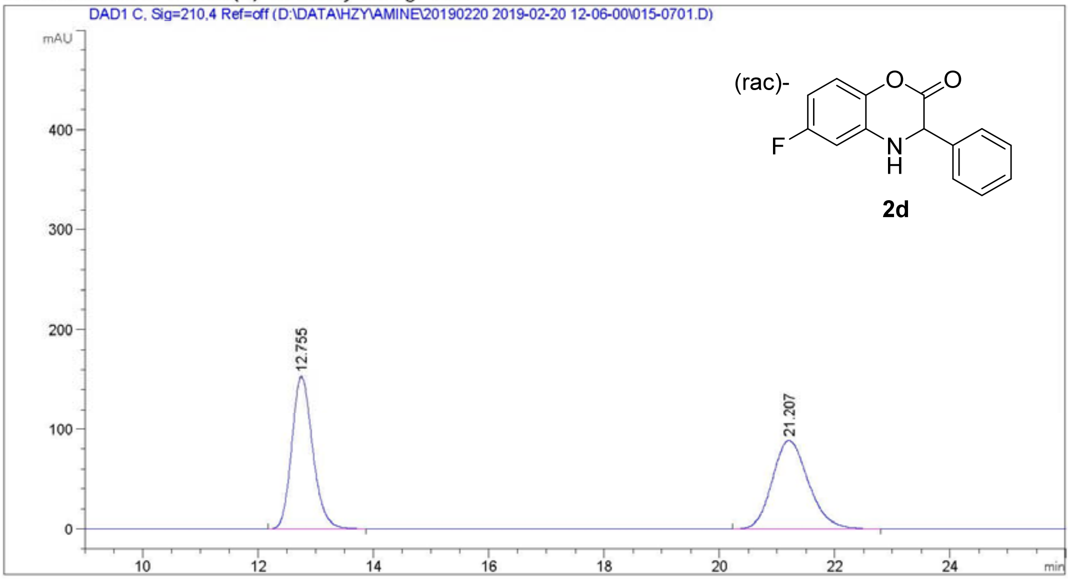

===

Area Percent Report

===

Sorted By : Signal
Multiplier : 1.0000
Dilution : 1.0000
Use Multiplier & Dilution Factor with ISTDs

Signal 1: DAD1 C, Sig=210,4 Ref=off

Peak #	RetTime [min]	Type	Width [min]	Area [mAU*s]	Height [mAU]	Area %
1	12.755	BB	0.3867	3840.82959	153.07249	49.9734
2	21.207	BB	0.6508	3844.91992	88.51109	50.0266

Totals : 7685.74951 241.58359

Data File E:\DATA\HZY\AMINE\ZHAOPHOS-171102 2017-11-02 10-46-55\043-0401.D
Sample Name: H-4

===

Acq. Operator : SYSTEM Seq. Line : 4
Acq. Instrument : 1260HPLC-DAD Location : Vial 43
Injection Date : 11/2/2017 11:50:35 AM Inj : 1
Inj Volume : 1.000 µl
Acq. Method : E:\DATA\HZY\AMINE\ZHAOPHOS-171102 2017-11-02 10-46-55\DAD-OD(1-2)-80-20-1ML-1UL-25MIN.M
Last changed : 11/2/2017 10:46:57 AM by SYSTEM
Analysis Method : E:\DATA\HZY\AMINE\ZHAOPHOS-171102 2017-11-02 10-46-55\DAD-OD(1-2)-80-20-1ML-1UL-25MIN.M (Sequence Method)
Last changed : 11/2/2017 3:00:28 PM by SYSTEM
(modified after loading)
Additional Info : Peak(s) manually integrated

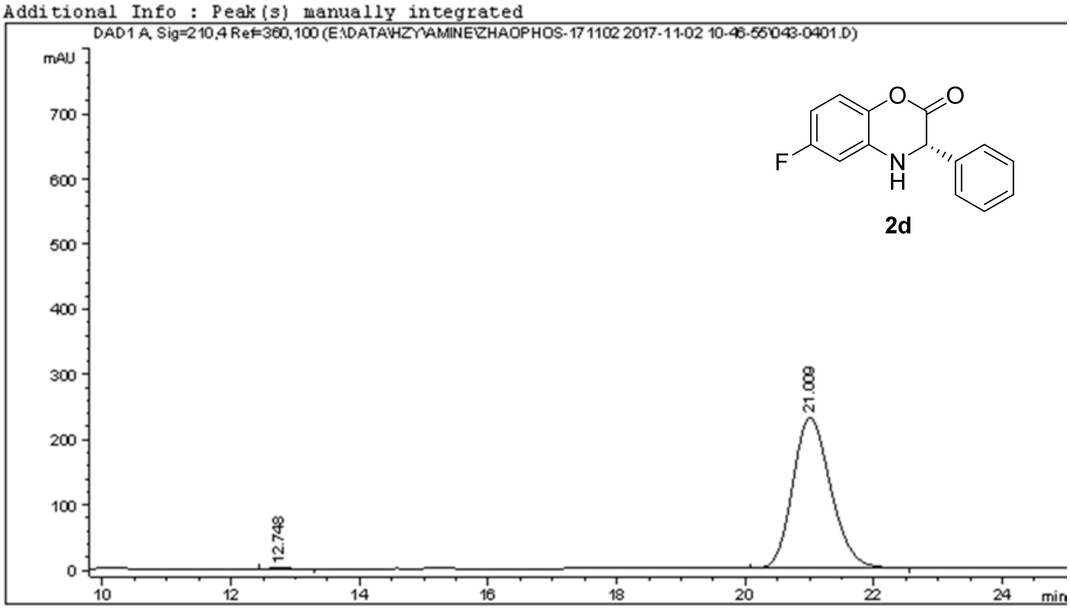

===

Area Percent Report

===

Sorted By : Signal
Multiplier : 1.0000
Dilution : 1.0000
Do not use Multiplier & Dilution Factor with ISTDs

Signal 1: DAD1 A, Sig=210,4 Ref=360,100

Peak #	RetTime [min]	Type	Width [min]	Area [mAU*s]	Height [mAU]	Area %
1	12.748	BB	0.2726	30.38393	1.38122	0.3200
2	21.009	BB	0.6325	9465.70020	230.79195	99.6800

Totals : 9496.08413 232.17317

===

*** End of Report ***

1260HPLC-DAD 11/2/2017 3:00:33 PM SYSTEM Page 1 of 1

Data File E:\DATA\HZY\AMINE\SUB-20170926 2017-09-26 11-16-47\082-1401.D
Sample Name: H-2-RAC

===

Acq. Operator : SYSTEM Seq. Line : 14
Acq. Instrument : 1260HPLC-VWD Location : Vial 82
Injection Date : 9/26/2017 6:16:00 PM Inj : 1
Inj Volume : 5.000 µl
Acq. Method : E:\DATA\HZY\AMINE\SUB-20170926 2017-09-26 11-16-47\VWD-AD(1-6)-90-10-1ML-5UL-220NM-45MIN.M
Last changed : 9/26/2017 6:14:38 PM by SYSTEM
Analysis Method : E:\DATA\HZY\AMINE\SUB-20170926 2017-09-26 11-16-47\VWD-AD(1-6)-90-10-1ML-5UL-220NM-45MIN.M (Sequence Method)
Last changed : 9/26/2017 7:38:49 PM by SYSTEM
(modified after loading)
Additional Info : Peak(s) manually integrated

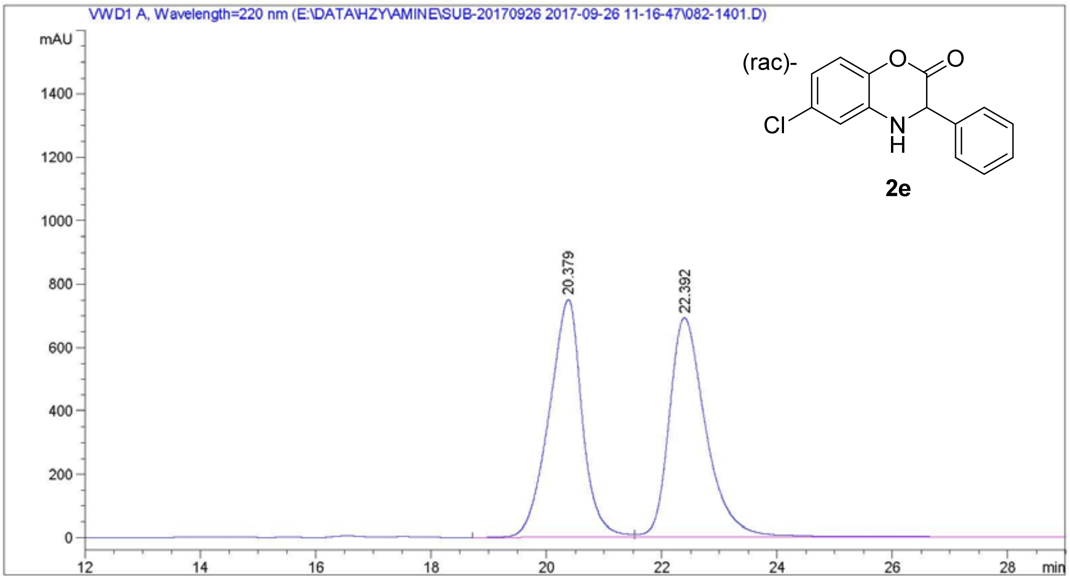

===
Area Percent Report
===

Sorted By : Signal
Multiplier : 1.0000
Dilution : 1.0000
Use Multiplier & Dilution Factor with ISTDs

Signal 1: VWD1 A, Wavelength=220 nm

Peak #	RetTime [min]	Type	Width [min]	Area [mAU*s]	Height [mAU]	Area %
1	20.379	BV	0.5892	2.98932e4	751.27502	49.1149
2	22.392	VB	0.6747	3.09706e4	693.93481	50.8851

Totals : 6.08639e4 1445.20984

Data File C:\CHEM32\1\DATA\SNAPSHOT.D
Sample Name:

===

Acq. Operator : SYSTEM Seq. Line : 13
Location : Vial 72
Injection Date : 9/26/2017 5:40:14 PM Inj : 1
Acq. Method : VWD-AD(1-6)-90-10-1ML-5UL-220NM-45MIN.M
Analysis Method : E:\DATA\HZY\AMINE\CL-PH-RAC170924 2017-09-24 18-48-03\VWD-AD(1-6)-90-10-1ML-2UL-210NM-60MIN.M (Sequence Method)
Last changed : 9/26/2017 6:12:38 PM by SYSTEM
(modified after loading)
Additional Info : Peak(s) manually integrated

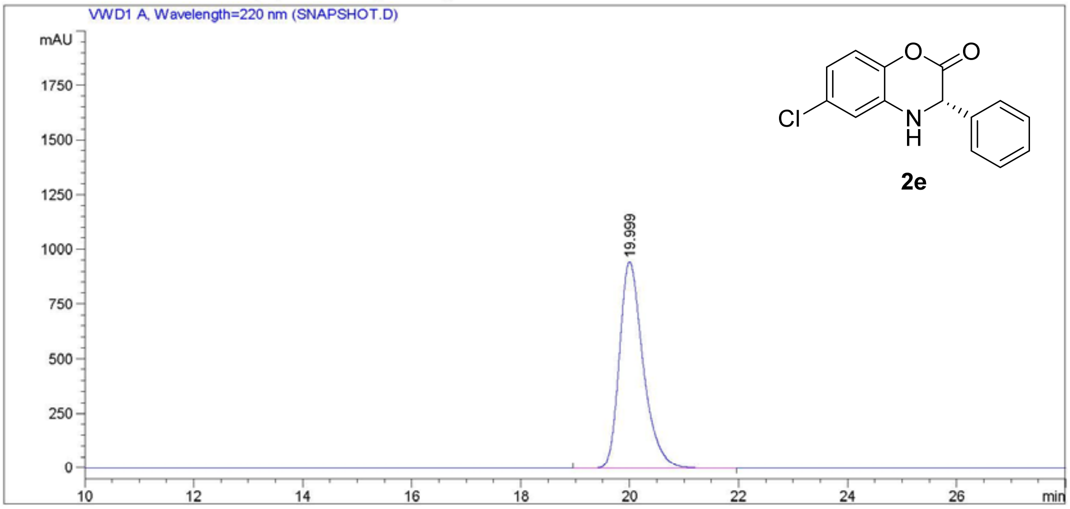

===

Area Percent Report

===

Sorted By : Signal
Multiplier : 1.0000
Dilution : 1.0000
Do not use Multiplier & Dilution Factor with ISTDs

Signal 1: VWD1 A, Wavelength=220 nm

Peak #	RetTime [min]	Type	Width [min]	Area [mAU*s]	Height [mAU]	Area %
1	19.999	BB	0.4579	2.84939e4	944.78058	100.0000

Totals : 2.84939e4 944.78058

===

*** End of Report ***

1260HPLC-VWD 9/26/2017 6:12:51 PM SYSTEM Page 1 of 1

Data File E:\DATA\HZY\AMINE\SUB-20170926 2017-09-26 11-16-47\085-0901.D
Sample Name: H-5-RAC

===

Acq. Operator : SYSTEM Seq. Line : 9
Acq. Instrument : 1260HPLC-VWD Location : Vial 85
Injection Date : 9/26/2017 3:12:00 PM Inj : 1
Inj Volume : 3.000 µl
Acq. Method : E:\DATA\HZY\AMINE\SUB-20170926 2017-09-26 11-16-47\VWD-OD(1-2)-80-20-1ML-3UL-220NM-45MIN.M
Last changed : 9/26/2017 11:46:03 AM by SYSTEM
Analysis Method : E:\DATA\HZY\AMINE\SUB-20170926 2017-09-26 11-16-47\VWD-OD(1-2)-80-20-1ML-3UL-220NM-45MIN.M (Sequence Method)
Last changed : 9/26/2017 4:08:27 PM by SYSTEM
(modified after loading)
Additional Info : Peak(s) manually integrated

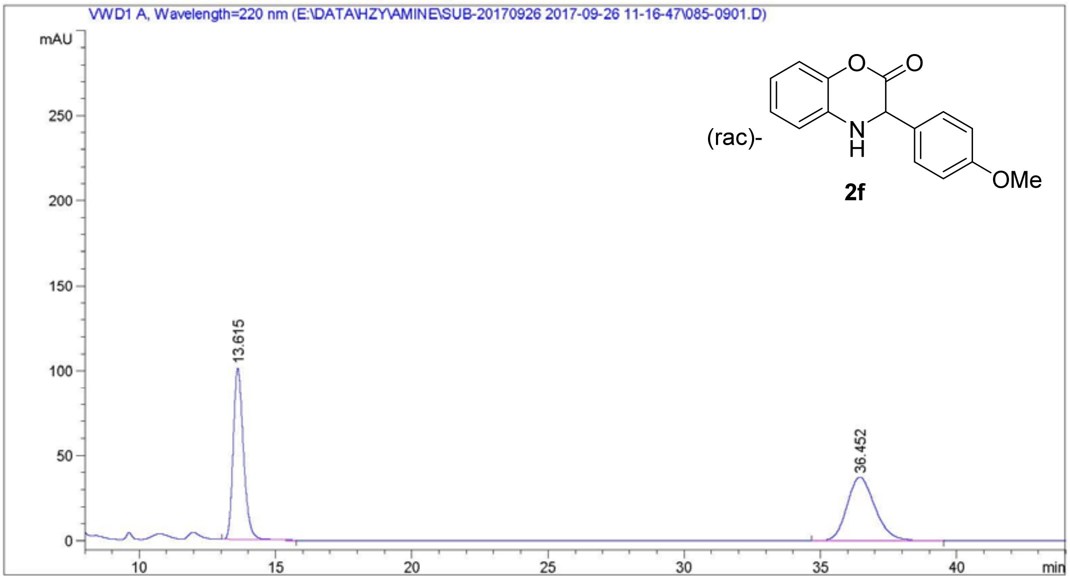

===

Area Percent Report

===

Sorted By : Signal
Multiplier : 1.0000
Dilution : 1.0000
Use Multiplier & Dilution Factor with ISTDs

Signal 1: VWD1 A, Wavelength=220 nm

Peak #	RetTime [min]	Type	Width [min]	Area [mAU*s]	Height [mAU]	Area %
1	13.615	BB	0.3988	2638.70752	101.32419	49.8710
2	36.452	BB	1.0877	2652.35864	37.29427	50.1290

Totals : 5291.06616 138.61846

Data File E:\DATA\HZY\AMINE\SUB-20170926 2017-09-26 11-16-47\075-0801.D
Sample Name: H-5-EE

===

Acq. Operator : SYSTEM Seq. Line : 8
Acq. Instrument : 1260HPLC-VWD Location : Vial 75
Injection Date : 9/26/2017 2:26:13 PM Inj : 1
Inj Volume : 3.000 µl
Acq. Method : E:\DATA\HZY\AMINE\SUB-20170926 2017-09-26 11-16-47\VWD-OD(1-2)-80-20-1ML-3UL-220NM-45MIN.M
Last changed : 9/26/2017 11:46:03 AM by SYSTEM
Analysis Method : E:\DATA\HZY\AMINE\SUB-20170926 2017-09-26 11-16-47\VWD-OD(1-2)-80-20-1ML-3UL-220NM-45MIN.M (Sequence Method)
Last changed : 9/26/2017 4:06:05 PM by SYSTEM
(modified after loading)
Additional Info : Peak(s) manually integrated

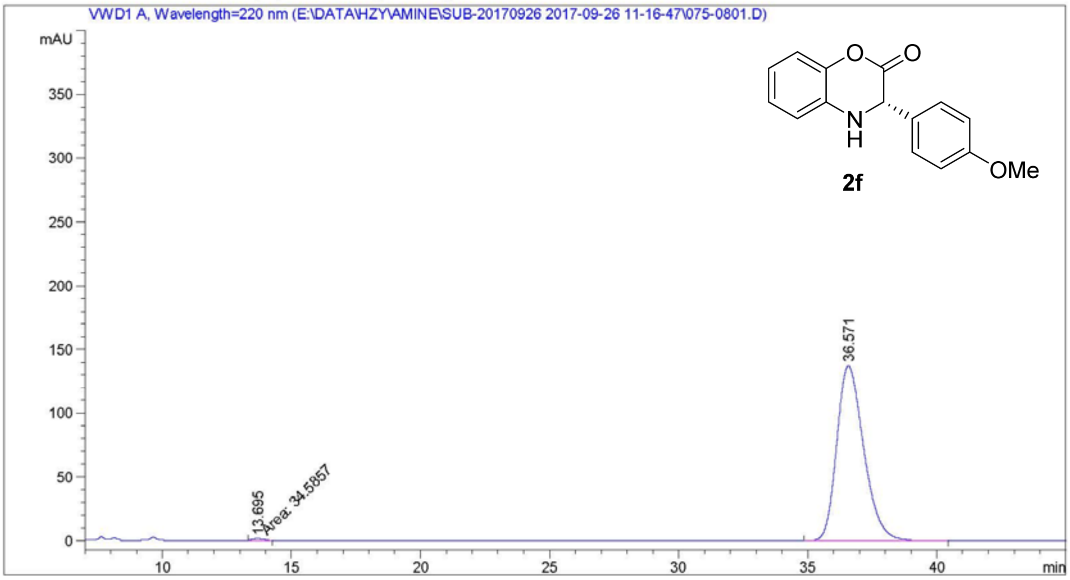

===

Area Percent Report

===

Sorted By : Signal
Multiplier : 1.0000
Dilution : 1.0000
Use Multiplier & Dilution Factor with ISTDs

Signal 1: VWD1 A, Wavelength=220 nm

Peak #	RetTime [min]	Type	Width [min]	Area [mAU*s]	Height [mAU]	Area %
1	13.695	MM	0.4144	34.58571	1.39101	0.3469
2	36.571	BB	1.1121	9935.48242	137.32356	99.6531

Totals : 9970.06813 138.71457

Data File E:\DATA\LXX\Y-COOET\LXX-4-168 2017-08-25 14-55-13\022-0501.D
Sample Name: HZY-PH-4F-RAC

===

Acq. Operator : SYSTEM Seq. Line : 5
Acq. Instrument : 1260HPLC-DAD Location : Vial 22
Injection Date : 8/25/2017 4:45:20 PM Inj : 1
Inj Volume : 1.000 µl
Acq. Method : E:\DATA\LXX\Y-COOET\LXX-4-168 2017-08-25 14-55-13\DAD-OD(1-2)-80-20-1ML-1UL-25MIN.M
Last changed : 8/25/2017 3:16:42 PM by SYSTEM
Analysis Method : E:\DATA\LXX\Y-COOET\LXX-4-168 2017-08-25 14-55-13\DAD-OD(1-2)-80-20-1ML-1UL-25MIN.M (Sequence Method)
Last changed : 8/25/2017 6:32:43 PM by SYSTEM
(modified after loading)
Additional Info : Peak(s) manually integrated

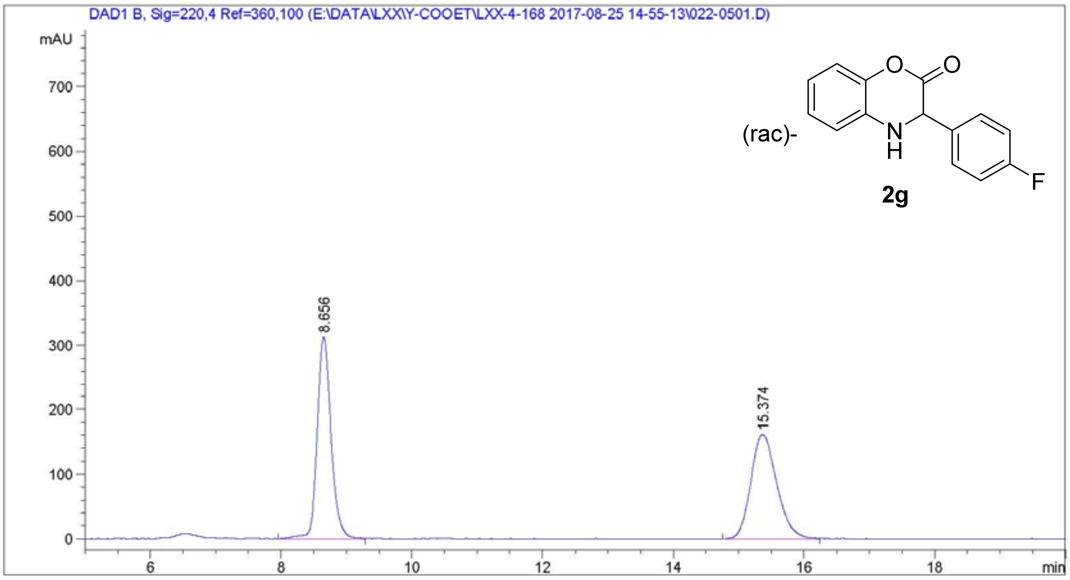

===

Area Percent Report

===

Sorted By : Signal
Multiplier : 1.0000
Dilution : 1.0000
Do not use Multiplier & Dilution Factor with ISTDs

Signal 1: DAD1 B, Sig=220,4 Ref=360,100

Peak #	RetTime [min]	Type	Width [min]	Area [mAU*s]	Height [mAU]	Area %
1	8.656	BV	0.2250	4591.43213	313.25784	50.8430
2	15.374	BV	0.4086	4439.17236	161.55267	49.1570

Totals : 9030.60449 474.81052

Data File E:\DATA\HZY\AMINE\PH-4FS 2017-08-26 10-27-06\023-0301.D
Sample Name: 4F-EE-REPEAT

===

Acq. Operator : SYSTEM Seq. Line : 3
Acq. Instrument : 1260HPLC-DAD Location : Vial 23
Injection Date : 8/26/2017 11:05:11 AM Inj : 1
Inj Volume : 1.000 µl
Acq. Method : E:\DATA\HZY\AMINE\PH-4FS 2017-08-26 10-27-06\DAD-OD(1-2)-80-20-1ML-1UL-25MIN.M
Last changed : 8/26/2017 11:04:04 AM by SYSTEM
Analysis Method : E:\DATA\HZY\AMINE\PH-4FS 2017-08-26 10-27-06\DAD-OD(1-2)-80-20-1ML-1UL-25MIN.M (Sequence Method)
Last changed : 8/26/2017 11:32:59 AM by SYSTEM
(modified after loading)
Additional Info : Peak(s) manually integrated

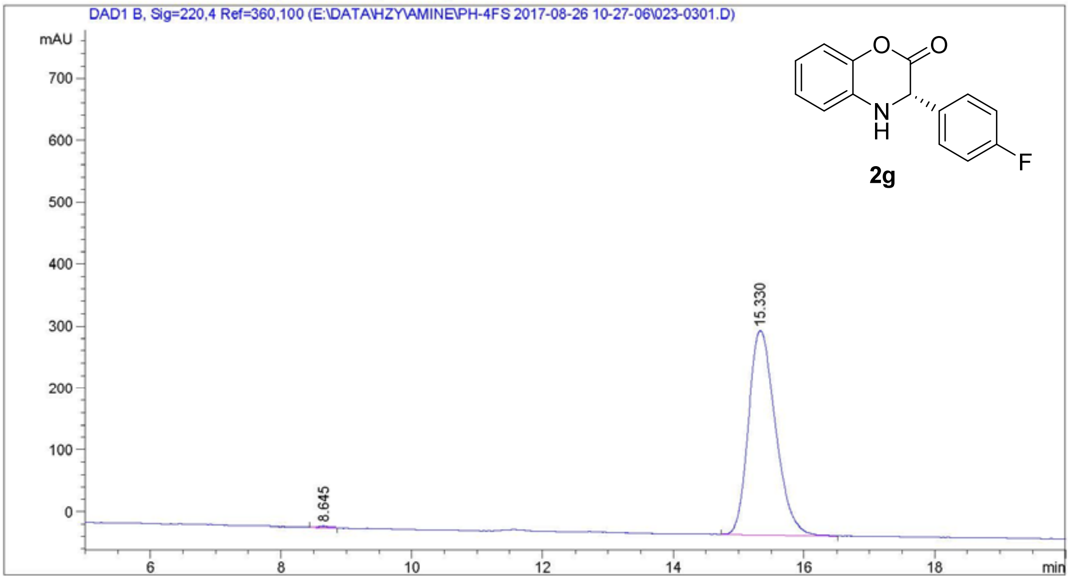

===

Area Percent Report

===

Sorted By : Signal
Multiplier : 1.0000
Dilution : 1.0000
Do not use Multiplier & Dilution Factor with ISTDs

Signal 1: DAD1 B, Sig=220,4 Ref=360,100

Peak #	RetTime [min]	Type	Width [min]	Area [mAU*s]	Height [mAU]	Area %
1	8.645	BV	0.1455	23.99336	2.09691	0.2529
2	15.330	VV	0.4379	9462.84766	330.66409	99.7471

Totals : 9486.84101 332.76100

Data File E:\DATA\HZY\AMINE\SUB-20170926 2017-09-26 11-16-47\086-1101.D
Sample Name: H-6-RAC

===

Acq. Operator : SYSTEM Seq. Line : 11
Acq. Instrument : 1260HPLC-VWD Location : Vial 86
Injection Date : 9/26/2017 4:43:36 PM Inj : 1
Inj Volume : 3.000 µl
Acq. Method : E:\DATA\HZY\AMINE\SUB-20170926 2017-09-26 11-16-47\VWD-OD(1-2)-80-20-1ML-3UL-220NM-45MIN.M
Last changed : 9/26/2017 11:46:03 AM by SYSTEM
Analysis Method : E:\DATA\HZY\AMINE\SUB-20170926 2017-09-26 11-16-47\VWD-OD(1-2)-80-20-1ML-3UL-220NM-45MIN.M (Sequence Method)
Last changed : 9/26/2017 6:09:23 PM by SYSTEM
(modified after loading)
Additional Info : Peak(s) manually integrated

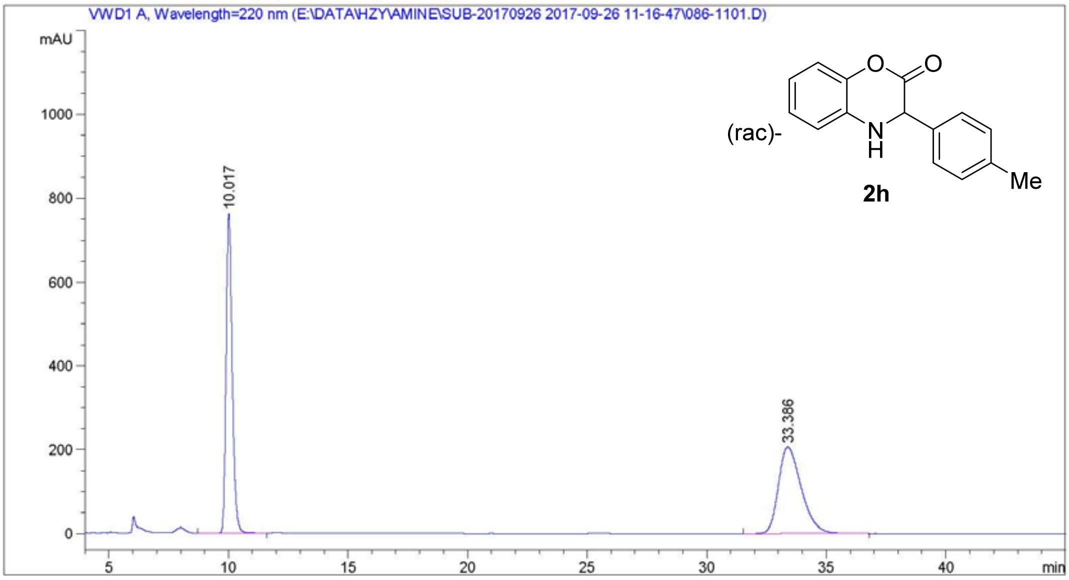

===

Area Percent Report

===

Sorted By : Signal
Multiplier : 1.0000
Dilution : 1.0000
Use Multiplier & Dilution Factor with ISTDs

Signal 1: VWD1 A, Wavelength=220 nm

Peak #	RetTime [min]	Type	Width [min]	Area [mAU*s]	Height [mAU]	Area %
1	10.017	BB	0.2737	1.35925e4	763.52167	49.8999
2	33.386	BB	1.0173	1.36470e4	205.88000	50.1001

Totals : 2.72395e4 969.40167

Data File E:\DATA\HZY\AMINE\SUB-20170926 2017-09-26 11-16-47\076-1001.D
Sample Name: H-6-EE

===

Acq. Operator : SYSTEM Seq. Line : 10
Acq. Instrument : 1260HPLC-VWD Location : Vial 76
Injection Date : 9/26/2017 3:57:50 PM Inj : 1
Inj Volume : 3.000 µl
Acq. Method : E:\DATA\HZY\AMINE\SUB-20170926 2017-09-26 11-16-47\VWD-OD(1-2)-80-20-1ML-3UL-220NM-45MIN.M
Last changed : 9/26/2017 11:46:03 AM by SYSTEM
Analysis Method : E:\DATA\HZY\AMINE\SUB-20170926 2017-09-26 11-16-47\VWD-OD(1-2)-80-20-1ML-3UL-220NM-45MIN.M (Sequence Method)
Last changed : 9/26/2017 5:16:58 PM by SYSTEM
(modified after loading)
Additional Info : Peak(s) manually integrated

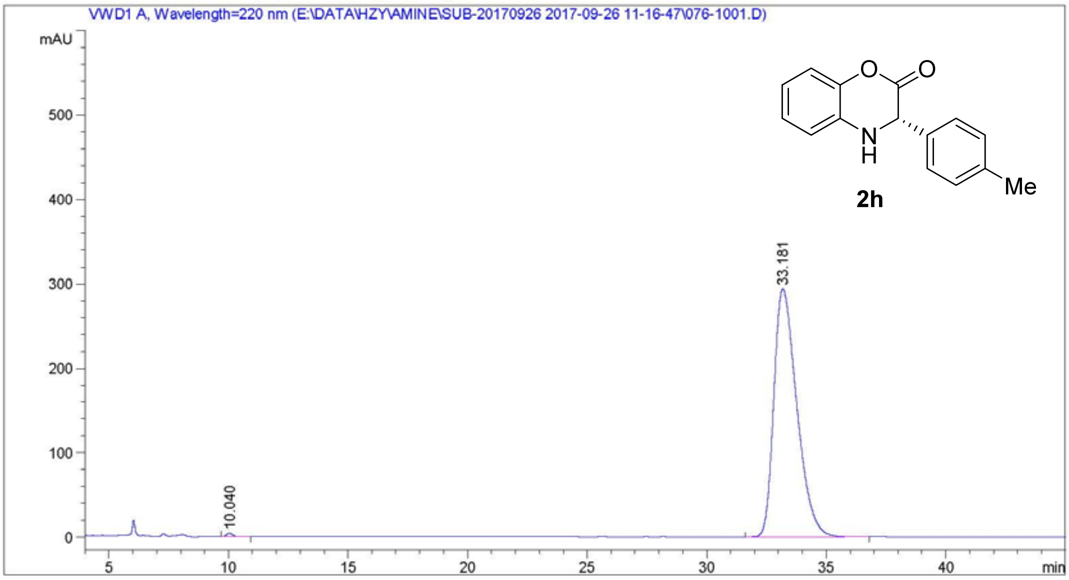

===

Area Percent Report

===

Sorted By : Signal
Multiplier : 1.0000
Dilution : 1.0000
Use Multiplier & Dilution Factor with ISTDs

Signal 1: VWD1 A, Wavelength=220 nm

Peak #	RetTime [min]	Type	Width [min]	Area [mAU*s]	Height [mAU]	Area %
1	10.040	BB	0.2714	71.31303	4.03077	0.3616
2	33.181	BB	1.0232	1.96487e4	293.80399	99.6384

Totals :	1.97200e4	297.83475

Data File E:\DATA\HZY\AMINE\SUB-20170926 2017-09-26 11-16-47\084-0701.D
Sample Name: H-4-RAC

===

Acq. Operator : SYSTEM Seq. Line : 7
Acq. Instrument : 1260HPLC-VWD Location : Vial 84
Injection Date : 9/26/2017 2:00:20 PM Inj : 1
Inj Volume : 2.000 µl
Acq. Method : E:\DATA\HZY\AMINE\SUB-20170926 2017-09-26 11-16-47\VWD-OD(1-2)-80-20-1ML-2UL-220NM-25MIN.M
Last changed : 9/26/2017 11:44:47 AM by SYSTEM
Analysis Method : E:\DATA\HZY\AMINE\SUB-20170926 2017-09-26 11-16-47\VWD-OD(1-2)-80-20-1ML-2UL-220NM-25MIN.M (Sequence Method)
Last changed : 9/26/2017 2:48:41 PM by SYSTEM
(modified after loading)
Additional Info : Peak(s) manually integrated

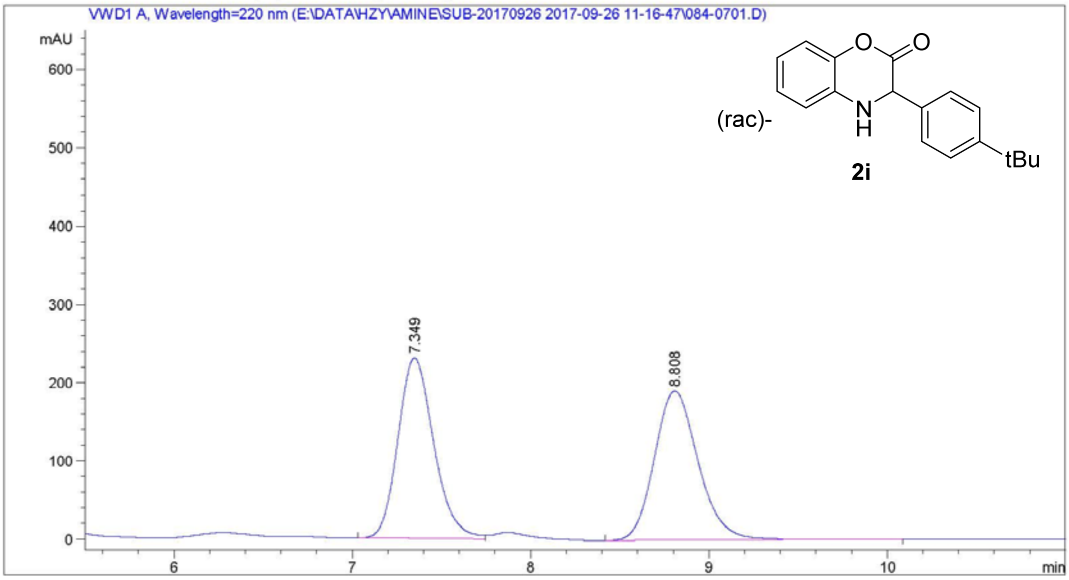

===

Area Percent Report

===

Sorted By : Signal
Multiplier : 1.0000
Dilution : 1.0000
Use Multiplier & Dilution Factor with ISTDs

Signal 1: VWD1 A, Wavelength=220 nm

Peak #	RetTime [min]	Type	Width [min]	Area [mAU*s]	Height [mAU]	Area %
1	7.349	BV	0.2148	3224.69531	231.08284	49.8026
2	8.808	BB	0.2624	3250.25928	190.18265	50.1974

Totals : 6474.95459 421.26549

Data File E:\DATA\HZY\AMINE\SUB-20170926 2017-09-26 11-16-47\074-0601.D
Sample Name: H-4-EE

```
=====================================================================
Acq. Operator   : SYSTEM                          Seq. Line :    6
Acq. Instrument : 1260HPLC-VWD                     Location : Vial 74
Injection Date  : 9/26/2017 1:34:34 PM                  Inj :    1
                                                 Inj Volume : 2.000 µl
Acq. Method     : E:\DATA\HZY\AMINE\SUB-20170926 2017-09-26 11-16-47\VWD-OD(1-2)-80-20-1ML-
                  2UL-220NM-25MIN.M
Last changed    : 9/26/2017 11:44:47 AM by SYSTEM
Analysis Method : E:\DATA\HZY\AMINE\SUB-20170926 2017-09-26 11-16-47\VWD-OD(1-2)-80-20-1ML-
                  2UL-220NM-25MIN.M (Sequence Method)
Last changed    : 9/26/2017 2:49:32 PM by SYSTEM
                  (modified after loading)
Additional Info : Peak(s) manually integrated
```

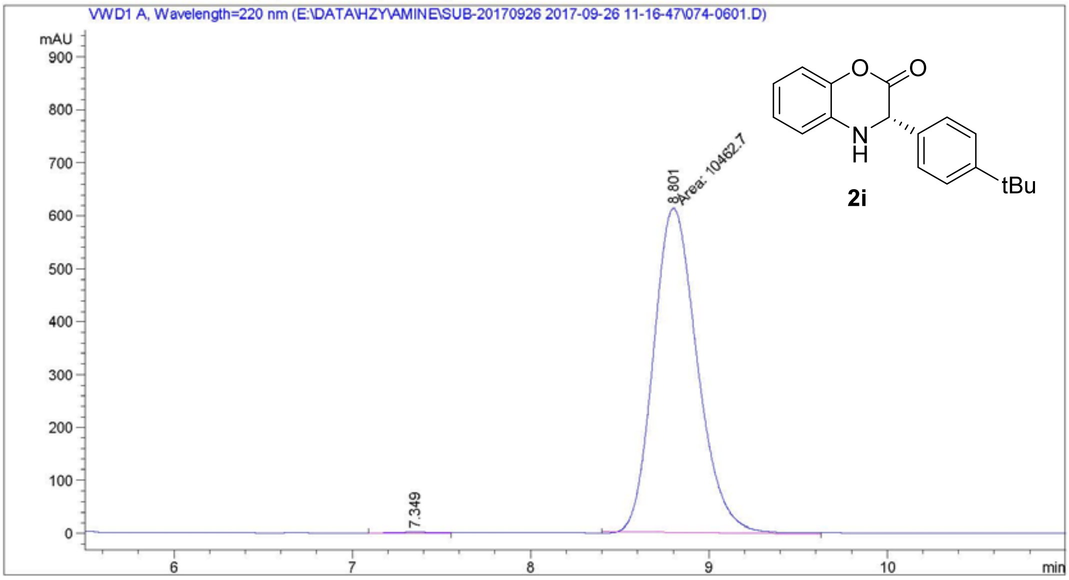


```
=====================================================================
                         Area Percent Report
=====================================================================

Sorted By             :      Signal
Multiplier            :      1.0000
Dilution              :      1.0000
Use Multiplier & Dilution Factor with ISTDs


Signal 1: VWD1 A, Wavelength=220 nm

Peak RetTime Type  Width     Area      Height     Area
  #   [min]        [min]   [mAU*s]     [mAU]        %
----|-------|----|-------|----------|----------|--------|
   1   7.349 BV    0.2105   32.48330    2.34623   0.3095
   2   8.801 MM    0.2845 1.04627e4  612.96301  99.6905

Totals :                  1.04952e4  615.30924
```

Data File E:\DATA\HY\HY-2017-9-29\HY-2017-9-29 2017-09-29 19-51-53\022-1201.D
Sample Name: PH-3ME-RAC

===

Acq. Operator : SYSTEM Seq. Line : 12
Acq. Instrument : 1260HPLC-DAD Location : Vial 22
Injection Date : 9/29/2017 11:37:58 PM Inj : 1
Inj Volume : 1.000 µl
Acq. Method : E:\DATA\HY\HY-2017-9-29\HY-2017-9-29 2017-09-29 19-51-53\DAD-OD(1-2)-80-20-1ML-1UL- ALL-50MIN.M
Last changed : 9/29/2017 8:27:55 PM by SYSTEM
Analysis Method : E:\DATA\HY\HY-2017-9-29\HY-2017-9-29 2017-09-29 19-51-53\DAD-OD(1-2)-80-20-1ML-1UL- ALL-50MIN.M (Sequence Method)
Last changed : 9/30/2017 9:12:45 AM by SYSTEM
(modified after loading)
Additional Info : Peak(s) manually integrated

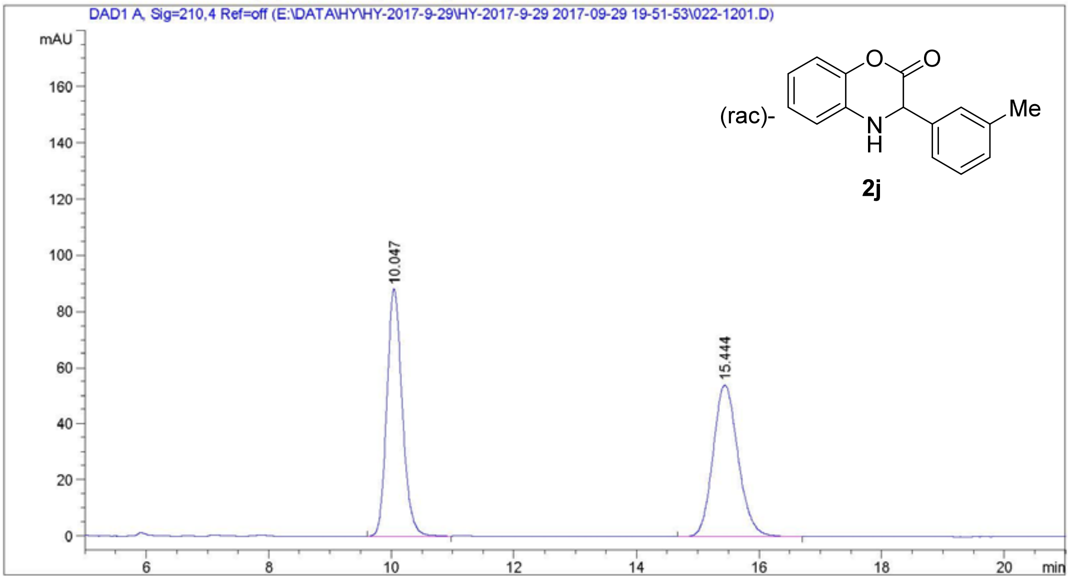

===

Area Percent Report

===

Sorted By : Signal
Multiplier : 1.0000
Dilution : 1.0000
Do not use Multiplier & Dilution Factor with ISTDs

Signal 1: DAD1 A, Sig=210,4 Ref=off

Peak #	RetTime [min]	Type	Width [min]	Area [mAU*s]	Height [mAU]	Area %
1	10.047	BB	0.2665	1532.39600	88.28191	50.0109
2	15.444	BB	0.4317	1531.72644	54.19786	49.9891

Totals : 3064.12244 142.47977

Data File E:\DATA\LYH\LYH-1-RAC\LYH-RAC-CYCLIC KETOESTER 2017-09-30 08-26-48\021-0601.D
Sample Name: 3ME

===

Acq. Operator : SYSTEM Seq. Line : 6
Acq. Instrument : 1260HPLC-DAD Location : Vial 21
Injection Date : 9/30/2017 10:32:26 AM Inj : 1
Inj Volume : 5.000 µl
Acq. Method : E:\DATA\LYH\LYH-1-RAC\LYH-RAC-CYCLIC KETOESTER 2017-09-30 08-26-48\DAD-OD(1-2)-80-20-1.0ML-5-ALL-25MIN.M
Last changed : 9/30/2017 10:51:11 AM by SYSTEM
(modified after loading)
Analysis Method : E:\DATA\LYH\LYH-1-RAC\LYH-RAC-CYCLIC KETOESTER 2017-09-30 08-26-48\DAD-OD(1-2)-80-20-1.0ML-5-ALL-25MIN.M (Sequence Method)
Last changed : 9/30/2017 10:56:16 AM by SYSTEM
(modified after loading)
Additional Info : Peak(s) manually integrated

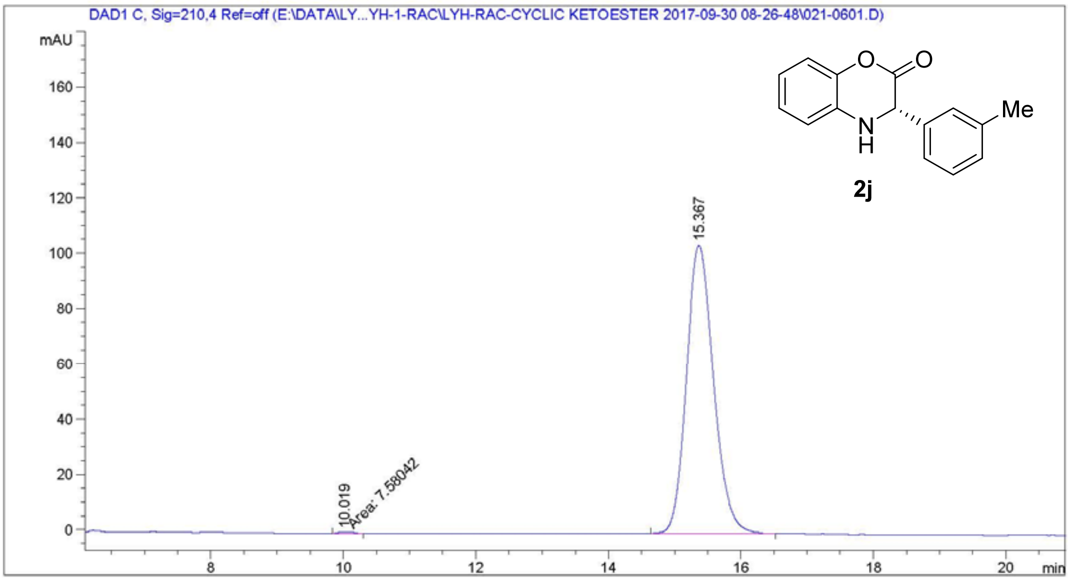

===

Area Percent Report

===

Sorted By : Signal
Multiplier : 1.0000
Dilution : 1.0000
Do not use Multiplier & Dilution Factor with ISTDs

Signal 1: DAD1 C, Sig=210,4 Ref=off

Peak #	RetTime [min]	Type	Width [min]	Area [mAU*s]	Height [mAU]	Area %
1	10.019	MM	0.2668	7.58042	4.73497e-1	0.2560
2	15.367	BB	0.4370	2953.37378	104.11096	99.7440

Totals : 2960.95420 104.58446

1260HPLC-DAD 9/30/2017 10:56:20 AM SYSTEM Page 1 of 2

Data File E:\DATA\LY...2-RAC\LYH-2-RAC-SULFAMINE-P-BR-P-OME 2017-10-20 19-20-45\072-0901.D
Sample Name: NAI1-PDC

===

Acq. Operator : SYSTEM Seq. Line : 9
Acq. Instrument : 1260HPLC-DAD Location : Vial 72
Injection Date : 10/20/2017 11:44:08 PM Inj : 1
Inj Volume : 1.000 µl
Acq. Method : E:\DATA\LYH\LYH-2-RAC\LYH-2-RAC-SULFAMINE-P-BR-P-OME 2017-10-20 19-20-45 \DAD-OD(1-2)-80-20-1ML-1UL- ALL-50MIN.M
Last changed : 10/20/2017 10:02:57 PM by SYSTEM
Analysis Method : E:\DATA\LYH\LYH-2-RAC\LYH-2-RAC-SULFAMINE-P-BR-P-OME 2017-10-20 19-20-45 \DAD-OD(1-2)-80-20-1ML-1UL- ALL-50MIN.M (Sequence Method)
Last changed : 10/21/2017 9:29:28 AM by SYSTEM
(modified after loading)
Additional Info : Peak(s) manually integrated

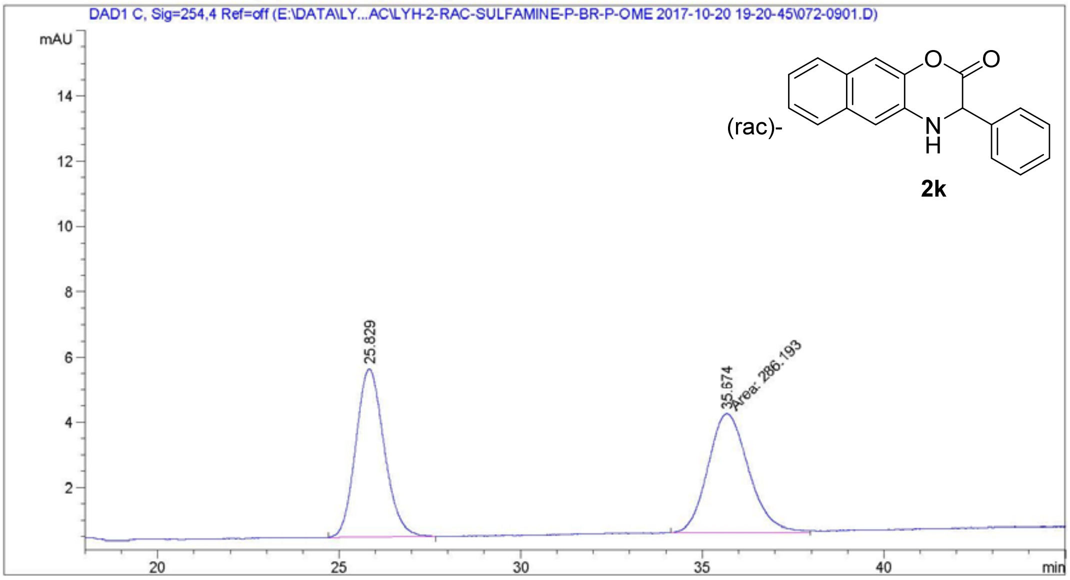

===

Area Percent Report

===

Sorted By : Signal
Multiplier : 1.0000
Dilution : 1.0000
Do not use Multiplier & Dilution Factor with ISTDs

Signal 1: DAD1 C, Sig=254,4 Ref=off

Peak #	RetTime [min]	Type	Width [min]	Area [mAU*s]	Height [mAU]	Area %
1	25.829	BB	0.7353	280.31314	5.16417	49.4811
2	35.674	MM	1.3062	286.19260	3.65182	50.5189

Totals : 566.50574 8.81599

Data File E:\DATA\LY...2-RAC\LYH-2-RAC-SULFAMINE-P-BR-P-OME 2017-10-20 19-20-45\071-0801.D
Sample Name: NAI1-EE

===

Acq. Operator : SYSTEM   Seq. Line : 8
Acq. Instrument : 1260HPLC-DAD   Location : Vial 71
Injection Date : 10/20/2017 10:53:14 PM   Inj : 1
Inj Volume : 1.000 µl
Acq. Method : E:\DATA\LYH\LYH-2-RAC\LYH-2-RAC-SULFAMINE-P-BR-P-OME 2017-10-20 19-20-45 \DAD-OD(1-2)-80-20-1ML-1UL- ALL-50MIN.M
Last changed : 10/20/2017 10:02:57 PM by SYSTEM
Analysis Method : E:\DATA\LYH\LYH-2-RAC\LYH-2-RAC-SULFAMINE-P-BR-P-OME 2017-10-20 19-20-45 \DAD-OD(1-2)-80-20-1ML-1UL- ALL-50MIN.M (Sequence Method)
Last changed : 10/21/2017 9:32:18 AM by SYSTEM
(modified after loading)
Additional Info : Peak(s) manually integrated

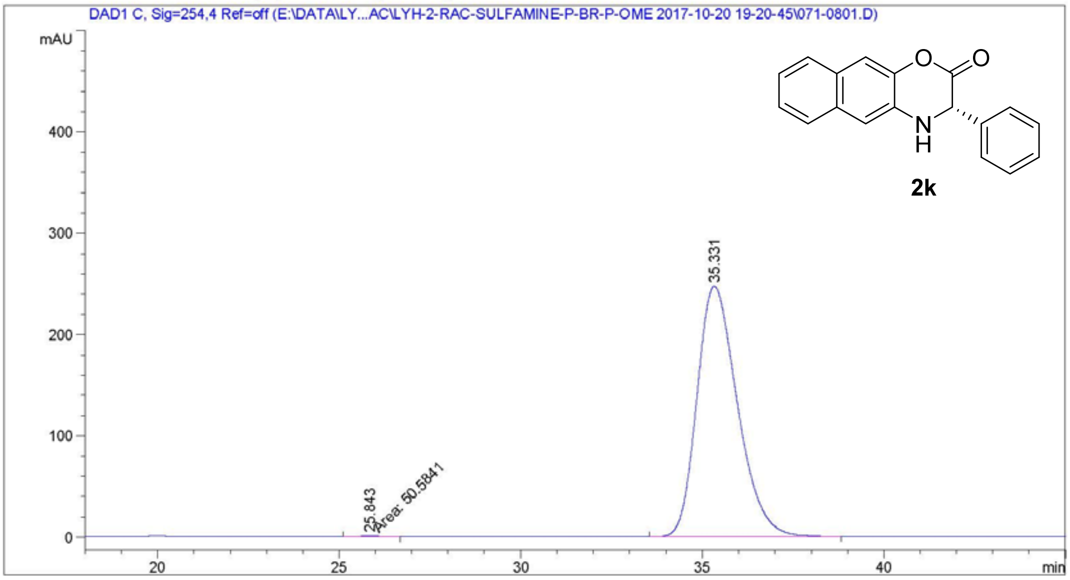

===

Area Percent Report

===

Sorted By : Signal
Multiplier : 1.0000
Dilution : 1.0000
Do not use Multiplier & Dilution Factor with ISTDs

Signal 1: DAD1 C, Sig=254,4 Ref=off

Peak #	RetTime [min]	Type	Width [min]	Area [mAU*s]	Height [mAU]	Area %
1	25.843	MM	0.8746	50.58405	9.63933e-1	0.2643
2	35.331	BB	1.1891	1.90906e4	247.16516	99.7357

Totals : 1.91412e4 248.12909

Data File E:\DATA\HZY\AMINE\NAI2-EE-171022 2017-10-21 20-13-48\073-0301.D
Sample Name: NAI2-RAC

===

Acq. Operator : SYSTEM   Seq. Line : 3
Acq. Instrument : 1260HPLC-DAD   Location : Vial 73
Injection Date : 10/21/2017 9:19:14 PM   Inj : 1
Inj Volume : 1.000 µl
Acq. Method : E:\DATA\HZY\AMINE\NAI2-EE-171022 2017-10-21 20-13-48\DAD-OD(1-2)-80-20-1ML-1UL- ALL-50MIN.M
Last changed : 10/21/2017 8:13:49 PM by SYSTEM
Analysis Method : E:\DATA\HZY\AMINE\NAI2-EE-171022 2017-10-21 20-13-48\DAD-OD(1-2)-80-20-1ML-1UL- ALL-50MIN.M (Sequence Method)
Last changed : 10/23/2017 9:47:27 PM by SYSTEM
(modified after loading)
Additional Info : Peak(s) manually integrated

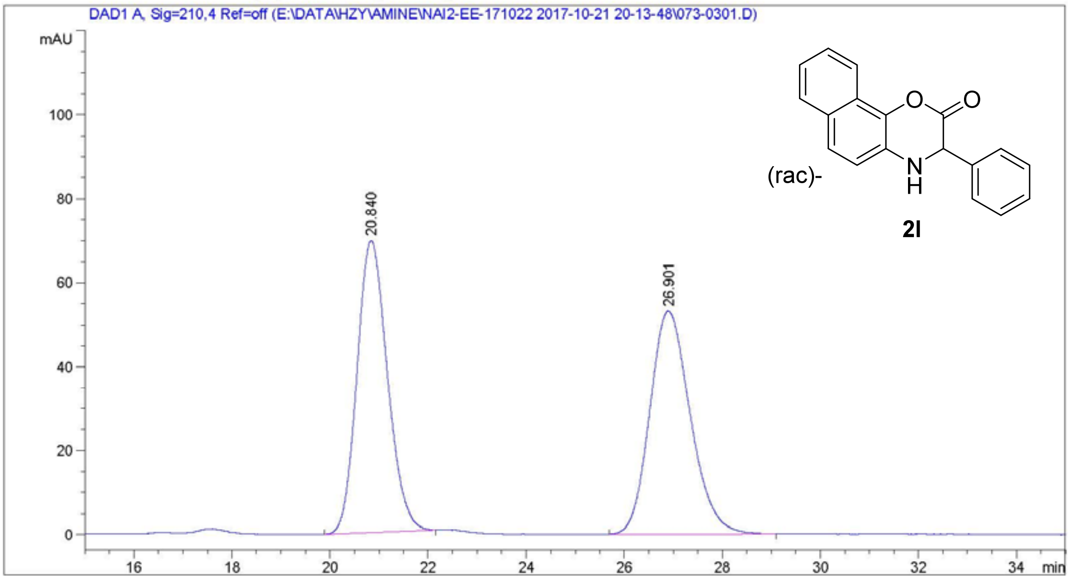

===
Area Percent Report
===

Sorted By : Signal
Multiplier : 1.0000
Dilution : 1.0000
Do not use Multiplier & Dilution Factor with ISTDs

Signal 1: DAD1 A, Sig=210,4 Ref=off

Peak #	RetTime [min]	Type	Width [min]	Area [mAU*s]	Height [mAU]	Area %
1	20.840	BB	0.6517	2993.80566	69.61648	49.5497
2	26.901	BB	0.8577	3048.21777	53.26270	50.4503

Totals : 6042.02344 122.87918

Data File E:\DATA\HZY\AMINE\NAI2-EE-171022 2017-10-21 20-13-48\074-0201.D
Sample Name: NAI2-EE

===

Acq. Operator : SYSTEM Seq. Line : 2
Acq. Instrument : 1260HPLC-DAD Location : Vial 74
Injection Date : 10/21/2017 8:28:18 PM Inj : 1
Inj Volume : 1.000 µl
Acq. Method : E:\DATA\HZY\AMINE\NAI2-EE-171022 2017-10-21 20-13-48\DAD-OD(1-2)-80-20-1ML-1UL- ALL-50MIN.M
Last changed : 10/21/2017 8:13:49 PM by SYSTEM
Analysis Method : E:\DATA\HZY\AMINE\NAI2-EE-171022 2017-10-21 20-13-48\DAD-OD(1-2)-80-20-1ML-1UL- ALL-50MIN.M (Sequence Method)
Last changed : 10/23/2017 9:48:35 PM by SYSTEM
(modified after loading)
Additional Info : Peak(s) manually integrated

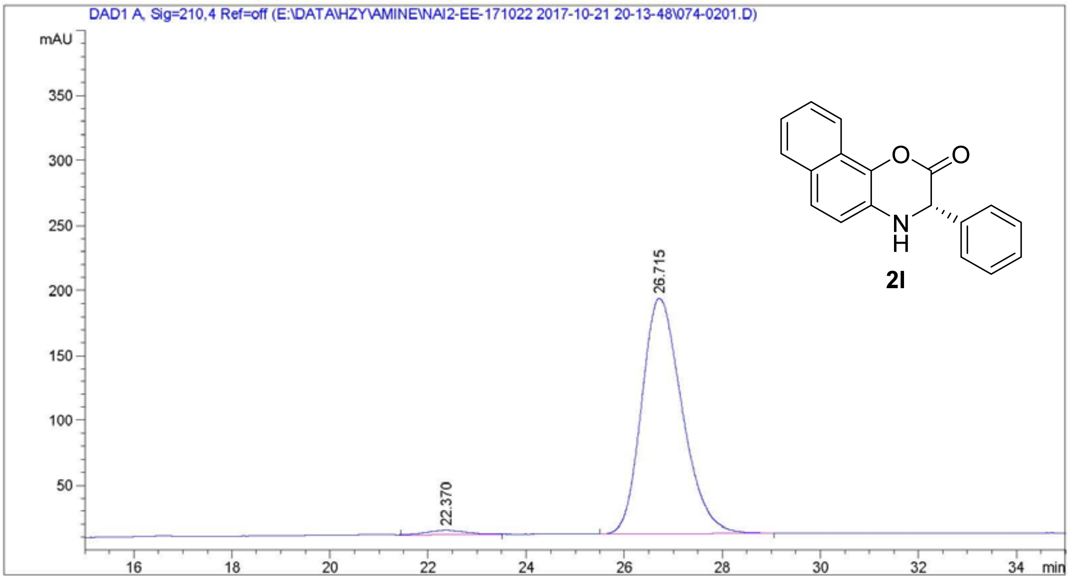

===

Area Percent Report

===

Sorted By : Signal
Multiplier : 1.0000
Dilution : 1.0000
Do not use Multiplier & Dilution Factor with ISTDs

Signal 1: DAD1 A, Sig=210,4 Ref=off

Peak #	RetTime [min]	Type	Width [min]	Area [mAU*s]	Height [mAU]	Area %
1	22.370	BB	0.6377	180.75034	3.32791	1.7128
2	26.715	BB	0.8809	1.03722e4	181.43884	98.2872

Totals : 1.05529e4 184.76675

Data File E:\DATA\HZY\AMINE\SF-NAI-171020 2017-10-20 10-52-27\041-0201.D
Sample Name: SF-PDC

===

Acq. Operator : SYSTEM Seq. Line : 2
Acq. Instrument : 1260HPLC-DAD Location : Vial 41
Injection Date : 10/20/2017 11:04:22 AM Inj : 1
Inj Volume : 1.000 µl
Acq. Method : E:\DATA\HZY\AMINE\SF-NAI-171020 2017-10-20 10-52-27\DAD-OD(1-2)-80-20-1ML-1UL-30MIN.M
Last changed : 10/20/2017 10:52:28 AM by SYSTEM
Analysis Method : E:\DATA\HZY\AMINE\SF-NAI-171020 2017-10-20 10-52-27\DAD-OD(1-2)-80-20-1ML-1UL-30MIN.M (Sequence Method)
Last changed : 10/25/2017 10:38:23 AM by SYSTEM
(modified after loading)
Additional Info : Peak(s) manually integrated

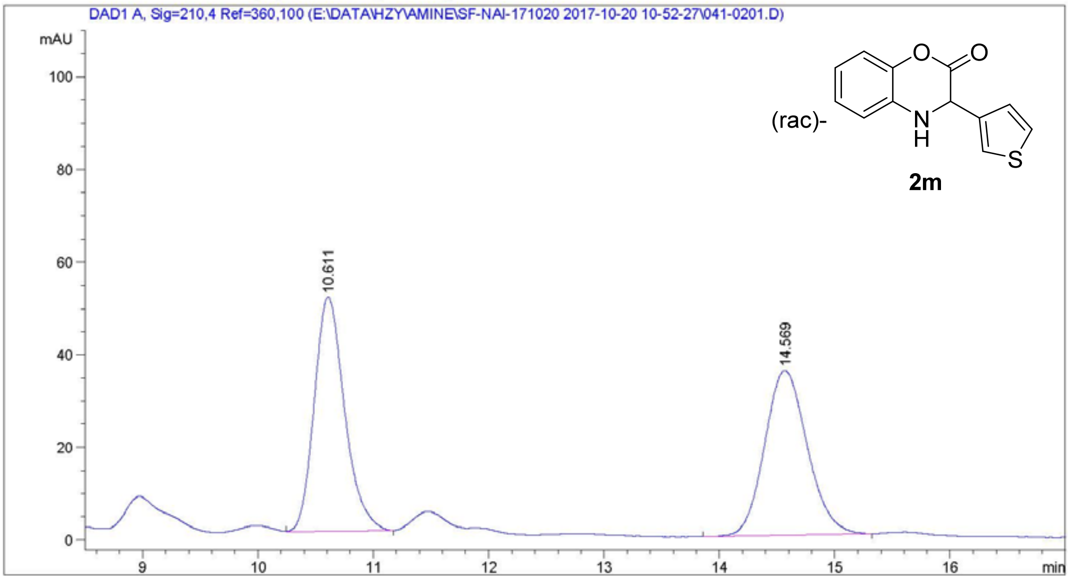

===

Area Percent Report

===

Sorted By : Signal
Multiplier : 1.0000
Dilution : 1.0000
Do not use Multiplier & Dilution Factor with ISTDs

Signal 1: DAD1 A, Sig=210,4 Ref=360,100

Peak #	RetTime [min]	Type	Width [min]	Area [mAU*s]	Height [mAU]	Area %
1	10.611	VB	0.2784	921.38617	50.62161	50.3326
2	14.569	BB	0.3980	909.20764	35.59722	49.6674

Totals : 1830.59381 86.21883

Data File E:\DATA\HZY\AMINE\RINGOPEN 2017-10-27 21-25-38\041-0401.D
Sample Name: SF-20171027

===

Acq. Operator : SYSTEM Seq. Line : 4
Acq. Instrument : 1260HPLC-DAD Location : Vial 41
Injection Date : 10/27/2017 10:27:04 PM Inj : 1
Inj Volume : 1.000 µl
Acq. Method : E:\DATA\HZY\AMINE\RINGOPEN 2017-10-27 21-25-38\DAD-OD(1-2)-80-20-1ML-1UL-30MIN.M
Last changed : 10/27/2017 9:25:38 PM by SYSTEM
Analysis Method : E:\DATA\HZY\AMINE\RINGOPEN 2017-10-27 21-25-38\DAD-OD(1-2)-80-20-1ML-1UL-30MIN.M (Sequence Method)
Last changed : 10/30/2017 9:05:45 AM by SYSTEM
(modified after loading)
Additional Info : Peak(s) manually integrated

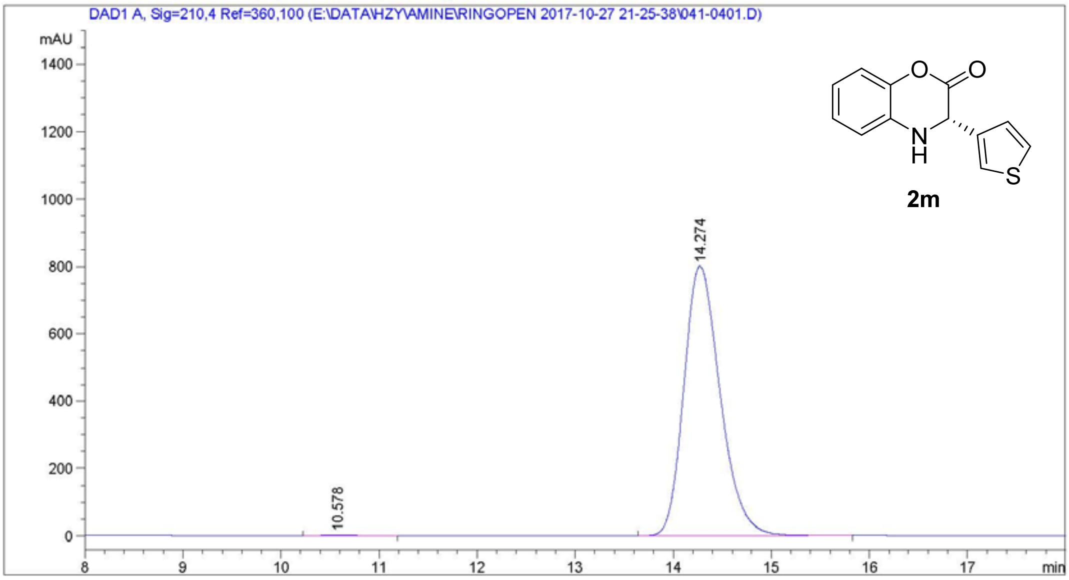

===
Area Percent Report
===

Sorted By : Signal
Multiplier : 1.0000
Dilution : 1.0000
Do not use Multiplier & Dilution Factor with ISTDs

Signal 1: DAD1 A, Sig=210,4 Ref=360,100

Peak #	RetTime [min]	Type	Width [min]	Area [mAU*s]	Height [mAU]	Area %
1	10.578	BB	0.2685	53.75104	2.97956	0.2604
2	14.274	BB	0.3959	2.05885e4	801.00885	99.7396

Totals : 2.06422e4 803.98841

Data File E:\DATA\HZY\AMINE\ME-EE 2017-07-15 08-55-42\023-0401.D
Sample Name: ME-PDC

===

Acq. Operator : SYSTEM Seq. Line : 4
Acq. Instrument : 1260HPLC-DAD Location : Vial 23
Injection Date : 7/15/2017 9:40:12 AM Inj : 1
Inj Volume : 2.000 µl
Acq. Method : E:\DATA\HZY\AMINE\ME-EE 2017-07-15 08-55-42\DAD-OD(1-2)-90-10-1ML-2UL-25MIN.M
Last changed : 7/15/2017 8:55:42 AM by SYSTEM
Analysis Method : E:\DATA\HZY\AMINE\ME-EE 2017-07-15 08-55-42\DAD-OD(1-2)-90-10-1ML-2UL-25MIN.M (Sequence Method)
Last changed : 7/17/2017 10:18:56 AM by SYSTEM
(modified after loading)
Additional Info : Peak(s) manually integrated

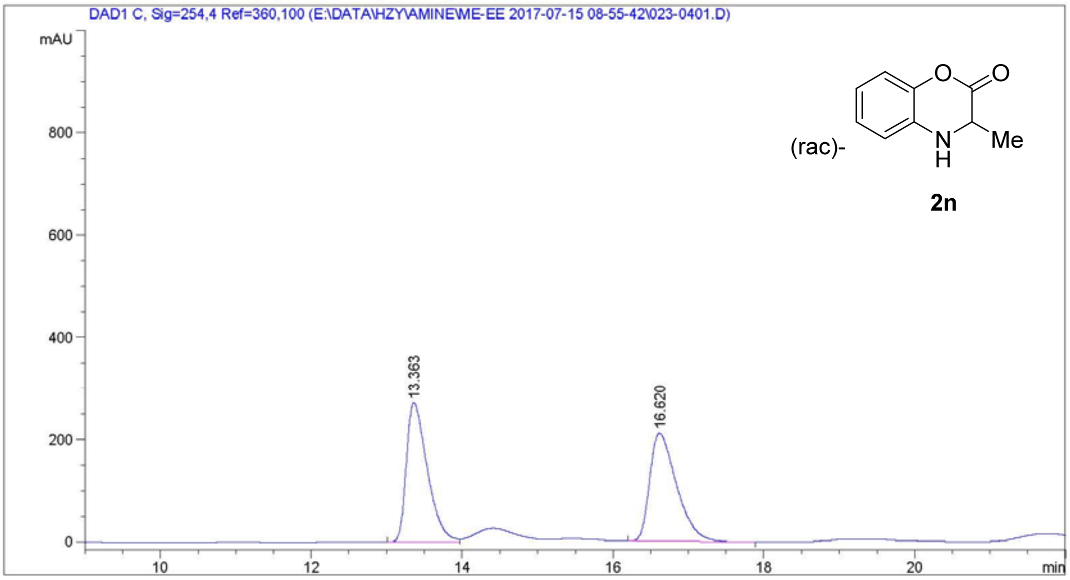

===

Area Percent Report

===

Sorted By : Signal
Multiplier : 1.0000
Dilution : 1.0000
Do not use Multiplier & Dilution Factor with ISTDs

Signal 1: DAD1 C, Sig=254,4 Ref=360,100

Peak #	RetTime [min]	Type	Width [min]	Area [mAU*s]	Height [mAU]	Area %
1	13.363	BV	0.2925	5232.71631	271.98199	50.0964
2	16.620	BB	0.3722	5212.57422	210.97859	49.9036

Totals : 1.04453e4 482.96059

Data File E:\DATA\HZY\AMINE\ME-EE-N-ME-L-OD 2017-09-07 09-56-36\001-0201.D
Sample Name: Hex

===

Acq. Operator : SYSTEM Seq. Line : 2
Acq. Instrument : 1260HPLC-VWD Location : Vial 1
Injection Date : 9/7/2017 10:10:03 AM Inj : 1
Inj Volume : 1.000 µl
Acq. Method : E:\DATA\HZY\AMINE\ME-EE-N-ME-L-OD 2017-09-07 09-56-36\VWD-AD(1-6)-90-10-1ML-1UL-10MIN.M
Last changed : 9/7/2017 10:34:42 AM by SYSTEM
(modified after loading)
Analysis Method : E:\DATA\HZY\AMINE\ME-EE-N-ME-L-OD 2017-09-07 09-56-36\VWD-AD(1-6)-90-10-1ML-1UL-10MIN.M (Sequence Method)
Last changed : 9/7/2017 10:35:48 AM by SYSTEM
(modified after loading)
Additional Info : Peak(s) manually integrated

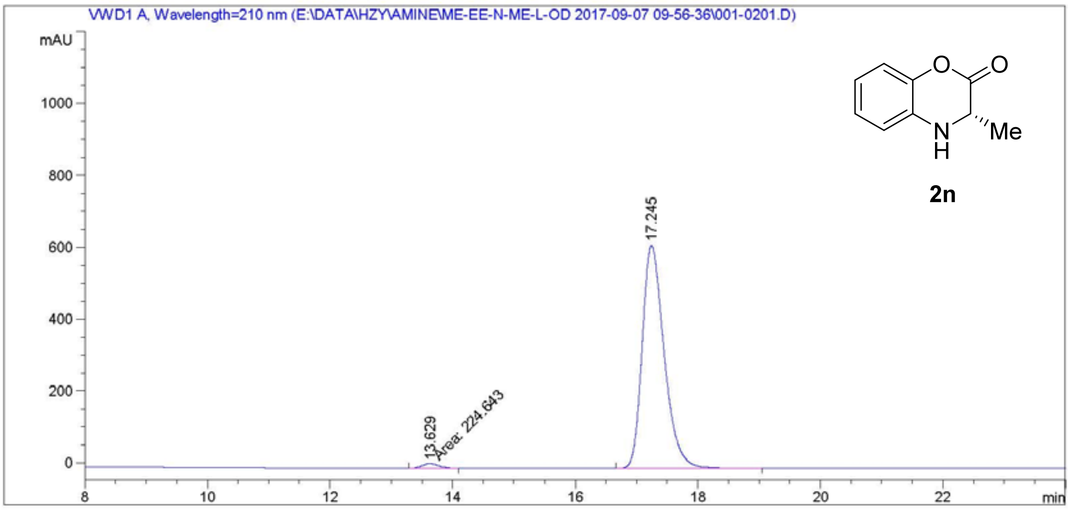

===

Area Percent Report

===

Sorted By : Signal
Multiplier : 1.0000
Dilution : 1.0000
Do not use Multiplier & Dilution Factor with ISTDs

Signal 1: VWD1 A, Wavelength=210 nm

Peak #	RetTime [min]	Type	Width [min]	Area [mAU*s]	Height [mAU]	Area %
1	13.629	MM	0.2945	224.64258	12.71405	1.4388
2	17.245	BB	0.3804	1.53891e4	620.48462	98.5612

Totals : 1.56137e4 633.19866

===

1260HPLC-VWD 9/7/2017 10:36:26 AM SYSTEM Page 1 of 1

Data File D:\DATA\LWD\LWD-5-5\LWD-5-5 2019-02-21 11-11-04\093-1001.D
Sample Name: IPR-RAC

===

Acq. Operator : Seq. Line : 10
Acq. Instrument : Instrument 2 Location : Vial 93
Injection Date : 2/21/2019 2:51:38 PM Inj : 1
Inj Volume : 2.000 µl
Acq. Method : D:\DATA\LWD\LWD-5-5\LWD-5-5 2019-02-21 11-11-04\DAD-OD(1-2)-90-10-1ML-2UL-ALL-40MIN.M
Last changed : 9/22/2018 11:08:15 AM
Analysis Method : D:\METHOD\HZY\DAD-OJ(1-6)-80-20-1ML-1UL-ALL-50MIN.M
Last changed : 2/21/2019 4:18:37 PM
(modified after loading)
Additional Info : Peak(s) manually integrated

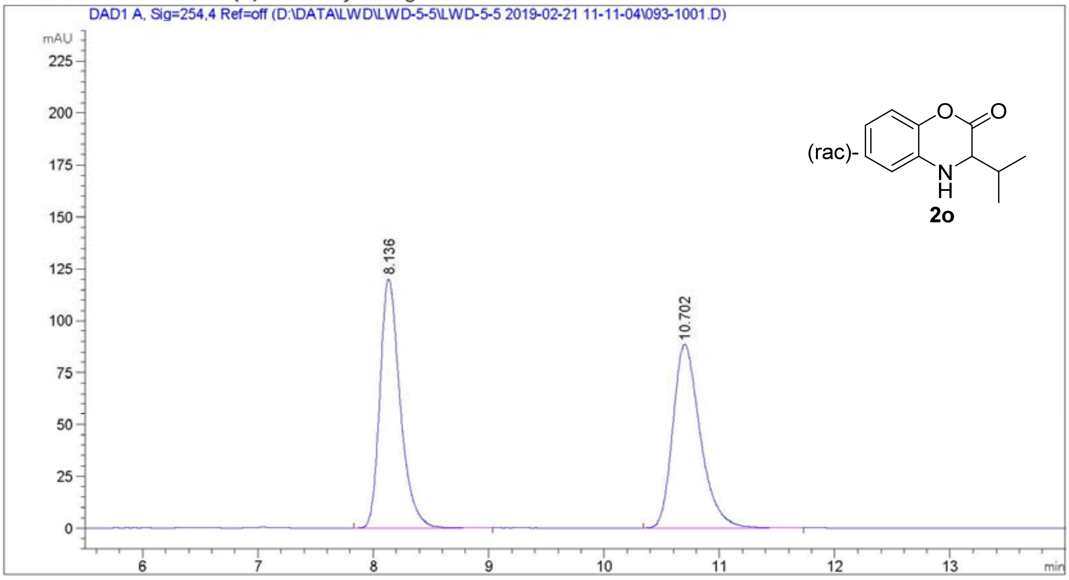

===
Area Percent Report
===

Sorted By : Signal
Multiplier : 1.0000
Dilution : 1.0000
Use Multiplier & Dilution Factor with ISTDs

Signal 1: DAD1 A, Sig=254,4 Ref=off

Peak #	RetTime [min]	Type	Width [min]	Area [mAU*s]	Height [mAU]	Area %
1	8.136	BB	0.1837	1457.57532	120.01909	49.9095
2	10.702	BB	0.2508	1462.86389	88.50265	50.0905

Totals : 2920.43921 208.52174

Data File D:\DATA\LWD\LWD-5-5\LWD-5-5 2019-02-21 11-11-04\092-0901.D
Sample Name: IPR-EE

===

Acq. Operator : Seq. Line : 9
Acq. Instrument : Instrument 2 Location : Vial 92
Injection Date : 2/21/2019 2:10:40 PM Inj : 1
Inj Volume : 2.000 µl
Acq. Method : D:\DATA\LWD\LWD-5-5\LWD-5-5 2019-02-21 11-11-04\DAD-OD(1-2)-90-10-1ML-2UL-ALL-40MIN.M
Last changed : 9/22/2018 11:08:15 AM
Analysis Method : D:\METHOD\HZY\DAD-OJ(1-6)-80-20-1ML-1UL-ALL-50MIN.M
Last changed : 2/21/2019 4:20:02 PM
(modified after loading)
Additional Info : Peak(s) manually integrated

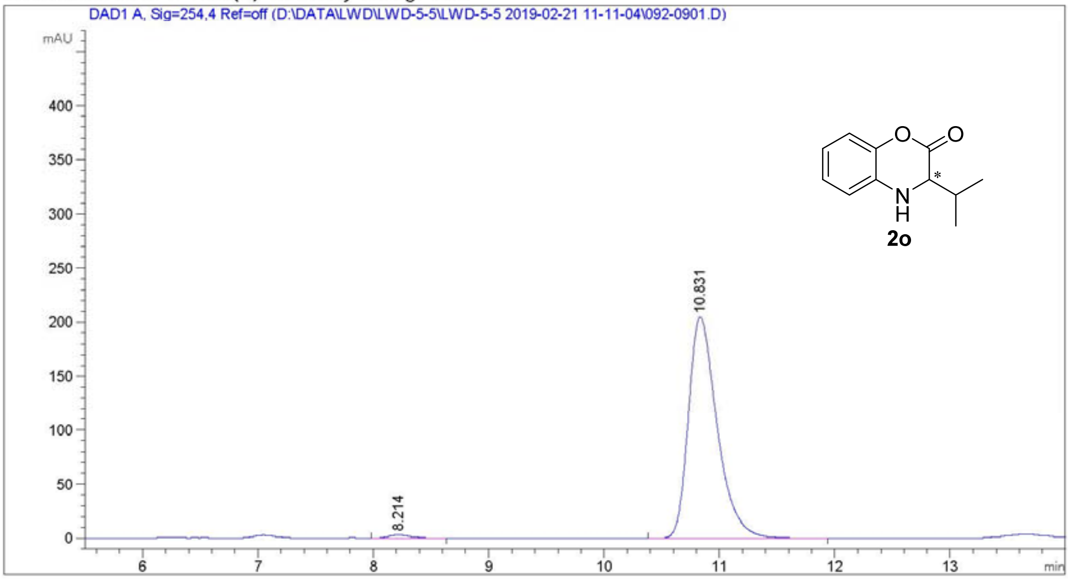

===

Area Percent Report

===

Sorted By : Signal
Multiplier : 1.0000
Dilution : 1.0000
Use Multiplier & Dilution Factor with ISTDs

Signal 1: DAD1 A, Sig=254,4 Ref=off

Peak #	RetTime [min]	Type	Width [min]	Area [mAU*s]	Height [mAU]	Area %
1	8.214	BB	0.1930	39.29892	3.07772	1.1013
2	10.831	BB	0.2611	3529.16602	204.70740	98.8987

Totals : 3568.46493 207.78512

Data File E:\DATA\HZY\AMINE\SUB-20170926 2017-09-26 11-16-47\083-0501.D
Sample Name: H-3-RAC

===

Acq. Operator : SYSTEM   Seq. Line : 5
Acq. Instrument : 1260HPLC-VWD   Location : Vial 83
Injection Date : 9/26/2017 12:58:42 PM   Inj : 1
Inj Volume : 3.000 µl
Acq. Method : E:\DATA\HZY\AMINE\SUB-20170926 2017-09-26 11-16-47\VWD-OD(1-2)-80-20-1ML-3UL-220NM-35MIN.M
Last changed : 9/26/2017 11:47:09 AM by SYSTEM
Analysis Method : E:\DATA\HZY\AMINE\SUB-20170926 2017-09-26 11-16-47\VWD-OD(1-2)-80-20-1ML-3UL-220NM-35MIN.M (Sequence Method)
Last changed : 10/16/2017 10:21:43 AM by SYSTEM
(modified after loading)
Additional Info : Peak(s) manually integrated

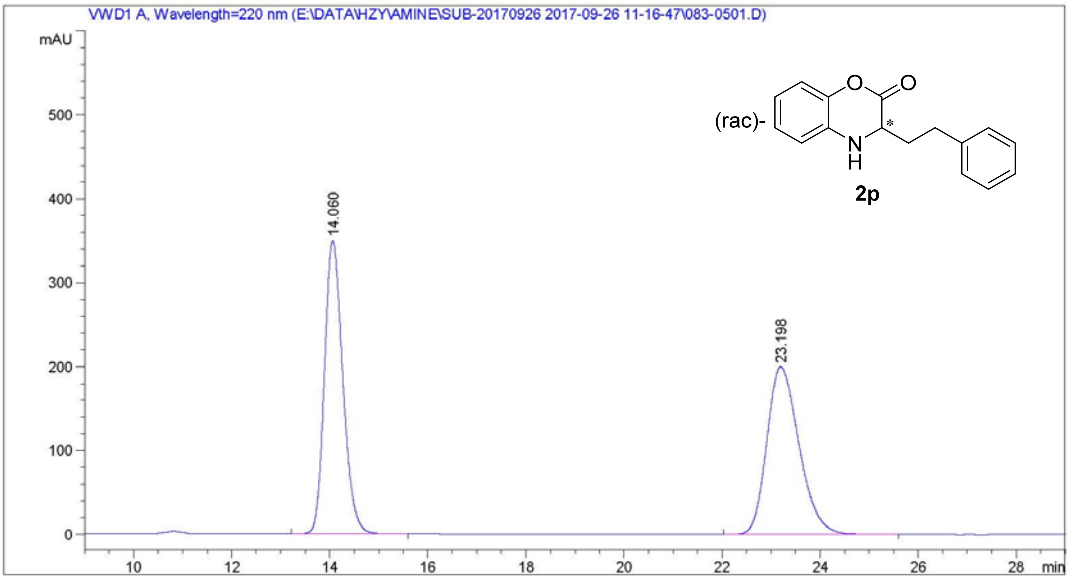

===

Area Percent Report

===

Sorted By : Signal
Multiplier : 1.0000
Dilution : 1.0000
Use Multiplier & Dilution Factor with ISTDs

Signal 1: VWD1 A, Wavelength=220 nm

Peak #	RetTime [min]	Type	Width [min]	Area [mAU*s]	Height [mAU]	Area %
1	14.060	BB	0.4013	9087.33691	349.60501	49.9043
2	23.198	BB	0.7052	9122.19727	199.98453	50.0957

Totals : 1.82095e4 549.58954

Data File E:\DATA\LYH\LYH-2-345\LYH-2-345 2017-10-14 16-30-48\041-1301.D
Sample Name: HZY-MEMEPH

===

Acq. Operator : SYSTEM Seq. Line : 13
Acq. Instrument : 1260HPLC-DAD Location : Vial 41
Injection Date : 10/14/2017 9:53:16 PM Inj : 1
Inj Volume : 1.000 µl
Acq. Method : E:\DATA\LYH\LYH-2-345\LYH-2-345 2017-10-14 16-30-48\DAD-OD(1-2)-80-20-1ML-1UL-30MIN.M
Last changed : 10/14/2017 8:22:51 PM by SYSTEM
Analysis Method : E:\DATA\LYH\LYH-2-345\LYH-2-345 2017-10-14 16-30-48\DAD-OD(1-2)-80-20-1ML-1UL-30MIN.M (Sequence Method)
Last changed : 10/16/2017 10:20:06 AM by SYSTEM
(modified after loading)
Additional Info : Peak(s) manually integrated

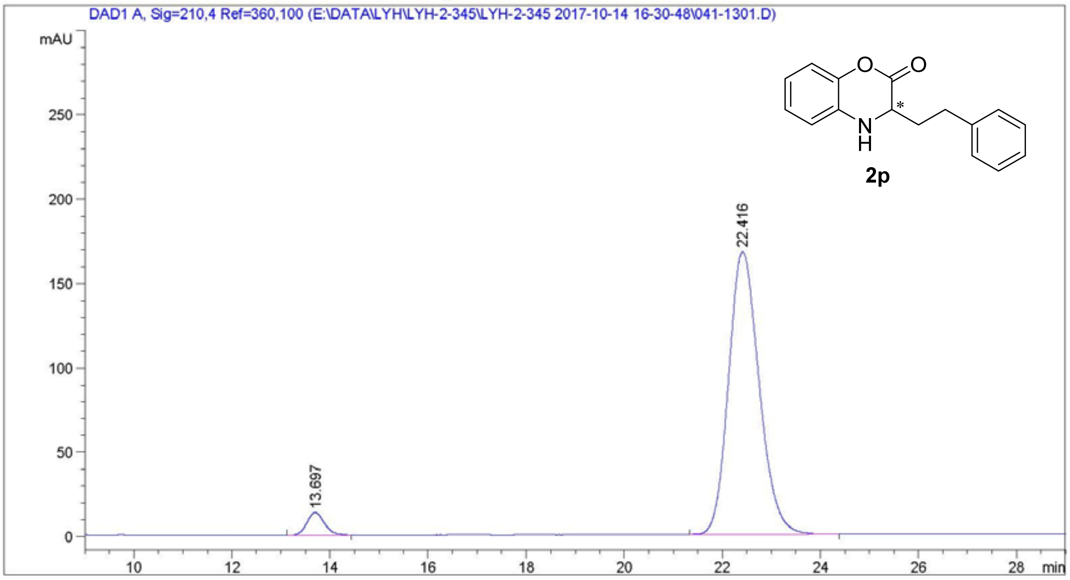

===

Area Percent Report

===

Sorted By : Signal
Multiplier : 1.0000
Dilution : 1.0000
Do not use Multiplier & Dilution Factor with ISTDs

Signal 1: DAD1 A, Sig=210,4 Ref=360,100

Peak #	RetTime [min]	Type	Width [min]	Area [mAU*s]	Height [mAU]	Area %
1	13.697	BB	0.3763	324.98087	13.24320	4.2965
2	22.416	BB	0.6643	7238.87744	167.45334	95.7035

Totals : 7563.85831 180.69654

Data File E:\DATA\HZY\AMINE\YSH-EE-20171229 2017-12-29 19-38-12\022-0401.D
Sample Name: YSH-BINAP

===

Acq. Operator : SYSTEM Seq. Line : 4
Acq. Instrument : 1260HPLC-DAD Location : Vial 22
Injection Date : 12/29/2017 9:22:22 PM Inj : 1
Inj Volume : 25.000 µl
Acq. Method : E:\DATA\HZY\AMINE\YSH-EE-20171229 2017-12-29 19-38-12\DAD-OD(1-2)-80-20-1ML-25UL-70MIN.M
Last changed : 12/29/2017 7:38:12 PM by SYSTEM
Analysis Method : E:\DATA\HZY\AMINE\YSH-EE-20171229 2017-12-29 19-38-12\DAD-OD(1-2)-80-20-1ML-25UL-70MIN.M (Sequence Method)
Last changed : 1/3/2018 3:38:45 PM by SYSTEM
(modified after loading)
Additional Info : Peak(s) manually integrated

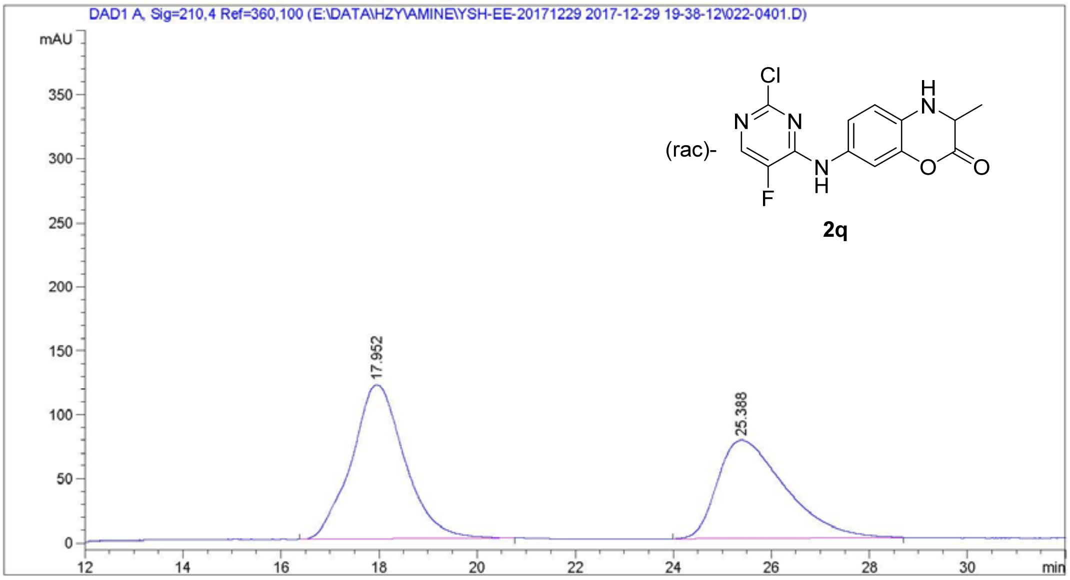

===

Area Percent Report

===

Sorted By : Signal
Multiplier : 1.0000
Dilution : 1.0000
Do not use Multiplier & Dilution Factor with ISTDs

Signal 1: DAD1 A, Sig=210,4 Ref=360,100

Peak #	RetTime [min]	Type	Width [min]	Area [mAU*s]	Height [mAU]	Area %
1	17.952	BB	1.0556	9064.62305	120.77467	55.2085
2	25.388	BB	1.2547	7354.26172	76.69269	44.7915

Totals : 1.64189e4 197.46736

Data File E:\DATA\HZY\AMINE\YSH-ZHAOPHOS-20171230 2017-12-30 10-34-22\021-0401.D
Sample Name: ysh-ee

===

Acq. Operator : SYSTEM Seq. Line : 4
Acq. Instrument : 1260HPLC-DAD Location : Vial 21
Injection Date : 12/30/2017 12:01:28 PM Inj : 1
Inj Volume : 1.000 µl
Acq. Method : E:\DATA\HZY\AMINE\YSH-ZHAOPHOS-20171230 2017-12-30 10-34-22\DAD-OD(1-2)-80-20-1ML-1UL- ALL-50MIN.M
Last changed : 12/30/2017 10:34:26 AM by SYSTEM
Analysis Method : E:\DATA\HZY\AMINE\YSH-ZHAOPHOS-20171230 2017-12-30 10-34-22\DAD-OD(1-2)-80-20-1ML-1UL- ALL-50MIN.M (Sequence Method)
Last changed : 1/3/2018 3:36:55 PM by SYSTEM
(modified after loading)
Additional Info : Peak(s) manually integrated

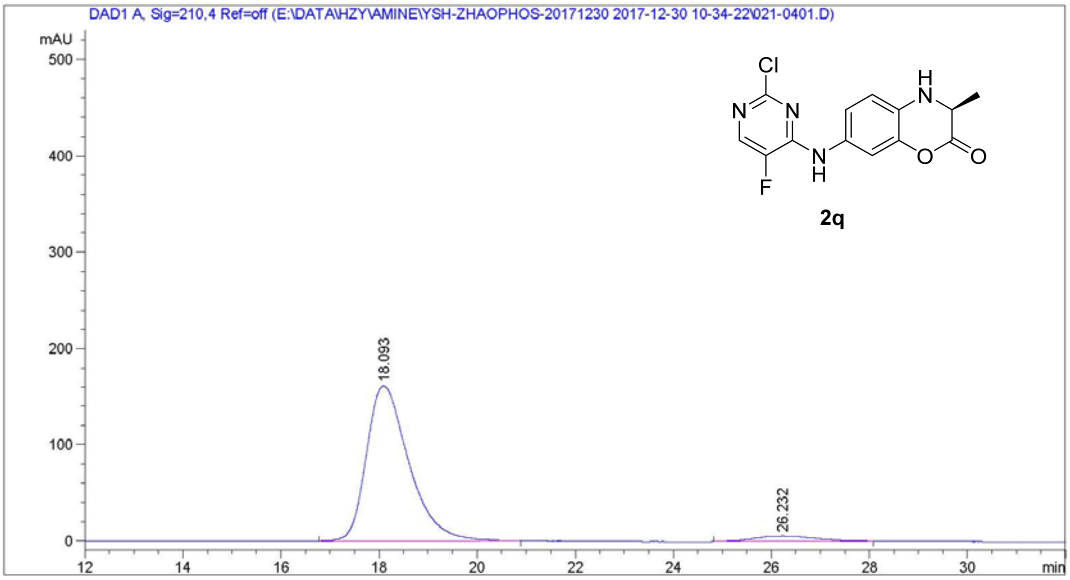

===

Area Percent Report

===

Sorted By : Signal
Multiplier : 1.0000
Dilution : 1.0000
Do not use Multiplier & Dilution Factor with ISTDs

Signal 1: DAD1 A, Sig=210,4 Ref=off

Peak #	RetTime [min]	Type	Width [min]	Area [mAU*s]	Height [mAU]	Area %
1	18.093	BB	0.8958	9664.89551	161.57863	95.3760
2	26.232	BB	1.0222	468.56891	5.44397	4.6240

Totals : 1.01335e4 167.02260

Data File E:\DATA\HZY\AMINE\O-N-EE 2017-07-19 09-58-03\012-0301.D
Sample Name: amide-RAC

===

Acq. Operator : SYSTEM Seq. Line : 3
Acq. Instrument : 1260HPLC-DAD Location : Vial 12
Injection Date : 7/19/2017 10:44:30 AM Inj : 1
Inj Volume : 1.000 µl
Acq. Method : E:\DATA\HZY\AMINE\O-N-EE 2017-07-19 09-58-03\DAD-OD(1-2)-80-20-1ML-1UL-30MIN.M
Last changed : 7/19/2017 9:59:10 AM by SYSTEM
Analysis Method : E:\DATA\HZY\AMINE\O-N-EE 2017-07-19 09-58-03\DAD-OD(1-2)-80-20-1ML-1UL-30MIN.M (Sequence Method)
Last changed : 7/19/2017 4:30:58 PM by SYSTEM
(modified after loading)
Additional Info : Peak(s) manually integrated

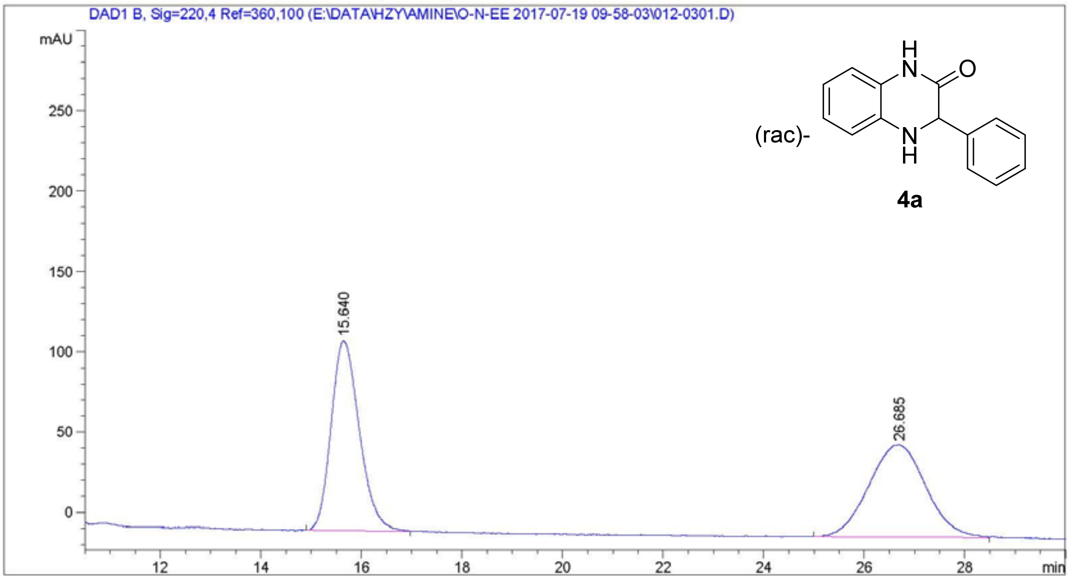

===

Area Percent Report

===

Sorted By : Signal
Multiplier : 1.0000
Dilution : 1.0000
Do not use Multiplier & Dilution Factor with ISTDs

Signal 1: DAD1 B, Sig=220,4 Ref=360,100

Peak #	RetTime [min]	Type	Width [min]	Area [mAU*s]	Height [mAU]	Area %
1	15.640	BV	0.5234	4606.42627	118.53674	50.3981
2	26.685	BV	0.9323	4533.64551	57.59998	49.6019

Totals : 9140.07178 176.13672

Data File E:\DATA\HZY\AMINE\ZHAOPHOS-171102 2017-11-02 10-46-55\044-1301.D
Sample Name: H-12-30MIN

===

Acq. Operator	: SYSTEM	Seq. Line :	13
Acq. Instrument	: 1260HPLC-DAD	Location :	Vial 44
Injection Date	: 11/2/2017 4:29:24 PM	Inj :	1
		Inj Volume :	1.000 µl

Acq. Method : E:\DATA\HZY\AMINE\ZHAOPHOS-171102 2017-11-02 10-46-55\DAD-OD(1-2)-80-20-1ML-1UL-30MIN.M
Last changed : 11/2/2017 3:04:51 PM by SYSTEM
Analysis Method : E:\DATA\HZY\AMINE\ZHAOPHOS-171102 2017-11-02 10-46-55\DAD-OD(1-2)-80-20-1ML-1UL-30MIN.M (Sequence Method)
Last changed : 11/2/2017 5:01:14 PM by SYSTEM
(modified after loading)
Additional Info : Peak(s) manually integrated

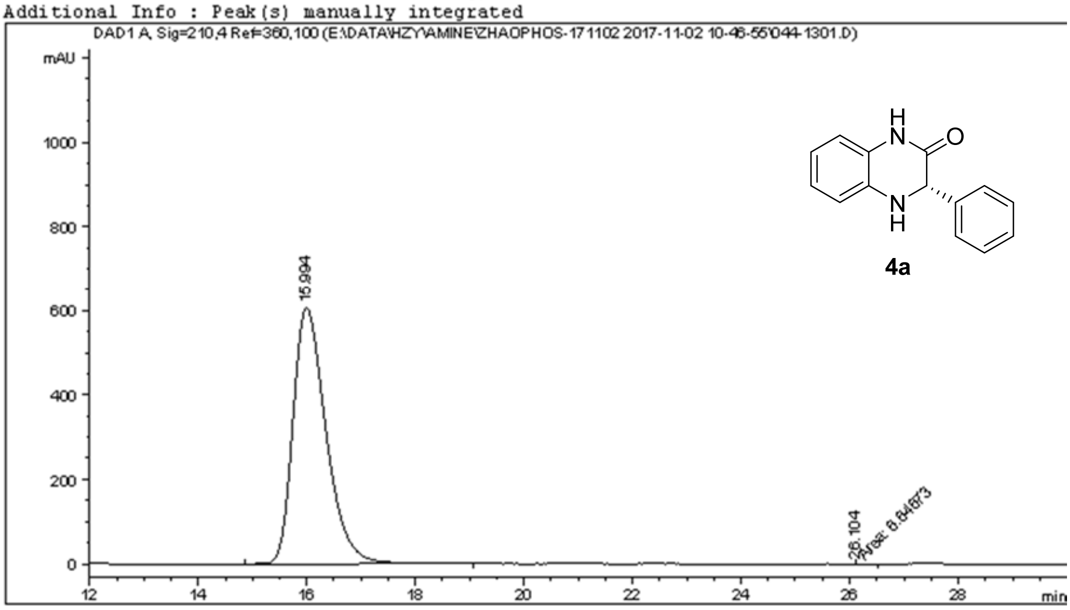

===
Area Percent Report
===

Sorted By : Signal
Multiplier : 1.0000
Dilution : 1.0000
Do not use Multiplier & Dilution Factor with ISTDs

Signal 1: DAD1 A, Sig=210,4 Ref=360,100

Peak #	RetTime [min]	Type	Width [min]	Area [mAU*s]	Height [mAU]	Area %
1	15.994	BB	0.6469	2.56347e4	606.70441	99.9741
2	26.104	MM	0.2573	6.64673	3.05338e-1	0.0259

Totals : 2.56414e4 607.00975

===
*** End of Report ***

1260HPLC-DAD 11/2/2017 5:02:18 PM SYSTEM Page 1 of 1

Data File E:\DATA\HZY\AMINE\SUB-20170926 2017-09-26 11-16-47\081-0301.D
Sample Name: H-1-RAC

===

Acq. Operator : SYSTEM Seq. Line : 3
Acq. Instrument : 1260HPLC-VWD Location : Vial 81
Injection Date : 9/26/2017 11:57:10 AM Inj : 1
Inj Volume : 2.000 µl
Acq. Method : E:\DATA\HZY\AMINE\SUB-20170926 2017-09-26 11-16-47\VWD-OD(1-2)-80-20-1ML-2UL-220NM-25MIN.M
Last changed : 9/26/2017 11:44:47 AM by SYSTEM
Analysis Method : E:\DATA\HZY\AMINE\SUB-20170926 2017-09-26 11-16-47\VWD-OD(1-2)-80-20-1ML-2UL-220NM-25MIN.M (Sequence Method)
Last changed : 9/26/2017 2:39:29 PM by SYSTEM
(modified after loading)
Additional Info : Peak(s) manually integrated

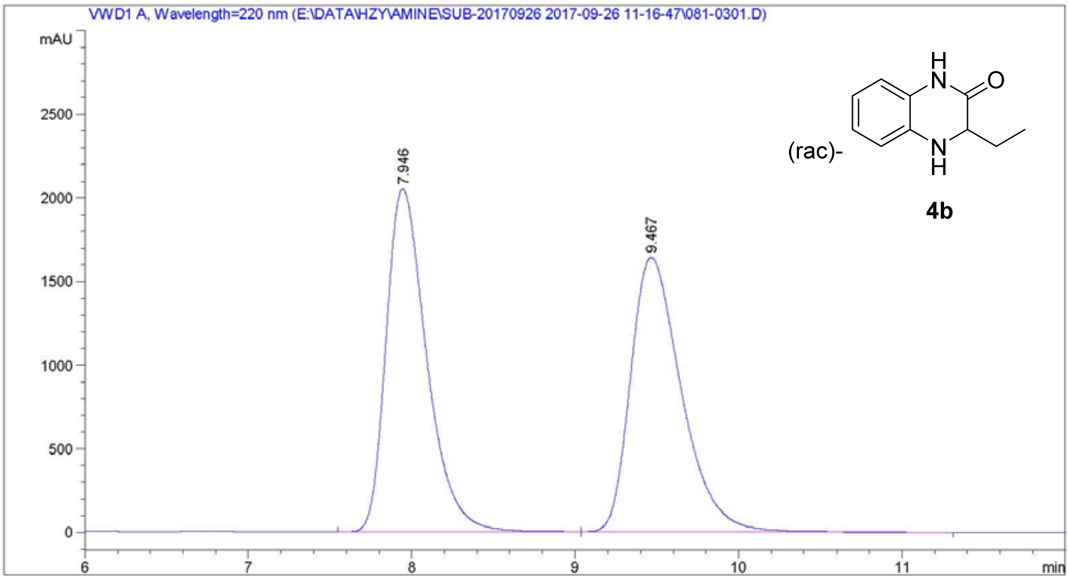

===

Area Percent Report

===

Sorted By : Signal
Multiplier : 1.0000
Dilution : 1.0000
Use Multiplier & Dilution Factor with ISTDs

Signal 1: VWD1 A, Wavelength=220 nm

Peak #	RetTime [min]	Type	Width [min]	Area [mAU*s]	Height [mAU]	Area %
1	7.946	VB	0.2637	3.52535e4	2049.50537	49.9302
2	9.467	BB	0.3313	3.53521e4	1641.07715	50.0698
Totals :				7.06057e4	3690.58252	

Data File E:\DATA\HZY\AMINE\SUB-20170926 2017-09-26 11-16-47\071-0201.D
Sample Name: H-1-EE

===

Acq. Operator : SYSTEM Seq. Line : 2
Acq. Instrument : 1260HPLC-VWD Location : Vial 71
Injection Date : 9/26/2017 11:31:23 AM Inj : 1
Inj Volume : 5.000 µl
Acq. Method : E:\DATA\HZY\AMINE\SUB-20170926 2017-09-26 11-16-47\VWD-OD(1-2)-80-20-1ML-5UL-220NM-25MIN.M
Last changed : 9/26/2017 11:16:47 AM by SYSTEM
Analysis Method : E:\DATA\HZY\AMINE\SUB-20170926 2017-09-26 11-16-47\VWD-OD(1-2)-80-20-1ML-5UL-220NM-25MIN.M (Sequence Method)
Last changed : 9/26/2017 2:40:58 PM by SYSTEM
(modified after loading)
Additional Info : Peak(s) manually integrated

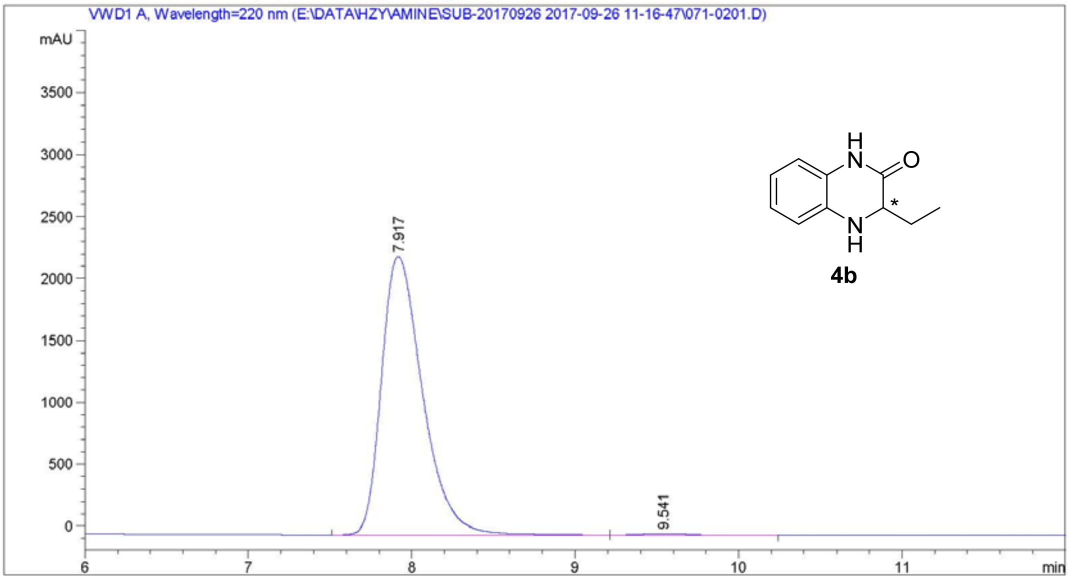

===

Area Percent Report

===

Sorted By : Signal
Multiplier : 1.0000
Dilution : 1.0000
Use Multiplier & Dilution Factor with ISTDs

Signal 1: VWD1 A, Wavelength=220 nm

Peak #	RetTime [min]	Type	Width [min]	Area [mAU*s]	Height [mAU]	Area %
1	7.917	BV	0.2672	3.91830e4	2249.79248	99.3748
2	9.541	VB	0.3517	246.52602	10.62627	0.6252

Totals : 3.94295e4 2260.41875

Data File C:\CHEM32\1\DATA\SNAPSHOT.D
Sample Name:

===

Acq. Operator : SYSTEM Seq. Line : 5
Location : Vial 32
Injection Date : 7/26/2017 11:17:33 AM Inj : 1
Acq. Method : VWD-AD(1-2)-80-20-1.0ML-1UL-210NM-25MIN.M
Analysis Method : E:\METHODS\HZY\VWD-AD(1-2)-80-20-1.0ML-1UL-210NM-25MIN.M
Last changed : 7/26/2017 11:40:17 AM by SYSTEM
(modified after loading)
Additional Info : Peak(s) manually integrated

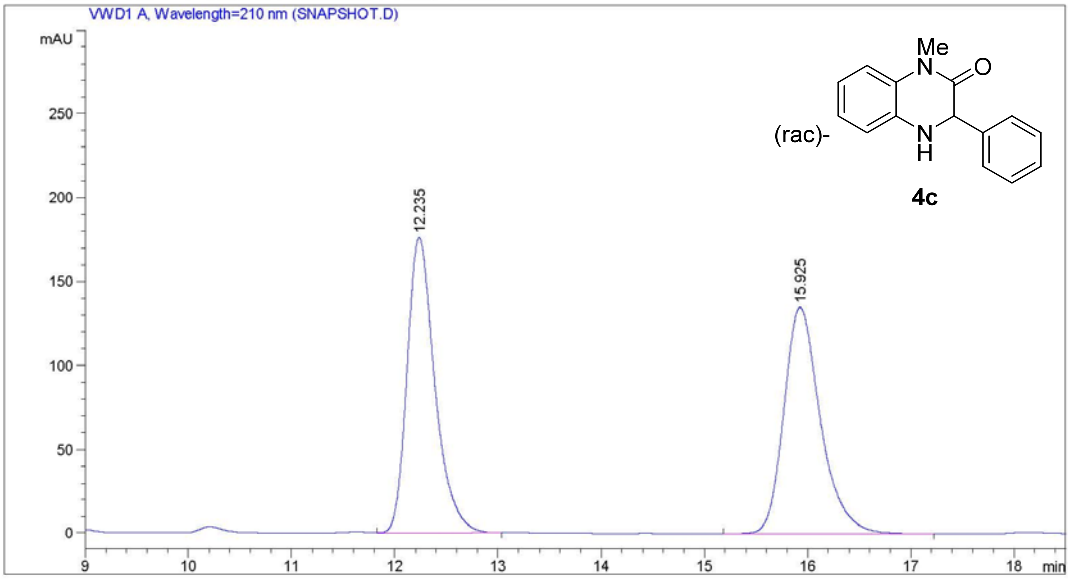

===

Area Percent Report

===

Sorted By : Signal
Multiplier : 1.0000
Dilution : 1.0000
Use Multiplier & Dilution Factor with ISTDs

Signal 1: VWD1 A, Wavelength=210 nm

Peak #	RetTime [min]	Type	Width [min]	Area [mAU*s]	Height [mAU]	Area %
1	12.235	VB	0.2831	3296.98560	176.39929	49.7965
2	15.925	BB	0.3729	3323.93262	135.14771	50.2035

Totals : 6620.91821 311.54700

===

*** End of Report ***

Data File E:\DATA\HZY\AMINE\LWD-2-160-2 2017-07-26 10-30-16\031-0401.D
Sample Name: N-ME

===

Acq. Operator : SYSTEM Seq. Line : 4
Acq. Instrument : 1260HPLC-VWD Location : Vial 31
Injection Date : 7/26/2017 10:51:50 AM Inj : 1
Inj Volume : 1.000 µl
Acq. Method : E:\DATA\HZY\AMINE\LWD-2-160-2 2017-07-26 10-30-16\VWD-AD(1-2)-80-20-1.0ML-1UL-210NM-25MIN.M
Last changed : 7/26/2017 10:31:56 AM by SYSTEM
Analysis Method : E:\DATA\HZY\AMINE\LWD-2-160-2 2017-07-26 10-30-16\VWD-AD(1-2)-80-20-1.0ML-1UL-210NM-25MIN.M (Sequence Method)
Last changed : 7/26/2017 11:37:48 AM by SYSTEM
(modified after loading)
Additional Info : Peak(s) manually integrated

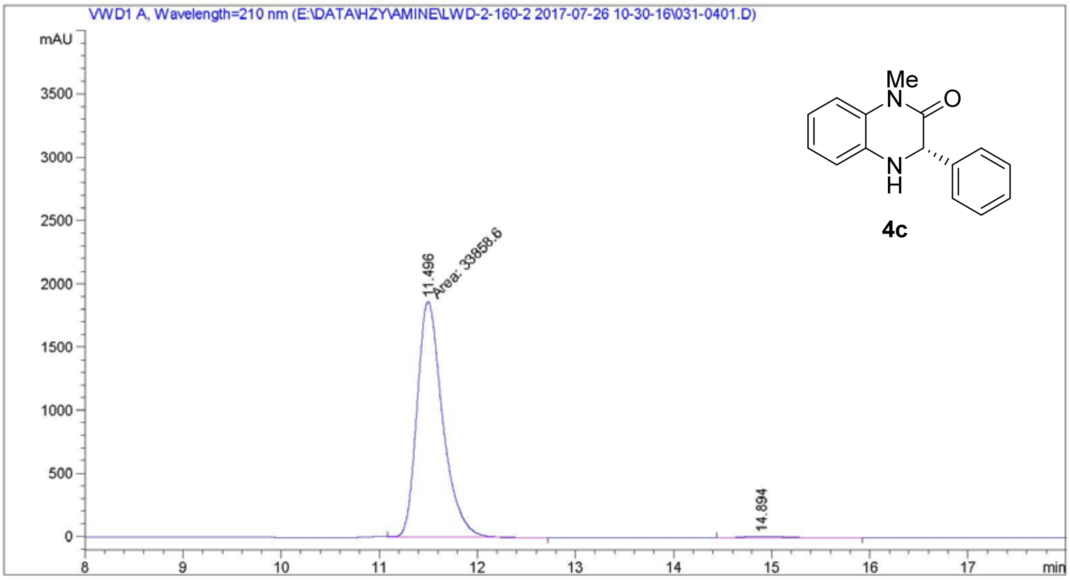

===

Area Percent Report

===

Sorted By : Signal
Multiplier : 1.0000
Dilution : 1.0000
Use Multiplier & Dilution Factor with ISTDs

Signal 1: VWD1 A, Wavelength=210 nm

Peak #	RetTime [min]	Type	Width [min]	Area [mAU*s]	Height [mAU]	Area %
1	11.496	MM	0.3023	3.38586e4	1866.80188	99.1713
2	14.894	BB	0.3561	282.94363	11.95377	0.8287

Totals : 3.41415e4 1878.75565

Data File D:\DATA\HZY\AMINE\20190220 2019-02-20 12-06-00\017-1101.D
Sample Name: S-PdC

===

Acq. Operator : Seq. Line : 11
Acq. Instrument : Instrument 2 Location : Vial 17
Injection Date : 2/20/2019 4:17:10 PM Inj : 1
Inj Volume : 1.000 µl
Acq. Method : D:\DATA\HZY\AMINE\20190220 2019-02-20 12-06-00\DAD-OJ(1-6)-80-20-1ML-1UL-ALL-50MIN.M
Last changed : 2/20/2019 12:04:34 PM
Analysis Method : D:\METHOD\HZY\DAD-OJ(1-6)-80-20-1ML-1UL-ALL-50MIN.M
Last changed : 2/20/2019 5:36:09 PM
(modified after loading)
Additional Info : Peak(s) manually integrated

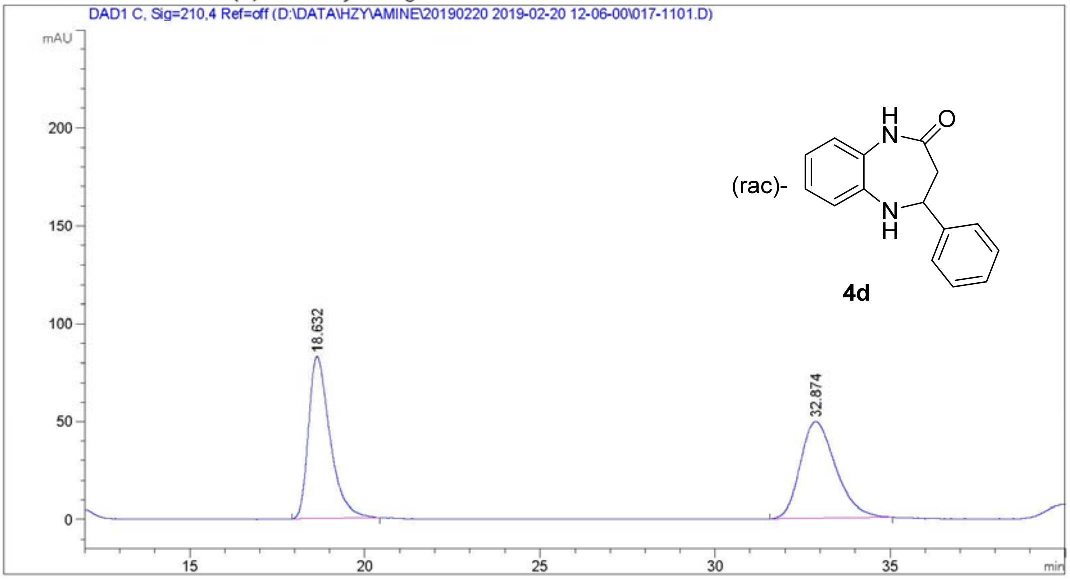

===

Area Percent Report

===

Sorted By : Signal
Multiplier : 1.0000
Dilution : 1.0000
Use Multiplier & Dilution Factor with ISTDs

Signal 1: DAD1 C, Sig=210,4 Ref=off

Peak #	RetTime [min]	Type	Width [min]	Area [mAU*s]	Height [mAU]	Area %
1	18.632	BB	0.6099	3546.97827	83.10571	50.2678
2	32.874	BB	0.8443	3509.19238	49.66652	49.7322

Totals : 7056.17065 132.77223

Data File D:\DATA\HZY\AMINE\20190220 2019-02-20 12-06-00\016-1001.D
Sample Name: S-EE

===

Acq. Operator : Seq. Line : 10
Acq. Instrument : Instrument 2 Location : Vial 16
Injection Date : 2/20/2019 3:26:17 PM Inj : 1
Inj Volume : 1.000 µl
Acq. Method : D:\DATA\HZY\AMINE\20190220 2019-02-20 12-06-00\DAD-OJ(1-6)-80-20-1ML-1UL-ALL-50MIN.M
Last changed : 2/20/2019 12:04:34 PM
Analysis Method : D:\METHOD\HZY\DAD-OJ(1-6)-80-20-1ML-1UL-ALL-50MIN.M
Last changed : 2/20/2019 5:37:55 PM
(modified after loading)
Additional Info : Peak(s) manually integrated

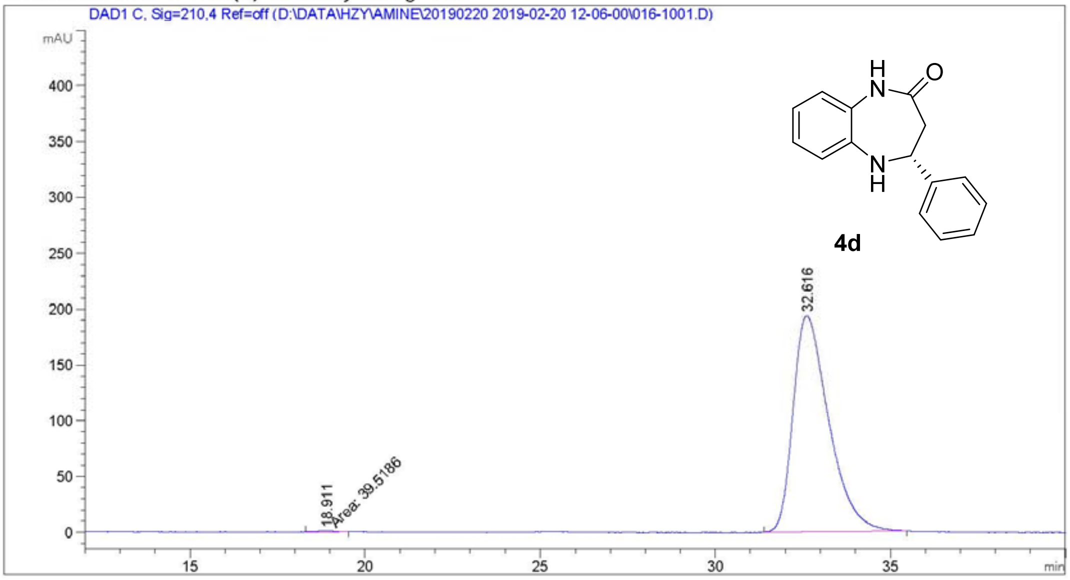

===

Area Percent Report

===

Sorted By : Signal
Multiplier : 1.0000
Dilution : 1.0000
Use Multiplier & Dilution Factor with ISTDs

Signal 1: DAD1 C, Sig=210,4 Ref=off

Peak #	RetTime [min]	Type	Width [min]	Area [mAU*s]	Height [mAU]	Area %
1	18.911	MM	0.6028	39.51863	1.09261	0.2881
2	32.616	BB	1.0034	1.36798e4	193.83629	99.7119

Totals : 1.37193e4 194.92890
